# Investigating NFE2L1 activators for targeted protein aggregate clearance: a follow-up study

**DOI:** 10.1039/d5md00584a

**Published:** 2025-10-22

**Authors:** Zuzana Smahelova, Lucie Svobodova, Jindrich Sedlacek, Michael Adamek, Marketa Pimkova Polidarova, Pavel Majer, Ales Machara, Klara Grantz Saskova

**Affiliations:** a Institute of Organic Chemistry and Biochemistry of the Czech Academy of Sciences Flemingovo n. 542/2 160 00 Prague 6 Czech Republic ales.machara@uochb.cas.cz; b Department of Genetics and Microbiology, Charles University and Research Center BIOCEV Prumyslova 595 252 50 Vestec Czech Republic saskova2@natur.cuni.cz; c Department of Organic Chemistry, Charles University Hlavova 2030/8 Prague 2 128 00 Czech Republic

## Abstract

Disruption of protein homeostasis (proteostasis), whether by acute proteotoxic stress or chronic expression of mutant proteins, can lead to the accumulation of toxic protein aggregates. Such aggregation is a hallmark of numerous diseases and is often associated with impaired protein clearance mechanisms. The transcription factor nuclear factor erythroid 2-related factor 1 (encoded by *NFE2L1*, also known as Nrf1) plays a central role in restoring proteostasis by increasing proteasome synthesis. Therefore, pharmacological activation of NFE2L1 under non-stress conditions represents a promising therapeutic strategy for neurodegenerative and other proteostasis-related diseases. In our previous study, we identified bis(phenylmethylene)cycloalkanone derivatives as NFE2L1 activators capable of inducing proteasome subunit expression, increasing heat shock protein levels, and stimulating autophagy. Building upon these findings, we have now developed a new library of structurally related compounds to identify novel more potent NFE2L1 activators. By systematically examining how specific chemical substitutions affect NFE2L1 activation, this work advances our understanding of the structure–activity relationships within this pathway.

## Abstract 2

NFE2L1 activator enhancing proteasome, heat shock, and autophagy pathways.

## Graphical abstract

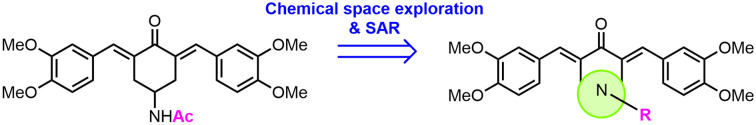

## Introduction

Proteins are essential to virtually all cellular functions across organisms, and their proper synthesis, folding, and degradation are tightly regulated to maintain cellular homeostasis.^1^ A protein's native structure is determined by its amino acid sequence. Many require assistance from molecular chaperones and other cellular factors to fold correctly and within a biologically relevant timeframe. Moreover, functional requirements often necessitate structural flexibility or intrinsically disordered regions, increasing susceptibility to misfolding and aggregation.^2–4^ Even typically stable proteins can unfold and aggregate when exposed to stressors. Misfolded or superfluous proteins must be efficiently degraded to prevent cellular toxicity. Therefore, proteostasis depends not only on the precise regulation of protein synthesis and folding but also on the maintenance of proper conformation, controlled abundance and localization, and timely degradation.^1,3^ Cells rely on two major systems to eliminate misfolded, aggregated, or nonfunctional proteins: the ubiquitin–proteasome system (UPS) and the autophagy–lysosomal pathway (ALP).^5,6^

The ubiquitin–proteasome system (UPS) is a central component of the cellular machinery responsible for maintaining proteostasis. It facilitates the targeted degradation of misfolded, damaged, or regulatory proteins and accounts for the turnover of approximately 80% of all cellular proteins.^5,7^ In addition to general proteostasis, the proteasome is essential for proper nervous system function, where it regulates diverse aspects of neuronal survival and activity.^8,9^ Increasing evidence implicates dysfunction of the UPS in the pathogenesis of several neurodegenerative diseases (NDs), including Alzheimer's disease (AD), Parkinson's disease (PD), and Huntington's disease (HD).^10^ These diseases are characterized by the accumulation of aggregation-prone proteins such as tau, huntingtin, α-synuclein and β-amyloid peptide, due to impaired UPS-mediated clearance.^11–15^ A prevailing hypothesis suggests that proteins containing repetitive amino acid sequences may evade proteasomal degradation, contributing to toxic intracellular aggregation.^8^ Neurodegenerative disorders can also stem from genetic mutations affecting components of the UPS. For instance, mutations in the E3 ubiquitin ligase gene parkin are linked to familial forms of PD,^16–18^ while mutations in VCP (valosin-containing protein, also known as p97) are associated with inclusion-body myositis (IBM), characterized by protein aggregates in muscle and brain tissue.^19,20^ Many NDs are age-related and are thought to emerge from cumulative, stress-induced defects in cellular quality control systems.^8,21^

In response to proteotoxic stress, cells activate adaptive mechanisms to restore proteostasis.^22–24^ A key component of this response is the regulation of proteasome function at multiple levels, including transcription, translation, assembly, and post-translational modifications.^24,25^ The transcription factor nuclear factor erythroid 2-related factor 1 (*NFE2L1*, also known as Nrf1 in mammals) is essential for the coordinated transcriptional upregulation of all proteasome subunit genes.^22,26,27^ In addition, NFE2L1 controls the expression of genes involved in protein quality control, including molecular chaperones and key components of the autophagy–lysosomal pathway, such as p62/SQSTM1 and GABARAPL1.^28–30^ Under normal, non-stressed conditions, NFE2L1 is anchored *via* its N-terminal domain to the endoplasmic reticulum (ER) membrane and is constitutively degraded through the ER-associated degradation (ERAD) pathway. This process is mediated by the ER-resident E3 ubiquitin ligase HRD1 and the AAA-ATPase VCP/p97.^23^ Upon proteasome malfunction, NFE2L1 escapes ERAD by undergoing deglycosylation *via N*-glycanase 1 (NGLY1), retrotranslocation from the ER membrane by VCP/p97, and proteolytic cleavage by DNA damage inducible 1 homolog 2 (DDI2).^26,29,31^ The activated form of NFE2L1 is then translocated to the nucleus, where it binds to antioxidant response elements (AREs/EpREs) in cooperation with small musculoaponeurotic fibrosarcoma (MAF) proteins. These regulatory elements are located in the promotor regions of genes encoding proteasome subunits, UPS-associated factors, and various detoxification and metabolic enzymes.^28,32^

Brain-specific deletion of NFE2L1 in mice results in age-related accumulation of polyubiquitinated protein aggregates and neuronal atrophy, recapitulating hallmark features of neurodegenerative disorders.^33,34^ Similarly, *NGLY1* deficiency in both human and rodent models leads to neurodegenerative phenotypes accompanied by aggregate formation, highlighting the critical role of this enzyme in NFE2L1 activation and proteostasis maintenance.^35–37^ Moreover, reduced NFE2L1 expression has been observed in the dopaminergic neurons of Parkinson's disease patients, where its loss exacerbates oxidative stress and impairs midbrain neuronal integrity.^38^

Collectively, these findings underscore NFE2L1 as a key regulator of proteasome biogenesis and a promising therapeutic target for restoring proteolytic capacity in aggregation disorders.^22,39,40^ Among current pharmacological approaches, the curcumin derivative ASC-JM17 has emerged as a notable candidate for modulating both the NFE2L1 and NFE2L2 pathways.^41,42^ This compound has received orphan drug designation by the European Medicines Agency (EMA; EU/3/16/1639) for the treatment of Kennedy's disease.

In our previous work, we identified a series of compounds capable of activating NFE2L1-dependent downstream events, including proteasome subunit synthesis, heat shock protein expression, and autophagy induction.^43^ These compounds enhanced proteasome activity and significantly reduced the size and number of protein aggregates in both cell culture and *Caenorhabditis elegans* models, without inducing cellular stress or disrupting the UPS.

Building on these findings, the present study aimed to expand our compound library and discover novel, potent NFE2L1 activators. The new series of compounds were synthesized based on structural modifications of our initial lead compound, RUN-47. We focused on systematically varying substituents in the chemical space surrounding the nitrogen atom to NFE2L1 activation. This resulting library offers new candidates for further validation and represents a promising step toward the development of therapeutic strategies targeting a broad range of proteinopathies, including debilitating neurodegenerative disorders.

## Results and discussion

### Design and synthesis of an extended library of NFE2L1 activators

**Scheme 1:**
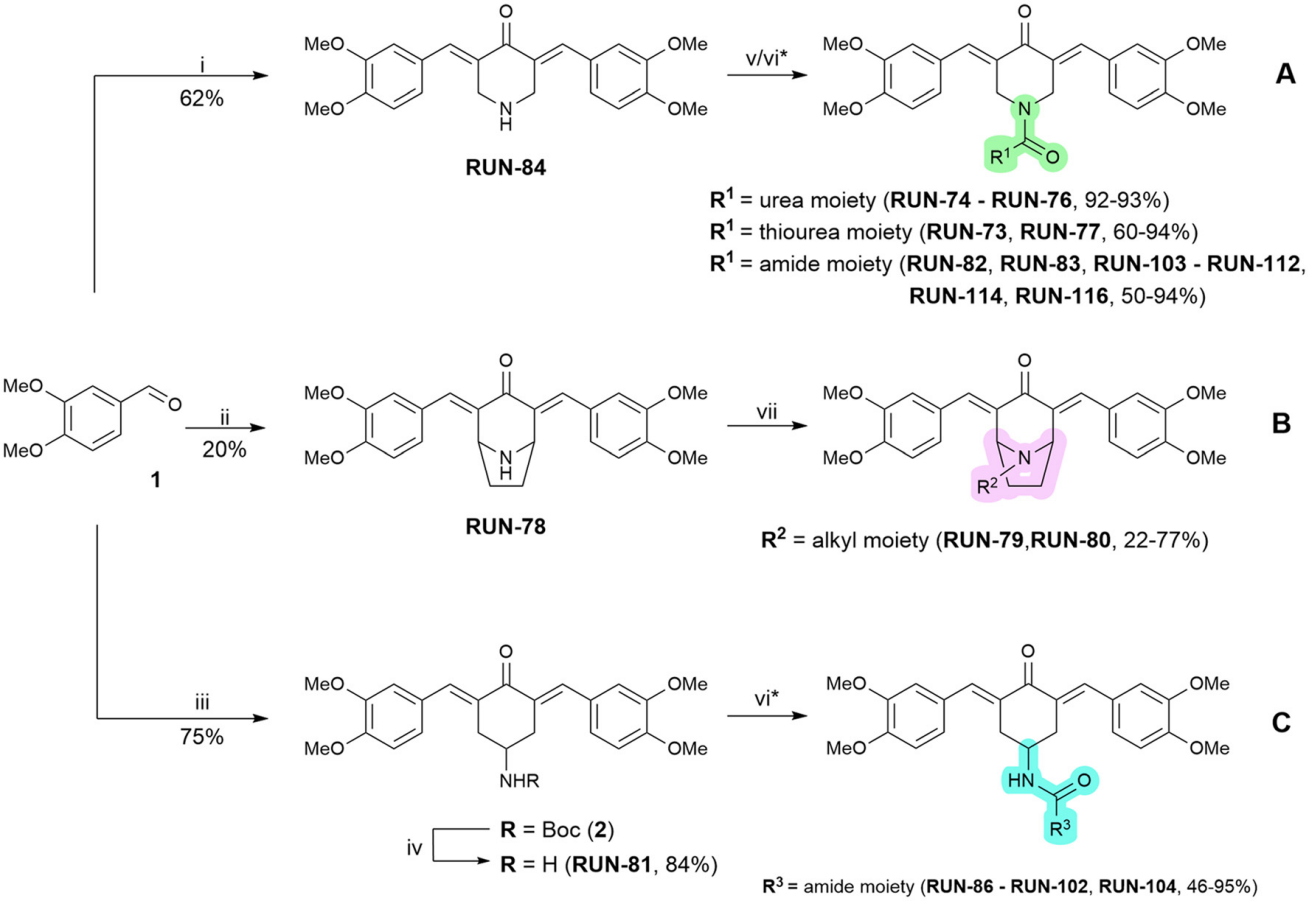
Reagents and reaction conditions: (i) 4-piperidone monohydrate hydrochloride, HCl (4 M in 1,4-dioxane), AcOH, ice-cooled → r.t., overnight; (ii) nortropinone hydrochloride, HCl (4 M in 1,4-dioxane), AcOH, ice-cooled → r.t., 2 days; (iii) *tert*-butyl (4-oxocyclohexyl)carbamate, NaOH (20% aq.), EtOH, r.t., overnight; (iv) TFA, DCM, 0 °C → r.t., 1.5 h; (v) isocyanate/isothiocyanate, Et_3_N, THF, r.t., overnight; (vi) carboxylic acid, Et_3_N, HBTU, DMF, r.t., 1 h – 2 days; (vii) alkylating agent, K_2_CO_3_, DMF, r.t., 1.5 h. * in the case of compounds RUN-85, RUN-92, RUN-96, RUN-100, RUN-104, RUN-113, RUN-115 and RUN-117: (vi) a) carboxylic acid, Et_3_N, HBTU, DMF, r.t., 1 h – 2 days, b) TFA, DCM, 0 °C → r.t., 1.5 h.

In our previous work, we identified RUN-47 (*N*-(3,5-bis((*E*)-3,4-dimethoxybenzylidene)-4-oxocyclohexyl)acetamide) as the front runner activator of the NFE2L1 pathway.^43,44^ Based on its structure, we employed a rational drug design approach to expand the compound library, preparing derivatives with three distinct scaffolds to study the influence of various moieties in the nitrogen region. Scaffold A contains a 4-piperidinone core, scaffold B explores the structural motif of the nortropinone core, and scaffold C features the original core from RUN-47 4-aminocyclohexanone (Scheme 1).

The synthesis of all three scaffolds started with the preparation of core structures *via* acid-catalyzed (cores A and B) or base-catalyzed (core C) aldol reactions between 3,4-dimethoxybenzaldehyde (1) and the corresponding ketones, resulting in amines RUN-84 and RUN-78 and Boc-protected amine 2 that had to be deprotected by a mixture of TFA/DCM, to obtain amine RUN-81. Final derivatives based on core A incorporated three distinct functional moieties: urea, thiourea, and amide. Urea and thiourea derivatives were prepared by reaction of various isocyanates and isothiocyanates with the amine RUN-84 in THF overnight. Using this procedure, we obtained five final compounds (RUN-73 to RUN-77). A large part of the library consisted of amide derivatives that were synthesized by amidation reactions involving the direct coupling of a wide variety of carboxylic acids with amine RUN-84 using HBTU as a coupling agent in combination with triethylamine, affording seventeen final products (RUN-82, RUN-83, RUN-103 to RUN-112, RUN-114, and RUN-116). After the coupling reaction, crude intermediates bearing Boc-protected groups were treated with a TFA/DCM mixture to remove the protecting group, yielding four final compounds: RUN-85, RUN-113, RUN-115 and RUN-117. For the second group of final derivatives with core C, which contains only an amide moiety, a similar approach was employed to prepare amide analogues *via* the amidation reaction using amine RUN-81. Eighteen final compounds, RUN-86–RUN-102 and RUN-104, were obtained. The final group of activators comprised two amino analogues, RUN-79 and RUN-80, featuring core B. These compounds were synthesized *via* a two-step procedure, involving the preparation of appropriate alkylating agents from alcohol using the Appel reaction, followed by the *N*-alkylation of RUN-78 (Scheme 1). Variations of R^1^, R^2^ and R^3^ moieties are detailed in Table 1.

**Table 1:** Overview of structural variations in the R moieties of all library derivatives, their effects on NFE2L1 pathway activation in the luciferase assay, and associated cytotoxicity

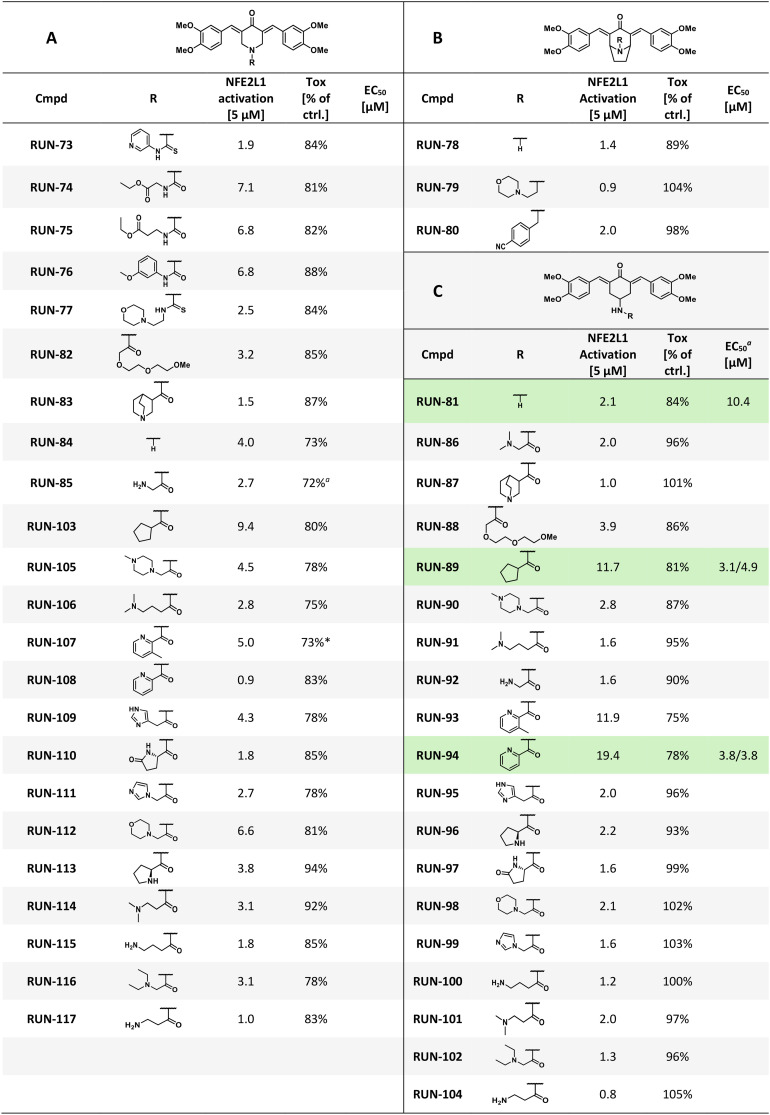

a EC_50_ values for selected compounds calculated using a four-parameter logistic model or bell-shaped dose–response model. For more information, see the SI Results, Fig. S1.

### Biological evaluation of the extended library of NFE2L1 activators

#### Selected RUN compounds activate the *NFE2L1* pathway without inducing oxidative stress, proteasome inhibition, or UPR activation

**Fig. 1:**
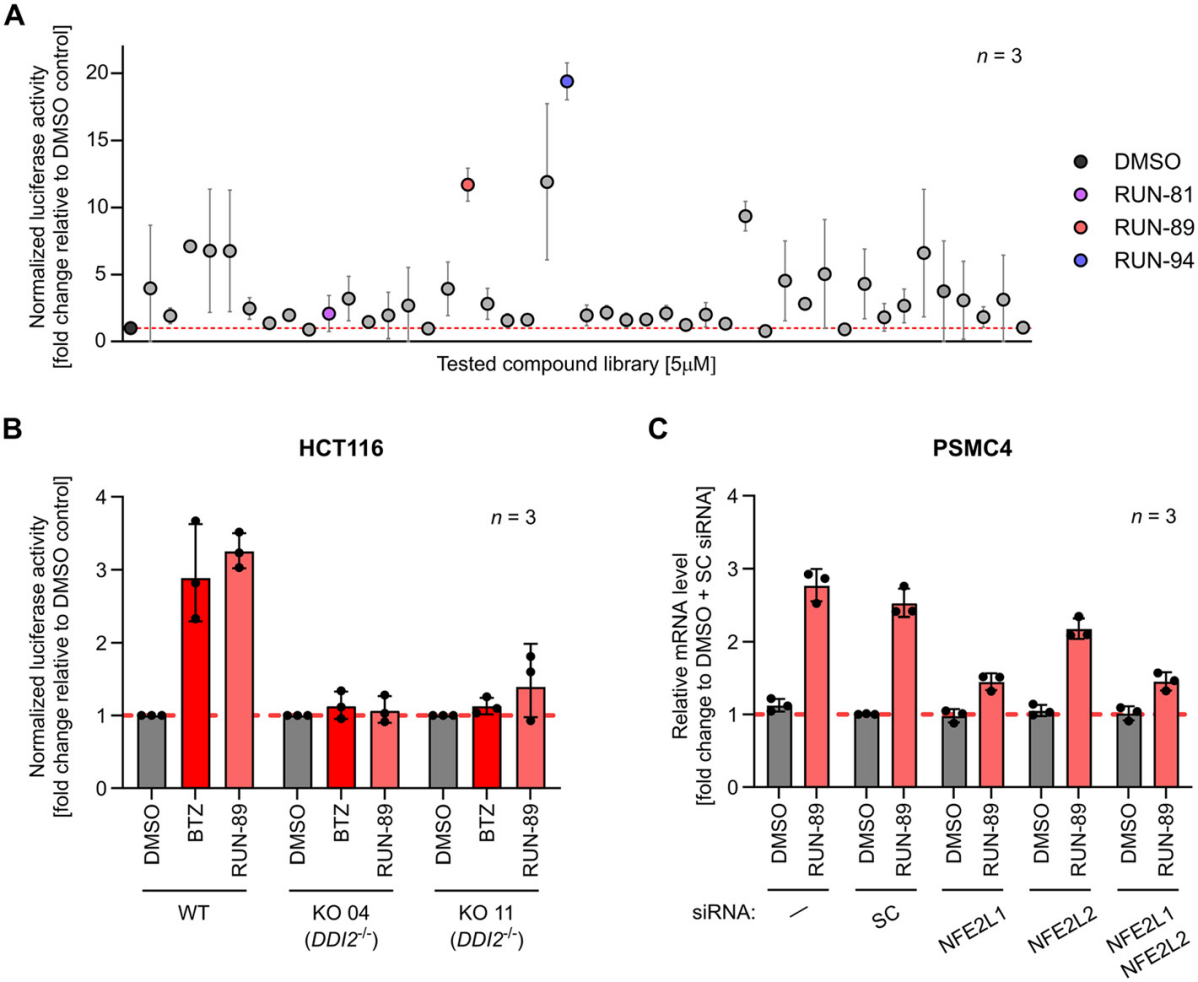
(A) Targeted library screening of RUN compounds using the 3×PSMA4-ARE-LUC dual reporter assay in HEK293 cells. Cells were exposed to RUN compounds (5 μM) for 16 hours, and luciferase activity was measured. Data are presented as the mean ± SD from three independent experiments (*n* = 3). Results are shown as a fold change in luminescence relative to DMSO-treated cells. Selected hit compounds are highlighted in distinct colours. (B) RUN-89 specifically activates the NFE2L1 pathway. The DDI2 knockout (KO) and parental (WT) HCT116 cells were transfected with the 3×PSMA4-ARE-LUC reporter and treated with 0.5 μM bortezomib (BTZ) and 5 μM RUN-89 for 16 hours. Data are presented as the Geomean ± GeoSD of three independent experiments (*n* = 3). Statistical analysis was performed on log_2_-transformed values using two-way repeated-measures ANOVA followed by Šídak's multiple comparison test. The results of the analysis are shown in SI Table S3. (C) RUN-89 selectively triggers downstream signaling events associated with NFE2L1 gene expression. HEK293 cells were transfected with lipid nanoparticles (LNPs) carrying siRNAs targeting NFE2L1 or NFE2L2. LNPs formulated with non-targeting siRNA served as a negative control. Following 24 hours of incubation, cells were treated with 7.5 μM RUN-89 or DMSO (vehicle) for an additional 16 hours. Expression of the proteasomal subunit gene *PSMC4* was then quantified by RT-qPCR. Data are shown as the Geomean ± GeoSD from three independent biological replicates (*n* = 3). Log_2_-transformed data were analyzed using two-way repeated-measures ANOVA with the Geisser–Greenhouse correction, followed by Tukey's multiple comparison test with individual variances computed for each comparison. The results of the analysis are shown in SI Table S4. Additional results for *NFE2L1*, *NFE2L2*, *PSMB7*, and *PSMD12* are presented in SI Fig. S10.

In our previous work,^43^ we developed a library of NFE2L1 stimulators using a cell-based luciferase reporter assay. This system was based on three tandem copies of the antioxidant response element (ARE) derived from the NFE2L1 target gene proteasome subunit alpha type-4 (3xPSMA4, PSMA4-ARE-LUC). It was designed to detect the proteasome “bounce-back” response induced by proteasome inhibitors such as MG132 or bortezomib.^22,43,45,46^ The reporter assay was adapted to a 384-well format and used to evaluate a new set of RUN compounds designed around the structural framework of the RUN-47 activator. We focused on identifying compounds that increased the 3×PSMA4-ARE-LUC signal (Fig. 1A), while simultaneously assessing cytotoxicity (see the SI, Fig. S2). Scaffold A derivatives, based on a 4-piperidinone core, showed modest activity overall, with fold change values typically ranging from 1.5 to 6.8. Within this series, simple amide derivatives displayed moderate activation, exemplified by RUN-103, which, bearing a cyclopentane carboxamide substituent, reached a fold change of 9.4. In contrast, thiourea- and urea-containing derivatives generally produced only weak to intermediate responses, rarely exceeding a fold change of 7. The unsubstituted analogue RUN-84 displayed a fold change of 4.0, indicating that additional substitution is required to enhance potency. Collectively, scaffold A provided valuable structure–activity information but did not yield top-tier activators.

Scaffold B, constructed around a nortropinone framework, performed poorly. All three tested derivatives displayed only marginal activation (fold change 0.9–2.0), and in some cases their tolerability was limited. This suggested that the nortropinone core is inherently less compatible with productive NFE2L1 activation, and further elaboration of this scaffold was not pursued.

By contrast, scaffold C, derived from the original RUN-47 4-aminocyclohexanone core, delivered the most promising results. The unsubstituted amine RUN-81 served as a minimal reference, exhibiting only low activity (fold change 2.1, EC_50_ 10.4 μM). Systematic introduction of amide substituents revealed marked differences in potency. Aliphatic derivatives were generally weak, as seen with glycine (RUN-92, fold change 1.6) or short-chain amino acids (RUN-91 and RUN-100). In contrast, heteroaromatic substituents significantly enhanced activation. RUN-93, bearing a methylpyridine carboxamide, reached a fold change of 11.9, while RUN-94, with a pyridine carboxamide, emerged as the most potent analogue with a fold change of 19.4 and an EC_50_ of 3.8 μM. This result highlights the favorable role of heteroaromatic rings in promoting activity. Similarly, RUN-89, containing a cyclopentane carboxamide, produced strong activation (fold change 11.7, EC_50_ 3.1 μM). In contrast, bulky or strongly basic moieties, such as quinuclidine (RUN-87) or morpholine (RUN-98), resulted in reduced activity.

Overall, these findings establish a clear scaffold preference, with scaffold C markedly outperforming scaffolds A and B. Within scaffold C, pyridyl substituents gave the strongest activation, and cyclopentane derivatives retained high potency, whereas amino acid-derived or basic substituents were weak. The identification of RUN-94 and RUN-89 as potent analogues, together with RUN-81 as a minimal reference, defines a coherent SAR pattern that underscores the privileged nature of the RUN-47 core.

For three representative compounds, RUN-81, RUN-89, and RUN-94, we additionally determined EC_50_ values using the same reporter assay, with the corresponding dose–response curves and fitted parameters provided in SI Fig. S1.

We next examined whether the selected RUN compounds activate the NFE2L1 pathway in a stress-independent manner, while preserving normal ubiquitin-dependent proteasomal degradation. This distinction is crucial, as the therapeutic benefit of NFE2L1 activation lies in its ability to alleviate proteotoxic stress and target disease-related proteins for degradation without compromising normal proteostasis. To assess this, U2OS cells stably expressing the short-lived fluorescent reporter Ub-G76V-GFP were treated with various concentrations of RUN compounds. The selected hits did not interfere with GFP degradation compared to the vehicle-treated controls, indicating preserved proteasome function (see the SI, Fig. S3). We also examined whether compound-induced activation of NFE2L1 could be attributed to the generation of reactive oxygen species (ROS). To test this, the 3×PSMA4-ARE-LUC reporter assay was performed in the presence or absence of the antioxidant *N*-acetyl-l-cysteine (NAC). A decrease in reporter activity upon NAC treatment would indicate pathway activation through oxidative stress. However, NFE2L1 activation by our compounds was unaffected by NAC, indicating that their activity is not mediated by oxidative stress or by indirect NFE2L2 involvement through oxidative modification of KEAP1 cysteine residues (see the SI, Fig. S4).

To clarify whether the representative compound RUN-89 activates the NFE2L1-specific luciferase reporter independently of the KEAP1–NFE2L2 pathway, we used the 3×PSMA4-ARE-LUC dual reporter in previously published DDI2 knockout HCT116 cells (from ATCC CCL-247 (RRID:CVCL_0291)), in which the protease essential for NFE2L1 activation is disrupted. In these DDI2-deficient cells, neither the proteasome inhibitor bortezomib nor RUN-89 induced reporter activity, in contrast to wild-type cells (Fig. 1B). These findings indicate that RUN-89 activates the reporter construct through NFE2L1.

To further confirm that RUN-89 acts through NFE2L1-dependent signalling, we investigated the effect of selective knockdown of *NFE2L1* and *NFE2L2* on the induction of proteasomal genes. HEK293 cells (from ATCC CCL-247 (RRID:CVCL_0291)) were transfected with lipid nanoparticles (LNPs) carrying siRNAs targeting either *NFE2L1* or *NFE2L2*, while non-targeting siRNA was used as a control. After treatment with RUN-89, transcript levels of several proteasome subunit genes were analyzed by RT-qPCR. Silencing of *NFE2L1*, but not *NFE2L2*, markedly reduced the induction of *PSMC4* expression by RUN-89 (Fig. 1C), and a similar pattern was observed for *PSMB7* and *PSMD12* (SI Fig. S10). These results provide direct evidence that the stimulatory activity of RUN-89 on proteasomal gene expression is mediated primarily through the NFE2L1 pathway rather than NFE2L2.

Next, we investigated whether the selected RUN compounds activate the NFE2L1 transcriptional program without triggering the unfolded protein response (UPR), a crucial cellular process induced by the accumulation of unfolded proteins in the endoplasmic reticulum (ER), potentially leading to ER stress. To monitor this, an alternative reporter was used to assess the activation of IRE1/XBP1s.^47^ IRE1 is a bifunctional serine/threonine kinase and endoribonuclease embedded in the ER membrane, playing a central role in UPR signaling. Its RNase activity facilitates the splicing of X-box binding protein 1 (XBP1) mRNA. The spliced XBP1 acts as a transcription factor, promoting the re-establishment of ER homeostasis by upregulating genes involved in the ER-associated degradation (ERAD) pathway.^48–50^ The reporter contains Gaussia luciferase positioned out of frame downstream of the XBP1 splice site, ensuring that active luciferase translation occurs only in the presence of ER stress.^51,52^ Cells transfected with this reporter were treated with RUN compounds at 1, 5, and 10 μM, and luciferase activity was measured. As shown in the SI (Fig. S5), none of the tested compounds induced significant reporter activation, indicating that ER stress was not triggered at the concentrations used. These results confirm that NFE2L1 activation by RUN compounds occurs independently of the UPR.

#### Selected RUN compounds induce *NFE2L1*-dependent gene expression and upregulate proteotoxic stress response proteins

**Fig. 2:**
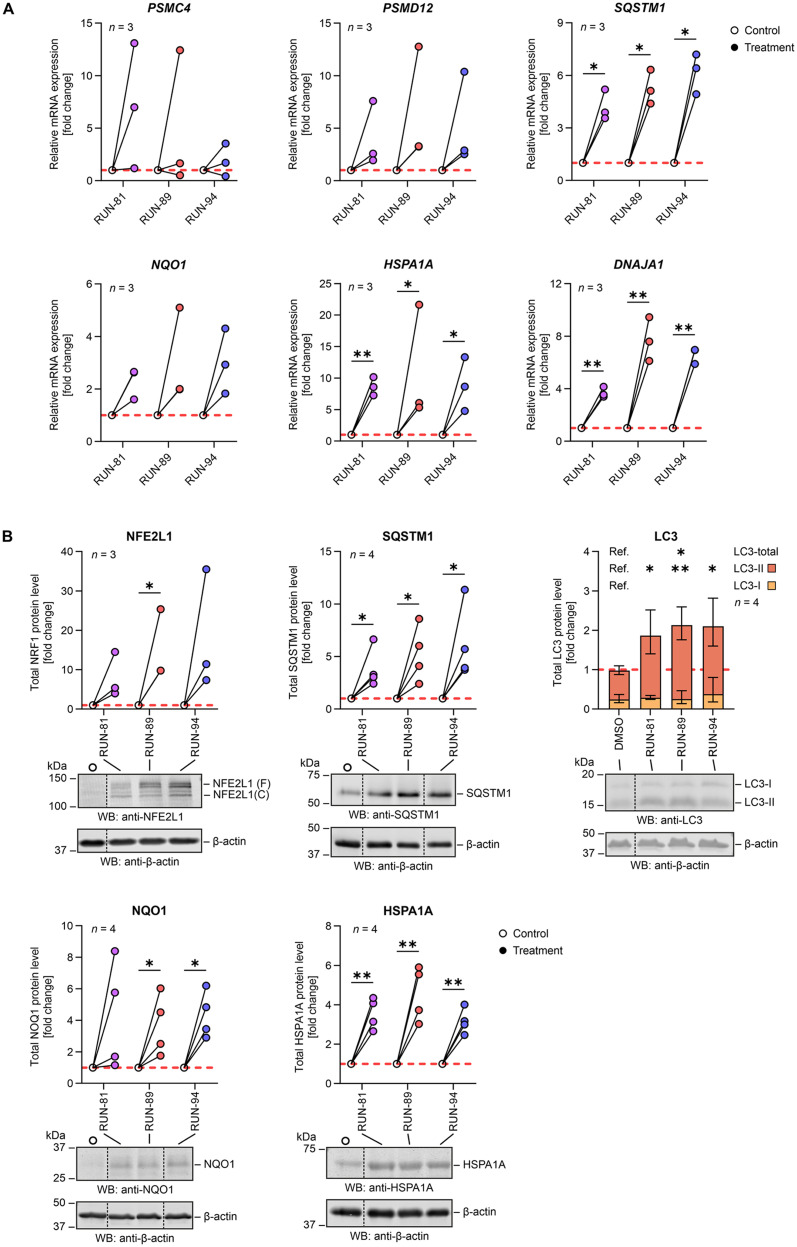
(A) qPCRs of HEK293 cells treated with RUN compounds (7.5 μM) for 16 hours. The extracted RNA was reverse transcribed into cDNA and utilized for RT-qPCR (primers are listed in the SI, Table S1); the mRNA levels of RPLP0 were used for normalization. The experiment was performed in three biological replicates (*n* = 3). (B) Immunoblot analysis of proteins NFE2L1/TCF11/NRF1, SQSTM1/p62, LC3, NQO1 and HSPA1A/HSP70-1 in HEK293 cells after 16 h treatment with 7.5 μM RUN compounds. The protein levels were quantified by measuring the integrated fluorescence of the corresponding protein bands and normalized to the β-actin load. The full-length (F) and cleaved (C) forms of NFE2L1 were analyzed together. The experiment was performed in four biological replicates (*n* = 4) except for NFE2L1 (*n* = 3). (A and B) Results of RUN compounds were compared to the DMSO control using ratio paired *t*-tests corrected for multiple comparisons with Holm–Šídák's method (*α* = 0.05). *p < 0.05, **p < 0.01. Detailed results of the analysis are shown in SI Tables S5 and S6.

NFE2L1 enhances the transcription of genes encoding proteasome subunits and molecular chaperones such as heat shock proteins (HSPs). To evaluate whether this effect is elicited by RUN compounds, we performed quantitative real-time PCR (RT-qPCR) analysis in HEK293 cells treated with selected RUN compounds (Fig. 2A).^28,53–55^ Among the genes analyzed, RUN-94 significantly upregulated *HSPA1A*, which encodes heat shock protein 70 (HSP70) compared to the vehicle control. Additionally, the expression of *DNAJA1*, a gene encoding an HSP40/DNAJ-family co-chaperone that modulates HSP70 activity, was significantly increased by all tested compounds (Fig. 2A). These results demonstrate that treatment with RUN compounds robustly induces molecular chaperone expression, supporting the activation of the NFE2L1-dependent cytoprotective transcriptional program.

NAD(P)H dehydrogenase (encoded by the *NQO1* gene) is a cytoprotective enzyme that plays a vital role in various stress responses, particularly in defending against oxidative and proteotoxic stress. The gene encoding this enzyme is strongly inducible by ARE/EpRE elements, which is why we also included this gene in the RT-qPCR analysis.^28,56–58^ Our results (Fig. 2A) revealed a clear increase in *NQO1* gene expression, suggesting a potential role in antioxidative activity following compound treatment. However, despite this increase in expression, the data did not support a significant functional contribution of NQO1 to antioxidant defence under the experimental conditions used.

In addition to regulating proteasome production, the NFE2L1 pathway also governs the expression of the autophagy receptor sequestosome 1, also known as p62 (SQSTM1/p62).^24,29,30,59^ Therefore, we examined whether our compounds increased *SQSTM1* at the gene level. Compared with the vehicle control (DMSO), all selected RUN compounds significantly increased *SQSTM1* expression (Fig. 2A). Sequestosome 1 plays a crucial role in sequestering ubiquitinated proteins into inclusion bodies and facilitating their degradation *via* autophagy. During this process, ubiquitinated protein aggregates are recruited into inclusion bodies through the PB1 domain of p62/SQSTM1. It then directly interacts with microtubule-associated protein 1A/1B-light chain 3 (LC3) *via* its LC3-interacting region (LIR), guiding the inclusions toward degradation through the autophagy–lysosome pathway.^59–63^ Our findings, along with those of previous studies, suggest that treatment with RUN compounds accelerates protein degradation, leading to a faster degradation rate.

To confirm NFE2L1-controlled gene expression at the protein level, western blot analysis was performed. HEK293 cells were treated with selected RUN compounds for 16 hours, after which the protein levels were analyzed. In all experiments, chosen RUN compounds increased expression levels of NFE2L1 transcription factor compared to the vehicle control (DMSO) (Fig. 2B). Similar observations were made with SQSTM1 and LC3. As mentioned earlier, SQSTM1 directly binds to the LC3 region to facilitate the delivery of proteins for autophagic clearance.^62,64,65^ Within cells, LC3 undergoes cleavage to form its cytosolic version, LC3-I, which is further converted into its membrane-bound form, LC3-II, which is anchored in autophagosomal membranes.^60,66^ Consequently, both SQSTM1 and LC3 are widely regarded as markers of autophagy.^65^ These results prove that RUN-89 and RUN-94 significantly elevate total LC3 protein levels, whereas all three compounds contribute to increased levels of the processed form of LC3-II (Fig. 2B). The increase in the LC3-II form indicates active autophagic processes. This, alongside elevated SQSTM1 levels, suggests that an increase in autophagosome formation leads to a higher degradation rate. Additionally, certain RUN compounds markedly increased HSPA1A/HSP70-1 chaperone levels, with RUN-94 notably boosting the cytoprotective protein NQO1 (Fig. 2B). These results align with our previous study,^43^ supporting the hypothesis that upstream stabilization leads to NFE2L1 transcriptional activation.

#### RUN compounds modulate proteasome activity, autophagy, and cell survival

**Fig. 3:**
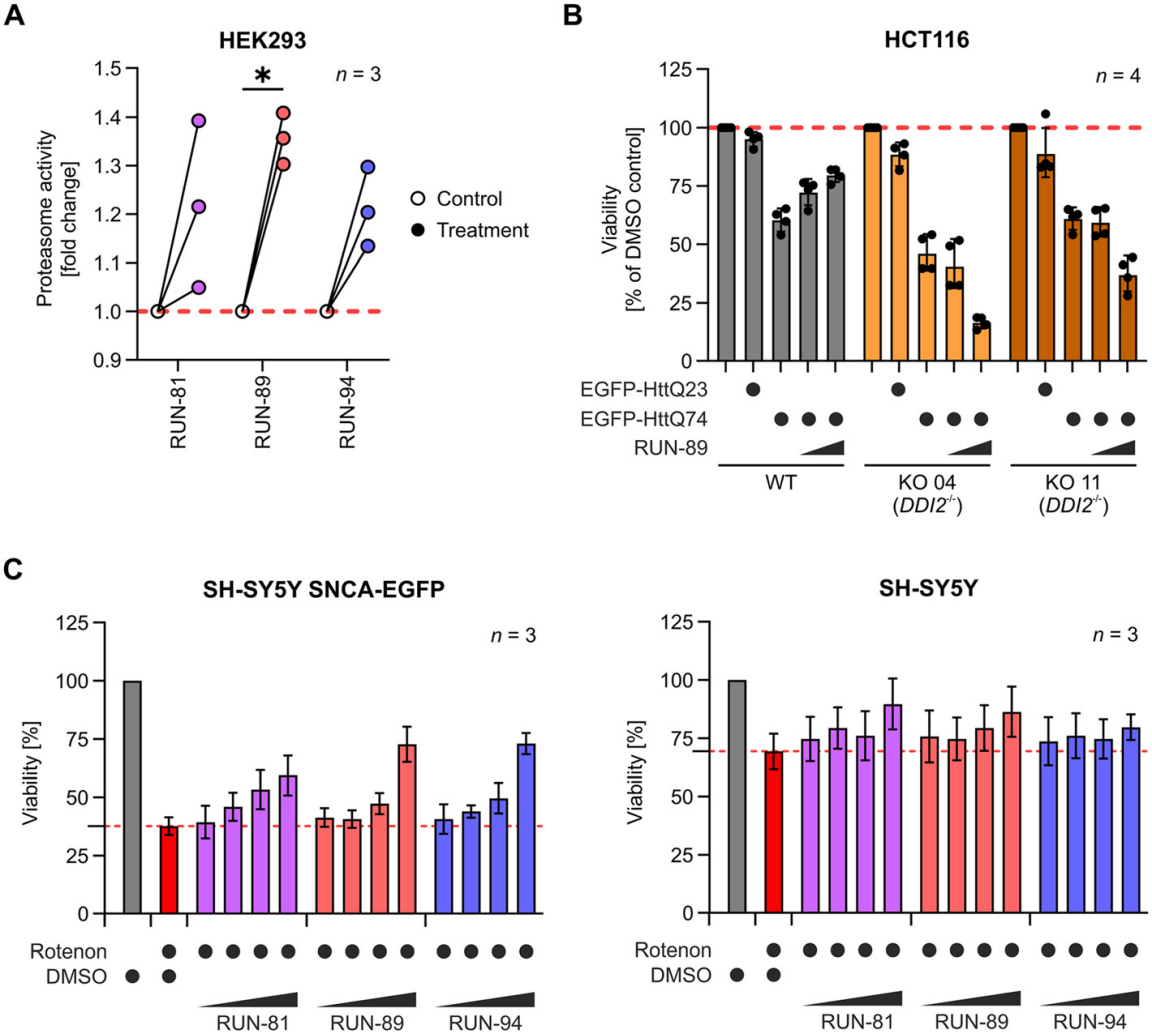
(A) HEK293 cells were treated with RUN compounds at a concentration of 7.5 μM for 16 hours. DMSO was used as a vehicle control. Proteasome chymotrypsin-like activity was measured in cell lysates at 37 °C. Data came from three biological replicates (*n* = 3). Statistical analysis was done using ratio paired *t*-tests corrected for multiple comparisons with Holm–Šídák's method (*α* = 0.05). *p < 0.05. Detailed results of the analysis are shown in SI Table S7. (B) RUN-89 protects against polyQ-mediated toxicity in an NFE2L1-dependent manner. HCT116 parental cells (WT) and two independent DDI2 knockout clones (KO 04 and KO 11), in which NFE2L1 activation is impaired, were transiently transfected with plasmids encoding non-aggregating EGFP-HttQ23 or aggregation-prone EGFP-HttQ74. Cells were treated with 1 or 3 μM RUN-89 for 48 hours, and viability was measured using the alamarBlue assay. RUN-89 increased the survival of WT cells expressing HttQ74 in a dose-dependent manner, whereas its protective effect was markedly reduced in DDI2 knockout cells, demonstrating that the rescue of polyQ-induced cytotoxicity by RUN-89 requires a functional NFE2L1 pathway. Data are shown as the Geomean ± GeoSD from four independent biological replicates (*n* = 4). Statistical analysis was performed on log_2_-transformed values using two-way repeated-measures ANOVA followed by Šídak's multiple comparison test. The results of the analysis are shown in SI Table S8. (C right) Parental SH-SY5Y cells (without α-synuclein expression) were treated in the same manner. (C left) The protective effect of RUN compounds was assessed by rotenone-induced α-synuclein proteotoxicity in SH-SY5Y cells, which mimics the PD-like phenotype. Cells were pre-treated with increasing concentrations of RUN compounds (0.312, 0.625, 1.25, and 2.5 μM) for 4 hours and then rotenone (1.125 μM) treatment was done for 24 hours. All compounds restored cell viability after proteotoxic stress induced by rotenone.

Building on previous findings, we investigated how selected RUN compounds influence proteasomal function itself. Specifically, we examined the chymotrypsin-like activity of the 26S proteasome following a 16 hour treatment with 7.5 μM RUN compounds in the HEK293 cell line. The most pronounced effect was observed with RUN-89 treatment, which significantly increased chymotrypsin-like activity compared to vehicle controls (Fig. 3A). In addition, two other cell lines, MCF7 and SH-SY5Y, were measured (see the SI, Fig. S6). In MCF-7 cells, RUN-89 was the compound that induced the greatest increase in chymotrypsin-like activity. In SH-SY5Y cells (from ATCC CRL-2266 (RRID:CVCL_0019)), however, biological variability led to inconsistent results, resulting in a higher median for RUN-89 without reaching statistical significance.

To further evaluate the protective capacity of RUN-89 in a polyQ aggregation model, we transiently transfected HCT116 parental cells (wt) and DDI2 knockout clones (KO4 and KO11) with plasmids encoding either a non-aggregating EGFP-HttQ23 or an aggregation-prone EGFP-HttQ74 construct. This model is widely used to study Huntington's disease (HD), a prototypical polyglutamine expansion disorder caused by expanded CAG repeats in the huntingtin gene.^67–69^ Previous studies^70^ have shown that cellular rescue from polyQ-induced proteotoxicity critically depends on a functional NRF1 pathway. Cells were treated with increasing concentrations of RUN-89 (1 and 3 μM) for 48 h, and viability was assessed using the alamarBlue assay (Fig. 3B). In wild-type cells expressing HttQ74, RUN-89 significantly improved survival in a dose-dependent manner, whereas this protective effect was strongly reduced in DDI2-deficient clones, consistent with the role of DDI2 as an essential activator of NFE2L1. Together, these results confirm that the beneficial activity of RUN-89 in the polyQ model is mediated through NFE2L1-dependent mechanisms.

A second stress assay was performed using rotenone, a mitochondrial complex I inhibitor. This pesticide is known to induce a Parkinson's disease-like phenotype in the presence of α-synuclein, leading to the formation of intracellular protein inclusions resembling Lewy bodies, as demonstrated in both *in vitro* and *in vivo* studies.^71,72^ To determine whether the RUN compounds have a protective effect under rotenone-induced stress conditions, we used the SH-SY5Y cell line. We used a parental cell line and then a cell line stably expressing α-synuclein with a GFP tag on its N-terminal end. Both cell lines were treated with increasing concentrations of RUN compounds (Fig. 3C). After 4 hours, rotenone (1.125 μM) was added and one day later the viability of the cells was measured. In the absence of compounds, SH-SY5Y cell viability decreased to approximately 30% following rotenone treatment. However, all RUN compounds demonstrated a protective effect against rotenone-induced toxicity in a dose-dependent manner, with RUN-89 showing the strongest response, increasing cell viability up to 70% (Fig. 3C right). Notably, parental SH-SY5Y cells treated with the combination of rotenone and RUN compounds exhibited no reduction in viability, indicating that the protective effect is linked to α-synuclein aggregation related toxicity and that the RUN compounds are non-cytotoxic at the tested concentrations in these cells.

Additionally, we examined the effects of the selected RUN compounds, including our lead candidate RUN-89, using a fluorescent EGFP-SNCA reporter. This reporter is prone to aggregation because of its slow protein turnover. To assess compound efficacy, we measured the fluorescence signal of GFP-tagged synuclein within aggregates. Our results (Fig. S7) indicate that RUN candidates reduce EGFP-SNCA aggregation, as evidenced by a significant decrease in the GFP signal, comparable to the reduction observed with two known proteasome activators, PD169316 and tadalafil.^73^

Furthermore, we also demonstrated that the selected RUN compounds do not induce the release of reactive oxygen species (see the SI, Fig. S8). For this experiment, a DCFH-DA (2′,7′-dichlorofluorescein diacetate) fluorescence assay was employed. *Tert*-butyl hydroperoxide (*t*-BHP) served as the positive control, a commonly used reference compound for studying the cellular mechanisms underlying oxidative stress in tissues and cells.^74–76^

#### RUN-89 prevents aggregate formation in cells

**Fig. 4:**
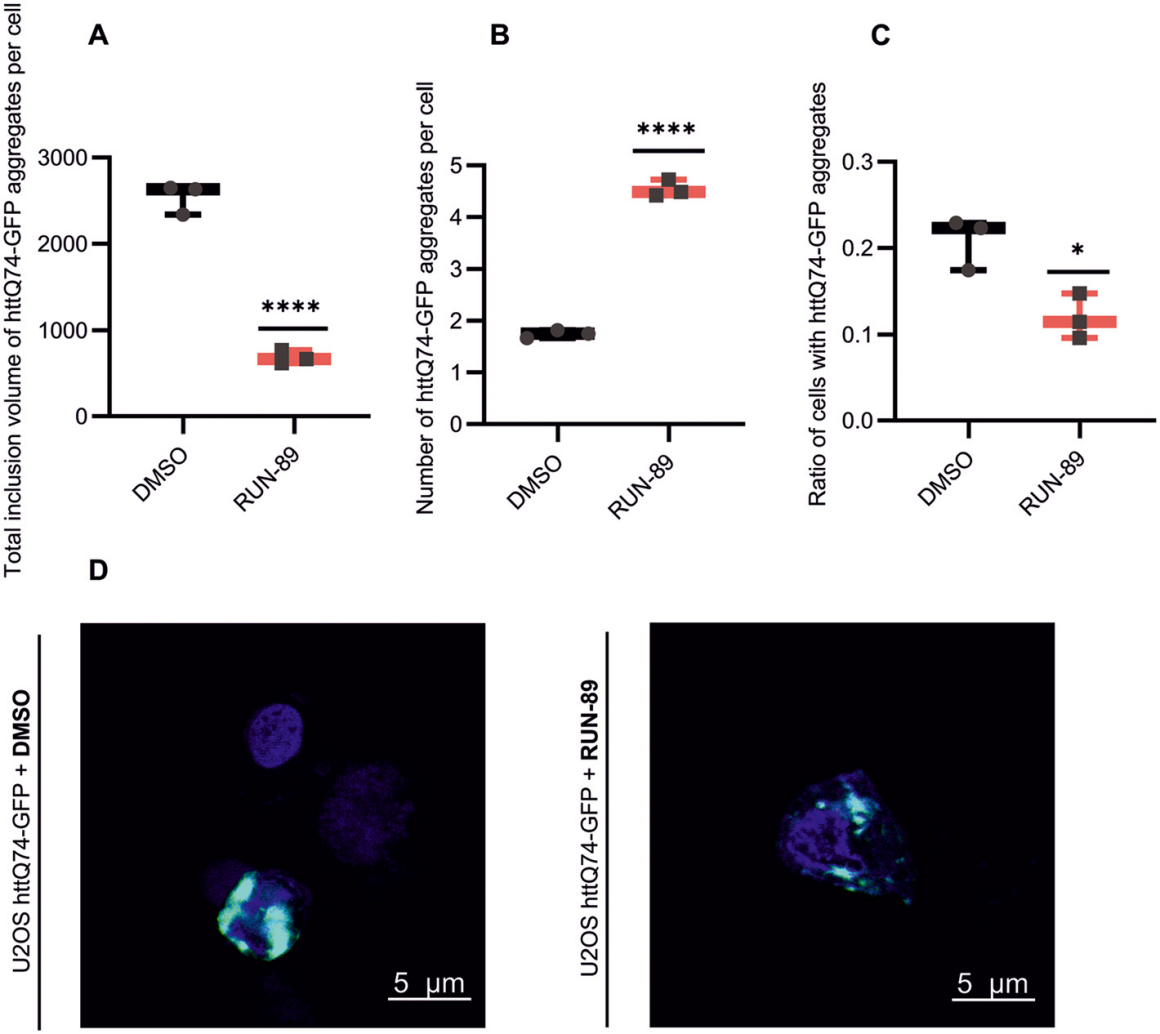
RUN compounds inhibit the formation of huntingtin Q74 aggregates. U2OS cells expressing EGFP-HttQ74 were treated for 24 hours with either the caspase inhibitor BOC-D-FMK, 5 μM RUN compounds, or DMSO (negative control). In total, cells from three independent experiments were analyzed (*n* = 3) and from every well 10 areas were screened. (A) After RUN-89 treatment, there was a reduction in the volume of polyQ aggregates within individual cells, further supporting the idea that the treatment prevents aggregate clustering. The volume value was measured in voxels. (B) A greater number of aggregates appear primarily due to a decrease in the total inclusion volume of aggregates. These findings suggest that treatment prevents the formation of larger protein clusters. (C) Addition of RUN-89 to cells also decreased the ratio of cells allowing the formation of aggregates. (A–C) Data are shown as the mean ± SD. Statistics was done using a two-tailed unpaired *t*-test on the means per well. *p < 0.05, ****p < 0.001. Detailed results of the analysis are shown in SI Table S9. (D) Fluorescence microscopy images represent U2OS cells overexpressing EGFP-HttQ74 after treatment with RUN-89 and DMSO for 24 h. Nuclei stained with Hoechst 34580 (blue) and EGFP-HttQ74 (shown in green). The images were taken at the same time on the same day, with all conditions kept identical at the Imaging Methods Core Facility at BIOCEV.

Considering these earlier findings, we assessed the impact of the most promising compound, RUN-89, on protein aggregate formation in cells. U2OS cells were transfected with the EGFP-HttQ74 plasmid, a model for polyglutamine (polyQ) aggregation, and then treated with the caspase inhibitor BOC-D-FMK in combination with either 5 μM RUN compounds or a vehicle control for 24 hours prior to the microscopy analysis (Fig. 4). The volume of aggregates decreased significantly in cells treated with the RUN-89 compound compared to the vehicle-treated group (Fig. 4A). A decrease in aggregate size leads to an increase in their number, as the formation of large aggregates is prevented. Instead, multiple smaller aggregates are formed, which, according to our assumption, could be more easily degraded (Fig. 4B). Additionally, RUN-89 treatment led to a clear reduction in the number of GFP-positive cells with polyQ aggregates (Fig. 4C). Representative fluorescence microscopy images of U2OS cells expressing EGFP-HttQ74 treated with RUN-89 are shown in Fig. 4D, highlighting the difference between RUN-89-treated and vehicle-treated cells.

#### RUN-89 activates the SKN-1A/NFE2L1 pathway in *C. elegans*, prevents protein aggregation, and enhances stress resilience *in vivo*

Given the *in vitro* findings that RUN-89 is very promising for preventing protein aggregate formation in cells, we also explored the *in vivo* effects of this treatment. The nematode *Caenorhabditis elegans* was chosen as an ideal model organism because of its single NFE2L1 orthologue, known as SKN-1A. SKN-1A controls the UPS in response to stress signals and may also play a role in autophagic protein clearance.^22,77,78^ SKN-1A was originally associated only with oxidative stress defense, but subsequent research has revealed its broader involvement in detoxification, immunity, proteostasis, and metabolism, playing a role in maintaining homeostasis across these vital processes. This information highlights that SKN-1A plays a crucial role in interventions that increase both lifespan and health span.^79^

Firstly, we investigated whether RUN-89 triggers SKN-1A activation in *C. elegans* by using the GR2183 strain. By using this strain, the activation of SKN-1A is indicated by GFP expression, which is driven by the promoter of the proteasome subunit gene (rpt-3p::GFP).^80,81^ After exposing young adults to 25 μM RUN-89 or a DMSO control for 24 hours at 20 °C, we observed a significantly higher average relative fluorescence intensity per GR2183 animal in those treated with RUN-89 compared to those treated with the vehicle (Fig. S9).

**Fig. 5:**
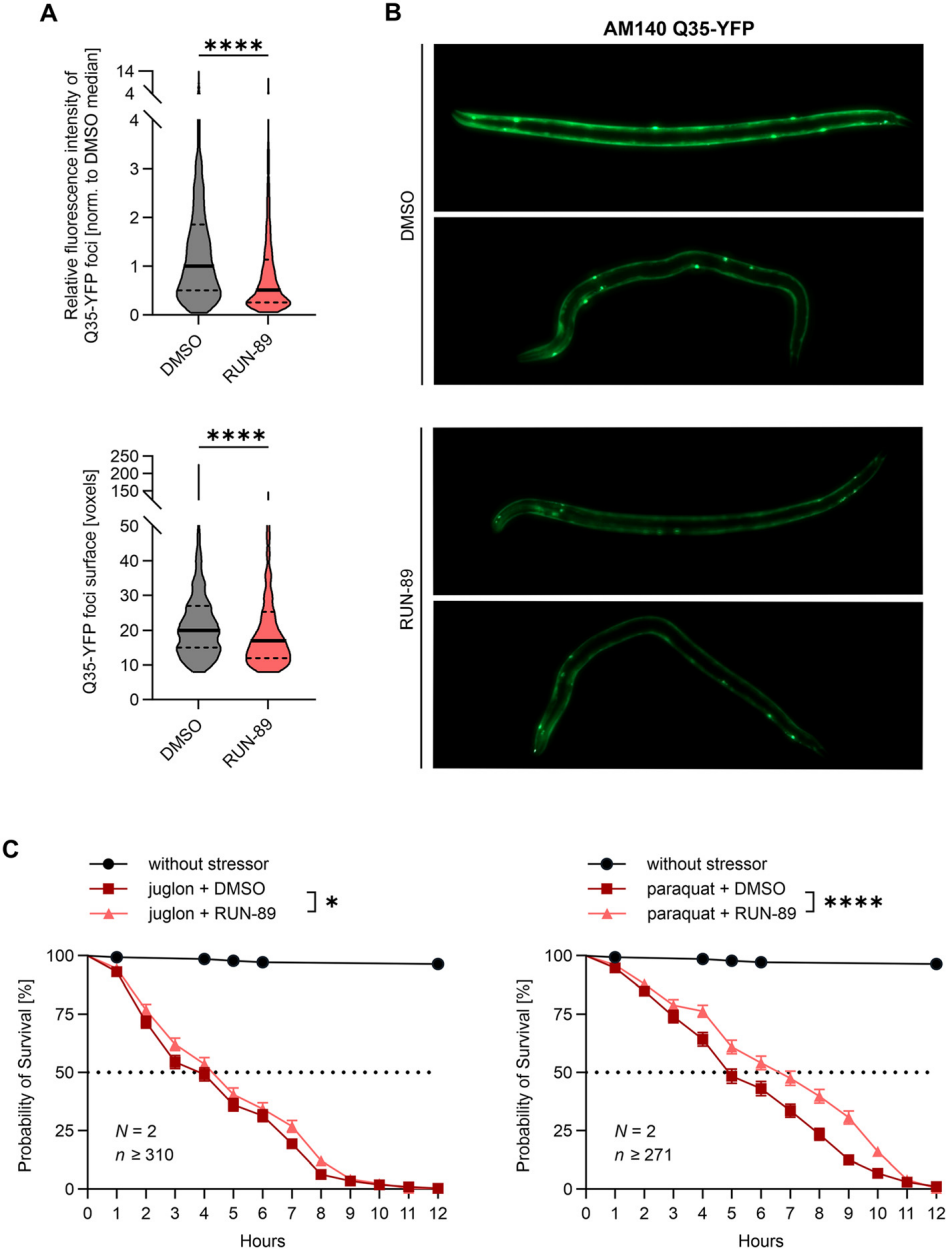
RUN-89 prevents protein aggregation and increases stress resistance in *C. elegans*. (A) The AM140 strain expressing aggregation-prone Q35-YFP was treated with 50 μM RUN-89 or the vehicle for 6 days, followed by microscopy analysis. Overall, 959 Q35-YFP foci (DMSO = 517; RUN-89 = 442) were identified in 102 animals (DMSO: *N* = 3 populations, *n* = 55 animals in total; RUN-89: *N* = 3 populations, *n* = 47 animals in total). The two dashed horizontal lines indicate the interquartile range, and the thick horizontal line represents the median. The Kolmogorov–Smirnov test verified a significant reduction of surface and integrated optical density of foci after RUN-89 treatment. (B) Representative images of two animals per treatment condition. Images were taken on the same day, using identical settings for the instruments used. (C) Protective effect of RUN-89 against juglone and paraquat-induced stress. A synchronized population of N2 wild-type worms was exposed to 50 μM RUN-89 for 24 hours to protect against acute oxidative stress triggered by 180 μM juglone and 200 mM paraquat. The mortality was monitored hourly over a 12 hour period. For paraquat treatment, 548 animals were examined (DMSO: *N* = 2 populations of 135 and 136 animals, *n* = 271 animals in total; RUN-89: *N* = 2 populations of 146 and 131 animals, *n* = 277 animals in total). For juglone treatment, 629 animals were examined (DMSO: *N* = 2 populations of 167 and 152 animals, *n* = 319 animals in total; RUN-89: *N* = 2 populations of 141 and 169 animals, *n* = 310 animals in total). Results were analyzed using the Kaplan–Meier method and are presented as survival percentages with standard errors. The survival curves were compared using the Gehan–Breslow–Wilcoxon test (juglone) and log-rank test (paraquat). (A and C) Detailed results of the analysis are shown in SI Tables S10 and S11. *p < 0.05, ****p < 0.001.

Furthermore, we explored the role of RUN-89 in preventing huntingtin protein aggregation in *C. elegans*. For this AM140 strain, stably expressing aggregation-prone 35-glutamine long repeats marked with yellow fluorescence protein (Q35-YFP) were used.^82,83^ A six-day treatment with 50 μM RUN-89 or a vehicle control was conducted, followed by microscopy analysis. The results shown in Fig. 5A and B revealed a significant decrease in both the foci surface area and relative fluorescence density in animals treated with RUN-89 compared to those treated with the DMSO vehicle. These findings highlight the substantial impact of RUN-89 on foci formation or persistence, further supporting its role in suppressing pathological clustering in protein aggregation diseases or reducing stress-response assemblies.

Next, we examined whether the compound RUN-89 protects animals from oxidative damage/death induced by increased reactive oxygen species (ROS) levels. In worms, the herbicide paraquat and the naturally occurring quinone compound juglone are well-known for inducing oxidative stress.^84^ In this experiment, the wild-type (N2) strain was used, and RUN-89 was administered at a concentration of 50 μM for 24 hours. Following this pretreatment, approximately 130 animals were transferred to plates containing either 180 μM juglone or 200 μM paraquat. Worm mortality was monitored over a 12-hour period. The results (Fig. 5C) demonstrated a significant protective effect of RUN-89 against lethal oxidative stress induced by juglone and paraquat.

## Conclusions

Based on the structure of RUN-47 described previously,^43^ we designed and synthesized a targeted library of 45 compounds with the goal of identifying novel NFE2L1 pathway activators. The library features structural diversity across three distinct scaffolds. Scaffold A contained a 4-piperidinone core, scaffold B explored the structural motif of the nortropinone core, and scaffold C featured the original core from RUN-47 4-aminocyclohexanone. These low-molecular-weight compounds serve as potent NFE2L1 activators and valuable tool compounds for studying the influence of various scaffolds and moieties in the vicinity of the nitrogen atom on NFE2L1 pathway activation. Scaffold C emerged as the most successful among the three tested architectures, and three compounds (RUN-81, RUN-89 and RUN-94) were selected for further investigation. The most promising compound, RUN-89, showed robust activation of the NFE2L1 pathway, leading to increased expression of proteasome subunit mRNAs and upregulation of several stress response transcripts. These included *HSPA1A* (encoding the major heat shock protein Hsp70), *DNAJA1* (encoding an HSP40/DNAJ-family co-chaperone that regulates Hsp70 activity), *NQO1* (a cytoprotective enzyme), and *SQSTM1* (p62, an autophagy receptor). In functional assays, RUN-89 enhanced proteasome activity, prevented protein aggregation in two cellular models, and reduced the size, accumulation, and formation of protein aggregates. Importantly, these effects were confirmed to be NFE2L1-dependent, as demonstrated by mechanistic validation in *DDI2* knockout cells and by siRNA-mediated knockdown of *NFE2L1* and *NFE2L2*. Additional validation using polyQ aggregation assays further supported the NFE2L1-specific mechanism of action. Notably, these effects were achieved without inducing cellular stress or interfering with protein degradation *via* the ubiquitin–proteasome system. Consistent with this, antioxidant treatment with NAC had no effect on RUN-induced NFE2L1 reporter activation, excluding indirect NFE2L2 involvement through oxidative stress.

In an *in vivo* model, RUN-89 prevented the accumulation of mutant huntingtin protein in the nematode *C. elegans*, an effect likely mediated by activation of the SKN-1A/NFE2L1 pathway. While RUN-89 displayed improved functional outcomes compared to the parent compound RUN-47, both compounds exhibited comparable levels of NFE2L1 pathway activation.

Overall, this study provides valuable insight into the impact of scaffold modifications and nitrogen-region substitutions, ultimately reinforcing RUN-47 as a validated lead compound. These findings highlight the potential of this chemical series as a promising foundation for the development of NFE2L1-targeted therapeutics.

## Declaration of generative AI and AI-assisted technologies in the writing process

The authors utilized ChatGPT solely to enhance the language and readability of this work. Following its use, they carefully reviewed and edited the content as needed, assuming full responsibility for the final publication.

## Author contributions

Conceptualization: A. M. and K. G. S.; data curation: L. S., Z. S., J. S. and M. A.; formal analysis and validation: L. S., Z. S., J. S., M. A., A. M. and K. G. S.; investigation: L. S., Z. S., J. S. and M. A.; methodology: L. S., Z. S., M. A., J. S., A. M. and K. G. S.; resources, P. M., A. M. and K. G. S.; supervision, K. G. S. and A. M.; visualization: L. S., Z. S., J. S., A. M. and K. G. S; writing – original draft: L. S. and Z. S.; writing – review & editing: L. S., Z. S., J. S., A. M. and K. G. S.; funding acquisition, P. M., A. M. and K. G. S.

## Conflicts of interest

J. S., Z. S., M. A., L. S., P. M., A. M., and K. G. S. have submitted multiple patent applications related to this work. All authors declare that they have no known competing financial interests that could have appeared to influence the work reported in this paper.

## Supplementary Material

MD-016-D5MD00584A-s001

MD-016-D5MD00584A-s002

## Data Availability

The data supporting this article have been included as part of the supplementary information (SI). Supplementary information is available. See DOI: https://doi.org/10.1039/d5md00584a.
