# Supplementary material for: Investigating NFE2L1 activators for targeted protein aggregate clearance: a follow-up study
Source: RSC Med Chem. 2025 Oct 22;16(12):6397–411. doi: 10.1039/d5md00584a (PMC12606464; doi:10.1039/d5md00584a)
Supplement: MD-016-D5MD00584A-s001 [file MD-016-D5MD00584A-s001.pdf]

Supplementary Information (SI) for RSC Medicinal Chemistry.
This journal is © The Royal Society of Chemistry 2025

SUPPORTING INFORMATION

Investigating NFE2L1 Activators for Targeted Protein Aggregate Clearance: A Follow-Up Study

Zuzana Smahelova,[a,b†] Lucie Svobodova,[a,c†] Jindrich Sedlacek,[a,b] Michael Adamek,[a,b] Marketa Pimkova Polidarova, [a,b] Pavel Majer,[a] Ales Machara[a*] and Klara Grantz Saskova[a,b*]

1. Institute of Organic Chemistry and Biochemistry of the Czech Academy of Sciences, Flemingovo n. 2, 16610 Prague, Czech Republic
2. Department of Genetics and Microbiology, Charles University and Research Center BIOCEV, Prumyslova 595, 25250 Vestec, Czech Republic
3. Department of Organic Chemistry, Charles University, Hlavova 2030/8, Prague 2, 12843, Czech Republic

* Corresponding authors: machara@uochb.cas.cz (A. M.); saskova2@natur.cuni.cz (K.G.S.)
† These authors contributed equally to this work

Contents

1 Supplementary Results ... 3
2 Biology Experimental Procedures ... 14
2.1 Constructs ... 14
2.2 Cell cultures ... 14
2.3 Dual luciferase phenotypic assays ... 14
2.4 Monitoring protein degradation using Ub^{G76V}-GFP reporter cells ... 15
2.5 Quantitative RT-PCR ... 15
2.6 Immunoblot analysis ... 16
2.7 Proteasome activity assay ... 17
2.8 XBP1-endoplasmic reticulum (ER) stress reporter assay ... 17
2.9 PolyQ toxicity assay ... 17
2.10 Oxidative stress detection ... 18
2.11 SNCA-EGFP degradation ... 18
2.12 Analysis of polyQ aggregation in cells ... 18
2.13 *C. elegans* strains and maintenance ... 19
2.14 Microscopy of *C. elegans* ... 19
2.15 Acute juglone/paraquat-induced oxidative stress resistance in *C. elegans* ... 19
2.16 Analysis of proteasome genes expression in NFE2L1/NFE2L2-knockdown cells 20
3 Chemistry Experimental Procedures ... 21
3.1 Experimental details for prepared compounds ... 21
3.2 List of compounds ... 21
3.3 ^{1}H & ^{13}C NMR Spectra of All New Compounds ... 45
4 References ... 91

1 Supplementary Results

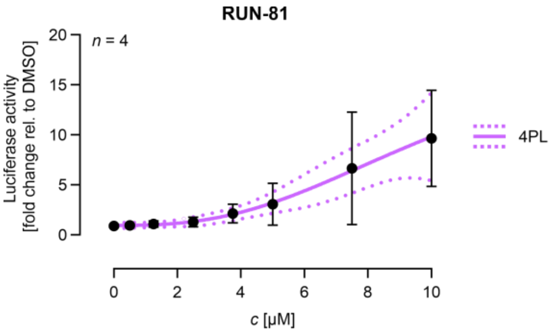

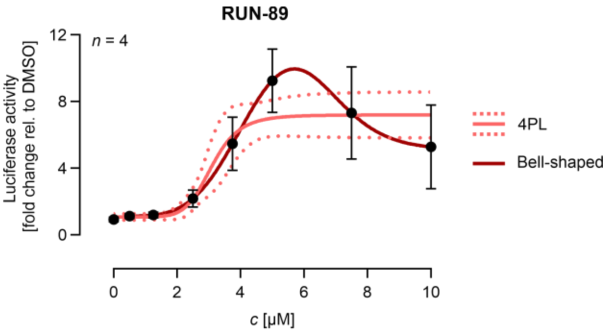

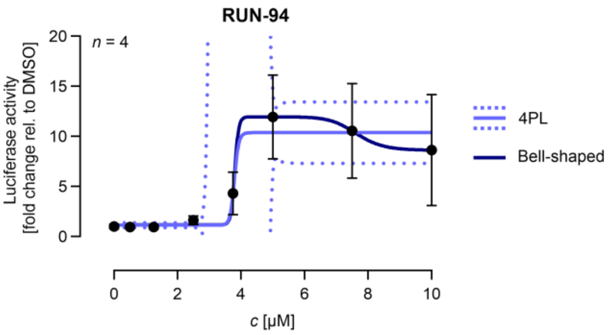

	RUN-81	RUN-89	RUN-94
**[Agonist] vs. Response - Variable slope (four parameters)**			
**Best-fit values**			
Bottom	0,95	1,08	1,13
Hillslope	2,70	7,14	55,77
Top	19,66	7,20	10,37
EC50	10,40	3,10	3,79
logEC50	1,02	0,49	0,58
Span	18,71	6,12	9,24
**95% CI**			
Bottom	0,7274 to 1,415	0,9316 to 1,347	1,009 to 1,663
Hillslope	1,605 to 7,790	3,950 to +infinity	??? to +infinity
Top	??? to +infinity	6,630 to 10,99	9,295 to ???
EC50	4,685 to +infinity	-infinity to 3,789	???
logEC50	0,6707 to +infinity	-infinity to 0,5785	???
**Goodness of Fit**			
R^2	0,60	0,83	0,71
**Bell-shaped, X is concentration**			
**Best-fit values**			
Plateau1		1,124	-2,174
Dip		19,12	8,602
Plateau2		5,392	11,95
EC50_1		4,889	3,803
nH1		-4,123	Unstable
EC50_2		6,813	7,634
nH2		-7,321	18,05
**95% CI**			
Plateau1		??? to 1,483	??? to +infinity
Dip		???	???
Plateau2		4,434 to +infinity	-infinity to +infinity
EC50_1		2,954 to +infinity	-7276426435157 to +infinity
nH1		-4,212 to ???	(Very wide)
EC50_2		-12889 to +infinity	-infinity to +infinity
nH2		-15,59 to ???	-infinity to +infinity
**Goodness of Fit**			
R^2		0,86	0,72

Figure S1: Concentration–response of luciferase cell-based assay (PSMA4-ARE-luc2P reporter) on treatment with **RUN-81**, **RUN-89**, and **RUN-94**. Concentration is plotted on the X-axis. Dose–response relationships were analyzed using a four-parameter logistic curve (4PL), or a bell-shaped dose-response curve, both with weighting 1/Y^2. Dotted lines represent 95% confidence bands. Calculated EC_{50} values, curve parameters, and asymmetrical confidence intervals are summarized in the accompanying table. Data are presented as mean ± SD from four independent experiments (*n*=4).

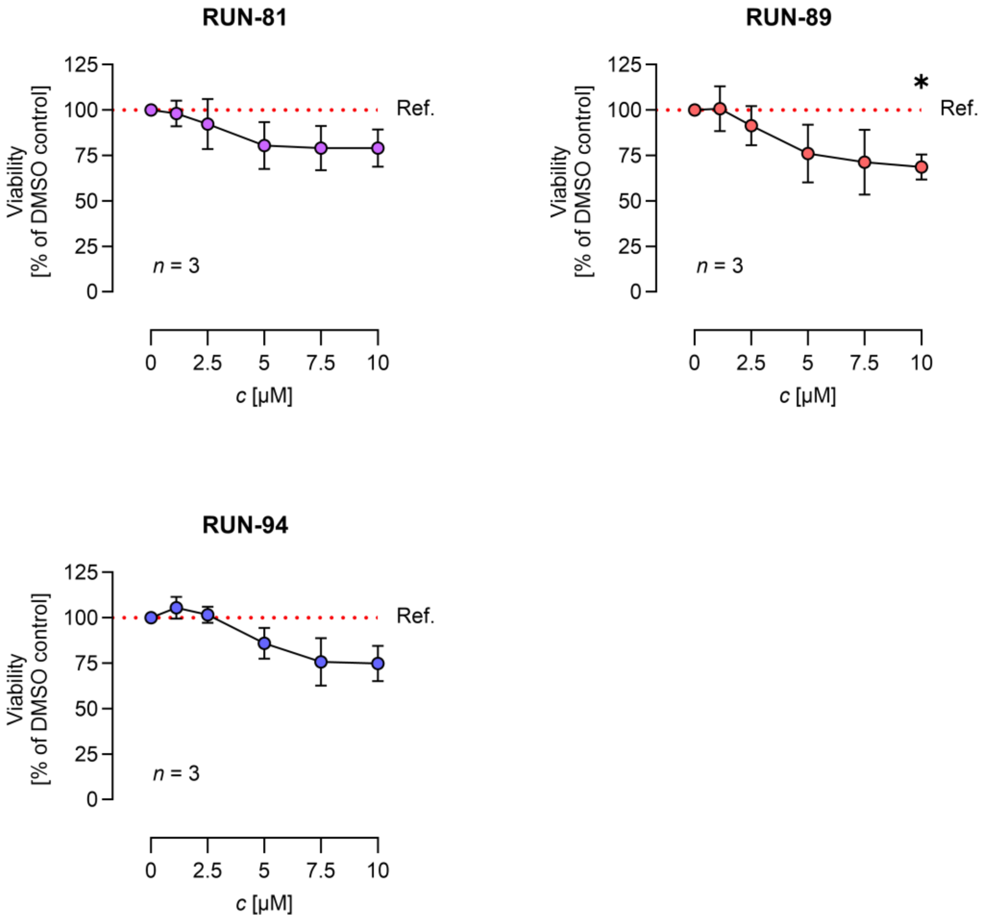

Figure S2: Concentration-dependent cytotoxicity assessment of RUN compounds.

HEK293 cells were treated for 16 hours with increasing concentrations of RUN compounds (1.25 µM; 2.5 µM; 5; 7.5 and 10 µM) or DMSO (negative control). Cell viability was determined using the alamarBlue assay following the protocol of Riss.(1) Data are presented as means ± SD from three independent experiments (*n* = 3). Statistical analysis was done using repeated measures one-way ANOVA with Geisser–Greenhouse correction, followed by Dunnett's multiple comparisons test. *$p<0.05$. Detailed results of the analysis are shown in **Supplementary Table S13.**

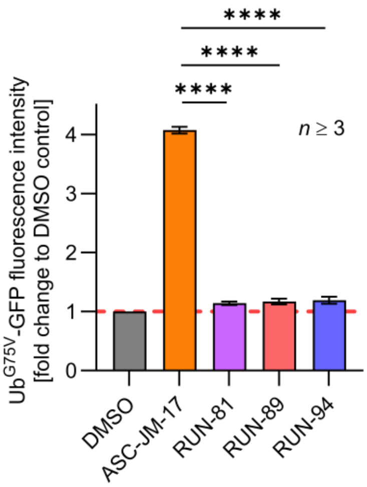

Figure S3: RUN compounds do not interfere with the ubiquitin-proteasome system.

U2OS cells stably expressing the Ub-G76V-GFP reporter were treated with the most effective RUN compound (5 µM) for 8 hours. Fluorescence intensity was normalized to cell viability (measured by the alamarBlue assay) and compared to vehicle control (DMSO). Unlike the clinically validated ASC-JM-17, which impaired proteasomal degradation, the RUN compounds showed no significant disruption of ubiquitin-system function. Data are presented as means ± SD from three independent experiments ($n = 3$). Statistical analysis was performed using a multiple unpaired t-test with Welch's correction, with adjustment for multiple comparisons by the Holm–Šídák method ($\alpha = 0.05$). ****$p<0.0001$. Detailed results of the analysis are shown in **Supplementary Table S14**.

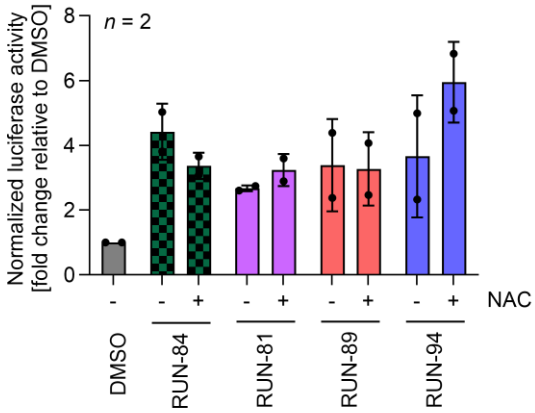

Figure S4: Effect of the antioxidant N-acetyl-L-cysteine (NAC) on RUN compound-induced NFE2L1 activation.

To determine whether RUN compounds activate the NFE2L1 pathway via reactive oxygen species (ROS) generation or through Keap1-NRF2 pathway crosstalk, we used the 3xPSMA4-ARE-LUC dual reporter assay in HEK293 cells. Cells were treated with RUN compounds (5 μM) for 16 hours, both in the presence and absence of NAC. A reduction in luminescence signal upon NAC co-treatment would indicate that the compound activates the reporter via ROS-mediated mechanism or Keap1-NRF2-dependent oxidative/electrophilic stress. For example, **RUN-84** was excluded due to its reduction in activation following the addition of NAC, suggesting ROS involvement. In contrast, **RUN-94** retained its activity and showed a possible synergistic effect with NAC. Bars with errors represent mean ± SD.

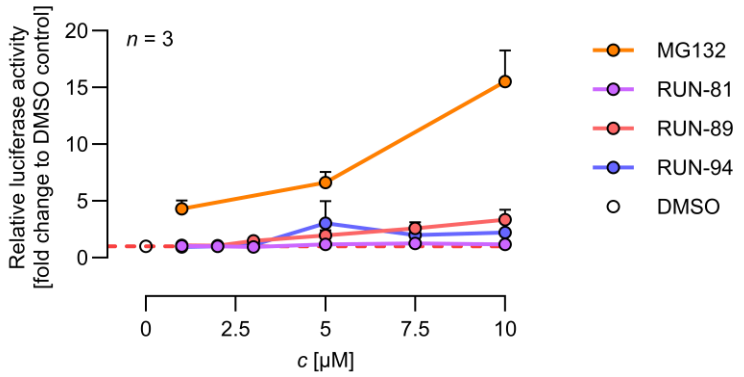

Figure S5: Impact of RUN compounds on ER stress-induced activation of the XBP1 reporter.

HEK293 cells were transfected with the CMV-XBP-GLuc reporter plasmid and allowed to stabilize overnight. Following stabilization, the cells were treated with MG132 or various concentrations of the test compounds (1, 2, 3, 5, 7.5, and 10 µM) for 16 hours. The Gaussia luciferase signal, which reflects XBP1 mRNA splicing, was adjusted based on cell viability as measured by the alamarBlue assay. Data are represented as Geomean ± GeoSD of three independent experiments ($n = 3$. Statistical comparison of MG132 and RUN compounds vs DMSO and RUN compounds vs MG132 was performed on $\log_2$-transformed values using two-way repeated-measures ANOVA with Geisser–Greenhouse correction, followed by Dunnett's multiple comparisons test with individual variances computed for each comparison. Results of the analysis are shown in **Supplementary Table S15**.

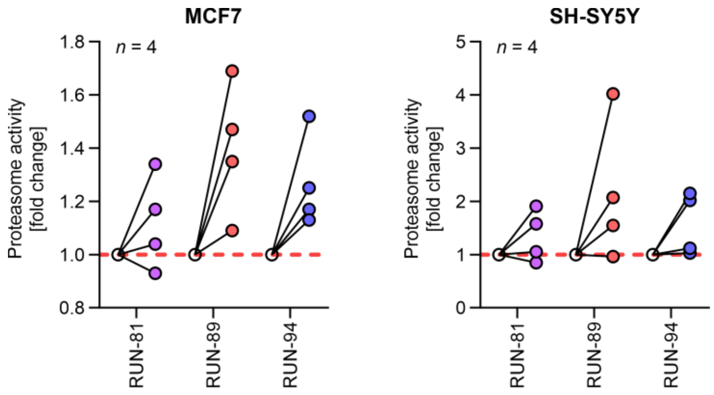

Figure S6: RUN compounds enhance chymotrypsin-like activity of the 26S proteasome in MCF7 and SH-SY5Y cells.

Cells were treated with 5 µM RUN compounds for 16 hours, with DMSO (0.5%) as the vehicle control. Proteasome chymotrypsin-like activity was measured in cell lysates at 37 °C. Four independent experiments were conducted. Statistical analysis was performed using ratio paired t-tests corrected for multiple comparisons with the Holm-Šídák's method ($\alpha = 0.05$). Detailed results of the analysis are shown in **Supplementary Table S16**.

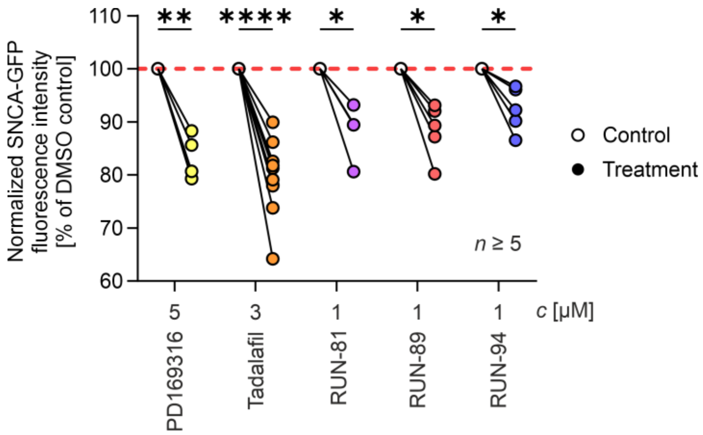

Figure S7: RUN compounds promote degradation of the aggregation-prone SNCA-EGFP reporter.

SH-SY5Y cells stably expressing SNCA-EGFP were treated for 16 hours with DMSO (vehicle control), proteasome activators PD169316 (3 µM) and tadalafil (5 µM), or compounds (1 µM). The GFP fluorescence signal was then normalized to that of the DMSO control and the cell number. The experiment was performed in at least five biological replicates. Statistical analysis was performed using paired t-tests with Holm–Šídák correction for multiple comparisons (α = 0.05). *p<0.05, **p<0.01,****p<0.0001. Detailed results of the analysis are shown in **Supplementary Table S17**.

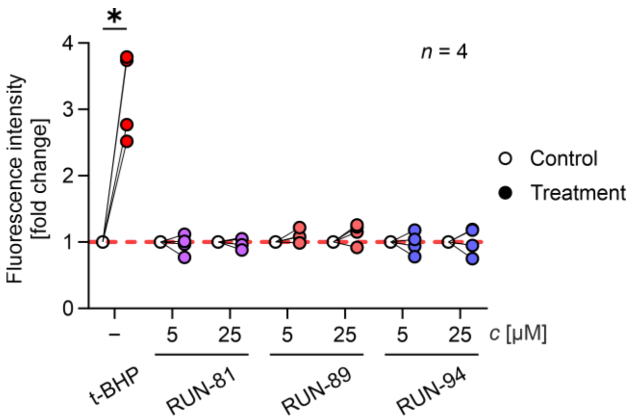

Figure S8. RUN compounds do not induce reactive oxygen species (ROS) production in the DCFH-DA (2',7'-dichlorofluorescein diacetate) fluorescence-based assay.

The DCF fluorescence intensity was measured after a 2-hour treatment with RUN compounds at concentrations of either 5 µM or 25 µM. DMSO served as the negative control, while 25 µM tert-butyl hydroperoxide (TBHP) was used as the positive control. Fluorescence values were normalized to DMSO control. Statistical comparison was performed using multiple ratio paired t-test with Holm–Šídák correction for multiple comparisons ($\alpha = 0.05$). *$p<0.05$. Detailed results of the analysis are shown in **Supplementary Table S18**.

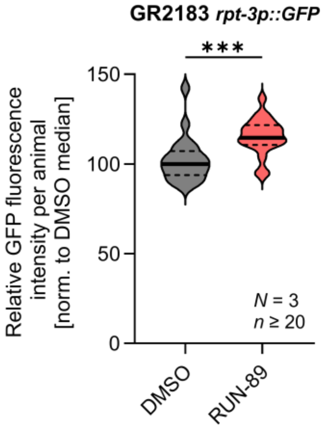

Figure S9. RUN-89 activates the *NFE2L1* orthologue gene *SKN-1A* in *C. elegans*.

In the GR2183 strain, activation of the SKN-1A is reported by the expression of GFP under the control of the proteasome subunit gene promoter (*rpt-3p::GFP*). Animals were treated with 25 µM **RUN-89** or DMSO (vehicle control) for 24 hours with subsequent microscopic analysis. Results of relative GFP fluorescence intensity per single animal are presented. Overall, 71 animals (*n* | DMSO = 20, RUN-89 = 21) from three independent experiments (*N)* were analyzed. The two dashed horizontal lines indicate the interquartile range, and the thick horizontal line represents the median. A significant increase in fluorescence intensity after **RUN-89** treatment was verified by a Kolmogorov-Smirnov test. ***$p<0.001$. Detailed results of the analysis are shown in **Supplementary Table S19**.

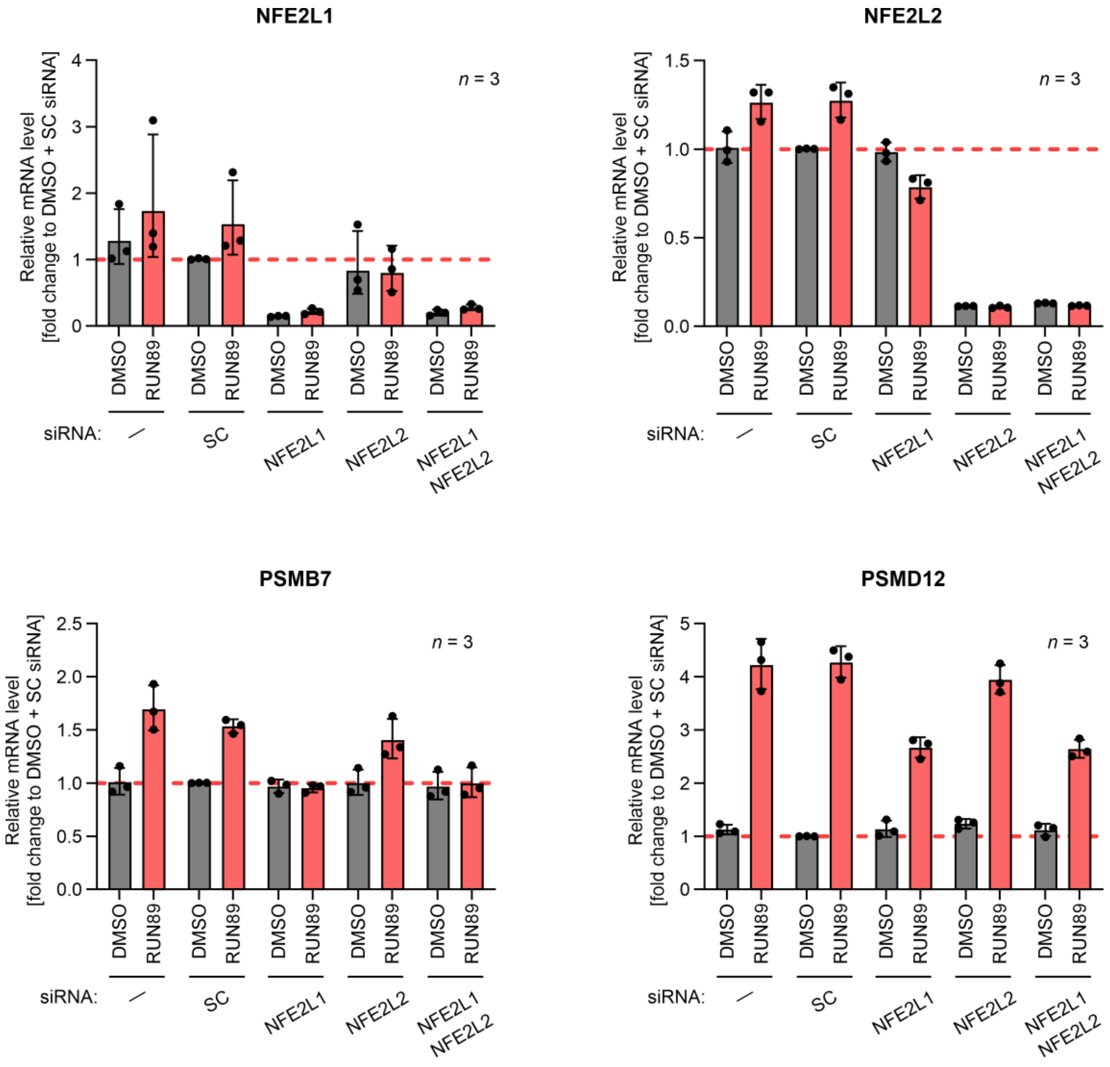

Figure S10: Effects of **RUN-89** on NFE2L1- and NFE2L2-associated target genes following siRNA silencing.

HEK293 cells were transfected with lipid nanoparticles (LNPs) containing siRNAs against *NFE2L1*, *NFE2L2*, or a non-targeting control siRNA. After 24 hours, cells were treated with 7.5 µM **RUN-89** or DMSO (vehicle) for an additional 16 h, and transcript levels of *NFE2L1*, *NFE2L2*, *PSMB4*, *PSMB7*, and *PSMD12* were quantified by RT-qPCR. Data are presented as mean ± SD from three independent biological replicates (n = 3). Log_2-transformed data were analyzed by two-way repeated-measures ANOVA with Geisser–Greenhouse correction, followed by Tukey's multiple comparison test with individual variances computed for each comparison. The results of the analysis are shown in Supplementary Table **S20**.

2 Biology Experimental Procedures

Safety statement: No unexpected or unusually high safety hazards were encountered.

2.1 Constructs

The 3xPSMA-ARE reporter plasmid used to evaluate NFE2L1-driven transcription has been described previously.(2) The pRL-TK plasmid (#E2241) was obtained from Promega (USA). The Ub G76V GFP (#11941), pEGFP-Q23 (#40261), and pEGFP-Q74 (#40262) plasmids were obtained from Addgene (USA). The pEGFP-N1-α-S plasmid was generously provided by Dr. Mireille M.A.E. Claessens (University of Twente, Netherlands. The pET28a-α-synuclein plasmid for the expression of recombinant protein in *E. coli* was kindly provided by Dr. Anna Filipek (Nencki Institute of Experimental Biology, Poland). The pLV-CMV-XBP-GLuc-bc-Puro plasmid was provided by Dr. Arnaud Zaldumbide (Leiden University, Netherlands).

2.2 Cell cultures

Cell lines were sourced from the ATCC (USA) and/or ECACC (UK), namely HCT116 (human colorectal carcinoma), ATCC CCL-247 (RRID:CVCL_0291), HEK-293 (human embryonic kidney), ATCC CRL-1573 (RRID:CVCL_0045), SH-SY5Y (human neuroblastoma), ATCC CRL-2266 (RRID:CVCL_0019). The characteristics of DDI2 knockout cells derived from HCT116 cells have been described previously (Sedlacek et al., 2025). All cell lines were tested for mycoplasma contamination. Most of the cell lines were cultured in Dulbecco's Modified Eagle Medium (DMEM) enriched with 10% fetal bovine serum (FBS) and 2 mM L-glutamine, under standard conditions of 37 °C with 5% CO_2. DNA transfection was performed with Lipofectamine 2000, following the manufacturer's instructions. Transgenic cells were then selected with either G418 (400 µg/mL) or hygromycin B (450 µg/mL), based on the selection marker encoded by the plasmid used. Cell viability was evaluated using the alamarBlue assay according to the standard protocol.(1)

2.3 Dual luciferase phenotypic assays

HEK293-derived cells stably expressing the 3×PSMA4-ARE-Luc reporter were maintained at low to mid passage numbers before use. For each experiment, cells were transiently transfected with the pRL-TK Renilla luciferase plasmid using Lipofectamine 2000. Approximately 5–6 hours post-transfection, cells were seeded into plate format according to the experiment (384-well plate mostly). On the following day, cells were treated with the indicated test compounds or DMSO vehicle control for 16 hours (unless otherwise specified). After treatment, cells were lysed and luciferase activities were quantified using the Dual-Luciferase® Reporter Assay System according to the manufacturer's instructions and established protocols.(2, 3) For assays using the CMV-XBP-Gluc construct encoding Gaussia luciferase, cells were processed following the protocol described in Tannous et al.(4) The DDI2 knockout clones KO4 and KO11, previously generated from the HCT116 parental cell line, together with the corresponding wild-type HCT116 cells, were also used in this assay. These cells were stably transfected with the 3×PSMA4-ARE-LUC dual reporter construct, containing a firefly luciferase gene under the control of three tandem NFE2L1-responsive antioxidant

response elements (AREs). To control for transfection efficiency, cells were co-transfected with a Renilla luciferase construct (pRL-TK). Cells were seeded into 96-well plates and, after overnight adherence, treated with 5 µM RUN-89, 0.5 µM bortezomib (positive control), or 0.1% DMSO (vehicle control) for 16 hours. Following treatment, luciferase activity was measured using the Dual-Luciferase Reporter Assay System according to the manufacturer's instructions, and firefly luciferase signals were normalized to Renilla luciferase. For all luciferase experiments, the obtained results were normalized to the corresponding DMSO control and expressed as fold change. Statistical analysis was then performed on the $\log_2$-transformed values. Depending on the experiment and the groups being compared, either a two-way repeated-measures ANOVA followed by a Šídak's multiple comparison test, or a two-way repeated-measures ANOVA with Geisser–Greenhouse correction followed by a Tukey's multiple comparison test with individual variances for each comparison, was used. Calculations were performed in GraphPad Prism (v10.6.1).

2.4 Monitoring protein degradation using Ub^{G76V}-GFP reporter cells

U2OS cells stably expressing the Ub-G76V-GFP reporter were seeded into 96-well plates at and cultured overnight under standard conditions. On the following day, cells were treated with the indicated test compounds or DMSO vehicle control for 8 hours. After treatment, GFP fluorescence intensity was measured, providing a readout of proteasome-dependent degradation of the Ub-G76V-GFP substrate. Cell viability was then assessed in the same wells using the alamarBlue assay according to the manufacturer's protocol. Viability values were used for normalization of fluorescence data to account for differences in cell number. Accumulation of GFP signal relative to DMSO-treated controls was interpreted as reduced degradation of UPS substrates, indicating potential effects of the compounds on the natural protein turnover. Statistical comparison of RUN compounds with ASC-JM17 was performed using a multiple unpaired t-test with Welch's correction, with adjustment for multiple comparisons by the Holm–Šídák method ($\alpha = 0.05$) in GraphPad Prism (v 10.6.1).

2.5 Quantitative RT-PCR

HEK-293 cells were seeded into 12-well plates and allowed to adhere overnight under standard culture conditions. On the following day, cells were treated with the indicated test compounds or DMSO vehicle control for 16 h. After treatment, cells were lysed directly in the wells, and the resulting lysates were collected for downstream RNA extraction. RNA was extracted using the RNeasy® Plus Micro Kit according to the manufacturer's instructions. Complementary DNA (cDNA) was synthesized from RNA using either the TATAA GrandScript cDNA Supermix (TATAA Biocenter) or the QuantiTect® Reverse Transcription Kit (Qiagen), following the respective protocols. Quantitative RT-PCR was performed on a LightCycler® 480 instrument (Roche Life Science) using the TATAA SYBR® GrandMaster® Mix (TATAA Biocenter). Primer sequences are listed in Table S1. Gene expression levels were normalized to RPLP0, and relative expression was calculated using the $\Delta\Delta$Ct method. Data were expressed as fold change relative to the corresponding DMSO control. Statistical analysis of changes in gene expression was performed with a ratio paired t-test corrected for multiple comparisons using Holm-Šídák's method ($\alpha = 0.05$) in GraphPad Prism (v 10.6.1).

Table S1. Sequences of primer pairs used in the qRT-PCR analysis of gene expression.

Gene	Primer	Sequence (5'- 3')
PMSB7	forward	TGCAAAGAGGGGATACAAGC
	reverse	GCAACAACCATCCCTTCAGT
PMSC4	forward	GGAAGACCATGTTGGCAAAG
	reverse	AAGATGATGGCAGGTGCATT
PSMD12	forward	GTGCGCGACTGACTAAAACA
	reverse	TAGGCAGAGCCTCATTTGCT
PSMB4	forward	GAAGCGTTTTTGGGGTCGC
	reverse	GAGTGGACGGAATGCGGTA
HSPA1A	forward	GCTGCGACAGTCCACTACCT
	reverse	TGCCGGTTCCCTGCTCTCTG
DNAJA1	forward	TCGACGAGGAGAAGACCTTT
	reverse	TCTGACCTGGATGAGAGGTG
NFE2L1	forward	GCCCTGTTTCACTTATAGGGTCTAGA
	reverse	GGCAAAGAGAACATTTAGCAGCTT
SQSTM1	forward	CGTCTGCCCAGACTACGACT
	reverse	GTGTCCGTGTTTCACCTTCC
RPLP0	forward	CACCATTGAAATCCTGAGTGATG
	reverse	TGACCAGCCCAAAGGAGAAG

2.6 Immunoblot analysis

HEK293 cells were treated with 7.5 µM RUN compounds for 16 h, then harvested and resuspended in lysis buffer [50 mM HEPES (pH 7.5), 5 mM EDTA, 150 mM NaCl, 2 mM ATP, and 1% Triton]. Cell lysates were subsequently centrifuged at 15,000 rpm and 4°C for 15 minutes, and then a protein concentration was determined using the Pierce BCA Protein Assay Kit (Thermo Fisher Scientific). Cell lysates were boiled for 5 min at 95 °C in a 6xSDS sample buffer [350 mM Tris-HCl (pH 6.8), 350 mM SDS, 30% (v/v) glycerol, 4% β-mercaptoethanol, 180 µM bromophenol blue]. Prepared samples (15 µg of proteins) were separated by SDS-PAGE (10, 12, or 16% Bis-Tris gels; 50 V for 5 min, then 200 V) and transferred onto a 0.45 µm nitrocellulose membrane (Bio-Rad). For LC3 level analysis, the 0.2 µm nitrocellulose membrane (Bio-Rad) was used. Membranes were blocked in Intercept® (TBS) Blocking Buffer (LI-COR Biosciences) for 1 h at room temperature and then incubated with primary antibodies in Intercept® (TBS) Blocking Buffer with 0.1% Tween-20 overnight at 4°C. Used primary antibodies: anti-NFE2L1/TCF11/SKN1/NRF1 monoclonal antibody (Cell Signalling; #8052; 1:750), anti-HSPA1A/HSP70-1 polyclonal antibody (Sigma-Aldrich; #AV33096; 1:1.000), anti-NQO1 polyclonal antibody (Sigma-Aldrich; # HPA007308; 1:5.000), anti-LC3 antibody

(Sigma-Aldrich; #L8918), anti-p62/SQSTM antibody (Sigma-Aldrich; #P0067), anti-β-actin monoclonal antibody (Sigma-Aldrich; #A5441; 1:10.000). The resulting membranes were washed and incubated with near-infrared fluorophore-conjugated goat anti-mouse IRDye 680RD (LI-COR Biosciences; #926-68070; 1:15.000) and goat anti-rabbit IRDye 800CW (LI-COR Biosciences; #926-32211; 1:15.000) for 1 h at room temperature. Membranes were imaged using Odyssey® M NIR Imaging System (LI-COR Biosciences) and quantified using Image Studio software v. 6.0 (LI-COR Biosciences). The protein levels were quantified by measuring the integrated fluorescence of the corresponding protein bands and normalized to the β-actin loading control. Changes in protein levels after treatment with RUN compounds were analyzed using paired t-tests with correction for multiple comparisons using the Holm-Šídák method ($\alpha = 0.05$) in GraphPad Prism (v 10.6.1).

2.7 Proteasome activity assay

The corresponding cell lines (HEK293, MCF7, or SH-SY5Y) were seeded into 6-well plates and allowed to adhere overnight under standard culture conditions. On the following day, HEK293 cells were treated with 7.5 µM RUN compounds, whereas MCF7 and SH-SY5Y cells were treated with 5 µM RUN compounds, for 16 hours each. After treatment, cells were harvested, resuspended in lysis buffer (50 mM HEPES, pH 7.5; 5 mM EDTA; 150 mM NaCl; 2 mM ATP; 1% Triton), and centrifuged at 15,000 rpm for 15 minutes at 4 °C. The resulting lysates (25 µg total protein) were incubated with 200 µM of the fluorogenic substrate Suc-Leu-Leu-Val-Tyr-AMC (Bachem, I1395) in assay buffer (50 mM Tris, pH 8.0; 10 mM $MgCl_2$; 1 mM ATP; 1 mM DTT) at 37 °C. Free AMC release was detected by measuring fluorescence at 360/460 nm. Results of RUN compounds were compared to the DMSO control using a ratio paired t-test corrected for multiple comparisons with the Holm-Šídák's method ($\alpha = 0.05$) in GraphPad Prism (v 10.6.1).

2.8 XBP1-endoplasmic reticulum (ER) stress reporter assay

HEK293 cells were seeded in 6-well plates and incubated for 24 hours before being transfected with a plasmid containing the CMV-XBP-Gluc reporter using Lipofectamine 2000.(4) After 6 hours, the transfected cells were subsequently transferred to 384-well plates, stabilized overnight, and treated with the proteasome inhibitor and ER stress inducer MG132 and/or various concentrations of RUN compounds (1, 5, and 10 µM) or vehicle (DMSO) for 16 hours. Luciferase activity was normalized to cell viability, which was assessed using the alamarBlue assay. The results are expressed as the fold change compared with the DMSO control, with the experiment conducted in biological triplicate. Two sets of comparisons: 1) treatment with MG132 inhibitor or RUN compounds with DMSO control, 2) RUN compounds vs MG132, were done by two-way repeated-measures ANOVA with Geisser–Greenhouse correction, followed by Dunnett's multiple comparisons test with individual variances in GraphPad Prism (v 10.6.1).

2.9 PolyQ toxicity assay

HCT116 parental cells (WT) and two independent DDI2 knockout clones (KO 04 and KO 11), in which NFE2L1 activation is impaired, were transiently transfected with plasmids encoding either a non-aggregating EGFP-HttQ23 or an aggregation-prone EGFP-HttQ74 construct using Lipofectamine 2000 (Thermo Fisher Scientific) according to the manufacturer's

instructions. After 5–6 h of transfection, cells were seeded into fresh 96-well plates. On the following day, cells were treated with 1 µM or 3 µM RUN-89 or with 0.1% DMSO (vehicle control) for 48 h. Cell viability was determined using the alamarBlue Cell Viability Reagent, and fluorescence was measured at 560/590 nm on a plate reader. Data were expressed as a fold change relative to the corresponding DMSO control. Changes in viability were analyzed using two-way repeated-measures ANOVA followed by Šídak's multiple comparison test on $\log_2$-transformed values in GraphPad Prism (v 10.6.1).

2.10 Oxidative stress detection

Cells were cultured in 96-well plates and washed with prewarmed HBSS buffer. A solution of 2',7'-dichlorofluorescein-diacetate (DCFH-DA) (100 µM) was added to the cells, followed by 30 minutes of incubation. The cells were then treated with RUN compounds (5 and 25 µM) dissolved in HBSS buffer for an additional 2 hours. Control cells received vehicle (DMSO), or tert-butyl hydroperoxide (TBHP), which is known for its ability to induce ROS. The fluorescence was read at 485/530 nm. Results were normalized to the relative DMSO vehicle and statistically analyzed using multiple ratio paired t-test with Holm–Šídák correction for multiple comparisons ($\alpha = 0.05$) in GraphPad Prism (v 10.6.1).

2.11 SNCA-EGFP degradation

SH-SY5Y cells stably expressing SNCA-EGFP were seeded on a plate and allowed to adhere overnight. The next day, they were exposed to proteasome activators PD169316 (3 µM), tadalafil (5 µM), or RUN compounds at a concentration of 1 µM or treated with an equivalent volume of DMSO dissolved in buffer as a vehicle control. The treatment was performed for 16 hours and then fluorescence was measured. The GFP signal intensity was normalized to both the DMSO control and the cell count. Decrease in fluorescence signal was verified using paired t-tests with Holm–Šídák correction for multiple comparisons ($\alpha = 0.05$) in GraphPad Prism (v 10.6.1).

2.12 Analysis of polyQ aggregation in cells

U2OS cells were plated onto slides in a 4-well plate and incubated overnight to facilitate attachment and stabilization. The following day, the cells were transfected with an EGFP-HttQ74 encoding vector using Lipofectamine 2000. After 6 hours, the medium was replaced with fresh culture medium, and the cells were treated for an additional 24 hours with RUN compounds (5 µM), DMSO, and the caspase inhibitor BOC D FMK. The cells were fixed in 4% paraformaldehyde in PBS for 15 minutes, followed by rinsing with PBS. After fixation, the cells were incubated in PBS supplemented with 0.1% Triton X-100 and Hoechst 33258 fluorescent dye (1 µg/mL) for 20 minutes at room temperature. Mounting medium was applied to the slides, and the cells were examined with the Nikon CSU-W1 spinning disc microscope. The images were taken at the same time on the same day, with all conditions kept identical at the Imaging Methods Core Facility at BIOCEV. The analysis workflow incorporated a variety of tools for image processing and statistical analysis. Huygens software was employed to perform deconvolution on the green channel, and a template was used to resolve aggregates. In Fiji, a macro was used to create a maximum intensity projection of the blue channel for segmentation with Stardist, alongside DIC offset correction. Another Fiji macro determined the

image threshold via Otsu's method on a stack, with a global threshold derived from the mean of individual thresholds. Cell segmentation was executed using a Python script with Cellpose, while aggregate segmentation was also carried out. Statistical analysis, including t-tests on the means per well, was performed in GraphPad Prism (v 10.6.1).

2.13 *C. elegans* strains and maintenance

The animals were kept at 20 °C according to standard cultivation conditions (Biron & Haspel, 2015). *Wild-type* N2 (Bristol), AM140 (*rmIs132* [*unc-54p::Q35::YFP*]), and GR2183 (*mgIs72* [*rpt-3p::GFP* + *dpy-5*(+)] II) of *Caenorhabditis elegans* strains and *E. coli* strain OP50 were obtained from the Caenorhabditis Genetic Centre (CGC, University of Minnesota, http://cbs.umn.edu/cgc/home).

2.14 Microscopy of *C. elegans*

Transgenic GR2183 worms carrying the reporter gene (rpt-3p::GFP) were exposed to **RUN-89** for 24 hours and for AM140 (*rmIs132* [*unc-54p::Q35::YFP*]) to aged 6 days under treatment to establish protein aggregation model. The worms were then anesthetized with 20 µM sodium azide and placed on glass slides with 5% agar for fluorescence microscope observation. The expression of the rpt-3p::GFP reporter in worms was directly related to the intensity of green fluorescence. Images were captured using a Zeiss Axioskop 2 Plus fluorescence microscope and a Zeiss Axio Observer A1 microscope designed for epifluorescence. Image analysis was performed to assess variations in aggregation (both the count and volume of aggregates) and fluorescence signal intensity using ImageJ software (https://imagej.nih.gov/ij/). Observed changes after RUN-89 treatment were compared by a Kolmogorov-Smirnov test performed in GraphPad Prism (v 10.6.1).

2.15 Acute juglone/paraquat-induced oxidative stress resistance in *C. elegans*

Eggs of the wild-type strain (N2) were placed in liquid S-basal medium supplemented with *E. coli* OP50 as a food source. Upon reaching the young adult stage, they were exposed to either 50 µM RUN 89 or an equivalent volume of solvent (control group) and incubated at 20 °C for 24 hours. Approximately 130 animals were placed on plates containing either 180 µM juglone or 200 µM paraquat to induce severe oxidative stress.(5, 6) The number of dead animals was recorded each hour throughout the 12-hour experiment. The data were then analyzed using Kaplan-Meier survival analysis, and the results were examined through this method. Survival curves were compared via the Gehan-Breslow-Wilcoxon test or the log-rank (Mantel-Cox) test in GraphPad Prism software (v 10.6.1).

2.16 Analysis of proteasome genes expression in NFE2L1/NFE2L2-knockdown cells

The corresponding HEK293 cells were seeded into 24-well culture plates (120,000 cells/well) and allowed to adhere overnight under standard culture conditions. On the following day, cells were transfected with siRNAs targeting NFE2L1, NFE2L2, or a non-targeting negative control siRNA (Table S2). Transfections were performed using a lipid nanoparticle (LNP) delivery system assembled as previously described,(7) with a final siRNA concentration of ~0.2 nmol per well. After 24 h of incubation, cells were treated with 7.5 µM **RUN-47** or DMSO (vehicle control) for 16 h. Total RNA was then extracted, converted into cDNA, and analyzed by RT-qPCR using primers targeting proteasome genes (listed in Section 2.5, Quantitative RT-PCR, Table S1). Expression levels were normalized to RPLP0 mRNA. The data were $\log_2$-transformed prior to statistical analysis. A two-way RM ANOVA with Geisser–Greenhouse correction was applied, followed by Tukey's multiple comparison test with individual variances calculated for each comparison.

Table S2. List of siRNAs.

siRNA	Source	Commercial Name	Cat. Num.	Sequence (5' → 3')
negative control (NC)	Santa Cruz Biotechnology, Inc.	Control siRNA-A	sc-37007	scrambled sequence
NFEL2L1	Eurofins Genomics	-	-	(GGG AUU CGG UGA AGA UUU G)TT
NFEL2L2	Santa Cruz Biotechnology, Inc.	Nrf2 siRNA (h)	sc-37030	pool of 3 target-specific 19-25 nt siRNAs

3 Chemistry Experimental Procedures

Safety statement: No unexpected or unusually high safety hazards were encountered.

3.1 Experimental details for prepared compounds

All reactions were carried out under argon in dry solvents. All the starting materials were used as purchased from Sigma Aldrich, Combi-Blocks, TCI, Thermo Fisher Scientific or Fluorochem. Revers-phase chromatography was performed using the flash chromatography Teledyne ISCO Combi Flash NextGen 300+ with RediSep Rf Gold C18 reversed-phase columns. Preparative HPLC was performed using the ECOM TOY18DAD800 (the flow rate of 15 mL/min, the gradient of 0–60% or 0–80%, H_2O (0.1% TFA)/MeCN in 60 min) with ProntoSIL 120-10-C18 ace-EPS column, 10 µm, 20 mm × 250 mm. The purity of compounds and the composition of the reaction mixtures were tested on a Waters UPLC-MS Acquity with QDa Mass Detector (the flow rate of 0.5 mL/min, the gradient of 0–100%, H_2O (0.1% formic acid)/MeCN in 7 min) with an ACQUITY UPLC BEH C18 Column, 130Å, 1.7 µm, 2.1 mm × 100 mm with a 2.1 mm × 5 mm pre-column. NMR spectra were recorded using Bruker Advance IIITM HD 400 MHz Prodigy. ESI high-resolution mass spectra were recorded using a Thermo Scientific LTQ Orbitrap XL (Thermo Fisher Scientific) controlled by MassLynx software.

3.2 List of compounds

3,5-Bis((*E*)-3,4-dimethoxybenzylidene)piperidin-4-one hydrochloride (RUN-84)

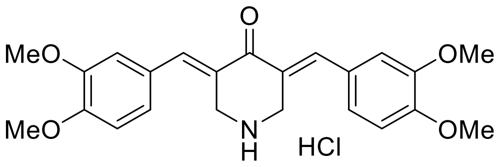

Piperidin-4-one hydrochloride monohydrate (4.00 g, 26.04 mmol) and 3,4-dimethoxybenzaldehyde (8.65 g, 52.08 mmol) were dissolved in ice-cooled glacial acetic acid (20 mL). 4 M solution of HCl in dioxane (4 mL, 16.00 mmol) was added dropwise and the reaction mixture was stirred at ambient temperature overnight. Formed suspension was filtered off and solid was purified by recrystallization from hot methanol. The reaction provided 7.01 g of the title compound in 62% yield. Spectral data agreed with literature values (Sedlacek et al., 2025).

^{1}H NMR (401 MHz, DMSO-d_6) δ 9.87 (br s, 2H), 7.87 – 7.81 (m, 2H), 7.19 – 7.15 (m, 2H), 7.14 – 7.08 (m, 4H), 4.54 – 4.49 (m, 4H), 3.84 (s, 6H), 3.82 (s, 6H). **^{13}C NMR** (101 MHz, DMSO-d_6) δ 182.0, 150.6, 148.7, 139.2, 126.5, 125.7, 124.2, 114.4, 111.8, 55.7, 55.7, 43.9. **HRMS** (ESI+): m/z calculated for $C_{23}H_{26}O_5N$ = 396.1806; found = 396.1807 $[M+H]^+$.

3,5-Bis((*E*)-3,4-dimethoxybenzylidene)-4-oxo-*N*-(pyridin-3-yl)piperidine-1-carbothioamide (RUN-73)

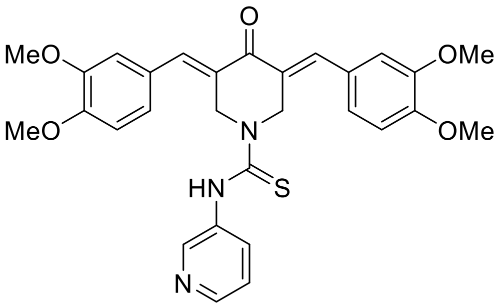

To a mixture of 3,5-bis((*E*)-3,4-dimethoxybenzylidene)-piperidin-4-one hydrochloride (**RUN-84**) (0.86 g, 2.00 mmol), Et_3N (0.42 mL, 3.00 mmol) in THF (31 mL) was added 3-isothiocyanatopyridine (0.30 g, 2.20 mmol) and the reaction mixture was stirred at ambient temperature overnight. Formed suspension was filtered off and the filter cake was transferred to a flask by dissolving in

DCM. Solvent was evaporated and the solid residue was recrystallized from hot methanol. The reaction provided 1.00 g of the title compound in 94% yield.
^{1}H NMR (401 MHz, DMSO-*d*$_6$) δ 9.78 (s, 1H), 8.37 (d, *J* = 2.5 Hz, 1H), 8.28 (dd, *J* = 4.8, 1.5 Hz, 1H), 7.70 – 7.65 (m, 2H), 7.62 (ddd, *J* = 8.2, 2.6, 1.5 Hz, 1H), 7.34 – 7.26 (m, 1H), 7.25 – 7.16 (m, 4H), 7.07 (d, *J* = 8.4 Hz, 2H), 5.37 (s, 4H), 3.82 (s, 6H), 3.81 (s, 6H). **^{13}C NMR** (101 MHz, DMSO-*d*$_6$) δ 185.9, 182.5, 150.2, 148.6, 146.7, 145.5, 137.6, 136.6, 133.0, 130.0, 127.1, 124.3, 123.0, 114.4, 111.7, 55.6, 55.6, 49.3. **HRMS** (ESI+): m/z calculated for $C_{29}H_{30}O_5N_3S$ = 532.1901; found = 532.1902 $[M+H]^+$.

Ethyl (3,5-bis((*E*)-3,4-dimethoxybenzylidene)-4-oxopiperidine-1-carbonyl)glycinate (RUN-74)

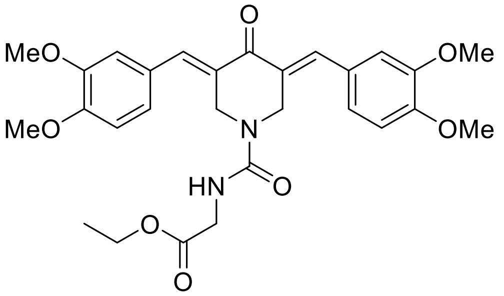

To a mixture of 3,5-bis((*E*)-3,4-dimethoxybenzylidene)-piperidin-4-one hydrochloride (**RUN-84**) (0.86 g, 2.00 mmol), Et_3N (0.42 mL, 3.00 mmol) in THF (31 mL) was added ethyl 2-isocyanatoacetate (0.28 g, 2.20 mmol) and the reaction mixture was stirred at ambient temperature overnight. Reaction mixture was diluted with water and extracted with DCM. Organic phases were combined, washed with brine, dried over $MgSO_4$ and filtrated. Solvent was evaporated and the solid residue was recrystallized from hot methanol. The reaction provided 0.97 g of the title compound in 92% yield.
^{1}H NMR (401 MHz, DMSO-*d*$_6$) δ 7.61 (s, 2H), 7.31 (t, *J* = 5.8 Hz, 1H), 7.18 – 7.11 (m, 4H), 7.07 (d, *J* = 8.7 Hz, 2H), 4.75 (s, 4H), 4.02 (q, *J* = 7.1 Hz, 2H), 3.83 (s, 12H), 3.64 (d, *J* = 5.5 Hz, 2H), 1.13 (t, *J* = 7.1 Hz, 3H). **^{13}C NMR** (101 MHz, DMSO-*d*$_6$) δ 186.2, 170.8, 157.1, 150.1, 148.6, 135.8, 131.1, 127.3, 124.2, 114.4, 111.7, 60.1, 55.6, 55.6, 45.0, 42.3, 14.1. **HRMS** (ESI+): m/z calculated for $C_{28}H_{32}O_8N_2Na$ = 547.2051; found = 547.2052 $[M+Na]^+$.

Ethyl 3-(3,5-bis((*E*)-3,4-dimethoxybenzylidene)-4-oxopiperidine-1-carboxamido)propanoate (RUN-75)

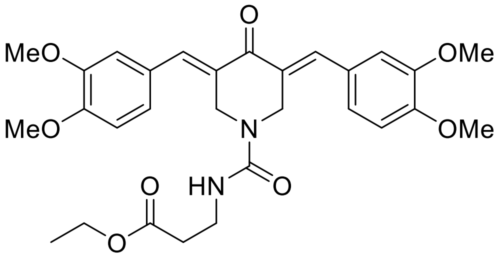

To a mixture of 3,5-bis((*E*)-3,4-dimethoxybenzylidene)-piperidin-4-one hydrochloride (**RUN-84**) (0.86 g, 2.00 mmol), Et_3N (0.42 mL, 3.00 mmol) in THF (20 mL) was added ethyl 3-isocyanatopropanoate (0.32 g, 2.20 mmol) and the reaction mixture was stirred at ambient temperature overnight. Reaction mixture was diluted with water and extracted with DCM. Organic phases were combined, washed with brine, dried over $MgSO_4$ and filtrated. Solvent was evaporated and the solid residue was recrystallized from hot methanol. The reaction provided 1.00 g of the title compound in 93% yield.
^{1}H NMR (400 MHz, DMSO-*d*$_6$) δ 7.62 – 7.56 (m, 2H), 7.16 – 7.12 (m, 4H), 7.10 – 7.06 (m, 2H), 6.89 (t, *J* = 5.5 Hz, 1H), 4.70 (s, 4H), 3.98 (q, *J* = 7.1 Hz, 2H), 3.83 (s, 6H), 3.82 (s, 6H), 3.23 – 3.13 (m, 2H), 2.36 (t, *J* = 7.0 Hz, 2H), 1.12 (t, *J* = 7.1 Hz, 3H). **^{13}C NMR** (101 MHz, DMSO-*d*$_6$) δ 186.3, 171.4, 157.0, 150.0, 148.6, 135.7, 131.2, 127.3, 124.1, 114.4, 111.7, 59.7,

55.6, 55.6, 45.0, 36.5, 34.5, 14.0. **HRMS** (ESI+): m/z calculated for $C_{29}H_{34}O_8N_2Na$ = 561.2207; found = 561.2208 $[M+Na]^+$.

3,5-Bis((*E*)-3,4-dimethoxybenzylidene)-*N*-(3-methoxyphenyl)-4-oxopiperidine-1-carboxamide (RUN-76)

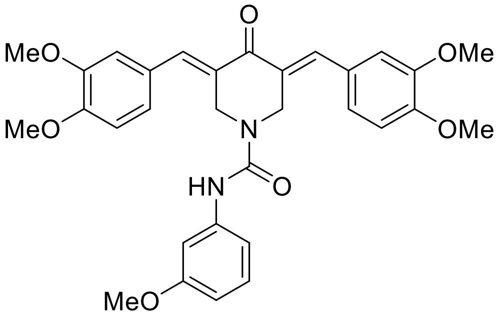

To a mixture of 3,5-bis((*E*)-3,4-dimethoxybenzylidene)-piperidin-4-one hydrochloride (**RUN-84**) (0.30 g, 0.70 mmol), Et_3N (0.15 mL, 1.04 mmol) in THF (8 mL) was added 1-isocyanato-3-methoxybenzene (0.11 g, 0.76 mmol) and the reaction mixture was stirred at ambient temperature overnight. Reaction mixture was diluted with water and extracted with DCM. Organic phases were combined, washed with brine, dried over $MgSO_4$ and filtrated. Solvent was evaporated and the solid residue was recrystallized from hot methanol. The reaction provided 0.35 g of the title compound in 92% yield.

^{1}H NMR (401 MHz, DMSO-d_6) δ 8.87 (s, 1H), 7.64 (s, 2H), 7.21 – 7.14 (m, 4H), 7.13 – 7.04 (m, 3H), 6.99 (t, J = 2.3 Hz, 1H), 6.86 (dd, J = 8.1, 1.9 Hz, 1H), 6.50 (dd, J = 8.3, 2.5 Hz, 1H), 4.87 (s, 4H), 3.84 (s, 6H), 3.83 (s, 6H), 3.66 (s, 3H). **^{13}C NMR** (101 MHz, DMSO-d_6) δ 186.3, 159.4, 155.1, 150.1, 148.6, 141.3, 135.9, 131.1, 129.0, 127.3, 124.1, 114.5, 112.0, 111.7, 107.6, 105.4, 55.6, 54.9, 45.6. **HRMS** (ESI+): m/z calculated for $C_{31}H_{33}O_7N_2$ = 545.2282; found = 545.2284 $[M+H]^+$.

3,5-Bis((*E*)-3,4-dimethoxybenzylidene)-*N*-(2-morpholinoethyl)-4-oxopiperidine-1-carbothioamide (RUN-77)

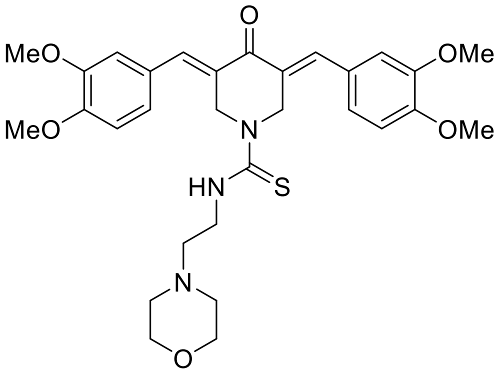

To a mixture of 3,5-bis((*E*)-3,4-dimethoxybenzylidene)-piperidin-4-one hydrochloride (**RUN-84**) (0.30 g, 0.70 mmol), Et_3N (0.15 mL, 1.04 mmol) in THF (8 mL) was added 4-(2-isothiocyanatoethyl)morpholine (0.13 g, 0.76 mmol) and the reaction mixture was stirred at ambient temperature overnight. Formed suspension was filtered off and the filter cake was recrystallized from hot MeOH:H_2O (9:1) mixture. The reaction provided 0.24 g of the title compound in 60% yield.

^{1}H NMR (401 MHz, DMSO-d_6) δ 7.72 (t, J = 5.2 Hz, 1H), 7.62 (s, 2H), 7.23 – 7.14 (m, 4H), 7.08 (d, J = 8.4 Hz, 2H), 5.21 (s, 4H), 3.83 (s, 12H), 3.57 – 3.48 (m, 2H), 3.41 (t, J = 4.6 Hz, 4H), 2.35 (t, J = 6.9 Hz, 2H), 2.27 (t, J = 4.7 Hz, 4H). **^{13}C NMR** (101 MHz, DMSO-d_6) δ 186.1, 181.6, 150.2, 148.6, 136.3, 130.4, 127.2, 124.2, 114.4, 111.7, 66.0, 56.5, 55.6, 55.6, 53.2, 48.5, 42.6. **HRMS** (ESI+): m/z calculated for $C_{30}H_{38}O_6N_3S$ = 568.2476; found = 568.2472 $[M+H]^+$.

3,5-Bis((*E*)-3,4-dimethoxybenzylidene)-1-(2-(2-(2-methoxyethoxy)ethoxy)acetyl)piperidin-4-one (RUN-82)

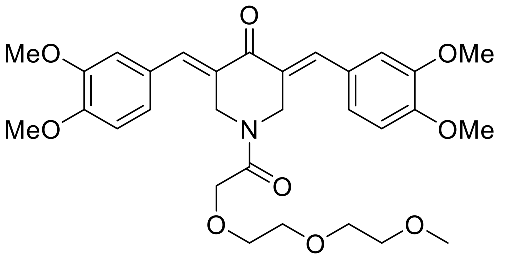

To a mixture of 3,5-bis((*E*)-3,4-dimethoxybenzylidene)-piperidin-4-one hydrochloride (**RUN-84**) (0.10 g, 0.23 mmol) and Et_3N (0.10 mL, 0.70 mmol) in DMF (3 mL) was added HBTU (0.12 g, 0.32 mmol) and 2-(2-(2-methoxyethoxy) ethoxy)acetic acid (0.06 g, 0.32 mmol). The reaction mixture was stirred 2 days at ambient temperature. Solvent was evaporated under reduced pressure and the residue was purified by reverse-phase flash chromatography (eluent H_2O:MeCN, gradient elution). The reaction provided 0.08 g of the title compound in 64% yield.

^{1}H NMR (401 MHz, DMSO-*d6*) δ 7.70 – 7.64 (m, 2H), 7.21 – 7.05 (m, 6H), 4.88 – 4.80 (m, 4H), 4.08 (s, 2H), 3.83 (s, 6H), 3.82 (s, 6H), 3.42 – 3.37 (m, 2H), 3.36 – 3.32 (m, 6H), 3.18 (s, 3H). **^{13}C NMR** (101 MHz, DMSO-*d*6) δ 185.5, 167.7, 150.2, 148.6, 136.5, 130.5, 127.1, 123.9, 114.3, 111.7, 71.2, 69.8, 69.6, 69.5, 69.4, 58.0, 55.6, 45.6, 42.6. **HRMS** (ESI+): m/z calculated for $C_{30}H_{37}O_9NNa$ = 578.2361; found = 578.2359 $[M+Na]^+$.

3-(3,5-Bis((*E*)-3,4-dimethoxybenzylidene)-4-oxopiperidine-1-carbonyl) quinuclidin-1-ium trifluoroacetate (RUN-83)

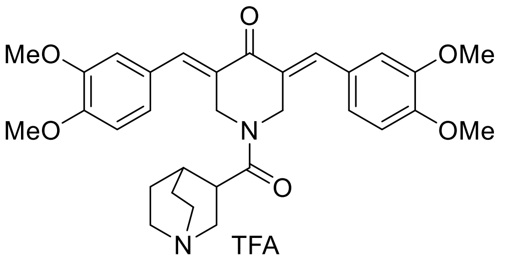

To a mixture of 3,5-bis((*E*)-3,4-dimethoxybenzylidene)-piperidin-4-one hydrochloride (**RUN-84**) (0.10 g, 0.23 mmol) and Et_3N (0.13 mL, 0.93 mmol) in DMF (3 mL) was added HBTU (0.12 g, 0.32 mmol) and quinuclidine-3-carboxylic acid hydrochloride (0.06 g, 0.32 mmol). The reaction mixture was stirred 2 days at ambient temperature. Solvent was evaporated under reduced pressure and the residue was purified by reverse-phase flash chromatography (eluent H_2O (0.1% TFA):MeCN, gradient elution). The reaction provided 0.09 g of the title compound in 63% yield.

^{1}H NMR (401 MHz, DMSO-*d*6) δ 9.55 (s, 1H), 7.72 – 7.67 (m, 2H), 7.21 – 7.13 (m, 4H), 7.13 – 7.07 (m, 2H), 5.06 – 4.76 (m, 4H), 3.84 (s, 9H), 3.82 (s, 3H), 3.58 – 3.49 (m, 1H), 3.29 – 3.00 (m, 6H), 1.96 – 1.86 (m, 1H), 1.64 – 1.52 (m, 2H), 1.52 – 1.40 (m, 1H), 1.35 – 1.30 (m, 1H). **^{13}C NMR** (101 MHz, DMSO-*d*6) δ 185.4, 169.3, 158.0 (q, J = 32.2 Hz), 150.3, 148.7, 148.7, 136.7, 136.6, 130.6, 130.3, 127.1, 126.9, 124.2, 124.0, 114.4, 114.0, 111.7, 55.7, 55.6, 47.6, 45.9, 45.4, 45.3, 43.2, 35.7, 22.3, 22.1, 18.6. **HRMS** (ESI+): m/z calculated for $C_{31}H_{37}O_6N_2$ = 533.2646; found = 533.2644 $[M+H]^+$.

2-(3,5-Bis((*E*)-3,4-dimethoxybenzylidene)-4-oxopiperidin-1-yl)-2-oxoethan-1-aminium trifluoroacetate (RUN-85)

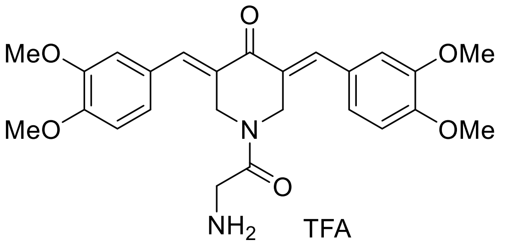

To a mixture of 3,5-bis((*E*)-3,4-dimethoxybenzylidene)-piperidin-4-one hydrochloride (**RUN-84**) (0.10 g, 0.23 mmol) and Et_3N (0.10 mL, 0.70 mmol) in DMF (3 mL) was added HBTU (0.12 g, 0.32 mmol) and (*tert*-butoxycarbonyl) glycine (0.049 g, 0.28 mmol). The reaction mixture was stirred overnight at ambient temperature. Solvent was evaporated under reduced pressure and the residue was purified by reverse-phase flash chromatography (eluent H_2O (0.1% TFA):MeCN, gradient elution) to obtain Boc-protected compound that was dissolved in DCM (0.5 mL), cooled down with ice bath and TFA (0.5 mL) was added dropwise. The solution was stirred at ambient temperature for 1 hour. Solvent was evaporated under reduced pressure and the residue was purified by reverse-phase preparative HPLC. The reaction provided 0.05 g of the title compound in 35% yield.

^{1}H NMR (401 MHz, DMSO-d_6) δ 7.96 – 7.89 (m, 3H), 7.70 (s, 1H), 7.67 (s, 1H), 7.22 – 7.12 (m, 4H), 7.12 – 7.06 (m, 2H), 4.91 (s, 2H), 4.80 (s, 2H), 3.91 – 3.84 (m, 2H), 3.84 (s, 3H), 3.84 (s, 6H), 3.82 (s, 3H). **^{13}C NMR** (101 MHz, DMSO-d_6) δ 185.4, 165.1, 150.3, 150.2, 148.7, 148.6, 136.9, 136.6, 130.1, 129.6, 127.0, 126.8, 124.4, 123.9, 114.3, 114.2, 111.8, 111.7, 55.6, 45.6, 42.5, 39.1 (signal from HSQC). **HRMS** (ESI+): m/z calculated for $C_{25}H_{29}O_6N_2$ = 453.2020; found = 453.2019 $[M+H]^+$.

1-(Cyclopentanecarbonyl)-3,5-bis((*E*)-3,4-dimethoxybenzylidene)piperidin-4-one (RUN-103)

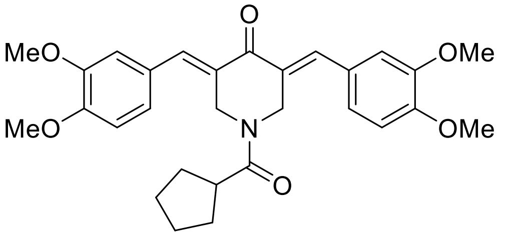

To a mixture of 3,5-bis((*E*)-3,4-dimethoxybenzylidene)-piperidin-4-one hydrochloride (**RUN-84**) (0.09 g, 0.21 mmol) and Et_3N (0.13 mL, 0.75 mmol) in DMF (3 mL) was added HBTU (0.12 g, 0.31 mmol) and cyclopentanecarboxylic acid (0.04 g, 0.31 mmol). The reaction mixture was stirred for 3 hours at ambient temperature. Solvent was evaporated under reduced pressure and the residue was purified by reverse-phase flash chromatography (eluent H_2O (0.1% TFA):MeCN, gradient elution). The reaction provided 0.08 g of the title compound in 77% yield.

^{1}H NMR (401 MHz, $CDCl_3$) δ 7.83 (s, 1H), 7.77 (s, 1H), 7.15 – 6.86 (m, 6H), 4.95 (s, 2H), 4.81 (s, 2H), 3.95 (s, 3H), 3.92 (s, 9H), 2.79 – 2.66 (m, 1H), 1.80 – 1.67 (m, 2H), 1.67 – 1.53 (m, 4H), 1.49 – 1.35 (m, 2H). **^{13}C NMR** (101 MHz, CDCl3) δ 186.7, 175.3, 150.6, 149.2, 149.0, 138.5, 137.3, 130.5, 130.1, 127.9, 127.7, 124.7, 123.7, 114.0, 113.6, 111.3, 56.1, 46.5, 44.0, 40.1, 30.0, 26.1. **HRMS** (ESI+): m/z calculated for $C_{29}H_{33}O_6NNa$ = 514.2200; found = 514.2199 $[M+Na]^+$.

1-(2-(3,5-Bis((*E*)-3,4-dimethoxybenzylidene)-4-oxopiperidin-1-yl)-2-oxoethyl)-4-methylpiperazine-1,4-diium di(trifluoroacetate) (RUN-105)

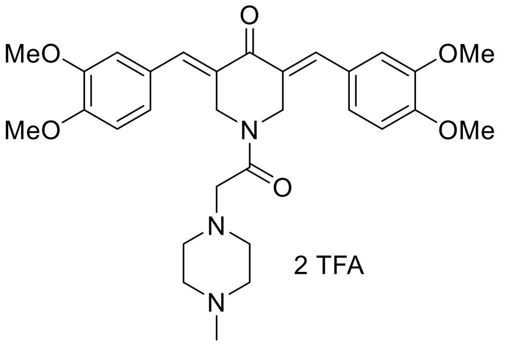

To a mixture of 3,5-bis((*E*)-3,4-dimethoxybenzylidene)-piperidin-4-one hydrochloride (**RUN-84**) (0.09 g, 0.21 mmol) and Et_3N (0.10 mL, 0.75 mmol) in DMF (3 mL) was added HBTU (0.12 g, 0.31 mmol) and 2-(4-methylpiperazin-1-yl)acetic acid (0.05 g, 0.31 mmol). The reaction mixture was stirred for 3 hours at ambient temperature. Solvent was evaporated under reduced pressure and the residue was purified by reverse-phase flash chromatography (eluent H_2O (0.1% TFA):MeCN gradient elution). The reaction provided 0.10 g of the title compound in 60% yield.

^{1}H NMR (401 MHz, DMSO-*d*$_6$) δ 7.73 – 7.66 (m, 2H), 7.26 – 7.12 (m, 4H), 7.09 (d, *J* = 8.4 Hz, 2H), 4.92 (s, 2H), 4.87 (s, 2H), 3.84 (s, 3H), 3.83 (s, 6H), 3.81 (s, 3H), 3.44 (br s, 4H), 3.26 (br s, 4H), 2.69 (s, 5H). **^{13}C NMR** (101 MHz, DMSO-*d*$_6$) δ 185.5, 158.2 (q, *J* = 33.8 Hz), 150.3, 150.2, 148.7, 148.7, 136.6, 136.1, 130.7, 130.4, 127.2, 127.1, 124.1, 123.9, 114.5, 114.3, 111.8, 111.8, 58.5, 55.7, 55.7, 55.6, 51.8, 48.9, 46.1, 42.8, 42.2. **HRMS** (ESI+): m/z calculated for $C_{30}H_{38}O_6N_3$ = 536.2755; found = 536.2753 $[M+H]^+$.

4-(3,5-Bis((*E*)-3,4-dimethoxybenzylidene)-4-oxopiperidin-1-yl)-*N*,*N*-dimethyl-4-oxobutan-1-aminium trifluoroacetate (RUN-106)

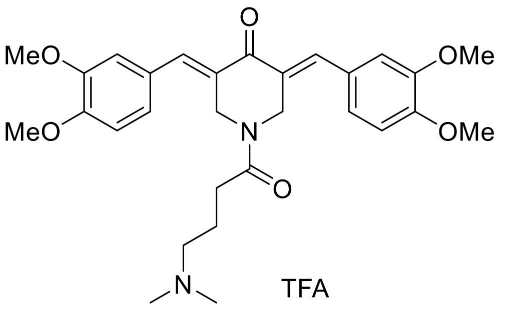

To a mixture of 3,5-bis((*E*)-3,4-dimethoxybenzylidene)-piperidin-4-one hydrochloride (**RUN-84**) (0.09 g, 0.21 mmol) and Et_3N (0.13 mL, 0.94 mmol) in DMF (3 mL) was added HBTU (0.12 g, 0.31 mmol) and 4-(dimethylamino)butanoic acid hydrochloride (0.05 g, 0.31 mmol). The reaction mixture was stirred for 3 hours at ambient temperature. Solvent was evaporated under reduced pressure and the residue was purified by reverse-phase flash chromatography (eluent H_2O (0.1% TFA):MeCN, gradient elution). The reaction provided 0.07 g of the title compound in 56% yield.

^{1}H NMR (401 MHz, DMSO-*d*$_6$) δ 9.49 (br s, 1H), 7.67 (s, 2H), 7.20 – 7.12 (m, 4H), 7.12 – 7.06 (m, 2H), 4.87 (s, 2H), 4.84 (s, 2H), 3.84 (s, 9H), 3.81 (s, 3H), 2.93 (t, *J* = 7.9 Hz, 2H), 2.70 (s, 6H), 2.37 (t, *J* = 6.7 Hz, 2H), 1.80 – 1.69 (m, 2H). **^{13}C NMR** (101 MHz, DMSO-*d*$_6$) δ 185.6, 169.8, 158.0 (q, *J* = 30.6 Hz), 150.2, 150.2, 148.7, 136.4, 136.4, 130.6, 130.3, 127.2, 127.0, 124.1, 123.9, 114.3, 111.7, 56.3, 55.6, 46.2, 42.5, 42.2, 28.6, 19.2. **HRMS** (ESI+): m/z calculated for $C_{29}H_{37}O_6N_2$ = 509.2646; found = 509.2644 $[M+H]^+$.

2-(3,5-Bis((*E*)-3,4-dimethoxybenzylidene)-4-oxopiperidine-1-carbonyl)-3-methylpyridin-1-ium trifluoroacetate (RUN-107)

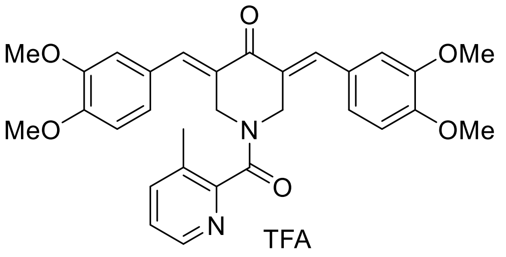

To a mixture of 3,5-bis((*E*)-3,4-dimethoxybenzylidene)-piperidin-4-one hydrochloride (**RUN-84**) (0.09 g, 0.21 mmol) and Et_3N (0.10 mL, 0.75 mmol) in DMF (3 mL) was added HBTU (0.12 g, 0.31 mmol) and 3-methylpicolinic acid (0.04 g, 0.31 mmol). The reaction mixture was stirred for 1 hour at ambient temperature. Solvent was evaporated under reduced pressure and the residue was purified by reverse-phase flash chromatography (eluent H_2O (0.1% TFA):MeCN, gradient elution). The reaction provided 0.11 g of the title compound in 80% yield.

^{1}H NMR (401 MHz, $CDCl_3$) δ 8.46 (br s, 1H), 8.40 – 8.34 (m, 1H), 7.89 – 7.83 (m, 1H), 7.73 – 7.68 (m, 1H), 7.53 – 7.46 (m, 1H), 7.30 – 7.22 (m, 1H), 7.17 (dd, *J* = 8.5, 2.1 Hz, 1H), 7.06 (d, *J* = 2.0 Hz, 1H), 6.98 (d, *J* = 8.4 Hz, 1H), 6.74 (d, *J* = 8.3 Hz, 1H), 6.68 (dd, *J* = 8.4, 2.0 Hz, 1H), 6.62 (d, *J* = 2.0 Hz, 1H), 5.14 (d, *J* = 1.8 Hz, 2H), 4.48 (d, *J* = 1.9 Hz, 2H), 3.95 (s, 6H), 3.88 (s, 3H), 3.79 (s, 3H), 2.15 (s, 3H). **^{13}C NMR** (101 MHz, $CDCl_3$) δ 186.2, 164.8, 160.4 (q, *J* = 39.7 Hz), 150.9, 150.5, 150.1, 149.2, 148.9, 143.5, 141.7, 139.7, 137.6, 132.9, 129.8, 128.7, 127.5, 127.1, 125.0, 124.8, 123.9, 114.2, 112.7, 111.4, 111.0, 56.2, 56.0, 47.0, 43.6, 17.6. **HRMS** (ESI+): m/z calculated for $C_{30}H_{30}O_6N_2Na$ = 537.1996; found = 537.1993 $[M+Na]^+$.

2-(3,5-Bis((*E*)-3,4-dimethoxybenzylidene)-4-oxopiperidine-1-carbonyl)pyridin-1-ium trifluoroacetate (RUN-108)

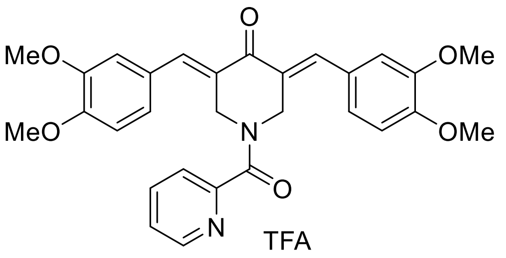

To a mixture of 3,5-bis((*E*)-3,4-dimethoxybenzylidene)-piperidin-4-one hydrochloride (**RUN-84**) (0.09 g, 0.21 mmol) and Et_3N (0.10 mL, 0.75 mmol) in DMF (3 mL) was added HBTU (0.12 g, 0.31 mmol) and picolinic acid (0.04 g, 0.31 mmol). The reaction mixture was stirred for 3 hours at ambient temperature. Solvent was evaporated under reduced pressure and the residue was purified by reverse-phase flash chromatography (eluent H_2O (0.1% TFA):MeCN, gradient elution). The reaction provided 0.11 g of the title compound in 85% yield.

^{1}H NMR (401 MHz, $CDCl_3$) δ 8.42 (ddd, *J* = 5.0, 1.7, 0.9 Hz, 1H), 7.86 (s, 1H), 7.77 (s, 1H), 7.69 (td, *J* = 7.8, 1.7 Hz, 1H), 7.51 (dt, *J* = 7.7, 1.1 Hz, 1H), 7.33 (ddd, *J* = 7.7, 5.0, 1.2 Hz, 1H), 7.17 (d, *J* = 8.4 Hz, 1H), 7.09 (s, 1H), 6.97 (d, *J* = 8.0 Hz, 1H), 6.81 – 6.74 (m, 3H), 5.11 (s, 2H), 4.85 (s, 2H), 3.95 (s, 6H), 3.90 (s, 3H), 3.84 (s, 3H). **^{13}C NMR** (101 MHz, $CDCl_3$) δ 186.5, 166.4, 160.4 (q, *J* = 39.7 Hz), 151.6, 150.8, 150.5, 149.1, 147.1, 139.3, 138.7, 137.7, 129.9, 129.1, 127.6, 127.5, 125.5, 124.6, 124.3, 124.1, 114.1, 113.2, 111.4, 111.1, 56.1, 56.0, 47.8, 44.4. **HRMS** (ESI+): m/z calculated for $C_{29}H_{28}O_6N_2Na$ = 523.1840; found = 523.1835 $[M+Na]^+$.

4-(2-(3,5-Bis((*E*)-3,4-dimethoxybenzylidene)-4-oxopiperidin-1-yl)-2-oxoethyl)-1*H*-imidazol-3-ium trifluoroacetate (RUN-109)

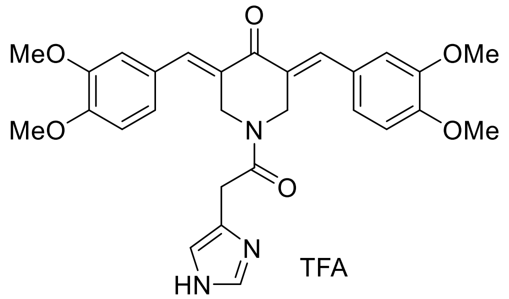

To a mixture of 3,5-bis((*E*)-3,4-dimethoxybenzylidene)-piperidin-4-one hydrochloride (**RUN-84**) (0.09 g, 0.21 mmol) and Et_3N (0.13 mL, 0.94 mmol) in DMF (3 mL) was added HBTU (0.12 g, 0.31 mmol) and 2-(1*H*-imidazol-4-yl)acetic acid hydrochloride (0.05 g, 0.31 mmol). The reaction mixture was stirred for 1.5 hour at ambient temperature. Solvent was evaporated under reduced pressure and the residue was purified by reverse-phase flash chromatography (eluent H_2O (0.1% TFA):MeCN, gradient elution). The reaction provided 0.12 g of the title compound in 93% yield.

^{1}H NMR (401 MHz, $CDCl_3$) δ 8.23 – 8.18 (m, 1H), 7.83 – 7.78 (m, 1H), 7.73 – 7.68 (m, 1H), 7.01 – 6.87 (m, 6H), 6.86 – 6.80 (m, 1H), 4.86 (s, 2H), 4.78 (s, 2H), 3.91 (s, 3H), 3.89 (s, 3H), 3.85 (s, 3H), 3.82 (s, 3H), 3.66 (s, 2H). **^{13}C NMR** (101 MHz, $CDCl_3$) δ 185.8, 166.7, 162.1 (q, *J* = 36.5 Hz), 150.9, 150.7, 149.3, 149.0, 138.7, 138.1, 133.3, 129.3, 127.5, 127.4, 127.1, 124.4, 123.7, 117.7, 114.1, 114.0, 111.4, 111.3, 56.1, 56.0, 56.0, 46.8, 44.1, 29.1. **HRMS** (ESI+): m/z calculated for $C_{28}H_{30}O_6N_3$ = 504.2129; found = 504.2127 $[M+H]^+$.

3,5-Bis((*E*)-3,4-dimethoxybenzylidene)-1-((*S*)-5-oxopyrrolidine-2-carbonyl)piperidin-4-one (RUN-110)

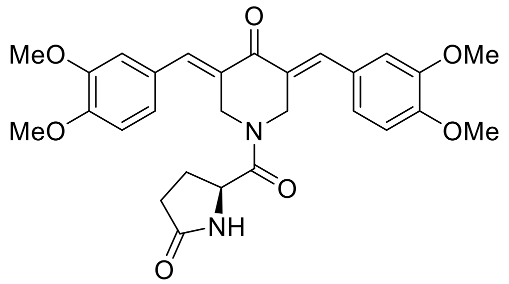

To a mixture of 3,5-bis((*E*)-3,4-dimethoxybenzylidene)-piperidin-4-one hydrochloride (**RUN-84**) (0.09 g, 0.21 mmol) and Et_3N (0.10 mL, 0.75 mmol) in DMF (3 mL) was added HBTU (0.12 g, 0.31 mmol) and L-pyroglutamic acid (0.04 g, 0.31 mmol). The reaction mixture was stirred for 1.5 hour at ambient temperature. Solvent was evaporated under reduced pressure and the residue was purified by reverse-phase flash chromatography (eluent H_2O (0.1% TFA):MeCN, gradient elution). The reaction provided 0.09 g of the title compound in 84% yield.

^{1}H NMR (401 MHz, $CDCl_3$) δ 7.87 (s, 1H), 7.79 (s, 1H), 7.08 (d, *J* = 8.5 Hz, 1H), 7.01 – 6.85 (m, 6H), 5.12 (d, *J* = 16.3 Hz, 1H), 4.76 (d, *J* = 14.9 Hz, 2H), 4.65 (d, *J* = 16.1 Hz, 1H), 4.35 (dd, *J* = 8.7, 5.2 Hz, 1H), 3.95 (s, 3H), 3.92 (s, 3H), 3.91 (s, 6H), 2.41 – 2.18 (m, 2H), 2.11 – 1.89 (m, 2H). **^{13}C NMR** (101 MHz, $CDCl_3$) δ 185.6, 179.3, 169.8, 150.9, 149.4, 149.1, 139.3, 138.0, 129.4, 129.1, 127.5, 127.1, 124.6, 123.4, 114.1, 113.8, 111.4, 111.3, 56.2, 56.1, 54.6, 45.8, 44.4, 29.3, 24.5. **HRMS** (ESI+): m/z calculated for $C_{28}H_{30}O_7N_2Na$ = 529.1945; found = 529.1946 $[M+Na]^+$.

1-(2-(3,5-Bis((*E*)-3,4-dimethoxybenzylidene)-4-oxopiperidin-1-yl)-2-oxoethyl)-1*H*-imidazol-3-ium trifluoroacetate (RUN-111)

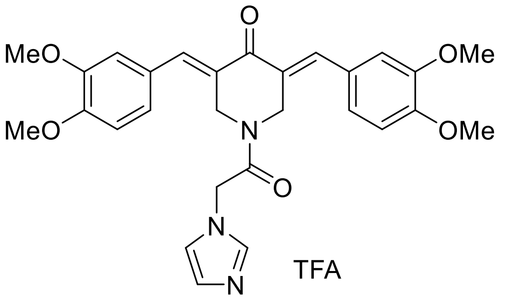

To a mixture of 3,5-bis((*E*)-3,4-dimethoxybenzylidene)-piperidin-4-one hydrochloride (**RUN-84**) (0.09 g, 0.21 mmol) and Et_3N (0.10 mL, 0.75 mmol) in DMF (3 mL) was added HBTU (0.12 g, 0.31 mmol) and 2-(1*H*-imidazol-1-yl)acetic acid (0.04 g, 0.31 mmol). The reaction mixture was stirred for 1.5 hour at ambient temperature. Solvent was evaporated under reduced pressure and the residue was purified by reverse-phase flash chromatography (eluent H_2O (0.1% TFA):MeCN, gradient elution). The reaction provided 0.11 g of the title compound in 82% yield.

^{1}H NMR (401 MHz, $CDCl_3$) δ 9.00 (s, 1H), 7.84 – 7.79 (m, 1H), 7.79 – 7.72 (m, 1H), 7.23 – 7.18 (m, 1H), 7.13 – 7.08 (m, 1H), 7.08 – 6.99 (m, 2H), 6.96 (d, J = 8.4 Hz, 1H), 6.94 – 6.90 (m, 2H), 6.87 (d, J = 8.4 Hz, 1H), 5.23 (s, 2H), 4.93 (s, 2H), 4.83 (s, 2H), 3.91 (s, 3H), 3.90 (s, 4H), 3.89 (s, 3H), 3.86 (s, 3H). **^{13}C NMR** (101 MHz, $CDCl_3$) δ 185.6, 163.4, 161.9 (q, J = 36.0 Hz), 151.0, 150.8, 149.3, 149.1, 139.1, 138.5, 137.1, 128.8, 128.6, 127.4, 126.9, 124.5, 124.0, 122.9, 119.8, 114.1, 114.0, 111.6, 111.3, 56.1, 56.1, 49.7, 46.1, 44.2. **HRMS** (ESI+): m/z calculated for $C_{28}H_{30}O_6N_3$ = 504.2129; found = 504.2128 $[M+H]^+$.

4-(2-(3,5-Bis((*E*)-3,4-dimethoxybenzylidene)-4-oxopiperidin-1-yl)-2-oxoethyl)morpholin-4-ium trifluoroacetate (RUN-112)

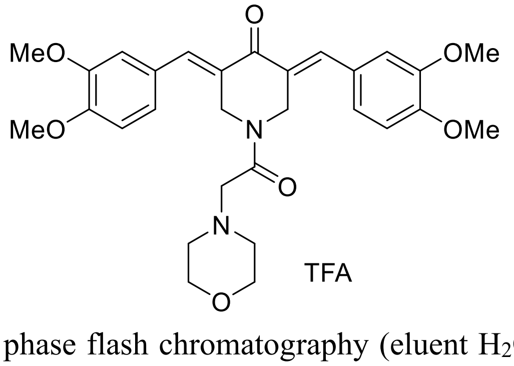

To a mixture of 3,5-bis((*E*)-3,4-dimethoxybenzylidene)-piperidin-4-one hydrochloride (**RUN-84**) (0.09 g, 0.21 mmol) and Et_3N (0.13 mL, 0.94 mmol) in DMF (3 mL) was added HBTU (0.12 g, 0.31 mmol) and 2-morpholinoacetic acid hydrochloride (0.06 g, 0.31 mmol). The reaction mixture was stirred for 2 hours at ambient temperature. Solvent was evaporated under reduced pressure and the residue was purified by reverse-phase flash chromatography (eluent H_2O (0.1% TFA):MeCN, gradient elution). The reaction provided 0.12 g of the title compound in 91% yield.

^{1}H NMR (401 MHz, $CDCl_3$) δ 7.82 – 7.73 (m, 2H), 7.07 – 6.95 (m, 2H), 6.98 – 6.93 (m, 2H), 6.93 – 6.87 (m, 2H), 4.92 (s, 2H), 4.66 (s, 2H), 3.97 (s, 2H), 3.95 – 3.91 (m, 10H), 3.90 (s, 6H), 3.34 (br s, 4H). **^{13}C NMR** (101 MHz, $CDCl_3$) δ 185.4, 162.2 (q, J = 37.5 Hz) 162.1, 151.1, 150.9, 149.4, 149.1, 139.3, 138.4, 128.6, 128.5, 127.3, 126.9, 124.5, 123.8, 114.1, 114.0, 111.5, 111.3, 64.1, 56.2, 56.1, 56.1, 52.8, 46.2, 43.8. **HRMS** (ESI+): m/z calculated for $C_{29}H_{35}O_7N_2$ = 523.2439; found = 523.2438 $[M+H]^+$.

(*S*)-2-(3,5-Bis((*E*)-3,4-dimethoxybenzylidene)-4-oxopiperidine-1-carbonyl)pyrrolidin-1-ium trifluoroacetate (RUN-113)

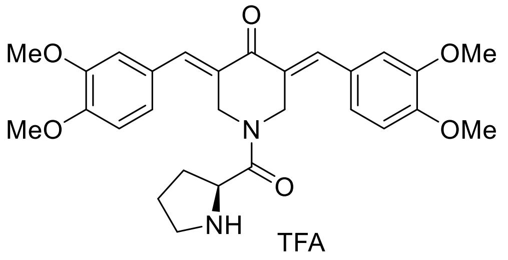

To a mixture of 3,5-bis((*E*)-3,4-dimethoxybenzylidene)-piperidin-4-one hydrochloride (**RUN-84**) (0.09 g, 0.21 mmol) and Et_3N (0.10 mL, 0.75 mmol) in DMF (3 mL) was added HBTU (0.12 g, 0.31 mmol) and (*tert*-butoxycarbonyl)-L-proline (0.07 g, 0.31 mmol). The reaction mixture was stirred 1.5 hour at ambient temperature. Solvent was evaporated under reduced pressure and the residue was purified by reverse-phase flash chromatography (eluent H_2O (0.1% TFA):MeCN, gradient elution) to obtain Boc-protected compound that was dissolved in DCM (0.5 mL), cooled down with ice bath and TFA (0.5 mL) was added dropwise. The solution was stirred at ambient temperature for 1 hour. Solvent was evaporated under reduced pressure and the residue was purified by reverse-phase preparative HPLC. The reaction provided 0.07 g of the title compound in 52% yield.

^{1}H NMR (401 MHz, $CDCl_3$) δ 7.90 (s, 1H), 7.85 – 7.76 (m, 1H), 7.09 – 7.03 (m, 1H), 7.00 – 6.89 (m, 5H), 5.54 – 5.45 (m, 1H), 4.92 (d, *J* = 16.2 Hz, 1H), 4.85 – 4.78 (m, 1H), 4.57 (dd, *J* = 16.2, 2.2 Hz, 1H), 4.40 (dd, *J* = 16.2, 2.3 Hz, 1H), 3.96 (s, 3H), 3.93 (s, 3H), 3.93 (s, 3H), 3.91 (s, 3H), 3.42 – 3.24 (m, 2H), 2.02 – 1.74 (m, 3H), 1.72 – 1.60 (m, 1H). **^{13}C NMR** (101 MHz, $CDCl_3$) δ 185.1, 167.3, 162.2 (q, *J* = 36.5 Hz), 151.2, 151.0, 149.5, 149.2, 139.5, 138.9, 128.3, 128.0, 127.3, 126.7, 124.5, 123.7, 114.1, 113.9, 111.7, 111.4, 57.8, 56.2, 56.2, 46.6, 46.2, 44.9, 29.0, 25.0. **HRMS** (ESI+): m/z calculated for $C_{28}H_{33}O_6N_2$ = 493.2333; found = 493.2332 $[M+H]^+$.

3-(3,5-Bis((*E*)-3,4-dimethoxybenzylidene)-4-oxopiperidin-1-yl)-*N*,*N*-dimethyl-3-oxopropan-1-aminium trifluoroacetate (RUN-114)

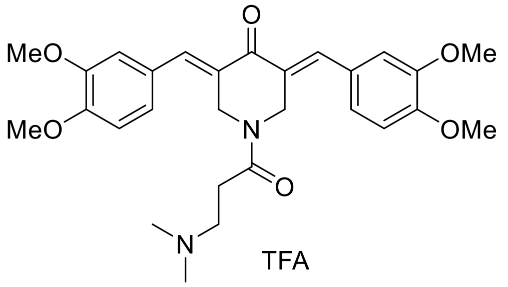

To a mixture of 3,5-bis((*E*)-3,4-dimethoxybenzylidene)-piperidin-4-one hydrochloride (**RUN-84**) (0.09 g, 0.21 mmol) and Et_3N (0.13 mL, 0.94 mmol) in DMF (3 mL) was added HBTU (0.12 g, 0.31 mmol) and 3-(dimethylamino)propanoic acid hydrochloride (0.05 g, 0.31 mmol). The reaction mixture was stirred for 2 hours at ambient temperature. Solvent was evaporated under reduced pressure and the residue was purified by reverse-phase flash chromatography (eluent H_2O (0.1% TFA):MeCN, gradient elution). The reaction provided 0.10 g of the title compound in 80% yield.

^{1}H NMR (401 MHz, $CDCl_3$) δ 7.81 – 7.74 (m, 2H), 7.11 – 7.01 (m, 2H), 7.01 – 6.95 (m, 2H), 6.92 (dd, *J* = 5.2, 3.2 Hz, 2H), 4.90 (s, 2H), 4.79 (s, 2H), 3.93 (s, 3H), 3.92 (s, 3H), 3.91 (s, 6H), 3.32 (t, *J* = 6.5 Hz, 2H), 2.88 (t, *J* = 6.6 Hz, 2H), 2.73 (s, 6H). **^{13}C NMR** (101 MHz, $CDCl_3$) δ 186.2, 167.6, 162.7 (q, *J* = 35.4 Hz), 150.9, 150.6, 149.3, 149.1, 138.5, 138.1, 129.5, 129.1, 127.6, 127.0, 124.4, 123.8, 114.2, 113.9, 111.6, 111.3, 56.1, 56.1, 53.7, 46.5, 43.6, 43.6, 28.2. **HRMS** (ESI+): m/z calculated for $C_{28}H_{35}O_6N_2$ = 495.2490; found = 495.2486 $[M+H]^+$.

4-(3,5-Bis((*E*)-3,4-dimethoxybenzylidene)-4-oxopiperidin-1-yl)-4-oxobutan-1-aminium trifluoroacetate (RUN-115)

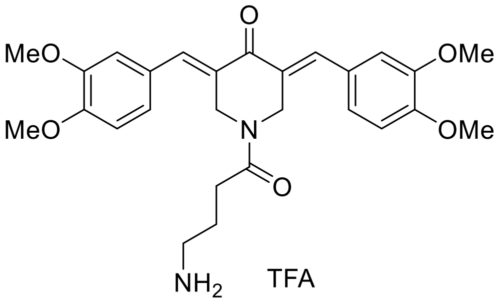

To a mixture of 3,5-bis((*E*)-3,4-dimethoxybenzylidene)-piperidin-4-one hydrochloride (**RUN-84**) (0.09 g, 0.21 mmol) and Et_3N (0.10 mL, 0.75 mmol) in DMF (3 mL) was added HBTU (0.12 g, 0.31 mmol) and 4-((*tert*-butoxycarbonyl)amino)butanoic acid (0.06 g, 0.31 mmol). The reaction mixture was stirred for 2 hours at ambient temperature. Solvent was evaporated under reduced pressure and the residue was purified by reverse-phase flash chromatography (eluent H_2O (0.1% TFA):MeCN, gradient elution) to obtain Boc-protected compound that was dissolved in DCM (0.7 mL), cooled down with ice bath and TFA (0.5 mL) was added dropwise. The solution was stirred at ambient temperature for 1 hour. Solvent was evaporated under reduced pressure and the residue was purified by reverse-phase preparative HPLC. The reaction provided 0.07 g of the title compound in 55% yield.

^{1}H NMR (401 MHz, DMSO-d_6) δ 7.69 – 7.62 (m, 5H), 7.20 – 7.11 (m, 4H), 7.11 – 7.06 (m, 2H), 4.86 (s, 2H), 4.84 (s, 2H), 3.83 (s, 9H), 3.81 (s, 3H), 2.77 – 2.64 (m, 2H), 2.38 (t, *J* = 6.9 Hz, 2H), 1.68 (p, *J* = 7.1 Hz, 2H). **^{13}C NMR** (101 MHz, DMSO-d_6) δ 185.7, 170.1, 158.1 (q, *J* = 32.5 Hz), 150.2, 150.2, 148.7, 136.4, 136.3, 130.7, 130.3, 127.2, 127.0, 124.1, 123.9, 114.3, 114.3, 111.8, 55.6, 46.2, 42.4, 38.4, 28.6, 22.4. **HRMS** (ESI+): m/z calculated for $C_{27}H_{33}O_6N_2$ = 481.2333; found = 481.2332 $[M+H]^+$.

2-(3,5-Bis((*E*)-3,4-dimethoxybenzylidene)-4-oxopiperidin-1-yl)-*N*,*N*-diethyl-2-oxoethan-1-aminium trifluoroacetate (RUN-116)

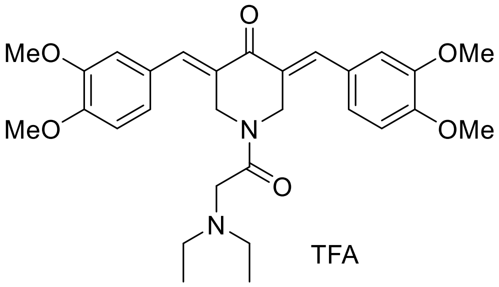

To a mixture of 3,5-bis((*E*)-3,4-dimethoxybenzylidene)-piperidin-4-one hydrochloride (**RUN-84**) (0.09 g, 0.21 mmol) and Et_3N (0.13 mL, 0.94 mmol) in DMF (3 mL) was added HBTU (0.12 g, 0.31 mmol) and diethylglycine hydrochloride (0.05 g, 0.31 mmol). The reaction mixture was stirred for 2 hours at ambient temperature. Solvent was evaporated under reduced pressure and the residue was purified by reverse-phase flash chromatography (eluent H_2O (0.1% TFA):MeCN, gradient elution). The reaction provided 0.12 g of the title compound in 89% yield.

^{1}H NMR (401 MHz, $CDCl_3$) δ 7.84 – 7.76 (m, 2H), 7.09 – 6.98 (m, 2H), 7.02 – 6.95 (m, 2H), 6.98 – 6.87 (m, 2H), 4.93 (s, 2H), 4.71 (s, 2H), 4.05 (s, 2H), 3.94 (s, 3H), 3.93 (s, 3H), 3.91 (s, 3H), 3.91 (s, 3H), 3.35 (br s, 4H), 1.24 (t, *J* = 7.3 Hz, 6H). **^{13}C NMR** (101 MHz, $CDCl_3$) δ 185.6, 162.8, 161.7 (q, *J* = 37.6 Hz), 151.2, 150.9, 149.4, 149.2, 139.2, 138.7, 128.6, 128.4, 127.4, 126.7, 124.5, 123.7, 114.2, 114.0, 111.6, 111.3, 56.2, 56.1, 56.1, 56.1, 50.4, 48.8, 46.1, 43.8, 9.7. **HRMS** (ESI+): m/z calculated for $C_{29}H_{37}O_6N_2$ = 509.2646; found = 509.2644 $[M+H]^+$.

3-(3,5-Bis((*E*)-3,4-dimethoxybenzylidene)-4-oxopiperidin-1-yl)-3-oxopropan-1-aminium trifluoroacetate (RUN-117)

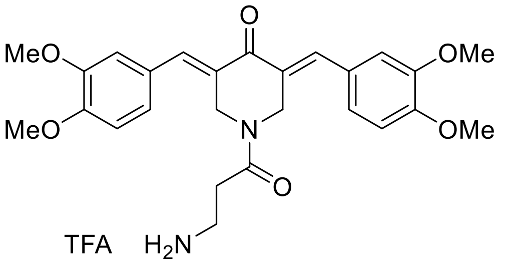

To a mixture of 3,5-bis((*E*)-3,4-dimethoxybenzylidene)-piperidin-4-one hydrochloride (**RUN-84**) (0.09 g, 0.21 mmol) and Et_3N (0.10 mL, 0.75 mmol) in DMF (3 mL) was added HBTU (0.12 g, 0.31 mmol) and 3-((*tert*-butoxycarbonyl)amino)propanoic acid (0.06 g, 0.31 mmol). The reaction mixture was stirred for 2 hours at ambient temperature. Solvent was evaporated under reduced pressure and the residue was purified by reverse-phase flash chromatography (eluent H_2O (0.1% TFA):MeCN, gradient elution) to obtain Boc-protected compound that was dissolved in DCM (1 mL), cooled down with ice bath and TFA (0.7 mL) was added dropwise. The solution was stirred at ambient temperature for 1 hour. Solvent was evaporated under reduced pressure and the residue was purified by reverse-phase preparative HPLC. The reaction provided 0.07 g of the title compound in 59% yield.

^{1}H NMR (401 MHz, DMSO-*d*$_6$) δ 7.73 – 7.66 (m, 2H), 7.57 (br s, 3H), 7.21 – 7.12 (m, 4H), 7.12 – 7.06 (m, 2H), 4.90 (s, 2H), 4.84 (s, 2H), 3.84 (s, 6H), 3.83 (s, 3H), 3.81 (s, 3H), 2.96 – 2.88 (m, 2H), 2.62 (t, *J* = 6.3 Hz, 2H). **^{13}C NMR** (101 MHz, DMSO-*d*$_6$) δ 185.4, 168.5, 150.3, 150.2, 148.7, 148.7, 136.6, 136.6, 130.4, 129.9, 127.1, 126.9, 124.2, 124.0, 114.3, 111.8, 55.6, 46.1, 42.4, 35.0, 29.4. **HRMS** (ESI+): m/z calculated for $C_{26}H_{31}O_6N_2$ = 467.2177; found = 467.2176 $[M+H]^+$.

2,4-Bis((*E*)-3,4-dimethoxybenzylidene)-8-azabicyclo[3.2.1]octan-3-one hydrochloride (RUN-78)

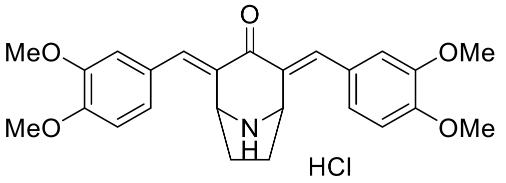

Nortropinone hydrochloride (5.00 g, 30.94 mmol) and 3,4-dimethoxybenzaldehyde (10.28 g, 61.87 mmol) was dissolved in ice-cooled glacial acetic acid (24 mL). 4 M solution of HCl in dioxane (4.8 mL, 19.20 mmol) was added dropwise and the reaction mixture was stirred at ambient temperature for 2 days. Formed suspension was filtered off and the solid was purified by recrystallization from hot methanol. The reaction provided 2.86 g of the title compound in 20% yield.

^{1}H NMR (401 MHz, DMSO-*d*$_6$) δ 9.89 (br s, 2H), 7.75 (s, 2H), 7.19 – 7.08 (m, 6H), 5.20 – 5.14 (m, 2H), 3.84 (s, 6H), 3.83 (s, 6H), 2.75 – 2.66 (m, 2H), 2.32 – 2.23 (m, 2H). **^{13}C NMR** (101 MHz, DMSO-*d*$_6$) δ 181.7, 150.7, 148.8, 137.7, 132.4, 126.0, 124.0, 114.2, 112.0, 55.7, 55.6, 55.0, 28.7. **HRMS** (ESI+): m/z calculated for $C_{25}H_{28}O_5N$ = 422.1962; found = 422.1960 $[M+H]^+$.

2,4-Bis((*E*)-3,4-dimethoxybenzylidene)-8-(2-morpholinoethyl)-8-azabicyclo[3.2.1]octan-3-one (RUN-79)

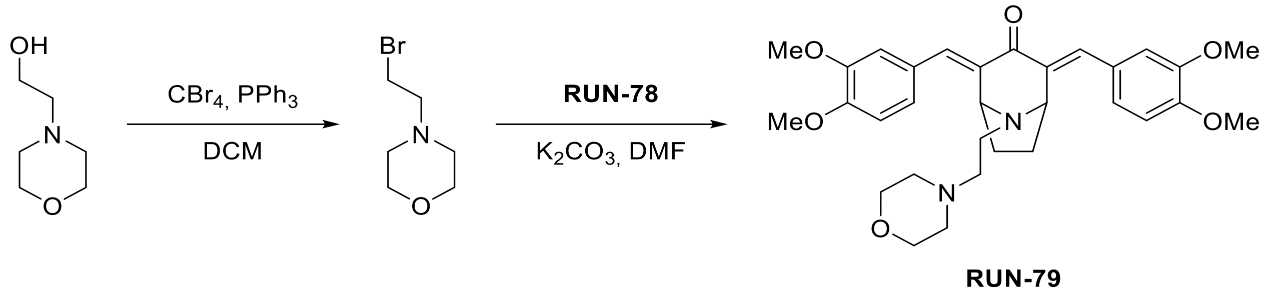

To a vigorously stirred mixture of 2-morpholinoethan-1-ol (0.50 g, 3.80 mmol) and carbon tetrabromide (1.39 g, 4.18 mmol) in DCM (5 mL) at 0 °C was added slowly triphenylphosphine (1.10 g, 4.18 mmol) and the reaction mixture was stirred overnight at ambient temperature. The mixture was concentrated under reduced pressure and the oily residue was added dropwise to a stirred cHex (15 mL). After 4 hours, formed suspension was filtered off and filtrate was concentrated under reduced pressure. The residue was purified by column chromatography (eluent cHex:EtOAc, 1:1). The reaction provided 0.45 g of 4-(2-bromoethyl)morpholine in 60% yield. Spectral data agreed with literature values.(8)
^{1}H NMR (401 MHz, $CDCl_3$) δ 3.71 (t, *J* = 4.7 Hz, 4H), 3.42 (t, *J* = 7.4 Hz, 2H), 2.78 (t, *J* = 7.2 Hz, 2H), 2.54 – 2.47 (m, 4H). **^{13}C NMR** (101 MHz, $CDCl_3$) δ 67.0, 60.3, 53.5, 28.7.

To a suspension of 2,4-bis((*E*)-3,4-dimethoxybenzylidene)-8-azabicyclo[3.2.1]octan-3-one hydrochloride (**RUN-79**) (0.23 g, 0.50 mmol) in DMF (4 mL) a 0 °C was added K_2CO_3 (0.14 g, 1.00 mmol). The reaction mixture was stirred at ambient temperature for 10 minutes and again cooled down with an ice-bath. 4-(2-bromoethyl)morpholine (0.11 g, 0.55 mmol) was added slowly to the mixture and the reaction was stirred at ambient temperature for 1.5 hour. The reaction mixture was diluted with water and extracted with EtOAc. Organic phases were combined, washed with brine, dried over $MgSO_4$ and filtrate. Solvent was evaporated and the solid residue was purified by column chromatography (eluent DCM:MeOH:NH_4OH, 97:2:1) followed by recrystallization from hot methanol. The reaction provided 0.10 g of the title compound in 37% yield.
^{1}H NMR (401 MHz, $CDCl_3$) δ 7.79 (s, 2H), 7.05 – 6.98 (m, 2H), 6.97 – 6.89 (m, 4H), 4.63 – 4.57 (m, 2H), 3.93 (s, 6H), 3.91 (s, 6H), 3.43 (t, *J* = 4.7 Hz, 4H), 2.63 (t, *J* = 6.9 Hz, 2H), 2.61 – 2.55 (m, 2H), 2.27 (t, *J* = 6.9 Hz, 2H), 2.22 (t, *J* = 4.6 Hz, 4H), 2.09 – 1.99 (m, 2H). **^{13}C NMR** (101 MHz, $CDCl_3$) δ 188.0, 150.0, 148.9, 136.9, 136.9, 128.1, 123.9, 113.6, 111.2, 66.9, 59.7, 58.7, 56.1, 56.0, 54.1, 44.9, 30.3. **HRMS** (ESI+): m/z calculated for $C_{31}H_{39}O_6N_2$ = 535.2803; found = 535.2801 $[M+H]^+$.

4-((2,4-Bis((*E*)-3,4-dimethoxybenzylidene)-3-oxo-8-azabicyclo[3.2.1]octan-8-yl)methyl)benzonitrile (RUN-80)

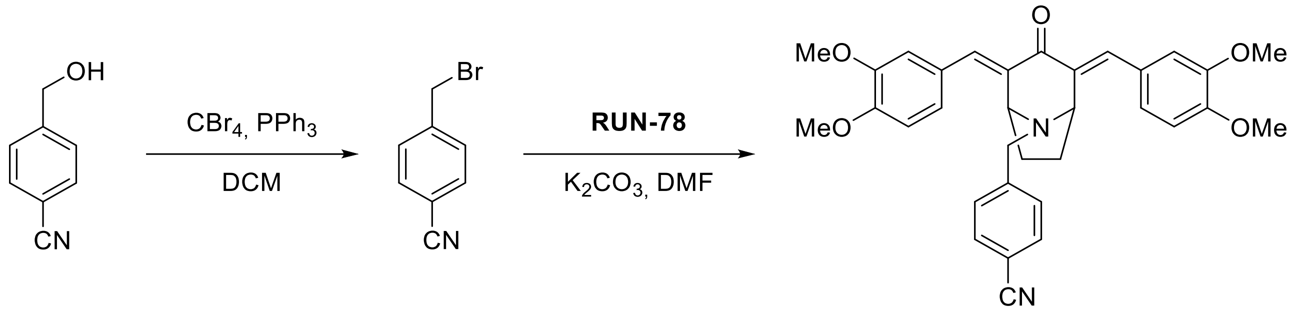

RUN-80

To a vigorously stirred mixture of 4-(hydroxymethyl)benzonitrile (0.50 g, 3.76 mmol) and carbon tetrabromide (1.37 g, 4.13 mmol) in DCM (5 mL) at 0 °C was added slowly triphenylphosphine (1.08 g, 4.13 mmol) and the reaction mixture was stirred for 6 hours at ambient temperature. The mixture was concentrated under reduced pressure and the residue was purified by column chromatography (eluent DCM). The reaction provided 0.71 g of 4-(bromomethyl)benzonitrile in 96% yield. Spectral data were in agreement with literature values.(9)

^{1}H NMR (401 MHz, $CDCl_3$) δ 7.64 (d, J = 8.2 Hz, 3H), 7.50 (d, J = 8.2 Hz, 3H), 4.48 (s, 3H). **^{13}C NMR** (101 MHz, $CDCl_3$) δ 143.0, 132.7, 129.9, 118.5, 112.4, 31.6.

To a suspension of 2,4-bis((*E*)-3,4-dimethoxybenzylidene)-8-azabicyclo[3.2.1]octan-3-one hydrochloride (**RUN-79**) (0.23 g, 0.50 mmol) in DMF (4 mL) a 0 °C was added K_2CO_3 (0.14 g, 1.00 mmol). The reaction mixture was stirred at ambient temperature for 10 minutes and again cooled down with an ice-bath. 4-(bromomethyl)benzonitrile (0.11 g, 0.55 mmol) was added slowly to the mixture and reaction was stirred at ambient temperature for 1.5 hour. The reaction mixture was diluted with water and extracted with EtOAc. Organic phases were combined, washed with brine, dried over $MgSO_4$ and filtrate. Solvent was evaporated and the solid residue was purified by column chromatography (eluent cHex:EtOAc, 5:1) followed by recrystallization from hot methanol. The reaction provided 0.21 g of the title compound in 76% yield.

^{1}H NMR (401 MHz, DMSO-d_6) δ 7.61 (s, 2H), 7.60 – 7.54 (m, 2H), 7.35 – 7.29 (m, 2H), 7.05 – 6.94 (m, 6H), 4.38 (dd, J = 4.6, 2.4 Hz, 2H), 3.79 (s, 6H), 3.78 (s, 6H), 3.70 (s, 2H), 2.59 (dt, J = 6.8, 2.8 Hz, 2H), 2.01 – 1.91 (m, 2H). **^{13}C NMR** (101 MHz, DMSO-d_6) δ 186.6, 149.7, 148.5, 144.8, 137.0, 134.9, 132.0, 129.1, 127.1, 123.4, 118.7, 113.8, 111.7, 109.5, 59.2, 55.6, 55.4, 52.2, 29.4. **HRMS** (ESI+): m/z calculated for $C_{33}H_{33}O_5N_2$ = 537.2384; found = 537.2383 $[M+H]^+$.

***Tert*-butyl (3,5-bis((*E*)-3,4-dimethoxybenzylidene)-4-oxocyclohexyl)carbamate (2)**

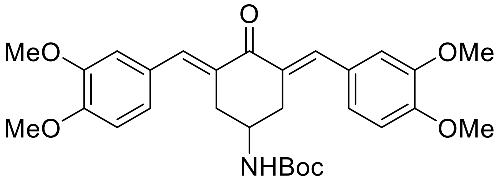

Tert-butyl (4-oxocyclohexyl)carbamate (2.00 g, 9.38 mmol) was dissolved in EtOH (10 mL) and 20% aq. solution of NaOH (9.3 mL) was added dropwise. Solution was stirred for 5 minutes and 3,4-dimethoxybenzaldehyde (3.90 g, 23.44 mmol) was added. Reaction mixture was stirred at ambient temperature overnight. Formed suspension was filtered off, the filter cake was washed

with water and cold EtOH and dried. The reaction provided 3.45 g of the title compound in 75% yield.

^{1}H NMR (401 MHz, $CDCl_3$) δ 7.91 – 7.85 (m, 2H), 7.11 (dd, *J* = 8.5, 2.0 Hz, 2H), 7.02 (d, *J* = 2.1 Hz, 2H), 6.93 (d, *J* = 8.4 Hz, 2H), 4.66 (br s, 1H), 4.09 (br s, 1H), 3.95 (s, 6H), 3.93 (s, 6H), 3.20 (dt, *J* = 15.2, 3.2 Hz, 2H), 3.12 – 3.06 (m, 2H), 1.37 (s, 9H). **^{13}C NMR** (101 MHz, $CDCl_3$) δ 188.7, 155.1, 150.1, 148.9, 139.8, 130.7, 128.5, 124.1, 113.9, 111.1, 79.8, 56.1, 56.1, 45.3, 34.2, 28.4.**HRMS** (ESI+): *m/z* calculated for $C_{29}H_{35}O_7NNa$ = 532.2306; found = 523.2302 $[M+Na]^+$.

3,5-Bis((*E*)-3,4-dimethoxybenzylidene)-4-oxocyclohexan-1-aminium trifluoroacetate (RUN-81)

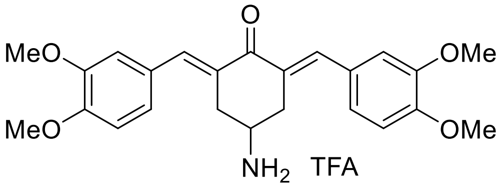

Tert-butyl (3,5-bis((*E*)-3,4-dimethoxybenzylidene)-4-oxocyclohexyl)carbamate (1.00 g, 1.96 mmol) was dissolved in DCM (5 mL), cooled down with ice bath and TFA (5 mL) was added dropwise. The solution was stirred at ambient temperature for 1.5 hour. Solvent was evaporated under reduced pressure and the residue was purified by reverse-phase flash chromatography (eluent H_2O (0.1% TFA):MeCN, gradient elution). The reaction provided 0.87 g of the title compound in 84% yield.

^{1}H NMR (401 MHz, DMSO-*d*$_6$) δ 8.12 (br s, 3H), 7.74 (d, *J* = 2.2 Hz, 2H), 7.18 – 7.12 (m, 4H), 7.09 (d, *J* = 8.3 Hz, 2H), 3.83 (s, 6H), 3.82 (s, 6H), 3.55 – 3.41 (m, 1H), 3.37 (dd, *J* = 15.7, 4.0 Hz, 2H), 3.04 – 2.93 (m, 2H). **^{13}C NMR** (101 MHz, DMSO-*d*$_6$) δ 186.0, 157.9 (q, *J* = 30.9 Hz), 150.0, 148.6, 138.4, 129.7, 127.5, 123.8, 114.2, 111.7, 55.7, 55.6, 45.9, 31.7. **HRMS** (ESI+): m/z calculated for $C_{24}H_{28}O_5N$ = 410.1962; found = 410.1961 $[M+H]^+$.

2-((3,5-Bis((*E*)-3,4-dimethoxybenzylidene)-4-oxocyclohexyl)amino)-*N,N*-dimethyl-2-oxoethan-1-aminium trifluoroacetate (RUN-86)

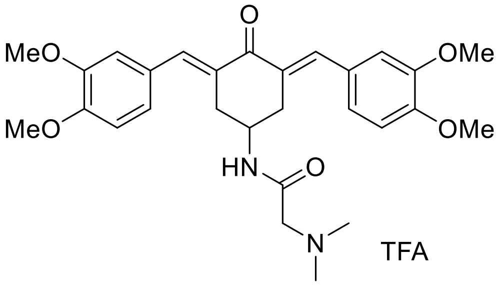

To a mixture of 3,5-bis((*E*)-3,4-dimethoxybenzylidene)-4-oxocyclohexan-1-aminium trifluoroacetate (**RUN-81**) (0.10 g, 0.19 mmol) and Et_3N (0.10 mL, 0.72 mmol) in DMF (3 mL) was added HBTU (0.14 g, 0.35 mmol) and *N,N*-dimethylglycine (0.04 g, 0.36 mmol). The reaction mixture was stirred overnight at ambient temperature. Solvent was evaporated under reduced pressure and the residue was purified by reverse-phase flash chromatography (eluent H_2O (0.1% TFA):MeCN, gradient elution). The reaction provided 0.05 g of the title compound in 46% yield.

^{1}H NMR (401 MHz, $CDCl_3$) δ 8.47 (d, *J* = 7.2 Hz, 1H), 7.82 (s, 2H), 7.05 (dd, *J* = 8.4, 2.0 Hz, 2H), 6.95 (d, *J* = 2.0 Hz, 2H), 6.88 (d, *J* = 8.4 Hz, 2H), 4.23 – 4.10 (m, 1H), 3.90 (s, 6H), 3.88 (s, 6H), 3.71 (s, 2H), 3.23 (dd, *J* = 15.9, 4.0 Hz, 2H), 3.00 – 2.85 (m, 2H), 2.82 (s, 6H). **^{13}C NMR** (101 MHz, $CDCl_3$) δ 187.9, 162.8, 162.6 (q, *J* = 37.1 Hz), 150.2, 148.9, 139.7, 130.4, 128.4, 124.1, 113.9, 111.1, 59.1, 56.1, 56.1, 45.8, 43.5, 33.7. **HRMS** (ESI+): m/z calculated for $C_{28}H_{35}O_6N_2$ =495.2490; found = 495.2487 $[M+H]^+$.

3-((3,5-Bis((*E*)-3,4-dimethoxybenzylidene)-4-oxocyclohexyl)carbamoyl)quinuclidin-1-ium trifluoroacetate (RUN-87)

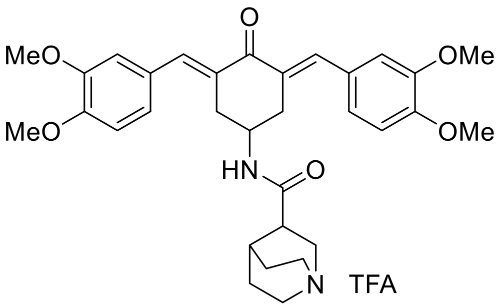

To a mixture of 3,5-bis((*E*)-3,4-dimethoxybenzylidene)-4-oxocyclohexan-1-aminium trifluoroacetate (**RUN-81**) (0.10 g, 0.19 mmol) and Et_3N (0.11 mL, 0.79 mmol) in DMF (3 mL) was added HBTU (0.11 g, 0.29 mmol) and 3-carboxyquinuclidin-1-ium hydrochloride (0.06 g, 0.29 mmol). The reaction mixture was stirred overnight at ambient temperature. Solvent was evaporated under reduced pressure and the residue was purified by reverse-phase flash chromatography (eluent H_2O (0.1% TFA):MeCN, gradient elution). The reaction provided 0.07 g of the title compound in 56% yield.

^{1}H NMR (401 MHz, $CDCl_3$) δ 7.87 – 7.78 (m, 2H), 7.06 (ddd, *J* = 8.6, 3.9, 1.9 Hz, 2H), 6.95 (dd, *J* = 3.8, 2.0 Hz, 2H), 6.89 (d, *J* = 8.5 Hz, 2H), 6.53 (d, *J* = 7.2 Hz, 1H), 4.40 – 4.31 (m, 1H), 3.91 (s, 3H), 3.90 (s, 3H), 3.89 (s, 3H), 3.88 (s, 3H), 3.69 – 3.59 (m, 1H), 3.30 – 3.16 (m, 6H), 3.16 – 3.03 (m, 3H), 2.83 – 2.73 (m, 1H), 2.16 – 2.08 (m, 1H), 1.95 – 1.72 (m, 3H), 1.59 – 1.43 (m, 1H). **^{13}C NMR** (101 MHz, $CDCl_3$) δ 188.5, 170.6, 162.0 (q, *J* = 35.7 Hz), 150.4, 150.3, 148.9, 140.5, 140.1, 130.3, 129.8, 128.2, 128.2, 124.3, 124.2, 114.0, 113.8, 111.2, 111.2, 56.1, 56.1, 56.1, 47.7, 46.2, 45.9, 44.8, 40.0, 33.5, 33.2, 24.8, 23.6, 19.3. **HRMS** (ESI+): m/z calculated for $C_{32}H_{39}O_6N_2$ = 547.2803; found = 547.2801 $[M+H]^+$.

***N*-(3,5-Bis((*E*)-3,4-dimethoxybenzylidene)-4-oxocyclohexyl)-2-(2-(2-methoxyethoxy)ethoxy)acetamide (RUN-88)**

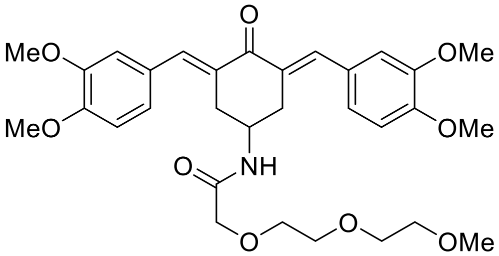

To a mixture of 3,5-bis((*E*)-3,4-dimethoxybenzylidene)-4-oxocyclohexan-1-aminium trifluoroacetate (**RUN-81**) (0.10 g, 0.19 mmol) and Et_3N (0.1 mL, 0.72 mmol) in DMF (3 mL) was added HBTU (0.11 g, 0.29 mmol) and 2-(2-(2-methoxyethoxy)ethoxy)acetic acid (0.05 g, 0.29 mmol). The reaction mixture was stirred overnight at ambient temperature. Solvent was evaporated under reduced pressure and the residue was purified by reverse-phase flash chromatography (eluent H_2O:MeCN, gradient elution). The reaction provided 0.08 g of the title compound in 74% yield.

^{1}H NMR (401 MHz, DMSO-d_6) δ 7.79 (d, *J* = 7.5 Hz, 1H), 7.67 (s, 2H), 7.16 – 7.07 (m, 4H), 7.05 (d, *J* = 8.2 Hz, 2H), 4.04 – 3.92 (m, 1H), 3.87 (s, 2H), 3.81 (s, 6H), 3.80 (s, 6H), 3.59 – 3.49 (m, 6H), 3.46 – 3.39 (m, 2H), 3.21 (s, 3H), 3.19 – 3.10 (m, 2H), 3.03 – 2.91 (m, 2H). **^{13}C NMR** (101 MHz, DMSO-d_6) δ 187.2, 168.9, 149.7, 148.5, 137.6, 131.6, 127.8, 123.6, 114.3, 111.6, 71.3, 70.1, 69.8, 69.6, 69.5, 58.0, 55.5, 44.2, 33.4. **HRMS** (ESI+): m/z calculated for $C_{31}H_{39}O_9NNa$ = 592.2517; found = 592.2513 $[M+Na]^+$.

N-(3,5-Bis((*E*)-3,4-dimethoxybenzylidene)-4-oxocyclohexyl)cyclopentanecarboxamide (RUN-89)

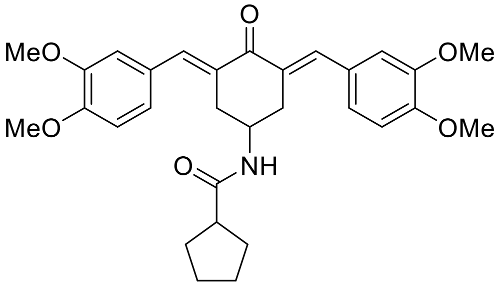

To a mixture of 3,5-bis((*E*)-3,4-dimethoxybenzylidene)-4-oxocyclohexan-1-aminium trifluoroacetate (**RUN-81**) (0.10 g, 0.19 mmol) and Et_3N (0.1 mL, 0.72 mmol) in DMF (3 mL) was added HBTU (0.11 g, 0.29 mmol) and cyclopentanecarboxylic acid (0.03 g, 0.29 mmol). The reaction mixture was stirred overnight at ambient temperature. Solvent was evaporated under reduced pressure and the residue was purified by reverse-phase flash chromatography (eluent H_2O:MeCN, gradient elution). The reaction provided 0.09 g of the title compound in 88% yield. **^{1}H NMR** (401 MHz, DMSO-d_6) δ 7.98 (d, *J* = 6.7 Hz, 1H), 7.70 – 7.62 (m, 2H), 7.18 – 7.08 (m, 4H), 7.04 (d, *J* = 8.3 Hz, 2H), 3.92 – 3.84 (m, 1H), 3.81 (s, 6H), 3.80 (s, 6H), 3.13 (dd, *J* = 16.0, 4.0 Hz, 2H), 2.93 – 2.82 (m, 2H), 2.61 – 2.50 (m, 1H), 1.74 – 1.60 (m, 2H), 1.63 – 1.50 (m, 4H), 1.52 – 1.40 (m, 2H). **^{13}C NMR** (101 MHz, DMSO-d_6) δ 187.5, 175.2, 149.7, 148.5, 137.5, 131.8, 127.9, 123.6, 114.1, 111.6, 55.6, 44.4, 44.0, 33.4, 30.0, 25.6. **HRMS** (ESI+): m/z calculated for $C_{30}H_{35}O_6NNa$ = 528.2357; found = 528.2353 $[M+Na]^+$.

1-(2-((3,5-Bis((*E*)-3,4-dimethoxybenzylidene)-4-oxocyclohexyl)amino)-2-oxoethyl)-4-methylpiperazine-1,4-diium di(trifluoroacetate) (RUN-90)

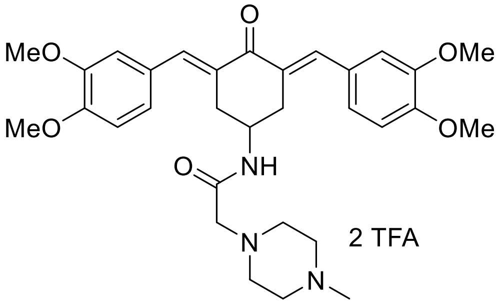

To a mixture of 3,5-bis((*E*)-3,4-dimethoxybenzylidene)-4-oxocyclohexan-1-aminium trifluoroacetate (**RUN-81**) (0.10 g, 0.19 mmol) and Et_3N (0.1 mL, 0.72 mmol) in DMF (3 mL) was added HBTU (0.11 g, 0.29 mmol) and 2-(4-methylpiperazin-1-yl)acetic acid (0.05 g, 0.29 mmol). The reaction mixture was stirred overnight at ambient temperature. Solvent was evaporated under reduced pressure and the residue was purified by reverse-phase flash chromatography (eluent H_2O (0.1% TFA):MeCN, gradient elution). The reaction provided 0.10 g of the title compound in 67% yield.

^{1}H NMR (401 MHz, $CDCl_3$) δ 7.88 (br s, 2H), 7.11 (dd, *J* = 8.4, 2.0 Hz, 2H), 7.03 – 6.97 (m, 3H), 6.93 (d, *J* = 8.4 Hz, 2H), 4.55 – 4.47 (m, 1H), 3.93 (s, 6H), 3.92 (s, 6H), 3.29 (dd, *J* = 16.2, 4.7 Hz, 2H), 3.16 – 3.07 (m, 2H), 3.07 (s, 2H), 2.82 (s, 3H), 2.79 (br s, 4H). **^{13}C NMR** (101 MHz, $CDCl_3$) δ 188.9, 167.8, 161.9 (q, *J* = 37.9 Hz), 150.7, 149.1, 140.6, 129.6, 128.0, 124.5, 113.9, 111.3, 60.1, 56.2, 56.2, 53.3, 49.8, 43.5, 43.3, 33.2. **HRMS** (ESI+): m/z calculated for $C_{31}H_{40}O_6N_3$ =550.2912; found = 550.2910 $[M+H]^+$.

4-((3,5-Bis((*E*)-3,4-dimethoxybenzylidene)-4-oxocyclohexyl)amino)-*N,N*-dimethyl-4-oxobutan-1-aminium trifluoroacetate (RUN-91)

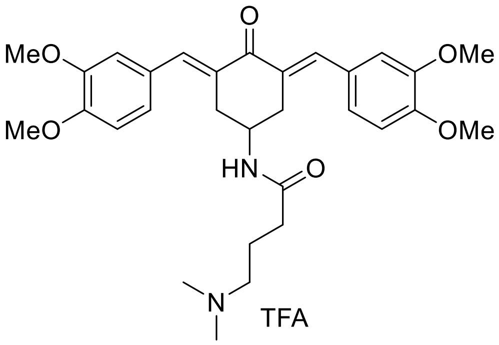

To a mixture of 3,5-bis((*E*)-3,4-dimethoxybenzylidene)-4-oxocyclohexan-1-aminium trifluoroacetate (**RUN-81**) (0.10 g, 0.19 mmol) and Et_3N (0.11 mL, 0.79 mmol) in DMF (3 mL) was added HBTU (0.11 g, 0.29 mmol) and 3-carboxy-*N,N*-dimethylpropan-1-aminium hydrochloride (0.05 g, 0.29 mmol). The reaction mixture was stirred overnight at ambient temperature. Solvent was evaporated under reduced pressure and the residue was purified by reverse-phase flash chromatography (eluent H_2O (0.1% TFA):MeCN, gradient elution). The reaction provided 0.07 g of the title compound in 53% yield.

^{1}H NMR (401 MHz, $CDCl_3$) δ 7.87 – 7.81 (m, 2H), 7.09 (dd, *J* = 8.4, 2.0 Hz, 2H), 6.98 (d, *J* = 2.0 Hz, 2H), 6.94 – 6.87 (m, 3H), 4.30 – 4.18 (m, 1H), 3.91 (s, 6H), 3.90 (s, 6H), 3.26 – 3.16 (m, 2H), 3.07 – 2.93 (m, 4H), 2.76 (s, 6H), 2.38 – 2.31 (m, 2H), 2.06 – 1.95 (m, 2H). **^{13}C NMR** (101 MHz, $CDCl_3$) δ 188.3, 170.9, 162.3 (q, *J* = 35.3 Hz), 150.1, 148.9, 139.7, 130.7, 128.5, 124.1, 114.0, 111.1, 57.3, 56.1, 45.1, 43.0, 34.0, 32.3, 20.5. **HRMS** (ESI+): m/z calculated for $C_{30}H_{39}O_6N_2$ =523.2803; found = 523.2800 $[M+H]^+$.

2-((3,5-Bis((*E*)-3,4-dimethoxybenzylidene)-4-oxocyclohexyl)amino)-2-oxoethan-1-aminium trifluoroacetate (RUN-92)

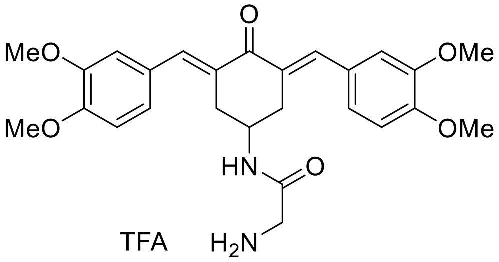

To a mixture of 3,5-bis((*E*)-3,4-dimethoxybenzylidene)-4-oxocyclohexan-1-aminium trifluoroacetate (**RUN-81**) (0.10 g, 0.19 mmol) and Et_3N (0.1 mL, 0.72 mmol) in DMF (3 mL) was added HBTU (0.11 g, 0.29 mmol) and (*tert*-butoxycarbonyl)glycine (0.05 g, 0.29 mmol). The reaction mixture was stirred overnight at ambient temperature. Solvent was evaporated under reduced pressure and the residue was purified by reverse-phase flash chromatography (eluent H_2O (0.1% TFA):MeCN, gradient elution) to obtain Boc-protected compound that was dissolved in DCM (0.5 mL), cooled down with ice bath and TFA (0.5 mL) was added dropwise. The solution was stirred at ambient temperature for 1 hour. Solvent was evaporated under reduced pressure and the residue was purified by reverse-phase flash chromatography (eluent H_2O (0.1% TFA):MeCN, gradient elution). The reaction provided 0.08 g of the title compound in 73% yield.

^{1}H NMR (401 MHz, DMSO-*d*$_6$) δ 8.60 (d, *J* = 6.7 Hz, 1H), 7.95 (br s, 3H), 7.71 (s, 2H), 7.17 – 7.09 (m, 4H), 7.04 (d, J = 8.2 Hz, 2H), 4.05 – 3.93 (m, 1H), 3.81 (s, 6H), 3.80 (s, 6H), 3.52 (br s, 2H), 3.20 (dd, J = 16.4, 4.1 Hz, 2H), 3.01 – 2.86 (m, 2H). **^{13}C NMR** (101 MHz, DMSO-*d*$_6$) δ 186.9, 165.7, 157.89 (q, *J* = 30.8 Hz), 149.8, 148.5, 137.9, 131.1, 127.8, 123.6, 114.3, 111.6, 55.6, 40.0, 44.8, 33.2. **HRMS** (ESI+): m/z calculated for $C_{26}H_{31}O_6N_2$ = 467.2177; found = 467.2175 $[M+H]^+$.

2-((3,5-Bis((*E*)-3,4-dimethoxybenzylidene)-4-oxocyclohexyl)carbamoyl)-3-methylpyridin-1-ium trifluoroacetate (RUN-93)

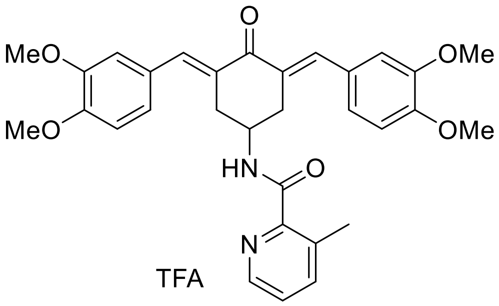

To a mixture of 3,5-bis((*E*)-3,4-dimethoxybenzylidene)-4-oxocyclohexan-1-aminium trifluoroacetate (**RUN-81**) (0.10 g, 0.19 mmol) and Et_3N (0.1 mL, 0.72 mmol) in DMF (3 mL) was added HBTU (0.11 g, 0.29 mmol) and 3-methylpicolinic acid (0.04 g, 0.29 mmol). The reaction mixture was stirred overnight at ambient temperature. Solvent was evaporated under reduced pressure and the residue was purified by reverse-phase flash chromatography (eluent H_2O (0.1% TFA):MeCN, gradient elution). The reaction provided 0.10 g of the title compound in 80% yield.

^{1}H NMR (401 MHz, $CDCl_3$) δ 8.44 – 8.36 (m, 2H), 7.90 (s, 2H), 7.75 – 7.68 (m, 1H), 7.42 (dd, *J* = 7.8, 4.9 Hz, 1H), 7.11 (dd, *J* = 8.4, 2.0 Hz, 2H), 7.01 (d, *J* = 2.0 Hz, 2H), 6.90 (d, *J* = 8.4 Hz, 2H), 4.54 – 4.41 (m, 1H), 3.91 (s, 6H), 3.90 (s, 6H), 3.40 – 3.30 (m, 2H), 3.17 – 3.06 (m, 2H), 2.63 (s, 3H). **^{13}C NMR** (101 MHz, $CDCl_3$) δ 188.9, 164.6, 150.1, 148.9, 146.7, 144.2, 142.5, 140.1, 136.4, 130.8, 128.5, 126.2, 124.1, 114.0, 111.1, 56.1, 45.0, 34.0, 20.2. **HRMS** (ESI+): m/z calculated for $C_{31}H_{32}O_6N_2Na$ = 551.2153; found = 551.2151 $[M+Na]^+$.

2-((3,5-Bis((*E*)-3,4-dimethoxybenzylidene)-4-oxocyclohexyl)carbamoyl)pyridin-1-ium trifluoroacetate (RUN-94)

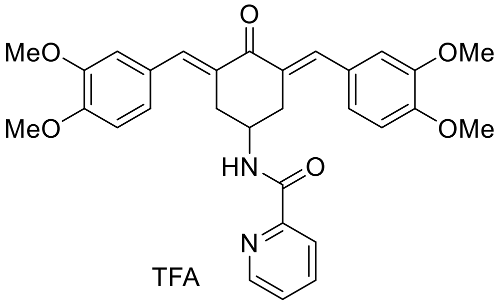

To a mixture of 3,5-bis((*E*)-3,4-dimethoxybenzylidene)-4-oxocyclohexan-1-aminium trifluoroacetate (**RUN-81**) (0.10 g, 0.19 mmol) and Et_3N (0.1 mL, 0.72 mmol) in DMF (3 mL) was added HBTU (0.11 g, 0.29 mmol) and picolinic acid (0.04 g, 0.29 mmol). The reaction mixture was stirred at ambient temperature for 3 hours. Solvent was evaporated under reduced pressure and the residue was purified by reverse-phase flash chromatography (eluent H_2O (0.1% TFA):MeCN, gradient elution). The reaction provided 0.10 g of the title compound in 82% yield.

^{1}H NMR (401 MHz, $CDCl_3$) δ 8.54 (ddd, *J* = 4.8, 1.7, 0.9 Hz, 1H), 8.33 (d, *J* = 8.1 Hz, 1H), 8.14 (dt, *J* = 7.9, 1.1 Hz, 1H), 7.93 (br s, 2H), 7.86 (td, *J* = 7.7, 1.7 Hz, 1H), 7.46 (ddd, *J* = 7.6, 4.8, 1.2 Hz, 1H), 7.12 (dd, *J* = 8.4, 1.8 Hz, 2H), 7.01 (d, *J* = 2.0 Hz, 2H), 6.90 (d, *J* = 8.4 Hz, 2H), 4.62 – 4.49 (m, 1H), 3.91 (s, 6H), 3.90 (s, 6H), 3.40 – 3.30 (m, 2H), 3.22 – 3.11 (m, 2H). **^{13}C NMR** (101 MHz, $CDCl_3$) δ 188.8, 164.1, 150.2, 149.2, 148.9, 148.0, 140.4, 138.0, 130.5, 128.5, 126.7, 124.1, 122.7, 114.1, 111.1, 56.1, 56.1, 44.8, 34.0. **HRMS** (ESI+): m/z calculated for $C_{30}H_{30}O_6N_2Na$ = 537.1996; found = 537.1994 $[M+Na]^+$.

4-(2-((3,5-Bis((*E*)-3,4-dimethoxybenzylidene)-4-oxocyclohexyl)amino)-2-oxoethyl)-1*H*-imidazol-3-ium trifluoroacetate (RUN-95)

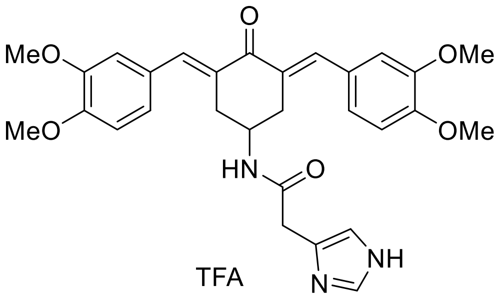

To a mixture of 3,5-bis((*E*)-3,4-dimethoxybenzylidene)-4-oxocyclohexan-1-aminium trifluoroacetate (**RUN-81**) (0.10 g, 0.19 mmol) and Et_3N (0.12 mL, 0.86 mmol) in DMF (3 mL) was added HBTU (0.11 g, 0.29 mmol) and 2-(1*H*-imidazol-4-yl)acetic acid (0.05 g, 0.29 mmol). The reaction mixture was stirred at ambient temperature for 1 hour. Solvent was evaporated under reduced pressure and the residue was purified by reverse-phase flash chromatography (eluent H_2O (0.1% TFA):MeCN, gradient elution). The reaction provided 0.08 g of the title compound in 66% yield.

^{1}H NMR (401 MHz, $CDCl_3$) δ 8.27 – 8.19 (m, 1H), 7.79 – 7.73 (m, 3H), 7.05 – 6.97 (m, 3H), 6.91 (d, *J* = 1.9 Hz, 2H), 6.83 (d, *J* = 8.5 Hz, 2H), 4.20 – 4.11 (m, 1H), 3.86 (s, 6H), 3.83 (s, 6H), 3.58 (s, 2H), 3.21 – 3.13 (m, 2H), 3.06 – 2.96 (m, 2H). **^{13}C NMR** (101 MHz, $CDCl_3$) δ 188.4, 167.8, 162.2 (q, *J* = 36.0 Hz), 150.2, 148.8, 139.8, 130.4, 128.3, 128.1, 124.3, 114.0, 111.2, 56.0, 45.5, 33.5, 31.9. **HRMS** (ESI+): m/z calculated for $C_{29}H_{32}O_6N_3$ = 518.2286; found = 518.2284 $[M+H]^+$.

(*S*)-2-((3,5-Bis((*E*)-3,4-dimethoxybenzylidene)-4-oxocyclohexyl)carbamoyl)pyrrolidin-1-ium trifluoroacetate (RUN-96)

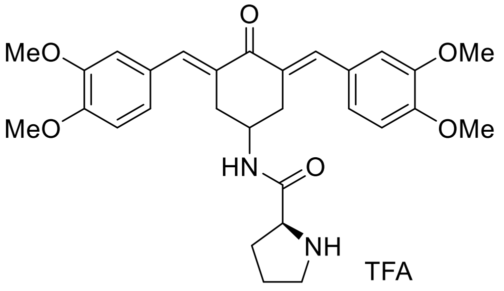

To a mixture of 3,5-bis((*E*)-3,4-dimethoxybenzylidene)-4-oxocyclohexan-1-aminium trifluoroacetate (**RUN-81**) (0.10 g, 0.19 mmol) and Et_3N (0.1 mL, 0.72 mmol) in DMF (3 mL) was added HBTU (0.11 g, 0.29 mmol) and (*tert*-butoxycarbonyl)-*L*-proline (0.06 g, 0.29 mmol). The reaction mixture was stirred at ambient temperature for 1 hour. Solvent was evaporated under reduced pressure and the residue was purified by reverse-phase flash chromatography (eluent H_2O (0.1% TFA):MeCN, gradient elution) to obtain Boc-protected compound that was dissolved in DCM (0.5 mL), cooled down with ice bath and TFA (0.5 mL) was added dropwise. The solution was stirred at ambient temperature for 1.5 hour. Solvent was evaporated under reduced pressure and the residue was purified by reverse-phase flash chromatography (eluent H_2O (0.1% TFA):MeCN, gradient elution). The reaction provided 0.07 g of the title compound in 58% yield.

^{1}H NMR (401 MHz, $CDCl_3$) δ 8.22 (d, *J* = 6.7 Hz, 1H), 7.84 – 7.80 (m, 1H), 7.80 – 7.78 (m, 1H), 7.08 – 7.00 (m, 2H), 6.98 – 6.83 (m, 4H), 4.51 (br s, 1H), 4.15 – 4.07 (m, 1H), 3.91 (s, 3H), 3.89 (s, 3H), 3.87 (s, 3H), 3.86 (s, 3H), 3.30 – 3.23 (m, 2H), 3.19 – 2.90 (m, 4H), 2.35 – 2.28 (m, 1H), 1.97 – 1.75 (m, 3H). **^{13}C NMR** (101 MHz, $CDCl_3$) δ 188.1, 168.5, 150.1, 148.9, 148.8, 140.0, 139.8, 130.4, 130.0, 128.3, 128.3, 124.1, 124.1, 114.3, 113.9, 111.1, 111.1, 59.4, 56.1, 46.7, 46.0, 33.5, 33.3, 30.6, 24.8. **HRMS** (ESI+): m/z calculated for $C_{29}H_{32}O_6N_3$ = 507.2490; found = 507.2488 $[M+H]^+$.

(*S*)-*N*-(3,5-Bis((*E*)-3,4-dimethoxybenzylidene)-4-oxocyclohexyl)-5-oxopyrrolidine-2-carboxamide (RUN-97)

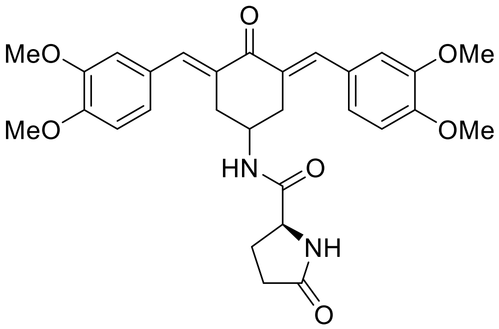

To a mixture of 3,5-bis((*E*)-3,4-dimethoxybenzylidene)-4-oxocyclohexan-1-aminium trifluoroacetate (**RUN-81**) (0.10 g, 0.19 mmol) and Et_3N (0.1 mL, 0.72 mmol) in DMF (3 mL) was added HBTU (0.11 g, 0.29 mmol) and (*S*)-5-oxopyrrolidine-2-carboxylic acid (0.04 g, 0.29 mmol). The reaction mixture was stirred at ambient temperature for 2.5 hours. Solvent was evaporated under reduced pressure and the residue was purified by reverse-phase flash chromatography (eluent H_2O (0.1% TFA):MeCN, gradient elution). The reaction provided 0.08 g of the title compound in 85% yield.

^{1}H NMR (401 MHz, $CDCl_3$) δ 7.85 – 7.80 (m, 1H), 7.80 – 7.77 (m, 1H), 7.10 – 7.00 (m, 2H), 7.00 – 6.88 (m, 4H), 6.90 – 6.81 (m, 2H), 4.47 – 4.38 (m, 1H), 4.14 – 4.06 (m, 1H), 3.89 (s, 6H), 3.88 (s, 6H), 3.28 – 3.17 (m, 2H), 3.20 – 3.06 (m, 2H), 2.47 – 2.32 (m, 1H), 2.21 (dd, *J* = 9.0, 7.0 Hz, 2H), 2.09 – 1.97 (m, 1H). **^{13}C NMR** (101 MHz, $CDCl_3$) δ 188.7, 180.1, 171.8, 150.3, 150.3, 149.0, 140.3, 140.1, 130.2, 130.2, 128.3, 128.2, 124.2, 124.0, 114.0, 113.9, 111.1, 57.4, 56.1, 56.1, 56.1, 44.5, 33.5, 33.1, 29.3, 25.8. **HRMS** (ESI+): m/z calculated for $C_{29}H_{32}O_7N_2Na$ = 543.2102; found = 543.2100 $[M+Na]^+$.

4-(2-((3,5-Bis((*E*)-3,4-dimethoxybenzylidene)-4-oxocyclohexyl)amino)-2-oxoethyl)morpholin-4-ium trifluoroacetate (RUN-98)

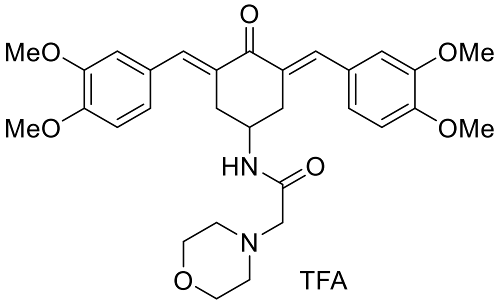

To a mixture of 3,5-bis((*E*)-3,4-dimethoxybenzylidene)-4-oxocyclohexan-1-aminium trifluoroacetate (**RUN-81**) (0.10 g, 0.19 mmol) and Et_3N (0.12 mL, 0.86 mmol) in DMF (3 mL) was added HBTU (0.11 g, 0.29 mmol) and 2-morpholinoacetic acid (0.05 g, 0.29 mmol). The reaction mixture was stirred at ambient temperature for 2 hours. Solvent was evaporated under reduced pressure and the residue was purified by reverse-phase flash chromatography (eluent H_2O (0.1% TFA):MeCN, gradient elution). The reaction provided 0.10 g of the title compound in 76% yield.

^{1}H NMR (401 MHz, $CDCl_3$) δ 8.30 (d, *J* = 7.2 Hz, 1H), 7.84 (s, 2H), 7.07 (dd, *J* = 8.4, 2.0 Hz, 2H), 6.97 (d, *J* = 2.1 Hz, 2H), 6.90 (d, *J* = 8.4 Hz, 2H), 4.25 – 4.14 (m, 1H), 3.91 (s, 6H), 3.90 (s, 6H), 3.90 – 3.86 (m, 4H), 3.57 (s, 2H), 3.22 (dd, *J* = 15.8, 3.9 Hz, 2H), 3.15 – 3.08 (m, 4H), 3.04 – 2.92 (m, 2H). **^{13}C NMR** (101 MHz, $CDCl_3$) δ 187.9, 163.0 (q, *J* = 36.9 Hz), 162.7, 150.2, 148.9, 139.8, 130.3, 128.3, 124.0, 113.9, 111.1, 64.1, 59.4, 56.1, 52.6, 45.7, 33.7. **HRMS** (ESI+): m/z calculated for $C_{30}H_{37}O_7N_2$ = 537.2595; found = 537.2593 $[M+H]^+$.

1-(2-((3,5-Bis((*E*)-3,4-dimethoxybenzylidene)-4-oxocyclohexyl)amino)-2-oxoethyl)-1*H*-imidazol-1-ium trifluoroacetate (RUN-99)

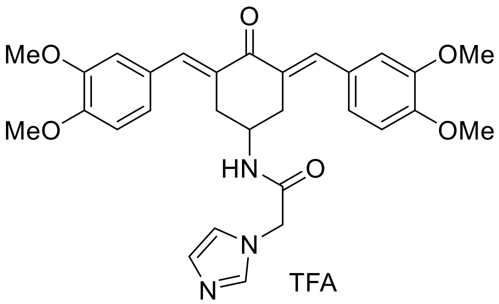

To a mixture of 3,5-bis((*E*)-3,4-dimethoxybenzylidene)-4-oxocyclohexan-1-aminium trifluoroacetate (**RUN-81**) (0.10 g, 0.19 mmol) and Et_3N (0.1 mL, 0.72 mmol) in DMF (3 mL) was added HBTU (0.11 g, 0.29 mmol) and 2-(1*H*-imidazol-1-yl)acetic acid (0.04 g, 0.29 mmol). The reaction mixture was stirred at ambient temperature for 2 hours. Solvent was evaporated under reduced pressure and the residue was purified by reverse-phase flash chromatography (eluent H_2O (0.1% TFA):MeCN, gradient elution). The reaction provided 0.10 g of the title compound in 80% yield.

^{1}H NMR (401 MHz, $CDCl_3$) δ 9.16 (s, 1H), 8.38 (d, *J* = 7.0 Hz, 1H), 7.79 – 7.73 (m, 2H), 7.38 – 7.32 (m, 1H), 7.21 – 7.15 (m, 1H), 7.00 (dd, *J* = 8.4, 2.0 Hz, 2H), 6.92 (d, *J* = 1.9 Hz, 2H), 6.83 (d, *J* = 8.4 Hz, 2H), 4.95 (s, 2H), 4.26 – 4.19 (m, 1H), 3.88 (s, 6H), 3.85 (s, 6H), 3.24 – 3.14 (m, 2H), 3.04 (dd, *J* = 15.5, 8.3 Hz, 2H). **^{13}C NMR** (101 MHz, $CDCl_3$) δ 188.3, 164.3, 162.1 (q, *J* = 36.0 Hz), 150.2, 148.8, 139.9, 136.1, 130.3, 128.3, 124.2, 122.5, 119.8, 113.9, 111.1, 56.0, 51.3, 45.5, 33.4. **HRMS** (ESI+): m/z calculated for $C_{29}H_{32}O_6N_3$ = 518.2286; found = 518.2284 $[M+H]^+$.

4-((3,5-Bis((*E*)-3,4-dimethoxybenzylidene)-4-oxocyclohexyl)amino)-4-oxobutan-1-aminium trifluoroacetate (RUN-100)

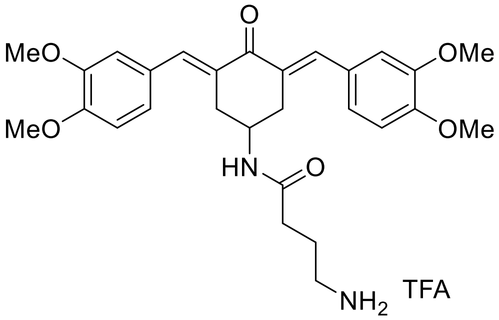

To a mixture of 3,5-bis((*E*)-3,4-dimethoxybenzylidene)-4-oxocyclohexan-1-aminium trifluoroacetate (**RUN-81**) (0.10 g, 0.19 mmol) and Et_3N (0.1 mL, 0.72 mmol) in DMF (3 mL) was added HBTU (0.11 g, 0.29 mmol) and 4-((*tert*-butoxycarbonyl)amino)butanoic acid (0.06 g, 0.29 mmol). The reaction mixture was stirred at ambient temperature for 1 hour. Solvent was evaporated under reduced pressure and the residue was purified by reverse-phase flash chromatography (eluent H_2O (0.1% TFA):MeCN, gradient elution) to obtain Boc-protected compound that was dissolved in DCM (0.5 mL), cooled down with ice bath and TFA (0.5 mL) was added dropwise. The solution was stirred at ambient temperature for 1.5 hour. Solvent was evaporated under reduced pressure and the residue was purified by reverse-phase flash chromatography (eluent H_2O (0.1% TFA):MeCN, gradient elution). The reaction provided 0.05 g of the title compound in 43% yield.

^{1}H NMR (401 MHz, DMSO-*d*$_6$) δ 8.17 (d, *J* = 6.6 Hz, 1H), 7.70 (br s, 3H), 7.67 (s, 2H), 7.17 – 7.08 (m, 4H), 7.04 (d, *J* = 8.2 Hz, 2H), 3.93 – 3.83 (m, 1H), 3.81 (s, 6H), 3.80 (s, 6H), 3.15 (dd, *J* = 15.9, 3.9 Hz, 2H), 2.95 – 2.80 (m, 2H), 2.78 – 2.70 (m, 2H), 2.21 – 2.13 (m, 2H), 1.80 – 1.64 (m, 2H). **^{13}C NMR** (101 MHz, DMSO-*d*$_6$) δ 187.3, 171.0, 149.7, 148.5, 137.6, 131.6, 127.9, 123.6, 114.2, 111.6, 55.6, 55.6, 44.5, 38.6, 33.4, 31.9, 23.1. **HRMS** (ESI+): m/z calculated for $C_{28}H_{35}O_6N_2$ = 495.2490; found = 495.2487 $[M+H]^+$.

3-((3,5-Bis((*E*)-3,4-dimethoxybenzylidene)-4-oxocyclohexyl)amino)-*N,N*-dimethyl-3-oxopropan-1-aminium trifluoroacetate (RUN-101)

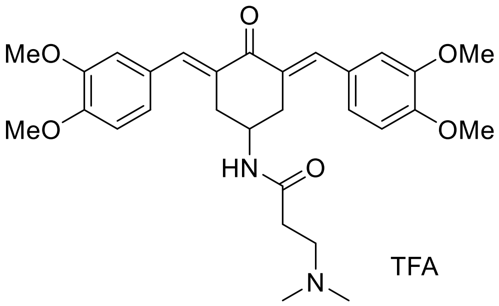

To a mixture of 3,5-bis((*E*)-3,4-dimethoxybenzylidene)-4-oxocyclohexan-1-aminium trifluoroacetate (**RUN-81**) (0.10 g, 0.19 mmol) and Et_3N (0.12 mL, 0.86 mmol) in DMF (3 mL) was added HBTU (0.11 g, 0.29 mmol) and 2-carboxy-*N,N*-dimethylethan-1-aminium hydrochloride (0.04 g, 0.29 mmol). The reaction mixture was stirred at ambient temperature for 2 hours. Solvent was evaporated under reduced pressure and the residue was purified by reverse-phase flash chromatography (eluent H_2O (0.1% TFA):MeCN, gradient elution). The reaction provided 0.09 g of the title compound in 78% yield.

^{1}H NMR (401 MHz, $CDCl_3$) δ 7.87 – 7.82 (m, 2H), 7.14 (d, *J* = 7.3 Hz, 1H), 7.07 (dd, *J* = 8.5, 2.0 Hz, 2H), 6.97 (d, *J* = 2.0 Hz, 2H), 6.89 (d, *J* = 8.4 Hz, 2H), 4.28 – 4.17 (m, 1H), 3.91 (s, 6H), 3.90 (s, 6H), 3.30 (t, *J* = 6.8 Hz, 2H), 3.26 – 3.16 (m, 2H), 3.06 – 2.95 (m, 2H), 2.74 – 2.69 (m, 8H). **^{13}C NMR** (101 MHz, $CDCl_3$) δ 188.1, 168.5, 162.6 (q, *J* = 35.7 Hz), 150.1, 148.9, 139.8, 130.5, 128.4, 124.1, 114.0, 111.1, 56.1, 54.3, 45.2, 43.5, 33.8, 31.8. **HRMS** (ESI+): m/z calculated for $C_{29}H_{37}O_6N_2$ = 509.2646; found = 509.2643 $[M+H]^+$.

2-((3,5-Bis((*E*)-3,4-dimethoxybenzylidene)-4-oxocyclohexyl)amino)-*N,N*-diethyl-2-oxoethan-1-aminium trifluoroacetate (RUN-102)

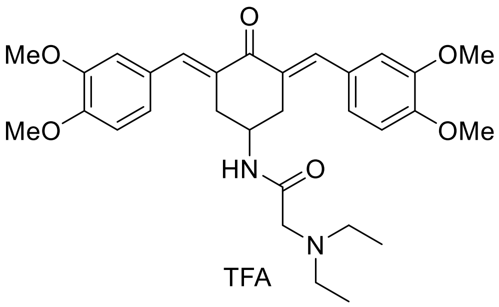

To a mixture of 3,5-bis((*E*)-3,4-dimethoxybenzylidene)-4-oxocyclohexan-1-aminium trifluoroacetate (**RUN-81**) (0.10 g, 0.19 mmol) and Et_3N (0.12 mL, 0.86 mmol) in DMF (3 mL) was added HBTU (0.11 g, 0.29 mmol) and *N*-(carboxymethyl)-*N*-ethylethanaminium hydrochloride (0.05 g, 0.29 mmol). The reaction mixture was stirred at ambient temperature for 2 hours. Solvent was evaporated under reduced pressure and the residue was purified by reverse-phase flash chromatography (eluent H_2O (0.1% TFA):MeCN, gradient elution). The reaction provided 0.10 g of the title compound in 79% yield.

^{1}H NMR (401 MHz, $CDCl_3$) δ 8.93 (d, *J* = 7.2 Hz, 1H), 7.83 (s, 2H), 7.07 (dd, *J* = 8.4, 2.0 Hz, 2H), 6.97 (d, *J* = 2.0 Hz, 2H), 6.89 (d, *J* = 8.4 Hz, 2H), 4.17 – 4.13 (m, 1H), 3.91 (s, 6H), 3.90 (s, 6H), 3.71 (s, 2H), 3.29 – 3.24 (m, 2H), 3.21 – 3.14 (m, 4H), 2.98 – 2.86 (m, 2H), 1.30 (t, *J* = 7.3 Hz, 6H). **^{13}C NMR** (101 MHz, $CDCl_3$) δ 187.9, 163.0 (q, *J* = 35.3 Hz), 162.8, 150.1, 148.9, 139.5, 130.7, 128.4, 124.0, 113.8, 111.1, 54.3, 48.3, 46.0, 33.9, 9.0. **HRMS** (ESI+): m/z calculated for $C_{30}H_{39}O_6N_2$ = 523.2803; found = 523.2799 $[M+H]^+$.

3-((3,5-Bis((*E*)-3,4-dimethoxybenzylidene)-4-oxocyclohexyl)amino)-3-oxopropan-1-aminium trifluoroacetate (RUN-104)

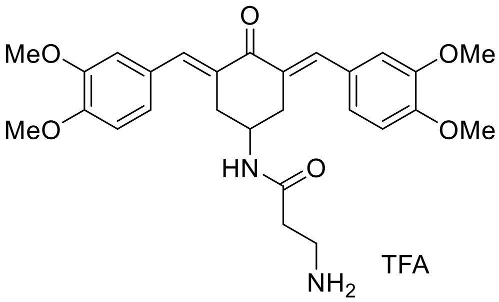

To a mixture of 3,5-bis((*E*)-3,4-dimethoxybenzylidene)-4-oxocyclohexan-1-aminium trifluoroacetate (**RUN-81**) (0.10 g, 0.19 mmol) and Et_3N (0.1 mL, 0.72 mmol) in DMF (3 mL) was added HBTU (0.11 g, 0.29 mmol) and 3-((*tert*-butoxycarbonyl)amino)propanoic acid (0.06 g, 0.29 mmol). The reaction mixture was stirred at ambient temperature for 2 hours. Solvent was evaporated under reduced pressure and the residue was purified by reverse-phase flash chromatography (eluent H_2O (0.1% TFA):MeCN, gradient elution) to obtain Boc-protected compound that was dissolved in DCM (0.5 mL), cooled down with ice bath and TFA (0.5 mL) was added dropwise. The solution was stirred at ambient temperature for 1.5 hour. Solvent was evaporated under reduced pressure and the residue was purified by reverse-phase flash chromatography (eluent H_2O (0.1% TFA):MeCN, gradient elution). The reaction provided 0.06 g of the title compound in 52% yield.

^{1}H NMR (401 MHz, DMSO-*d*$_6$) δ 8.39 (d, *J* = 6.8 Hz, 1H), 7.70 (br s, 3H), 7.68 (s, 2H), 7.16 – 7.09 (m, 4H), 7.05 (d, *J* = 8.2 Hz, 2H), 3.97 – 3.87 (m, 1H), 3.81 (s, 6H), 3.80 (s, 6H), 3.17 (dd, *J* = 16.2, 4.0 Hz, 2H), 2.98 – 2.83 (m, 4H), 2.44 (t, *J* = 6.9 Hz, 2H). **^{13}C NMR** (101 MHz, DMSO-*d*$_6$) δ 187.2, 169.1, 149.7, 148.5, 137.6, 131.6, 128.0, 127.9, 123.6, 114.3, 111.6, 55.6, 44.6, 35.2, 33.5, 32.0. **HRMS** (ESI+): m/z calculated for $C_{27}H_{33}O_6N_2$ = 481.2333; found = 481.2333 $[M+H]^+$.

3.3 ^{1}H & ^{13}C NMR Spectra of All New Compounds

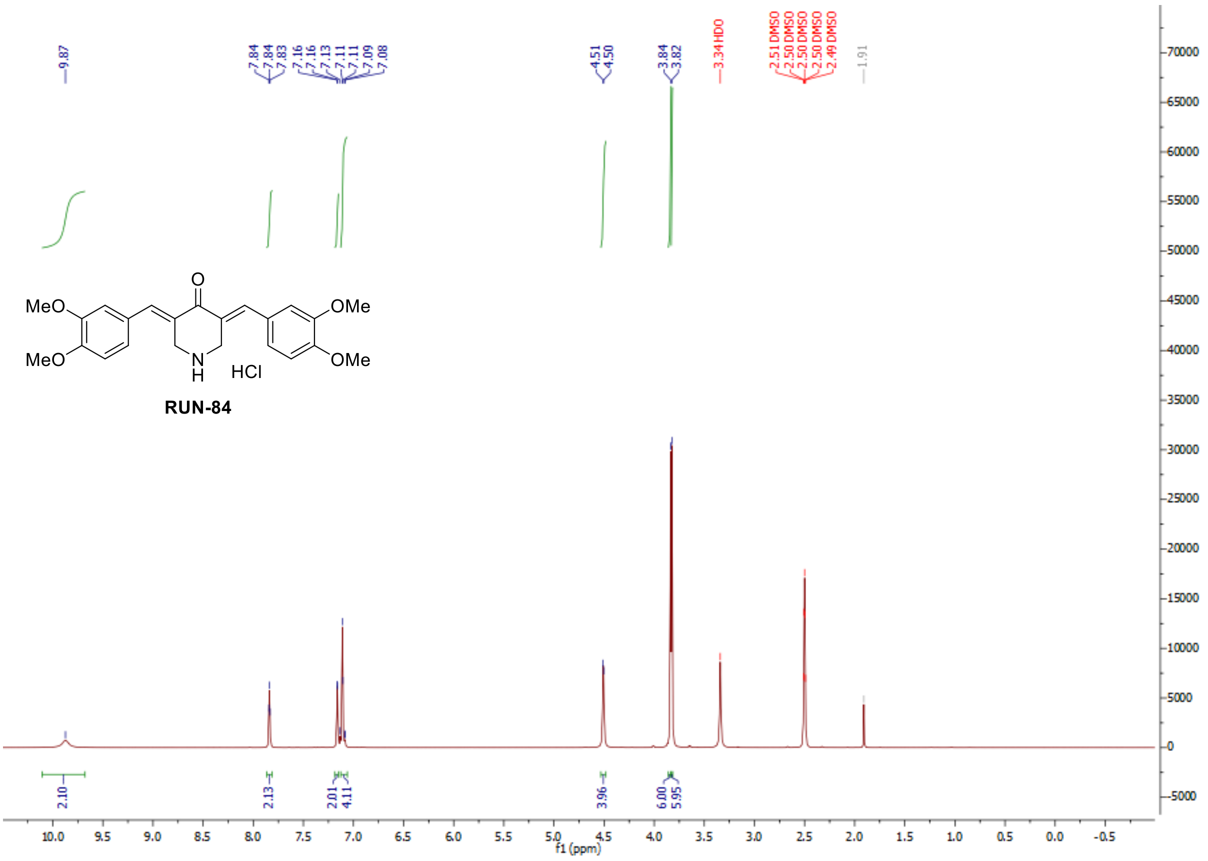

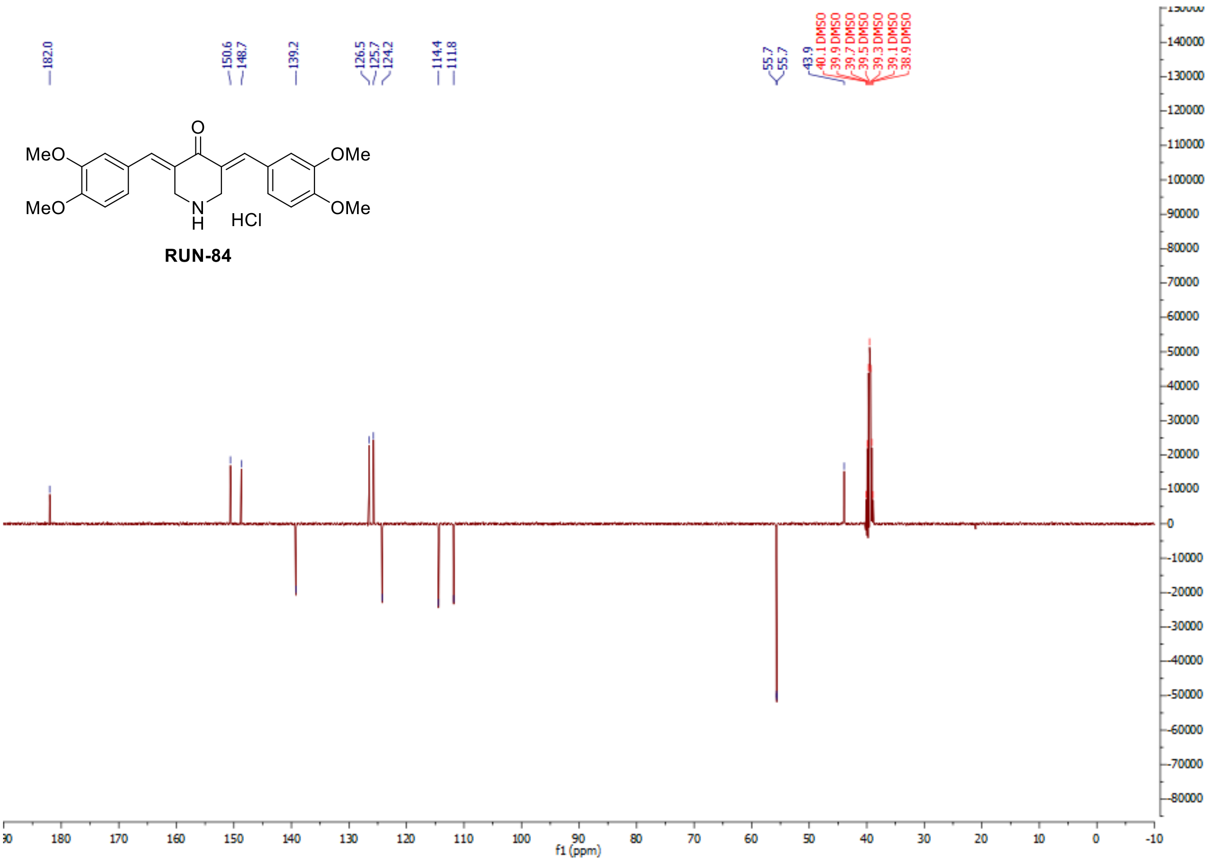

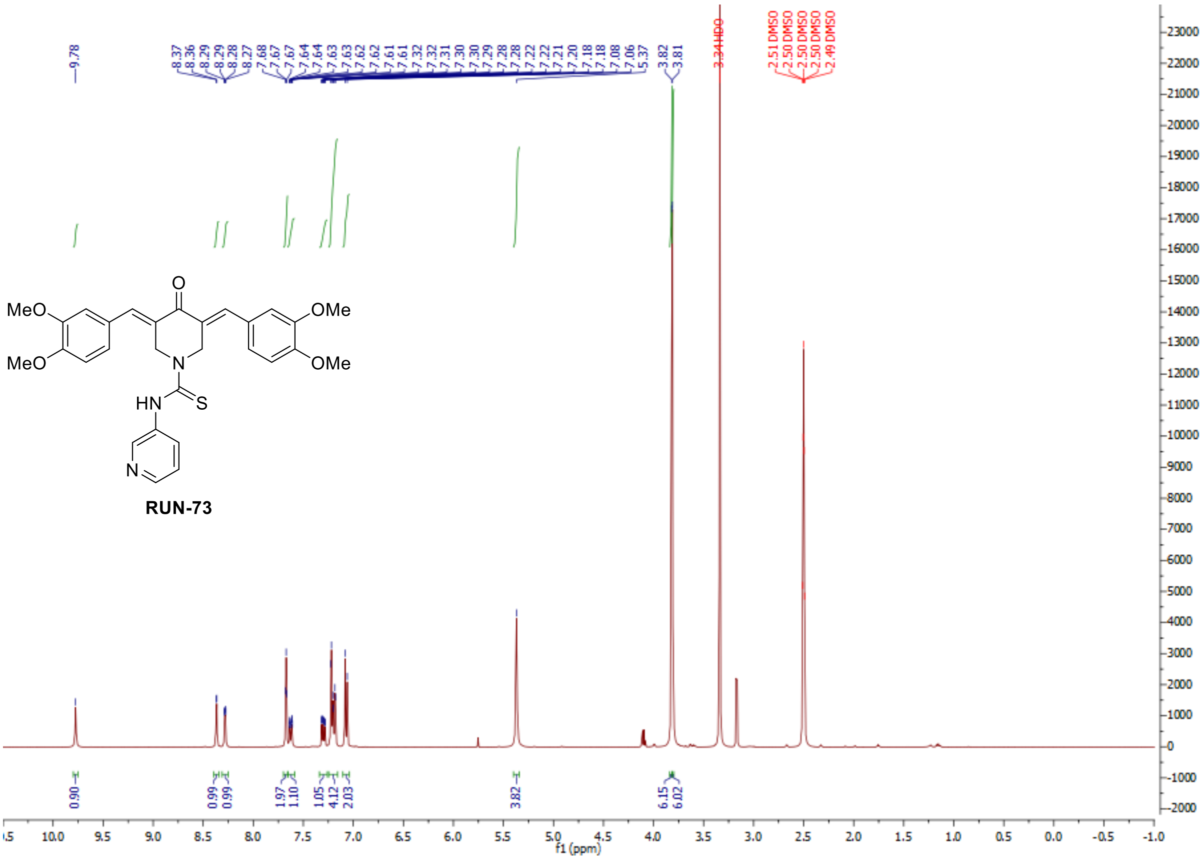

MeO
MeO
OMe
OMe
O
N
HN
S
N
RUN-73
f1 (ppm)

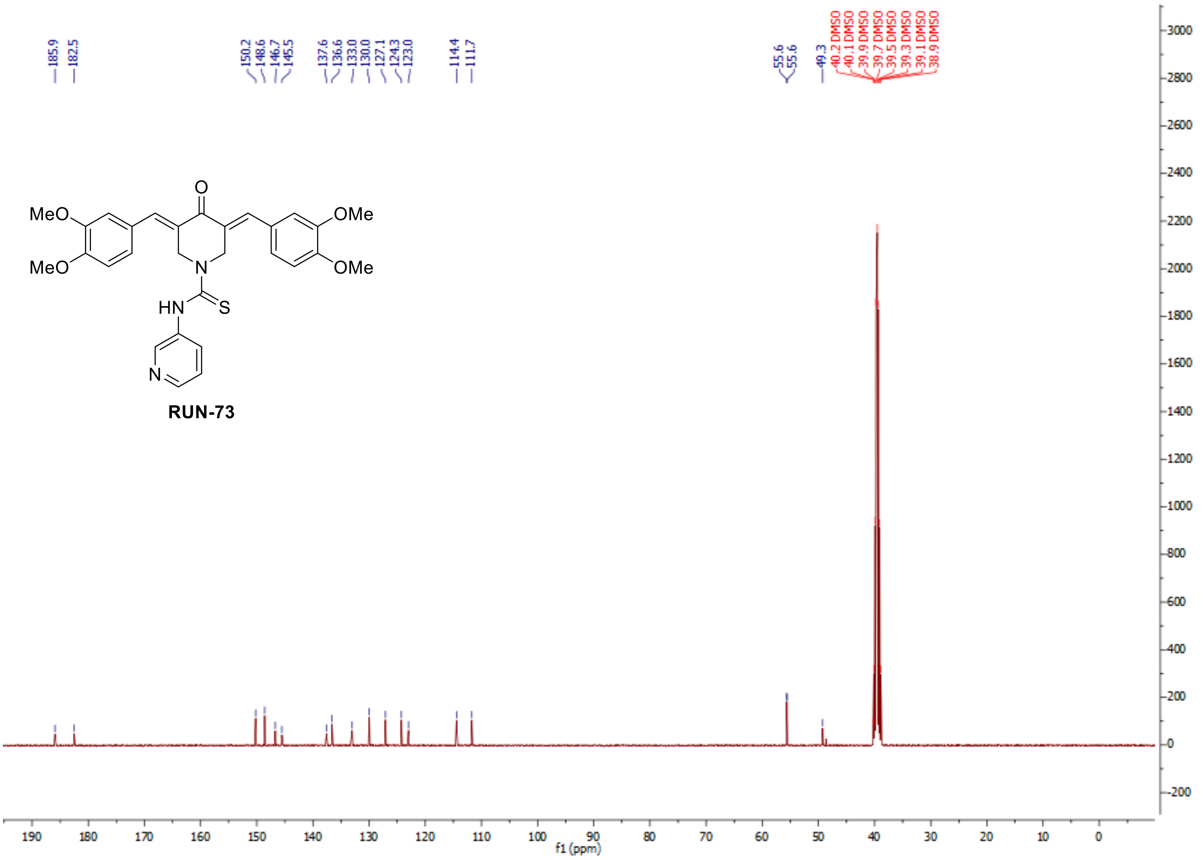

MeO
MeO
OMe
OMe
O
N
HN
S
N
RUN-73
f1 (ppm)

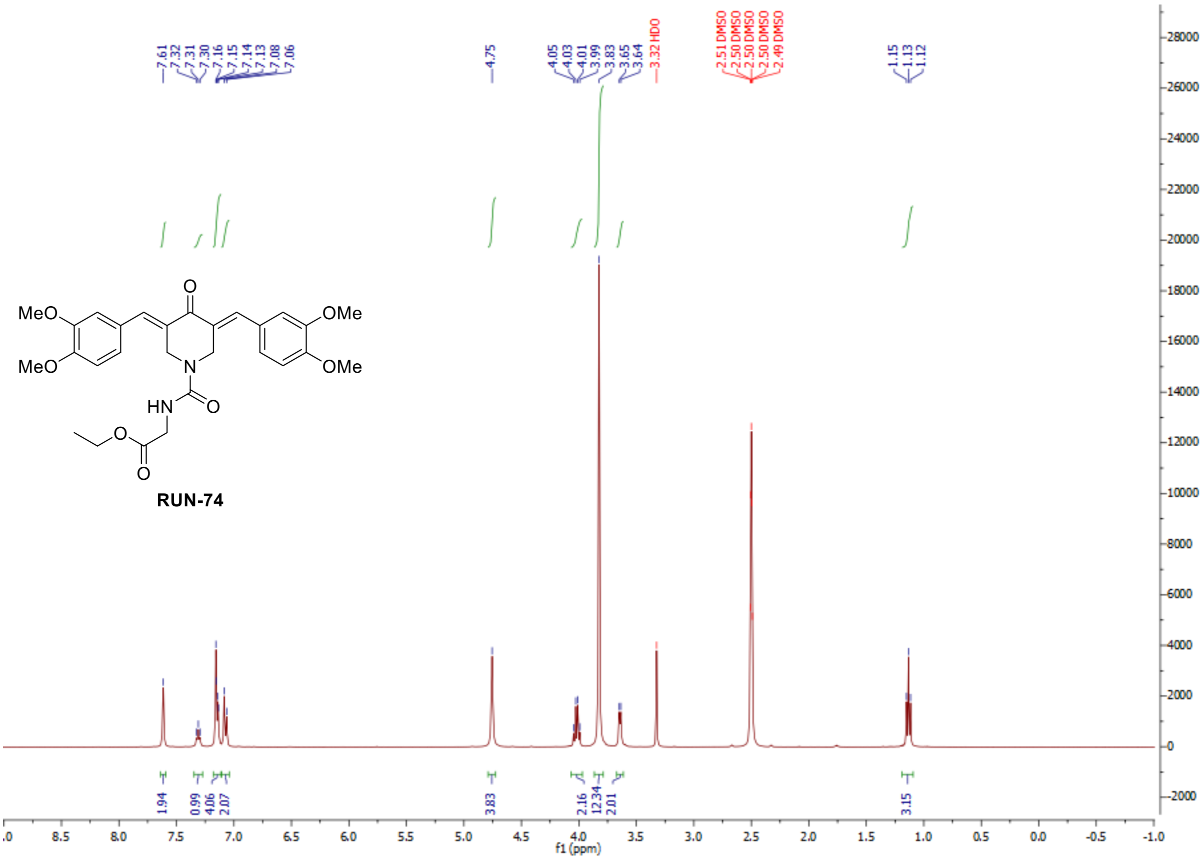

RUN-74

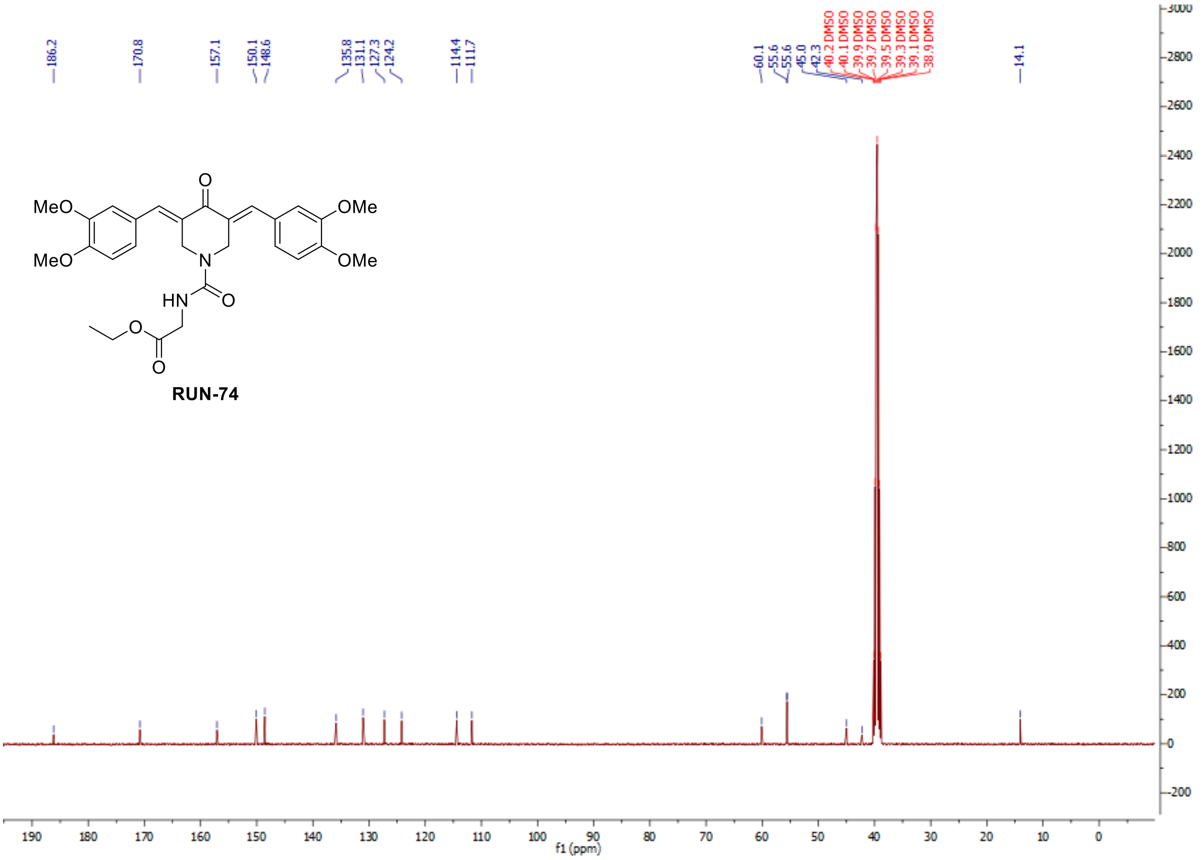

RUN-74

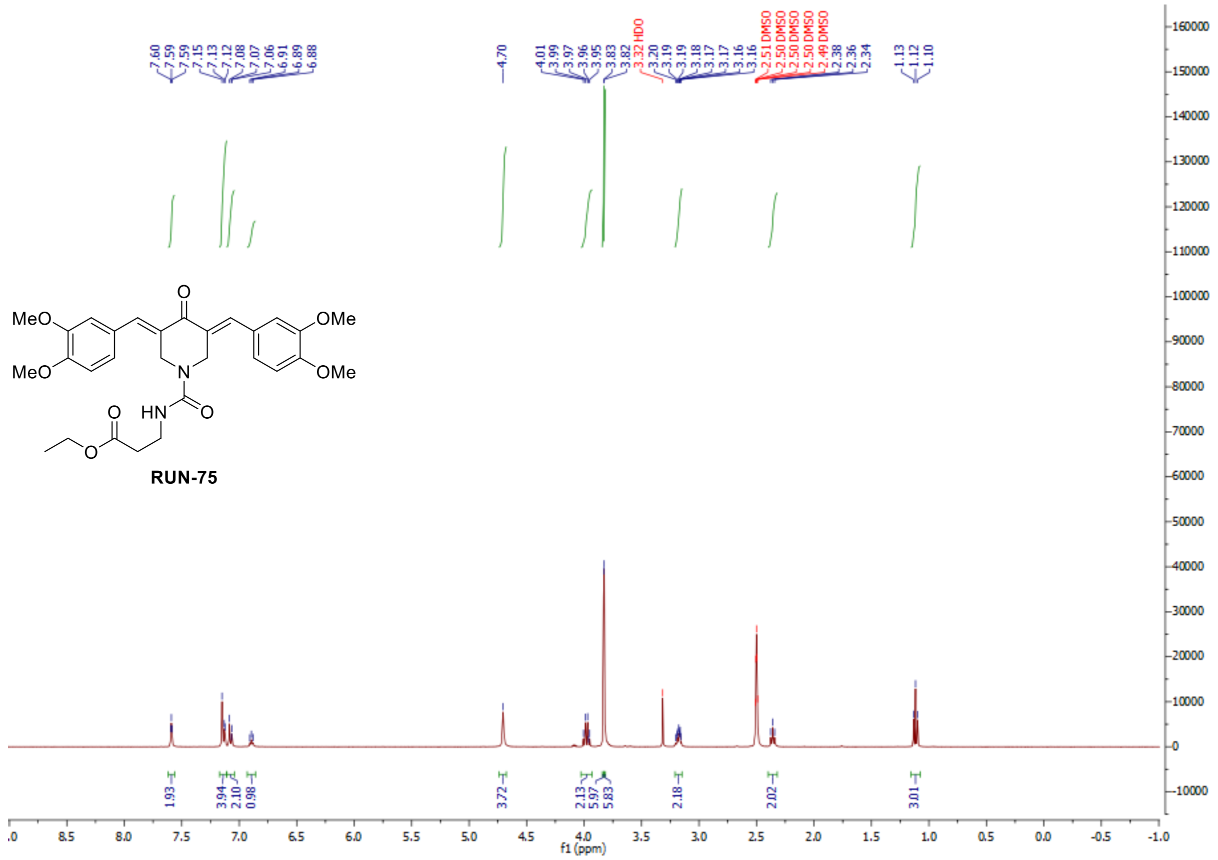
RUN-75
f1 (ppm)

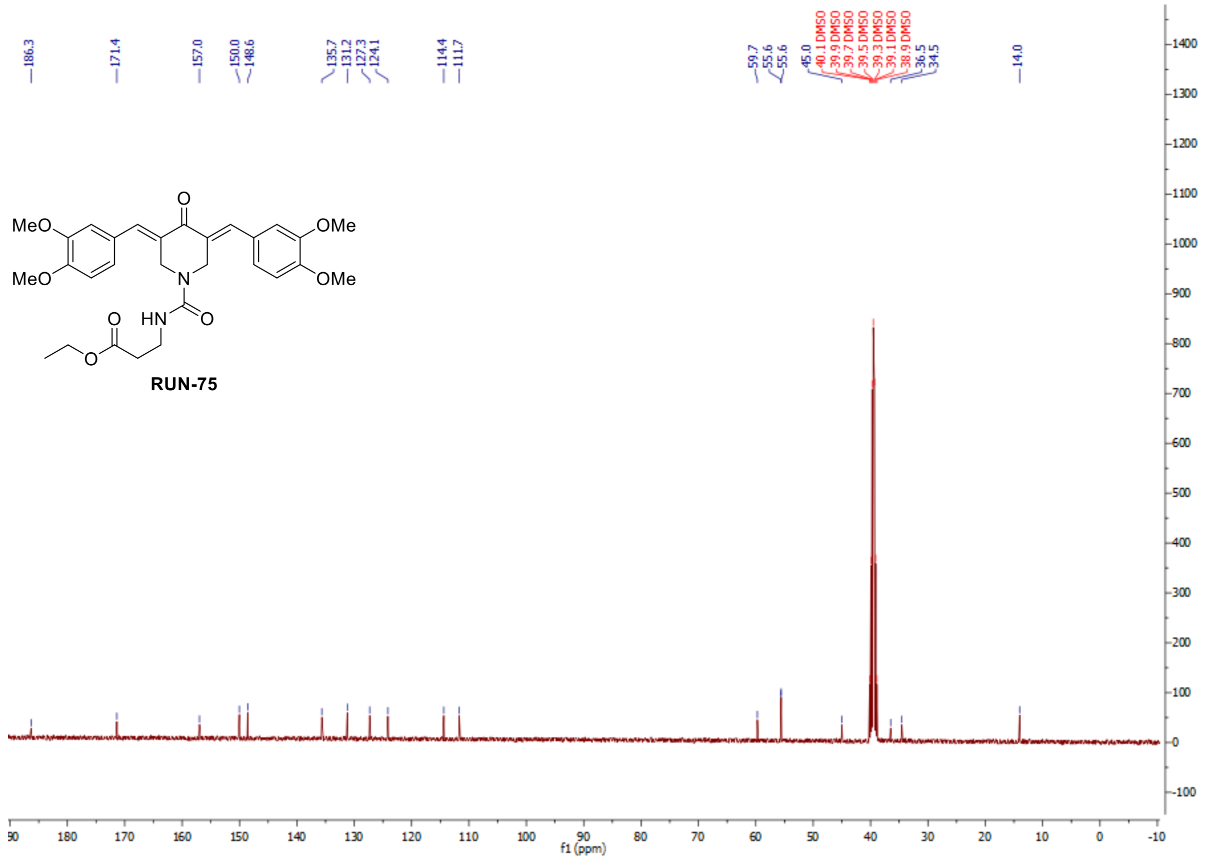
RUN-75
f1 (ppm)

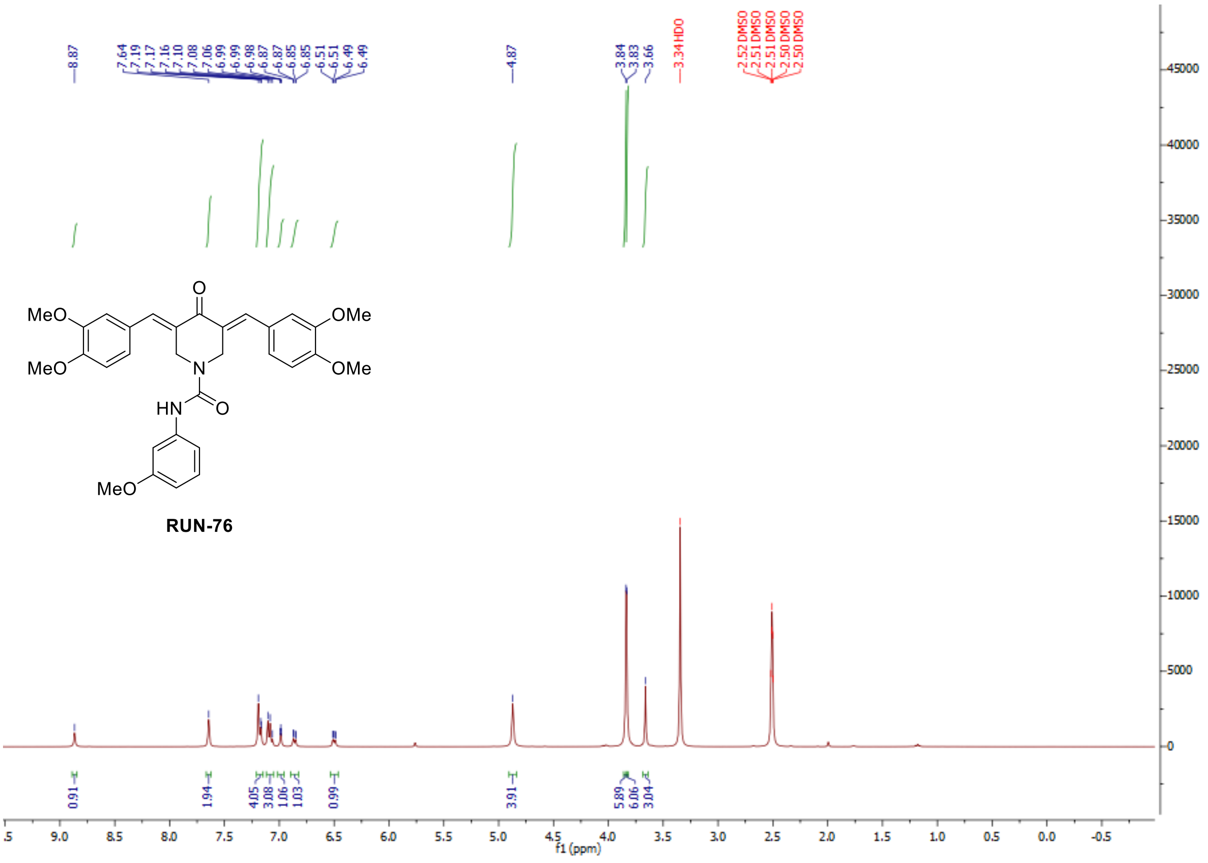

RUN-76

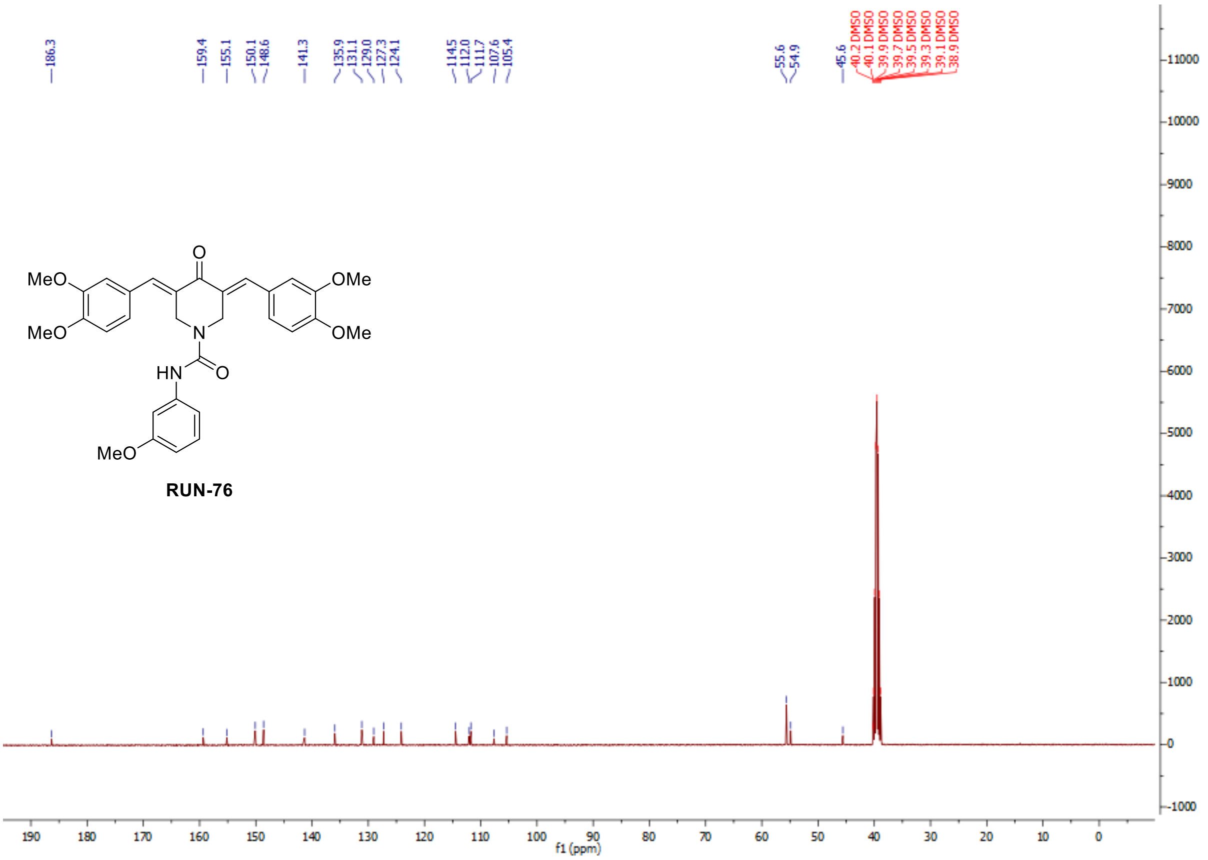

RUN-76

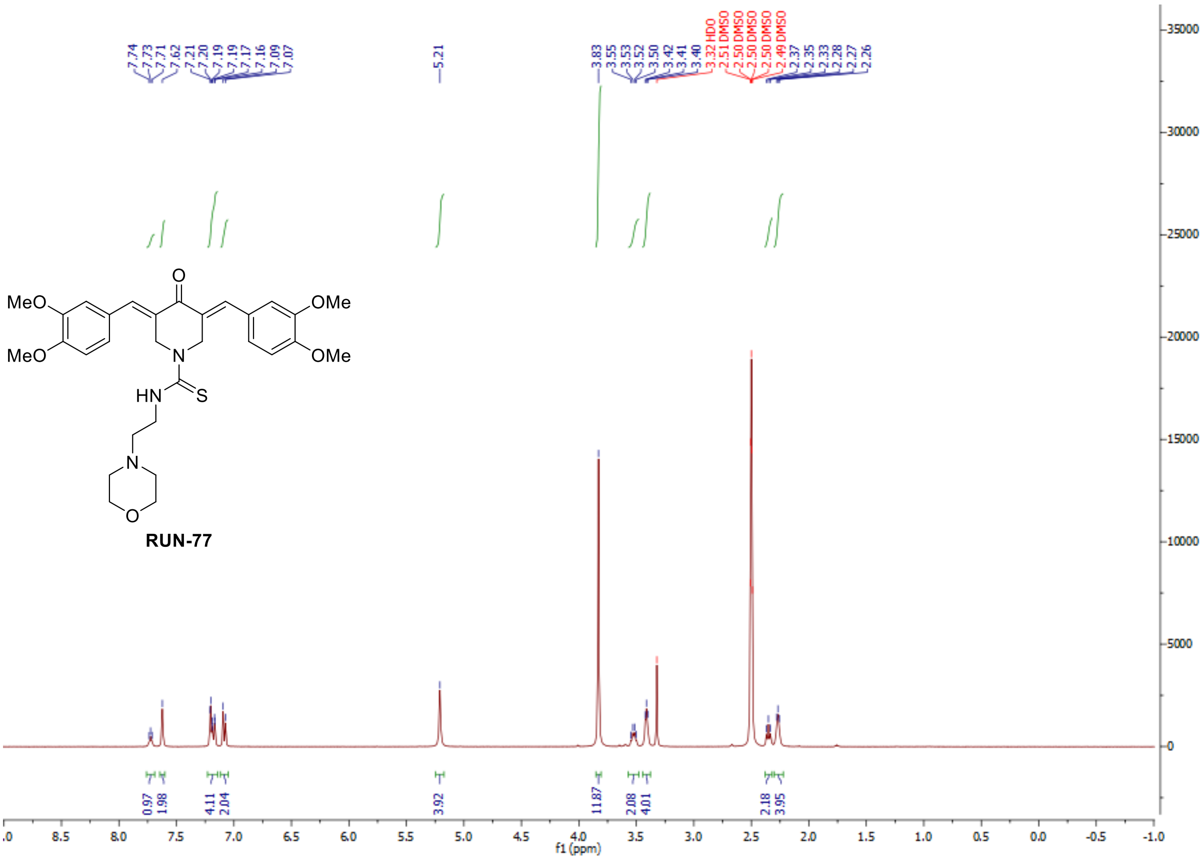
7.74
7.73
7.71
7.62
7.21
7.20
7.19
7.19
7.17
7.16
7.09
7.07
5.21
3.83
3.55
3.53
3.52
3.50
3.42
3.41
3.40
3.32 HDO
2.51 DMSO
2.50 DMSO
2.50 DMSO
2.50 DMSO
2.49 DMSO
2.37
2.35
2.33
2.28
2.27
2.26
MeO
MeO
O
OMe
OMe
N
HN
S
N
O
RUN-77
0.97
1.98
4.11
2.04
3.92
11.87
2.08
4.01
2.18
3.95
f1 (ppm)

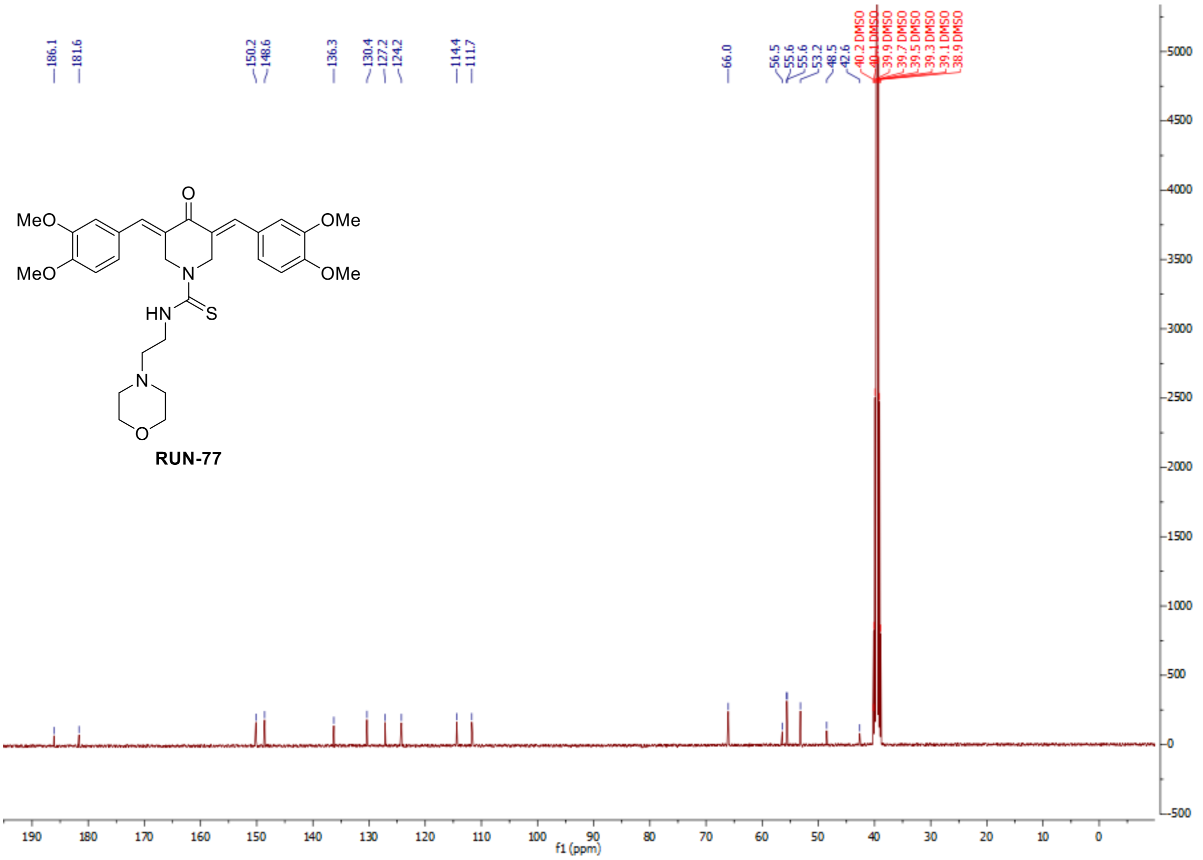
186.1
181.6
150.2
148.6
136.3
130.4
127.2
124.2
114.4
111.7
66.0
56.5
55.6
55.6
53.2
48.5
42.6
40.2 DMSO
40.1 DMSO
39.9 DMSO
39.7 DMSO
39.5 DMSO
39.3 DMSO
39.1 DMSO
38.9 DMSO
MeO
MeO
O
OMe
OMe
N
HN
S
N
O
RUN-77
f1 (ppm)

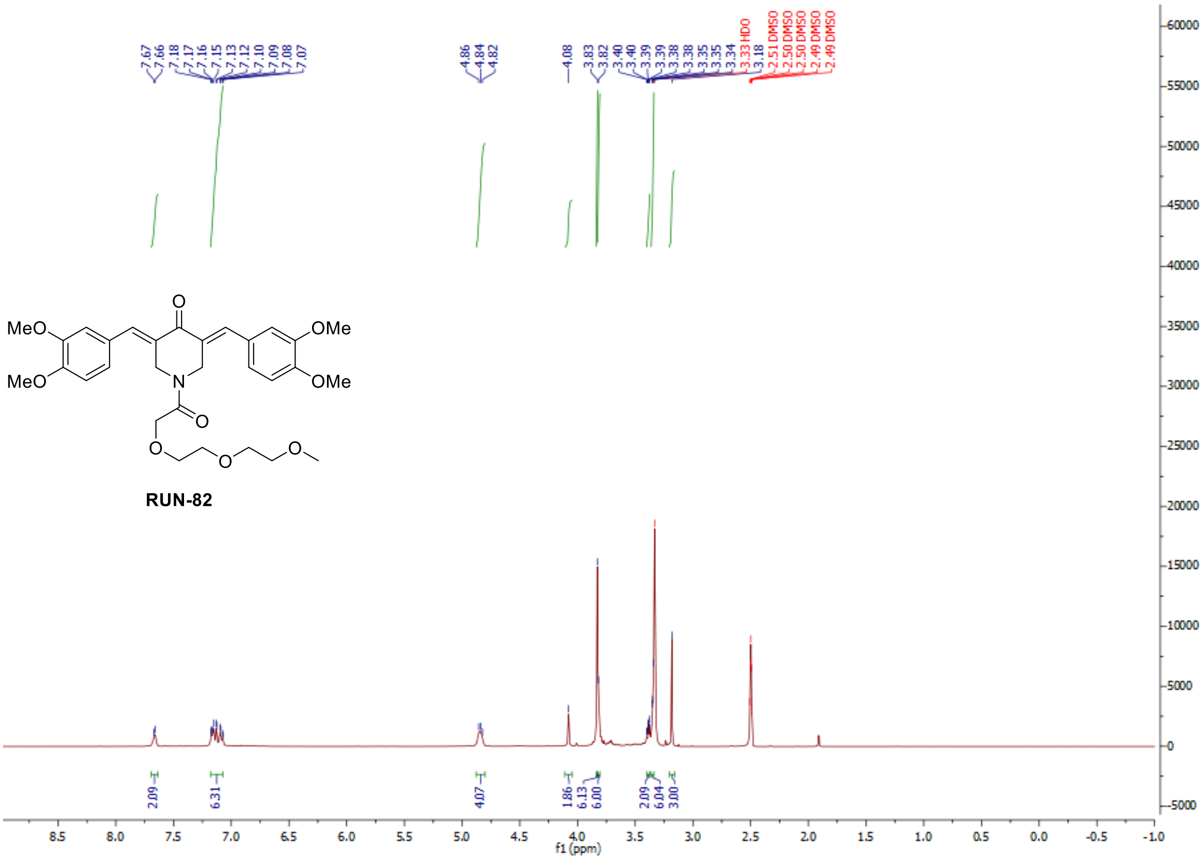

RUN-82

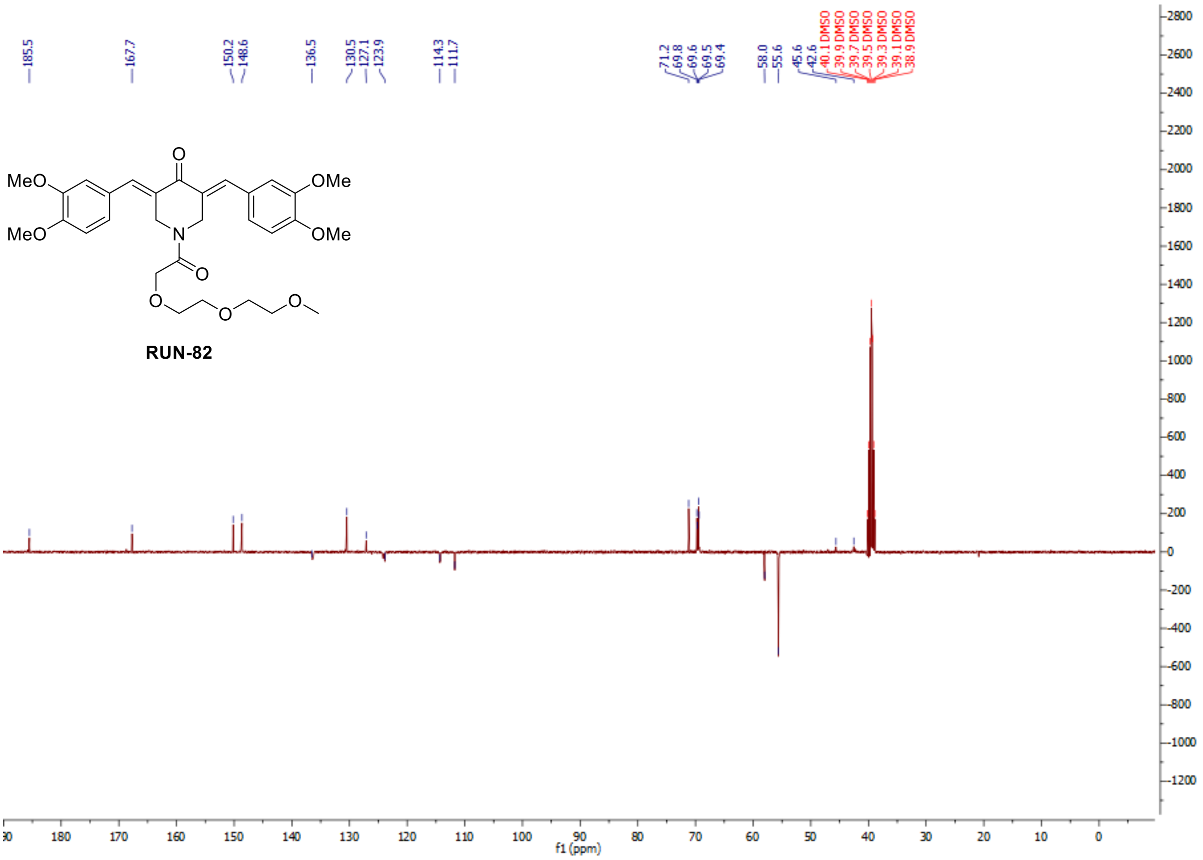

RUN-82

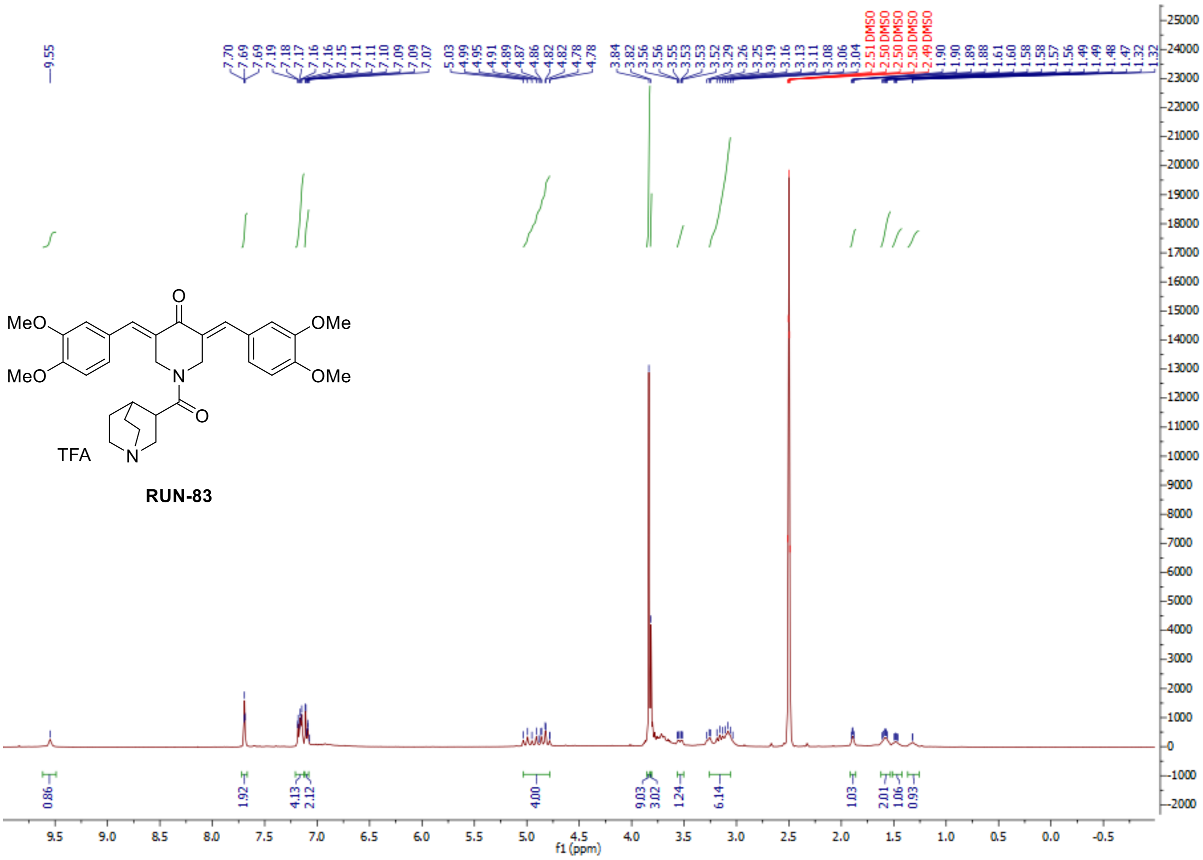
RUN-83
TFA
f1 (ppm)

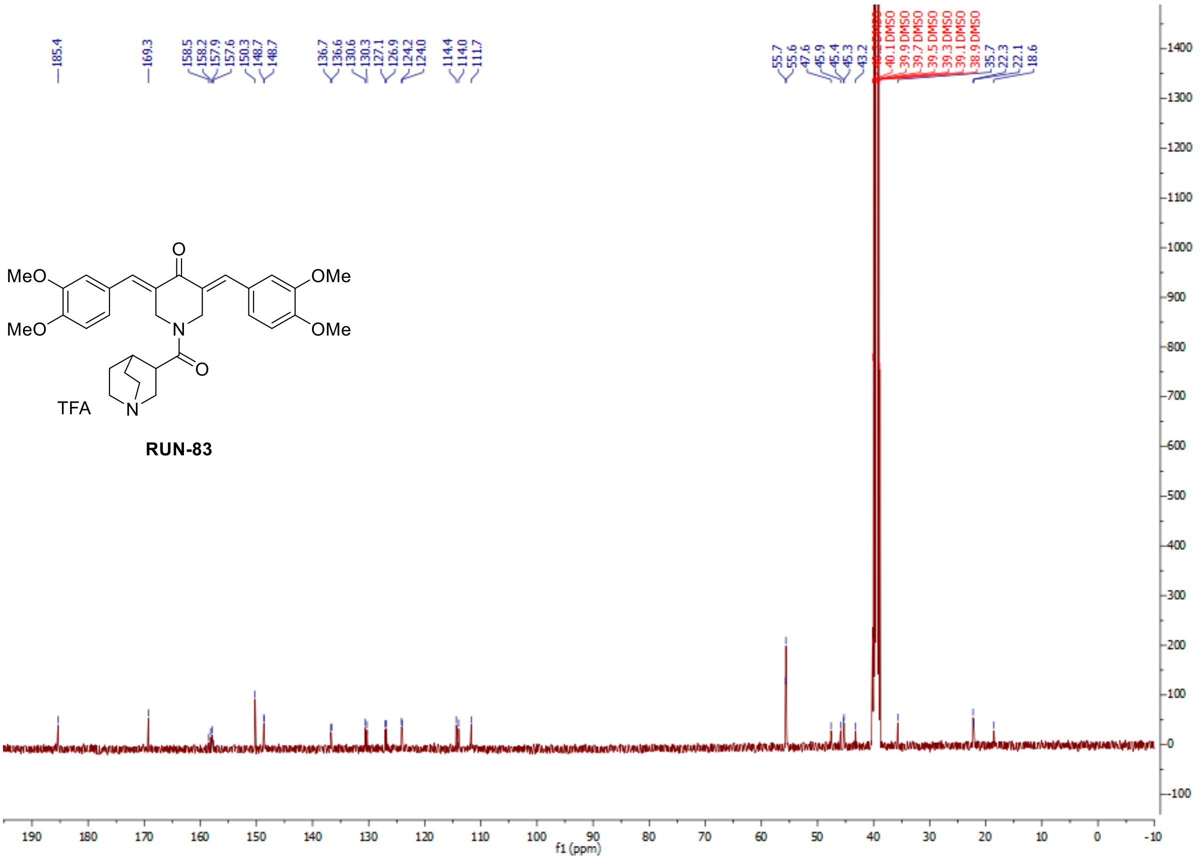
RUN-83
TFA
f1 (ppm)

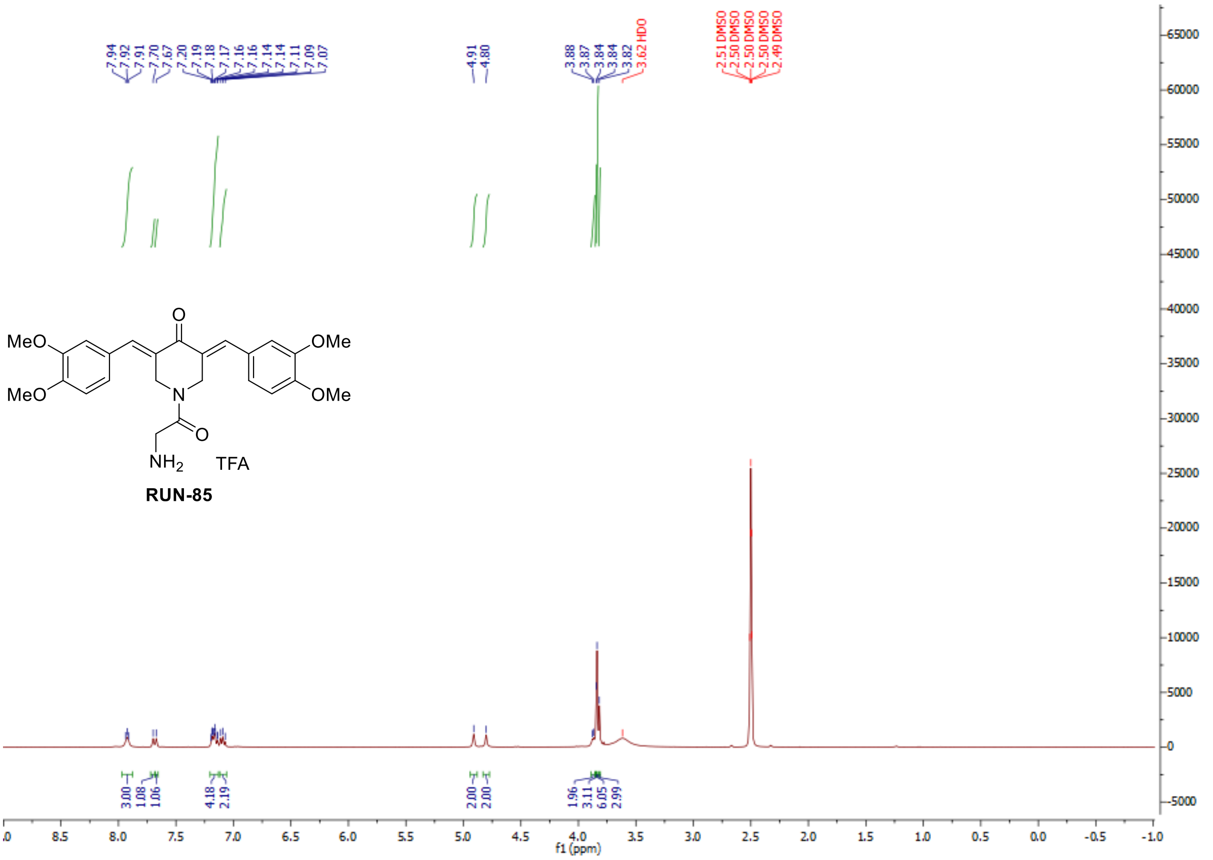

RUN-85
TFA
f1 (ppm)

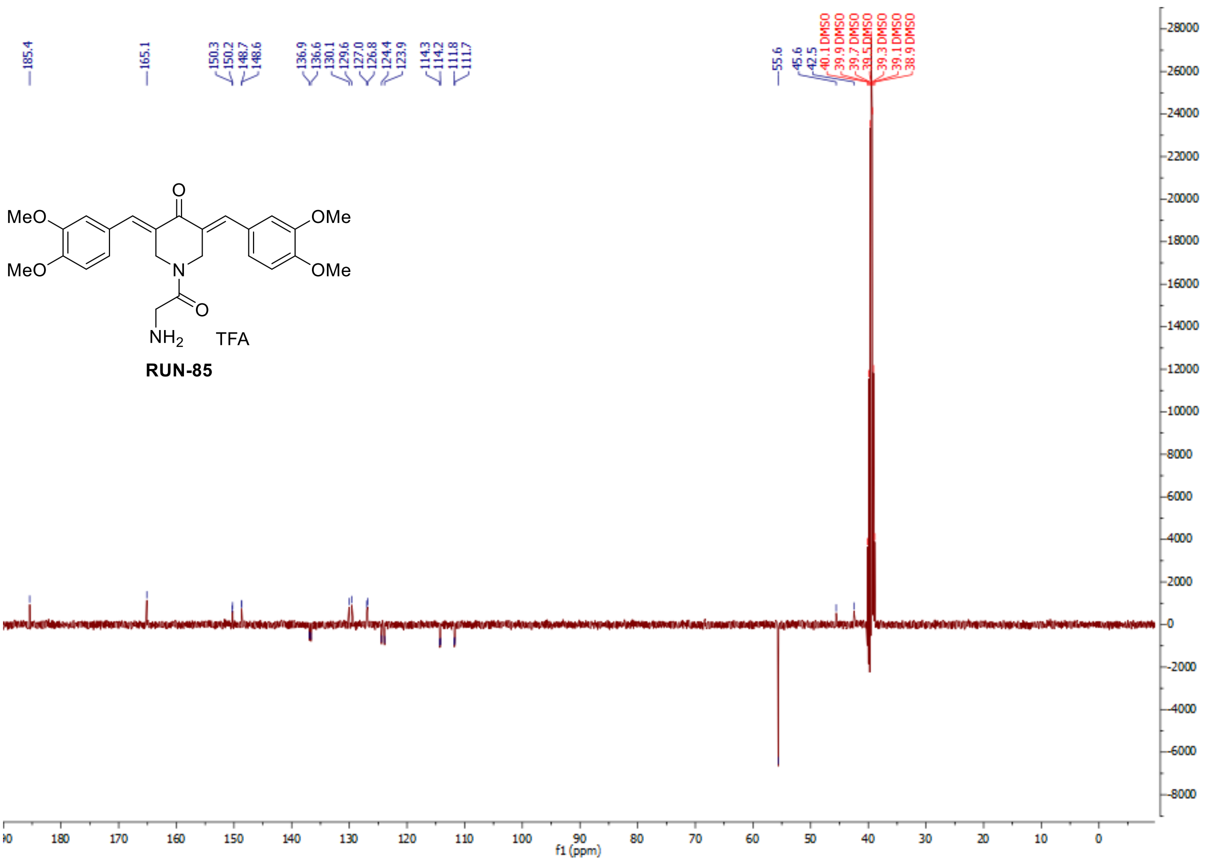

RUN-85
TFA
f1 (ppm)

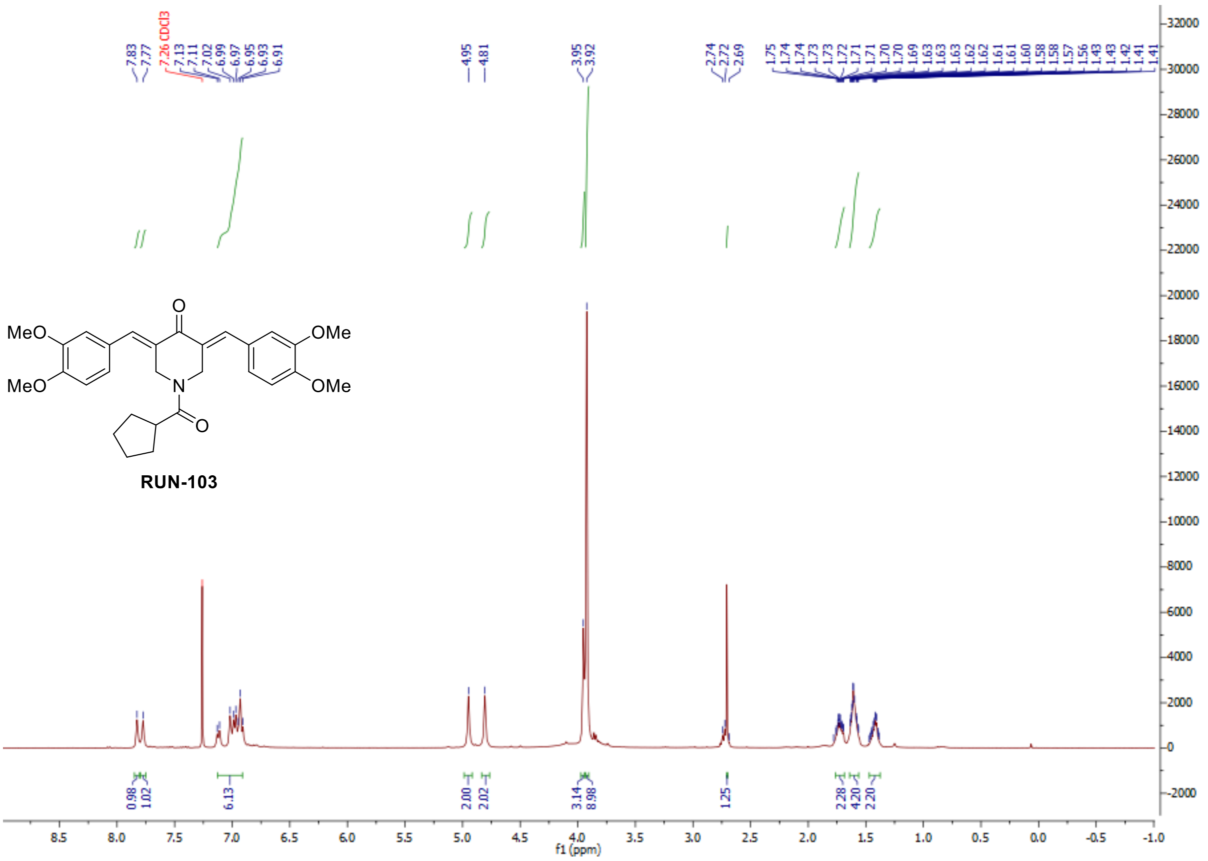

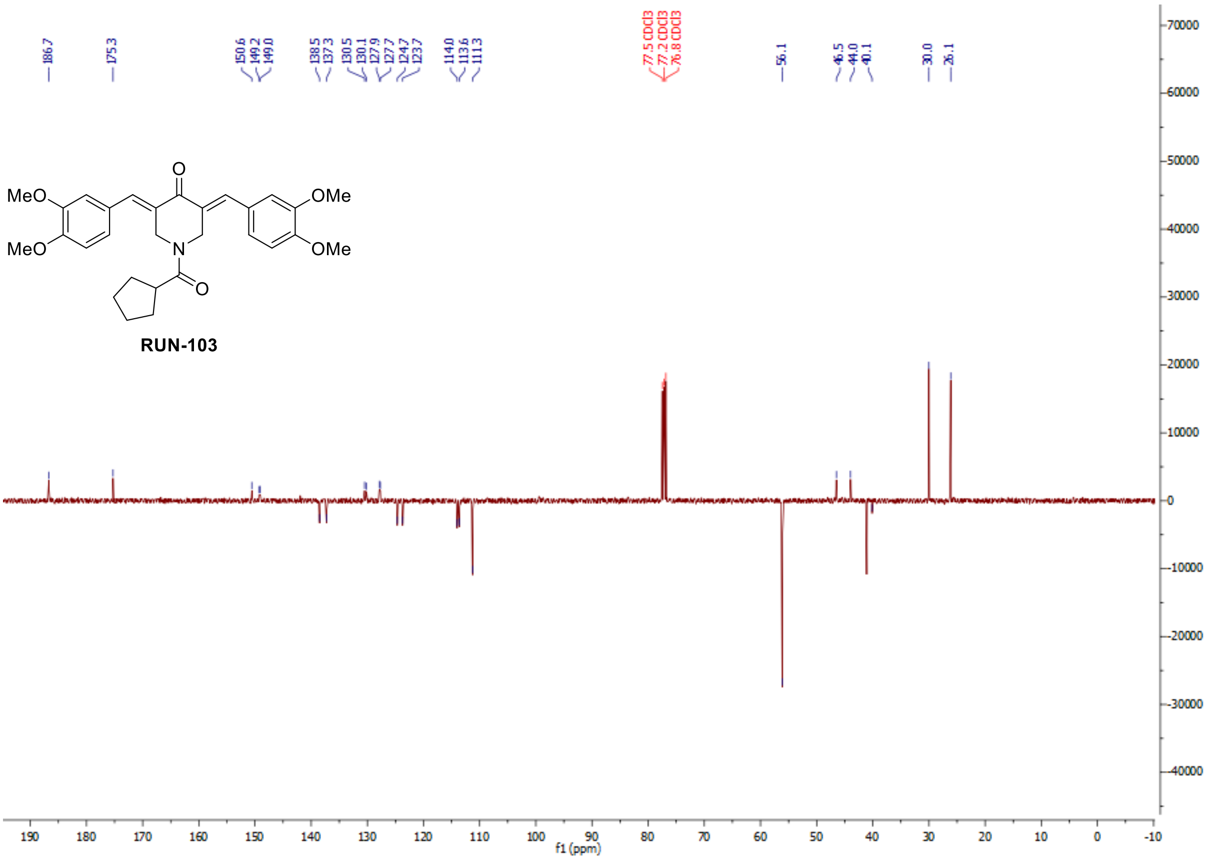

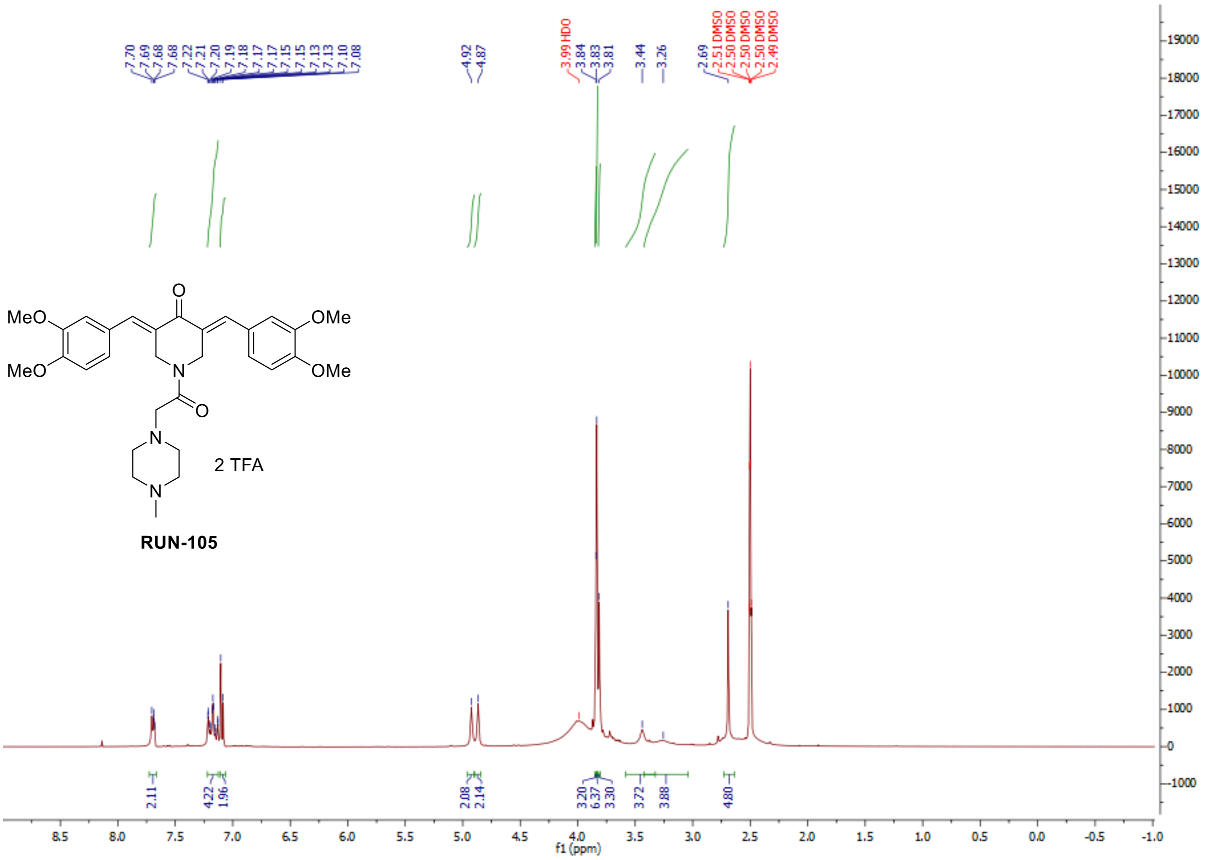

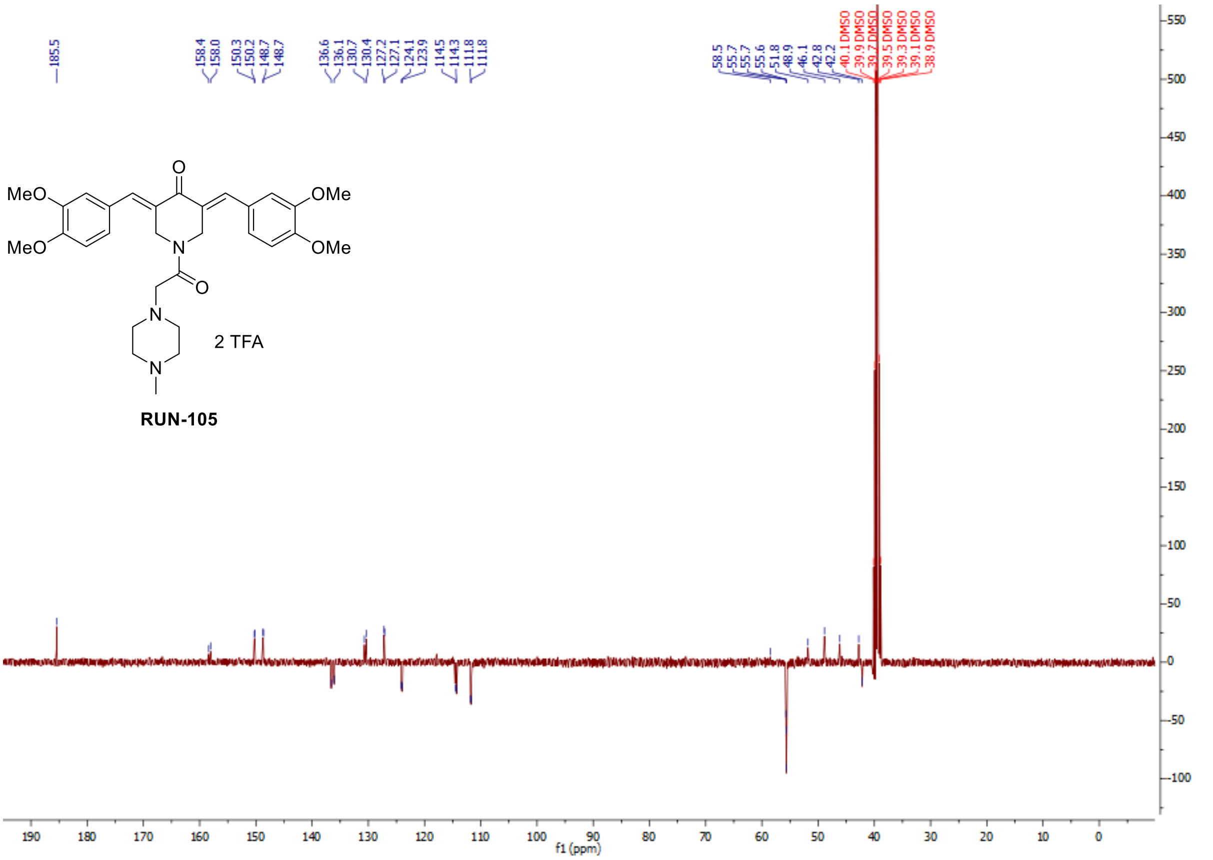

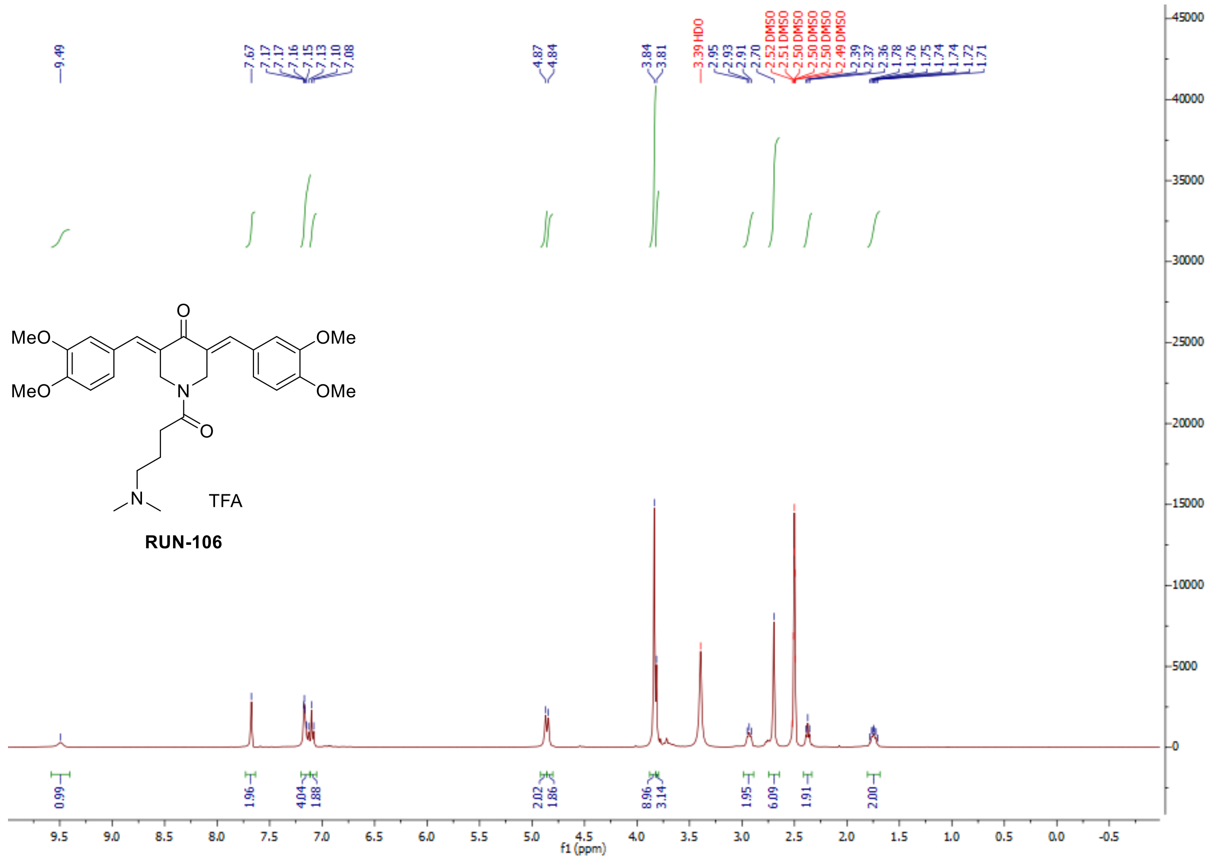

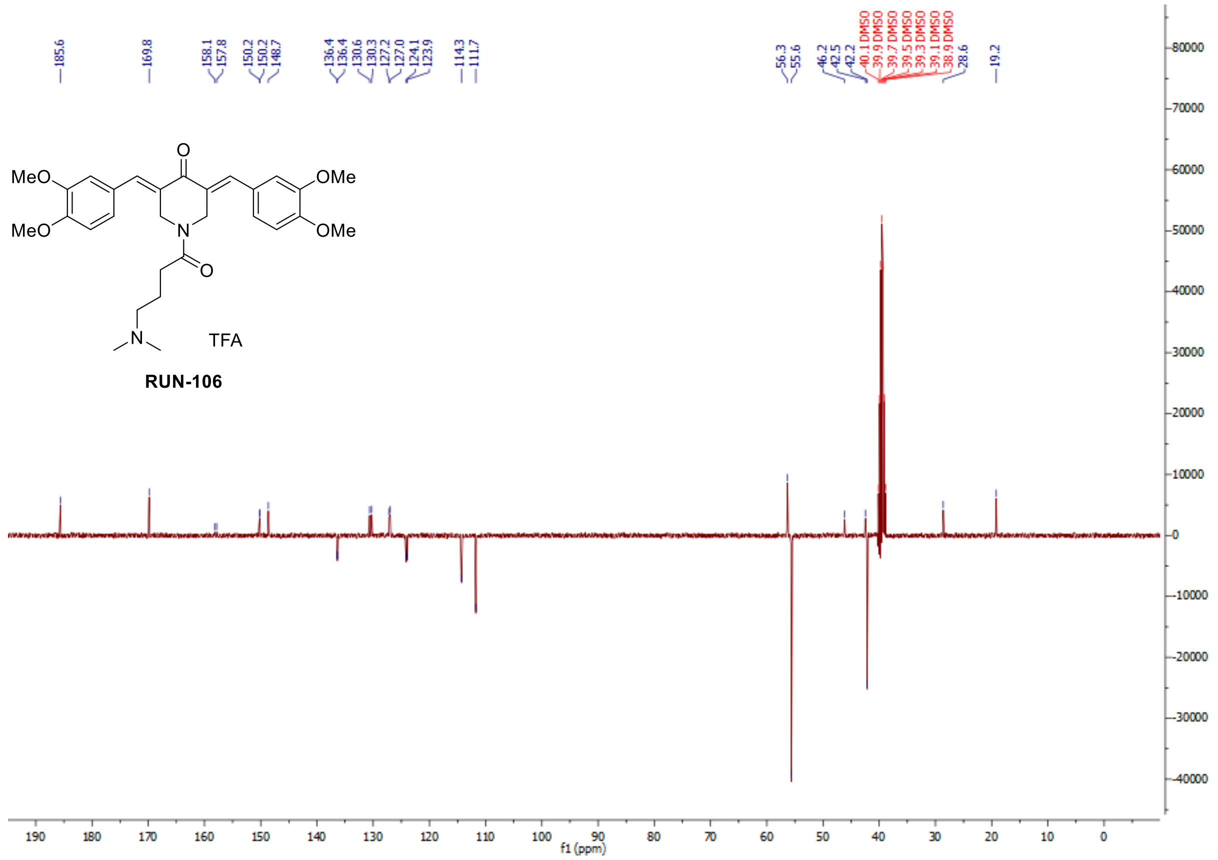

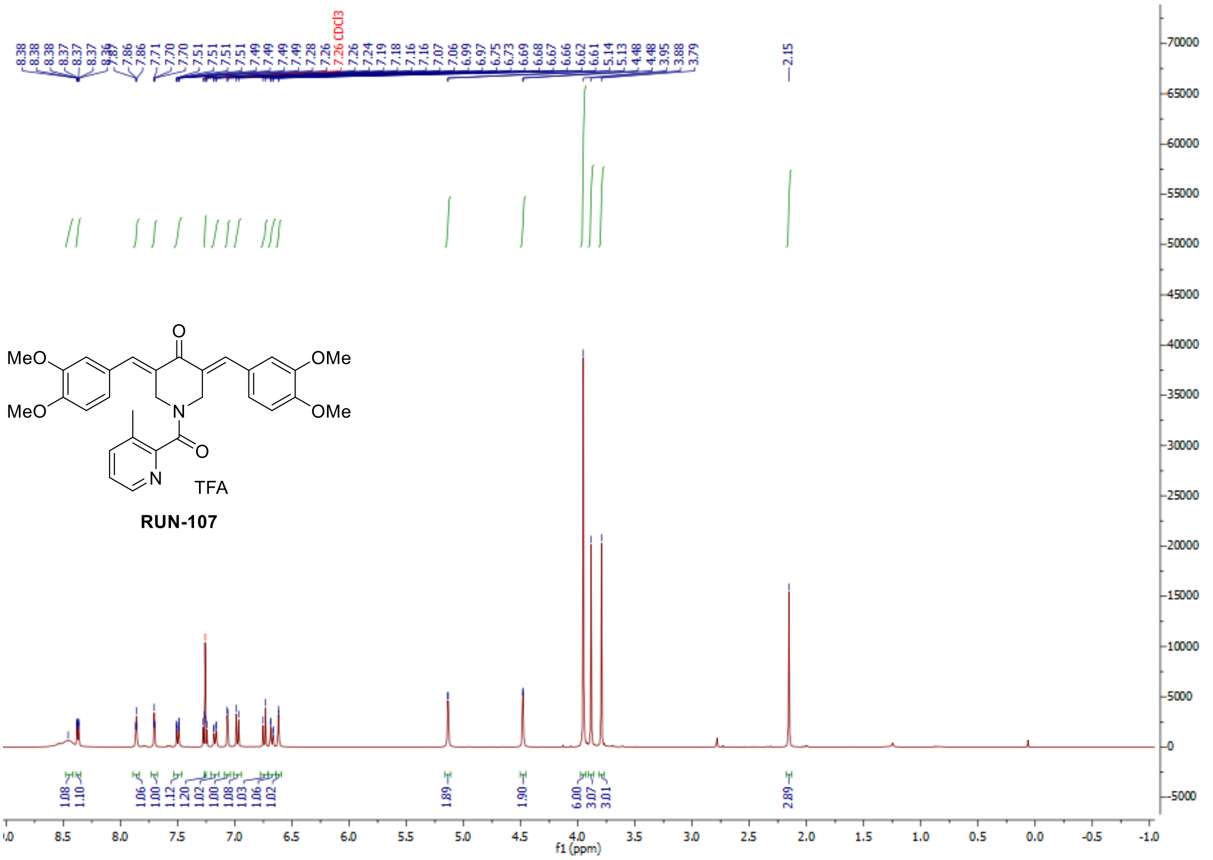
8.38 8.38 8.38 8.37 8.37 8.37 8.36 7.87 7.86 7.86 7.71 7.70 7.70 7.51 7.51 7.51 7.51 7.51 7.49 7.49 7.49 7.49 7.49 7.28 7.26 7.26 CDCl3 7.26 7.24 7.19 7.18 7.16 7.16 7.07 7.06 6.99 6.97 6.75 6.73 6.69 6.68 6.67 6.66 6.62 6.61 5.14 5.13 4.48 4.48 3.95 3.88 3.79
2.15
MeO
MeO
OMe
OMe
O
N
O
N
TFA
RUN-107
1.08 1.10 1.06 1.00 1.12 1.20 1.02 1.00 1.08 1.03 1.06 1.02 1.89 1.90 6.00 3.07 3.01 2.89
f1 (ppm)

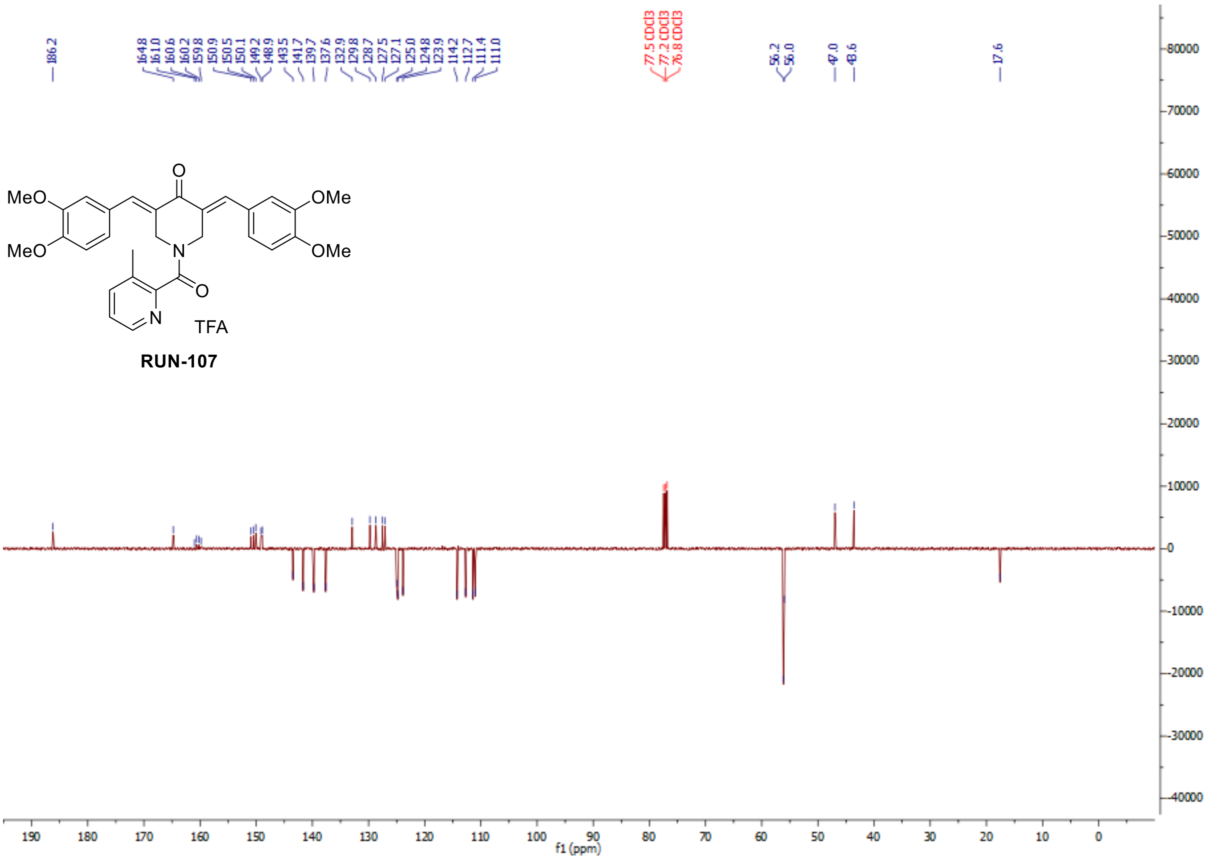
186.2
164.8 161.0 160.6 160.2 159.8 150.9 150.5 150.1 149.2 148.9 143.5 141.7 139.7 137.6 132.9 129.8 128.7 127.5 127.1 125.0 124.8 123.9 114.2 112.7 111.4 111.0
77.5 CDCl3 77.2 CDCl3 76.8 CDCl3
56.2 56.0
47.0 43.6
17.6
MeO
MeO
OMe
OMe
O
N
O
N
TFA
RUN-107
f1 (ppm)

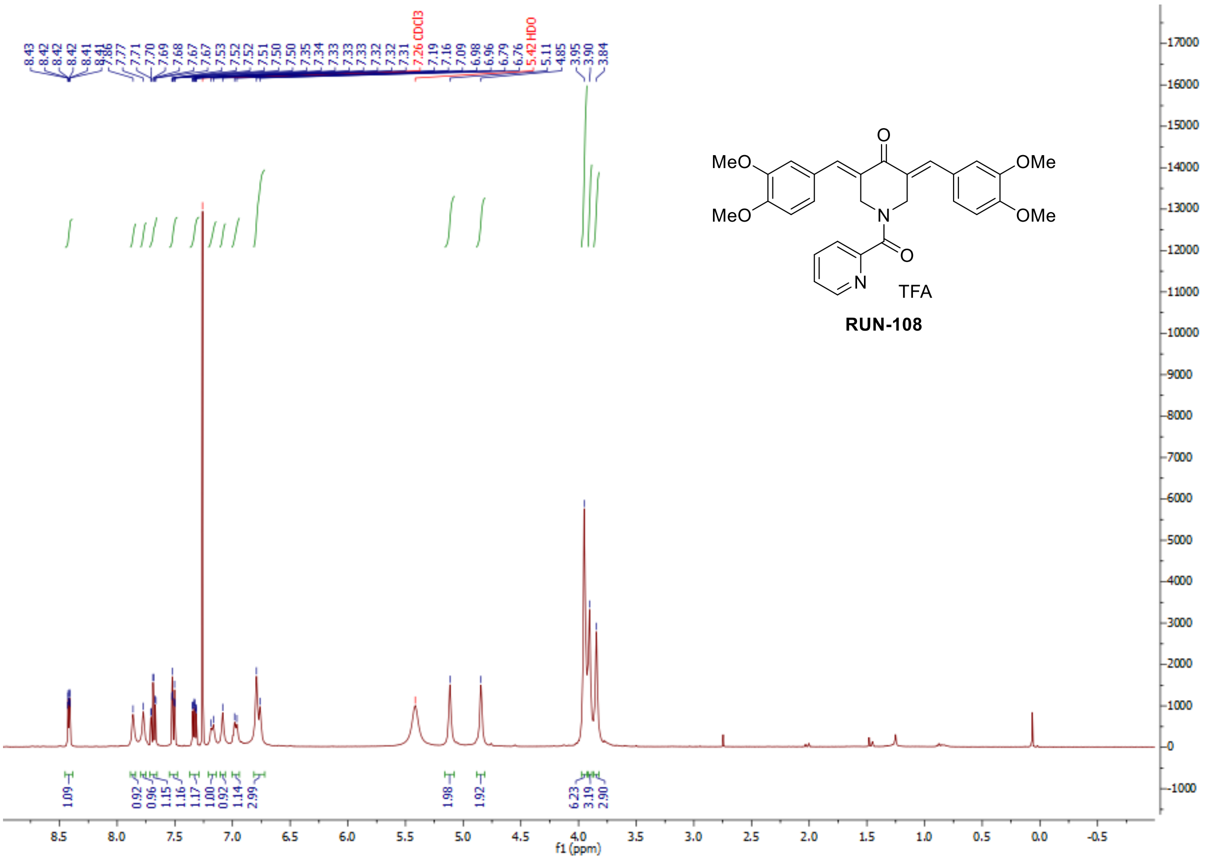

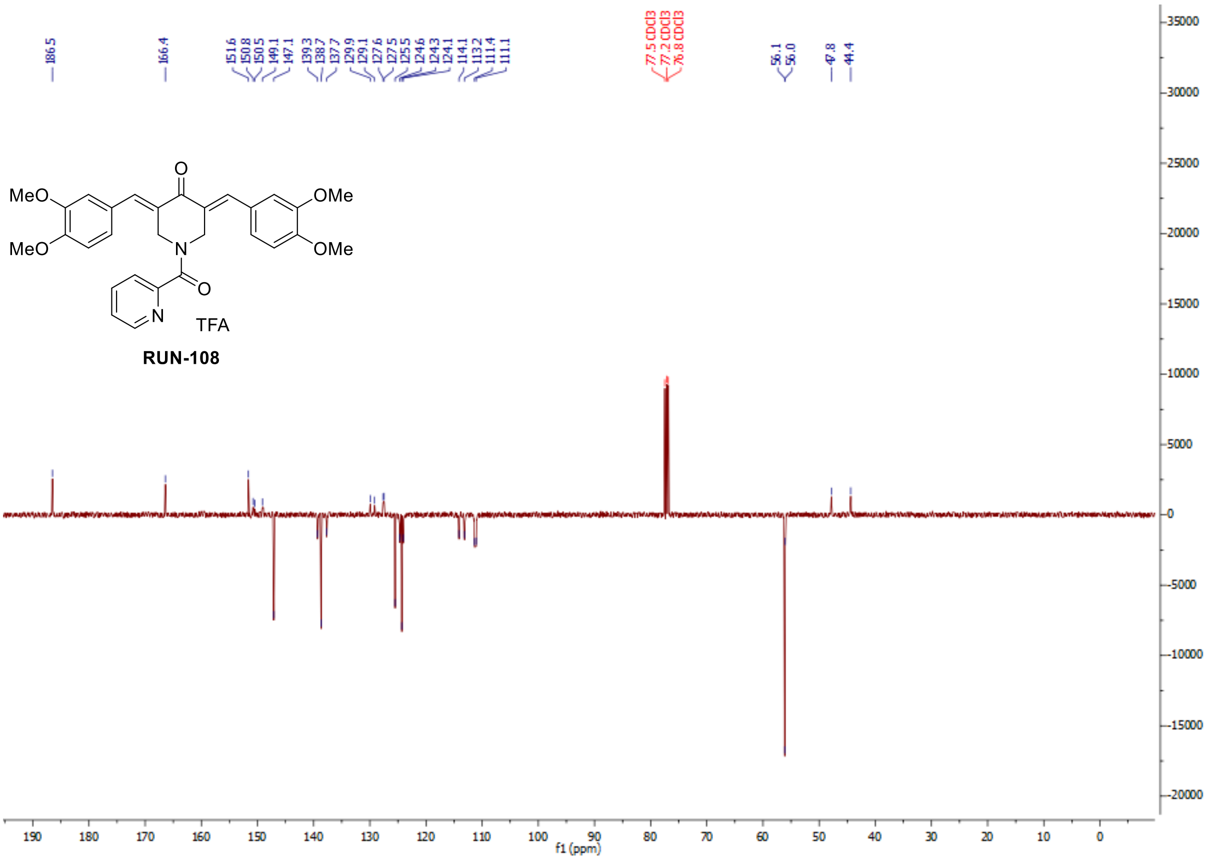

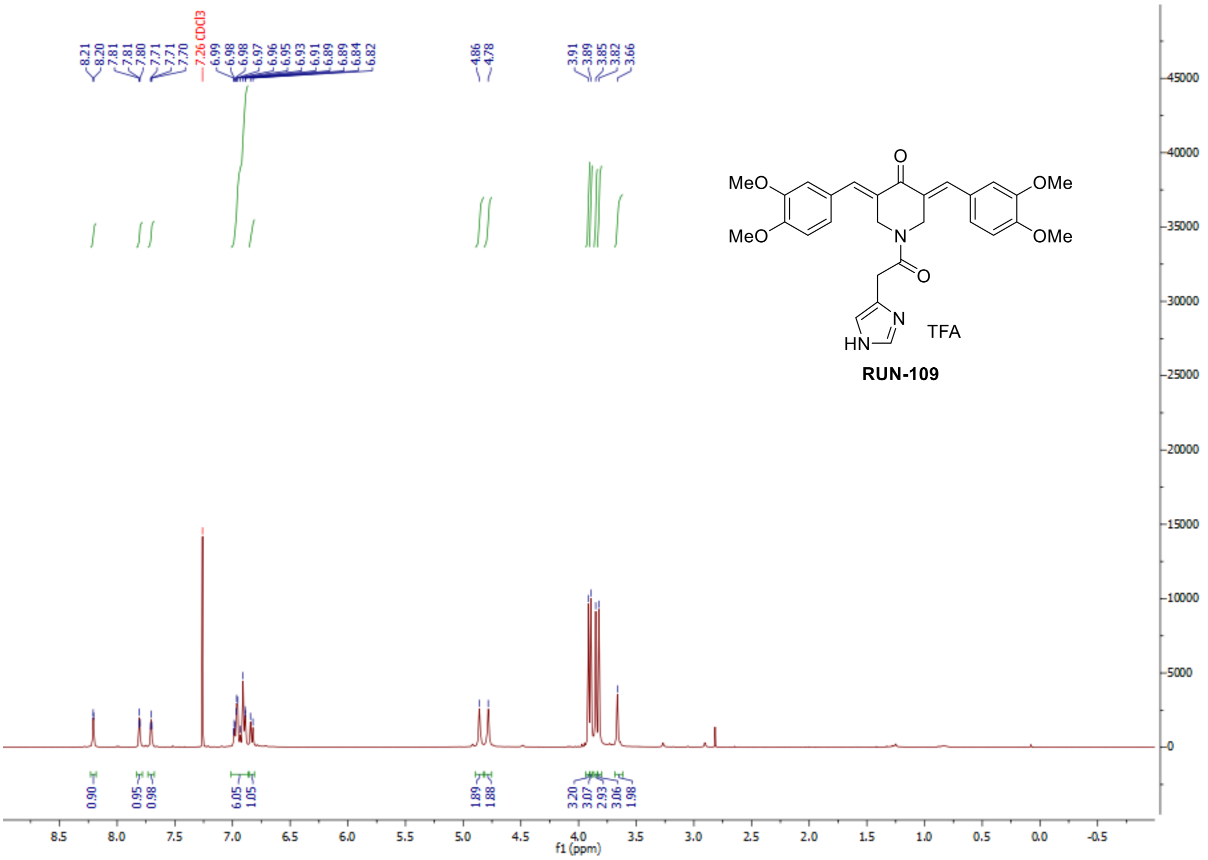

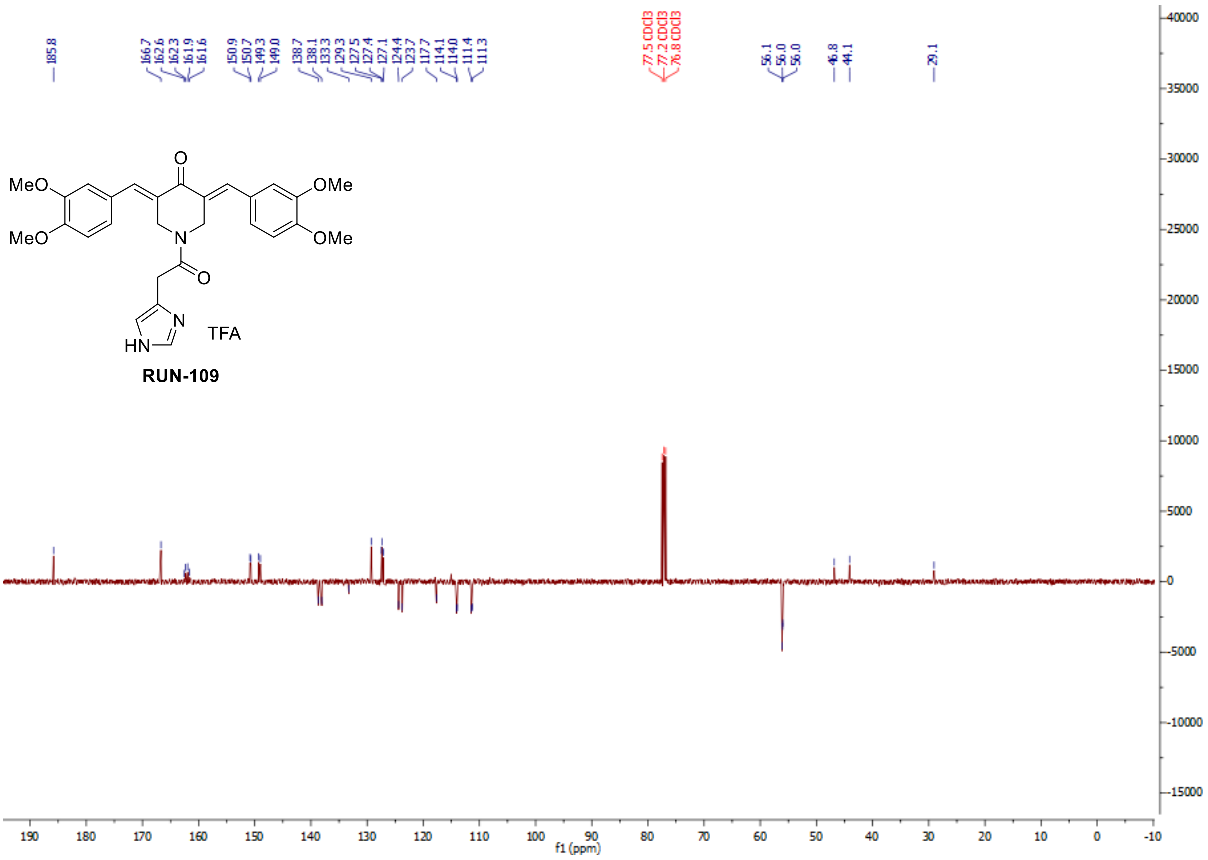

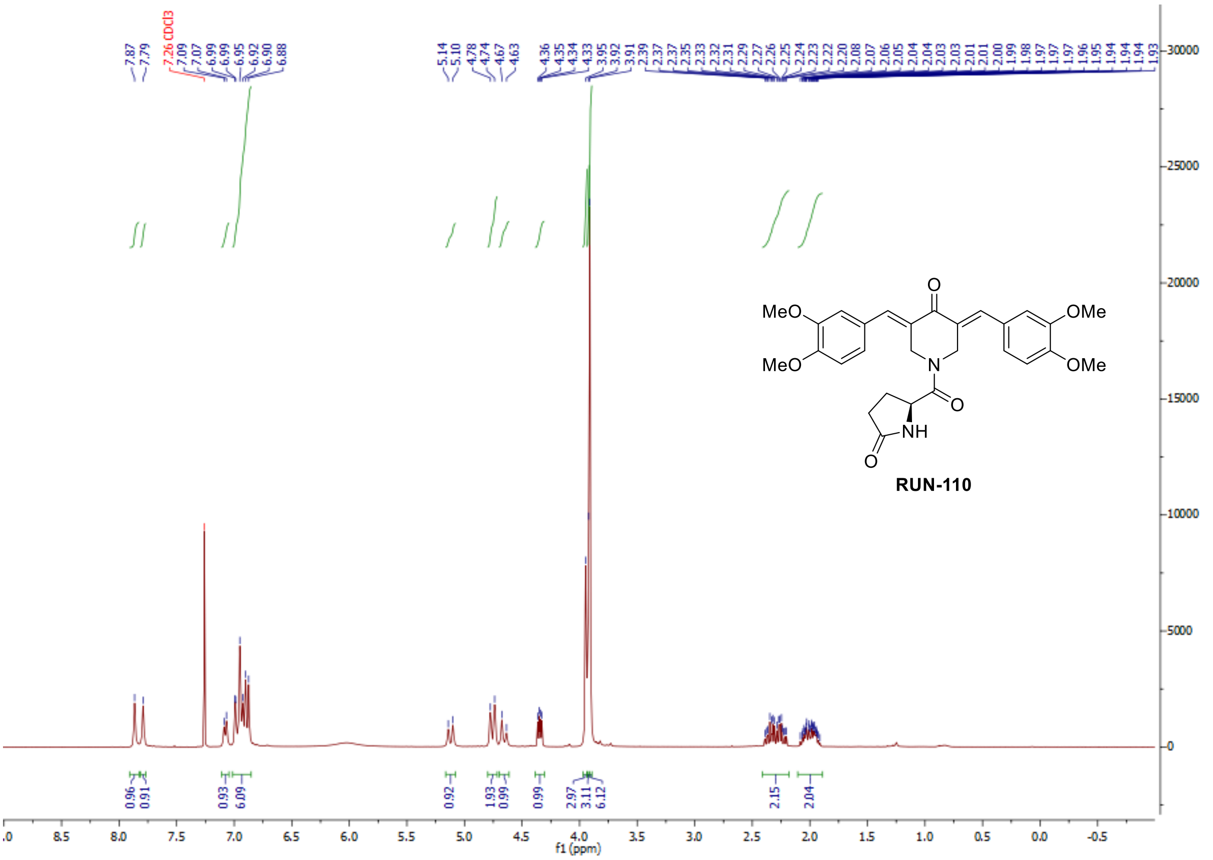

RUN-110

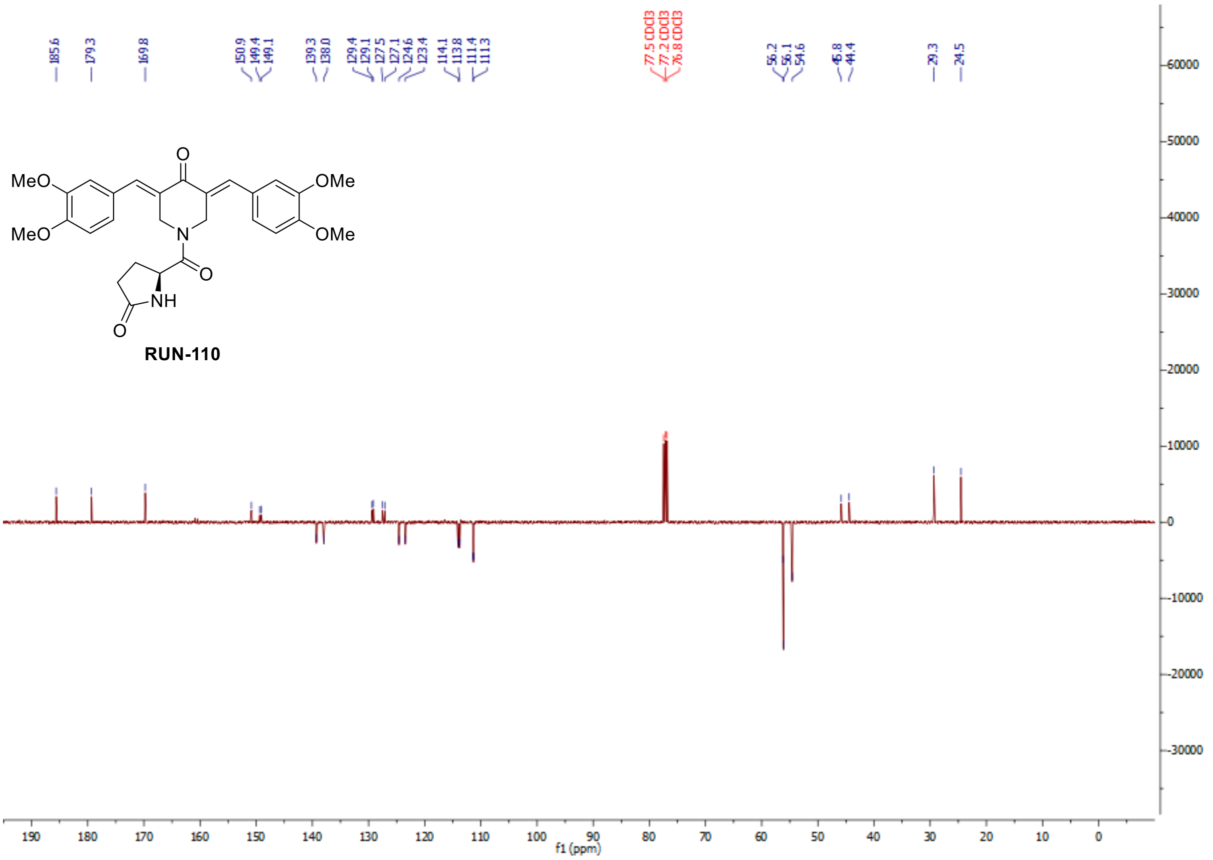

RUN-110

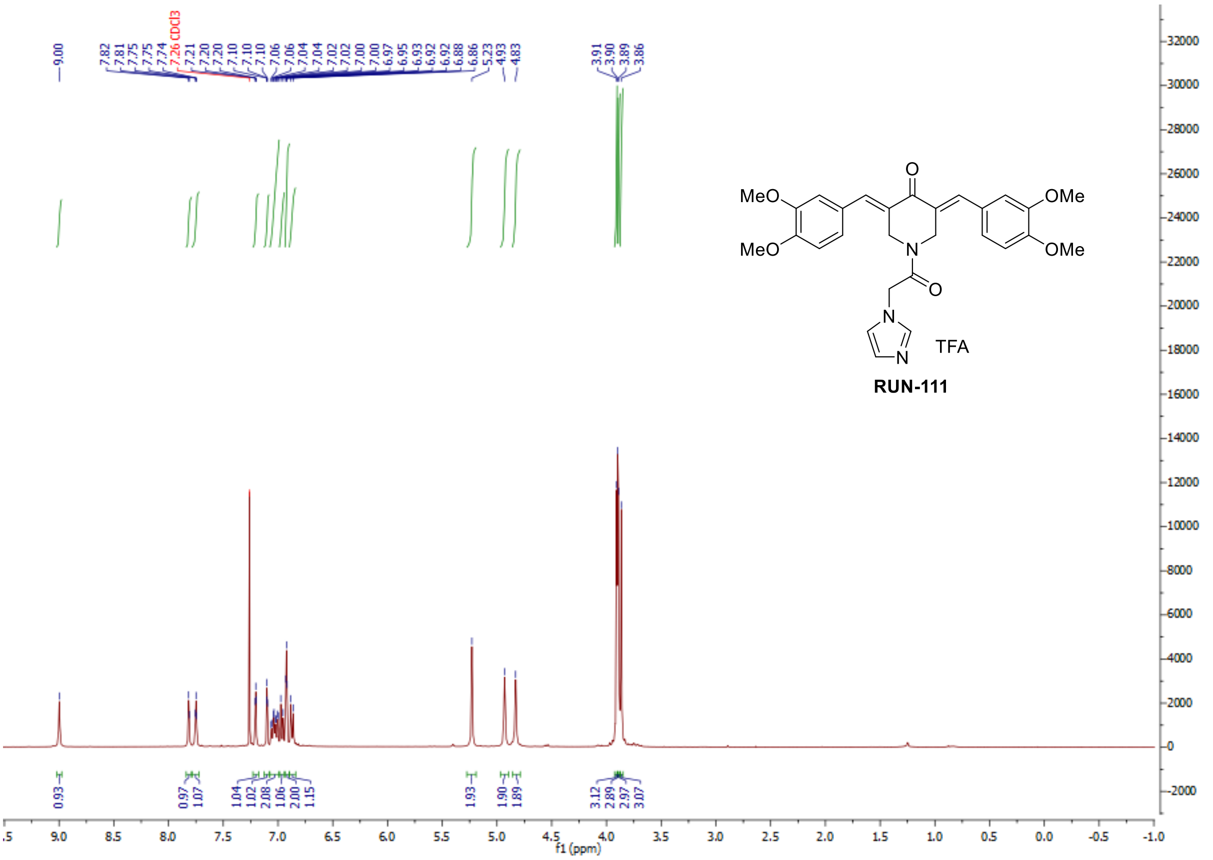

9.00
7.82
7.81
7.75
7.75
7.74
7.26 CDCl3
7.21
7.20
7.20
7.10
7.10
7.10
7.06
7.06
7.04
7.04
7.02
7.02
7.00
7.00
6.97
6.95
6.93
6.92
6.92
6.88
6.86
5.23
4.93
4.83
3.91
3.90
3.89
3.86
MeO
MeO
O
OMe
OMe
N
O
N
N
TFA
RUN-111
0.93
0.97
1.07
1.04
1.02
2.08
1.06
1.06
2.00
1.15
1.93
1.90
1.89
3.12
2.89
2.97
3.07
f1 (ppm)

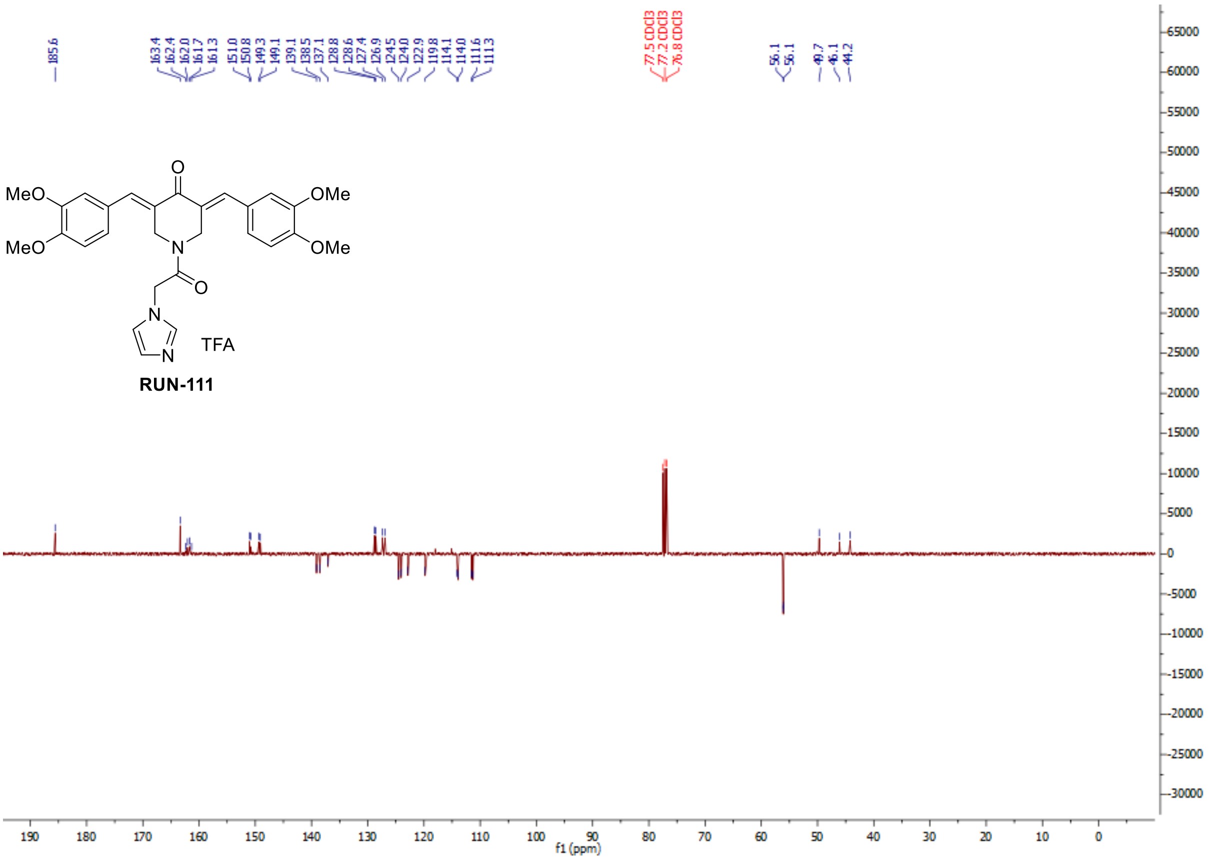

185.6
163.4
162.4
162.0
161.7
161.3
151.0
150.8
149.3
149.1
139.1
138.5
137.1
128.8
128.6
127.4
126.9
124.5
124.0
122.9
119.8
114.1
114.0
111.6
111.3
77.5 CDCl3
77.2 CDCl3
76.8 CDCl3
56.1
56.1
49.7
46.1
44.2
MeO
MeO
O
OMe
OMe
N
O
N
N
TFA
RUN-111
f1 (ppm)

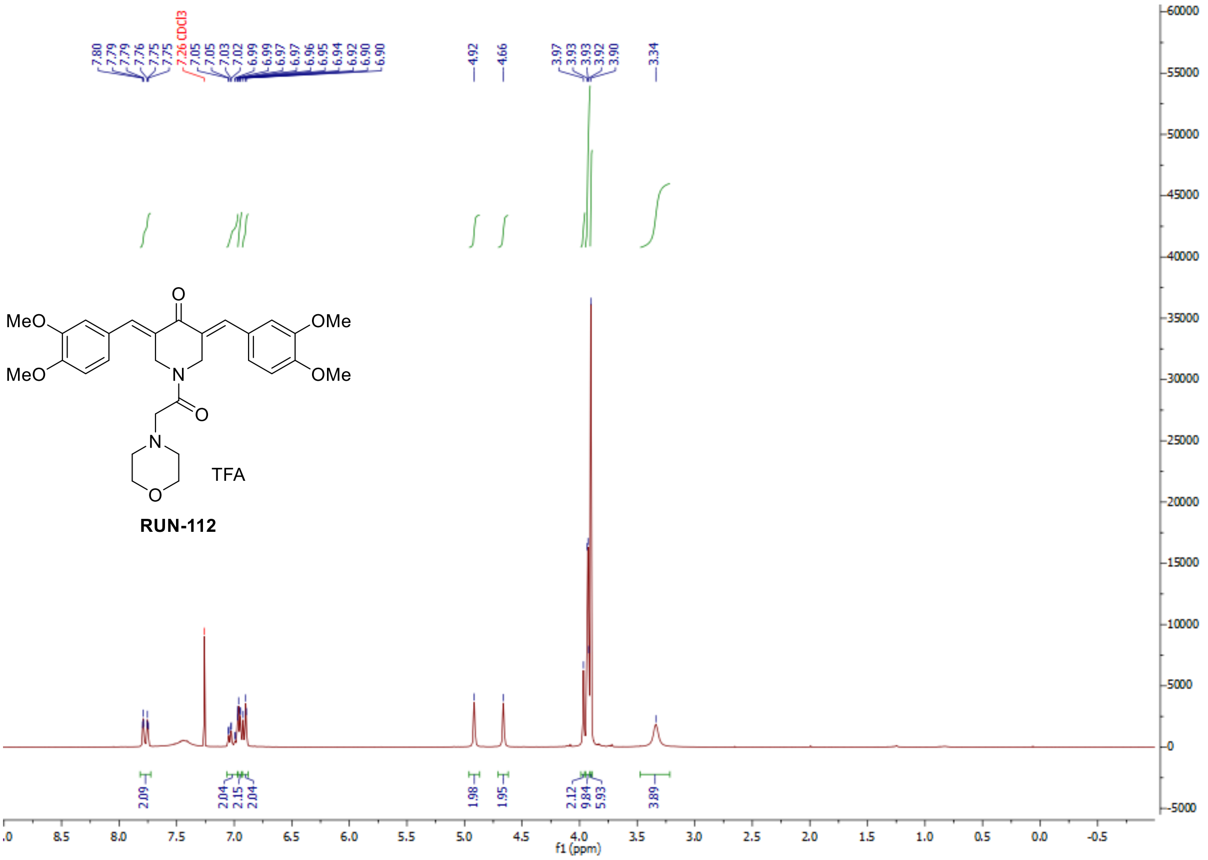

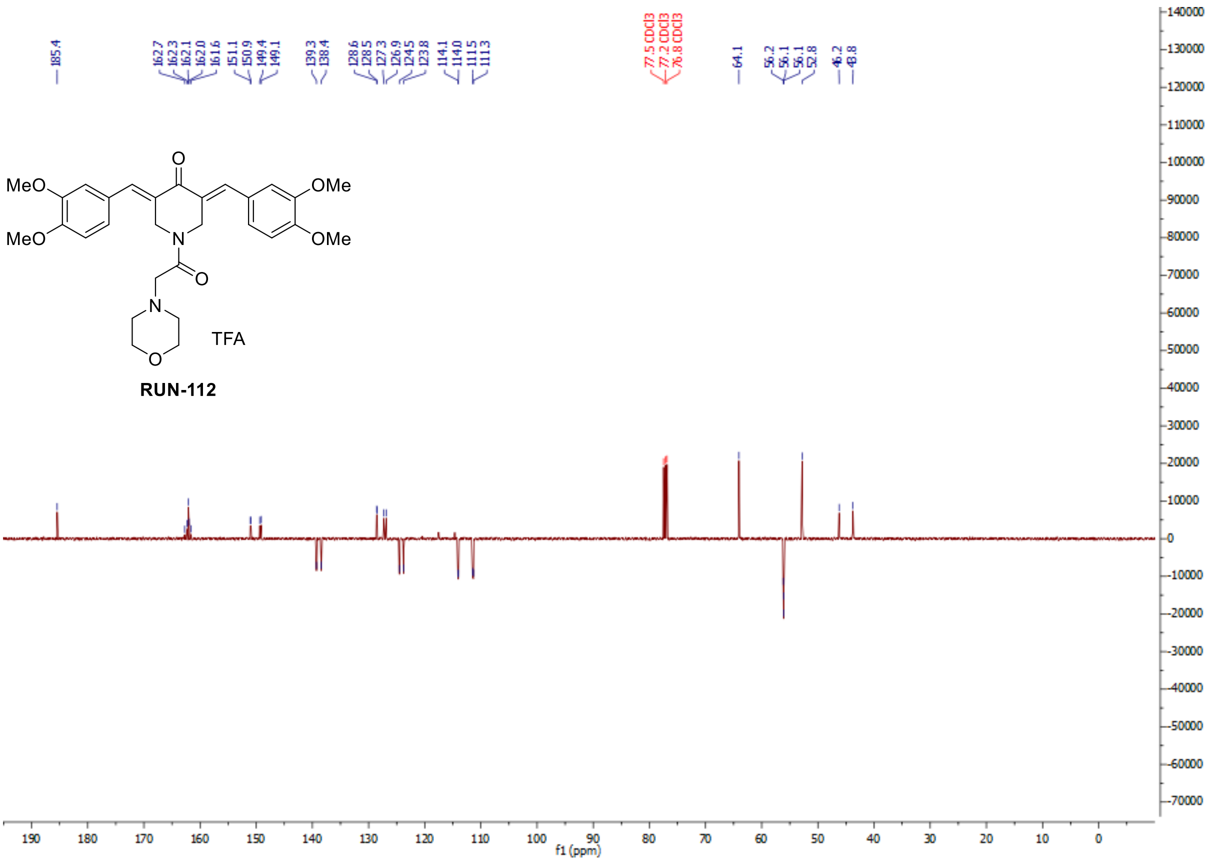

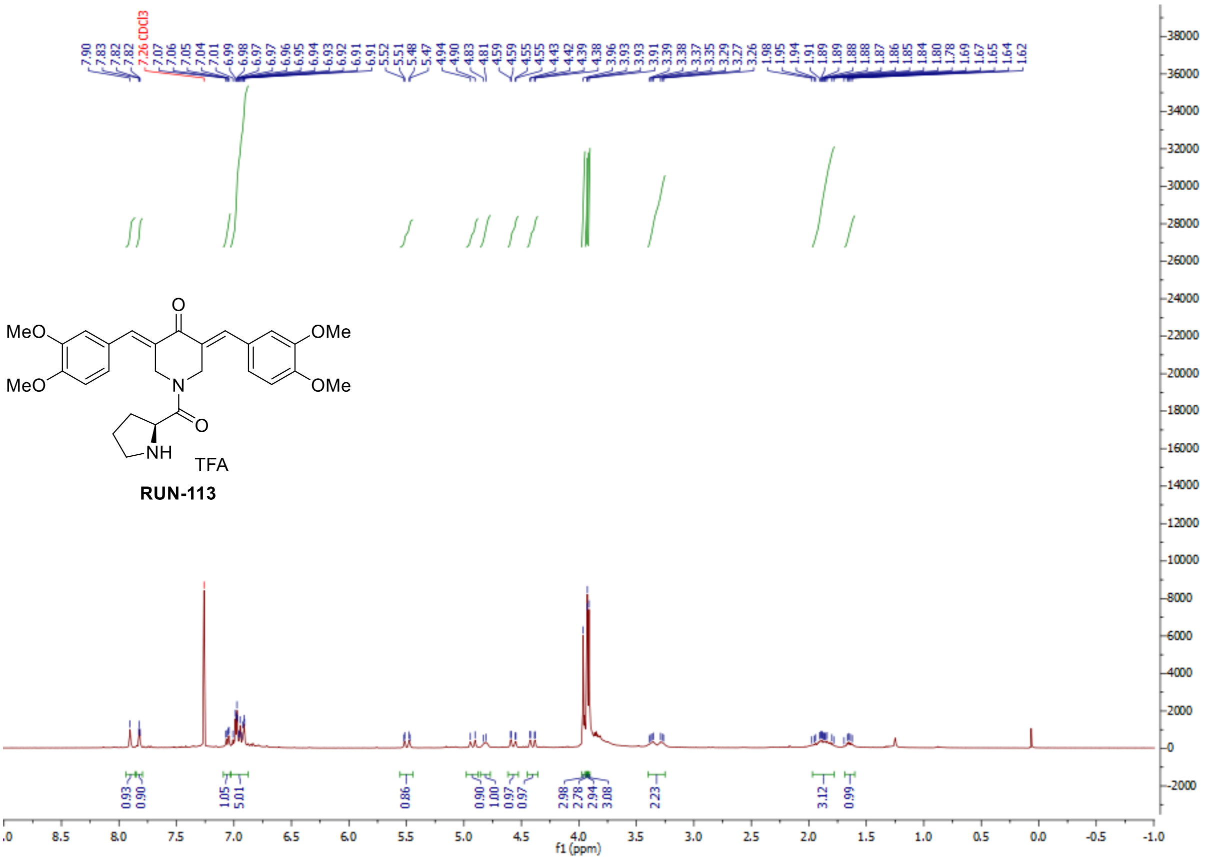

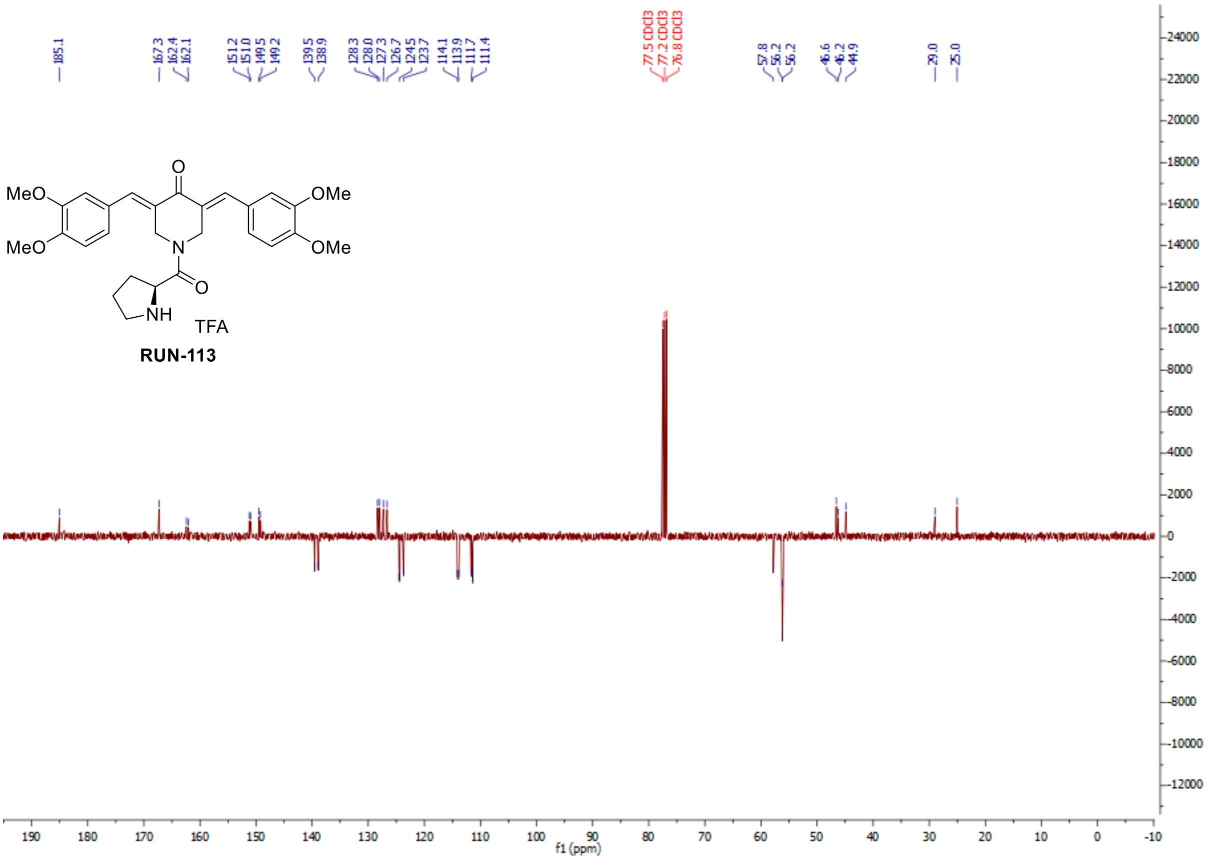

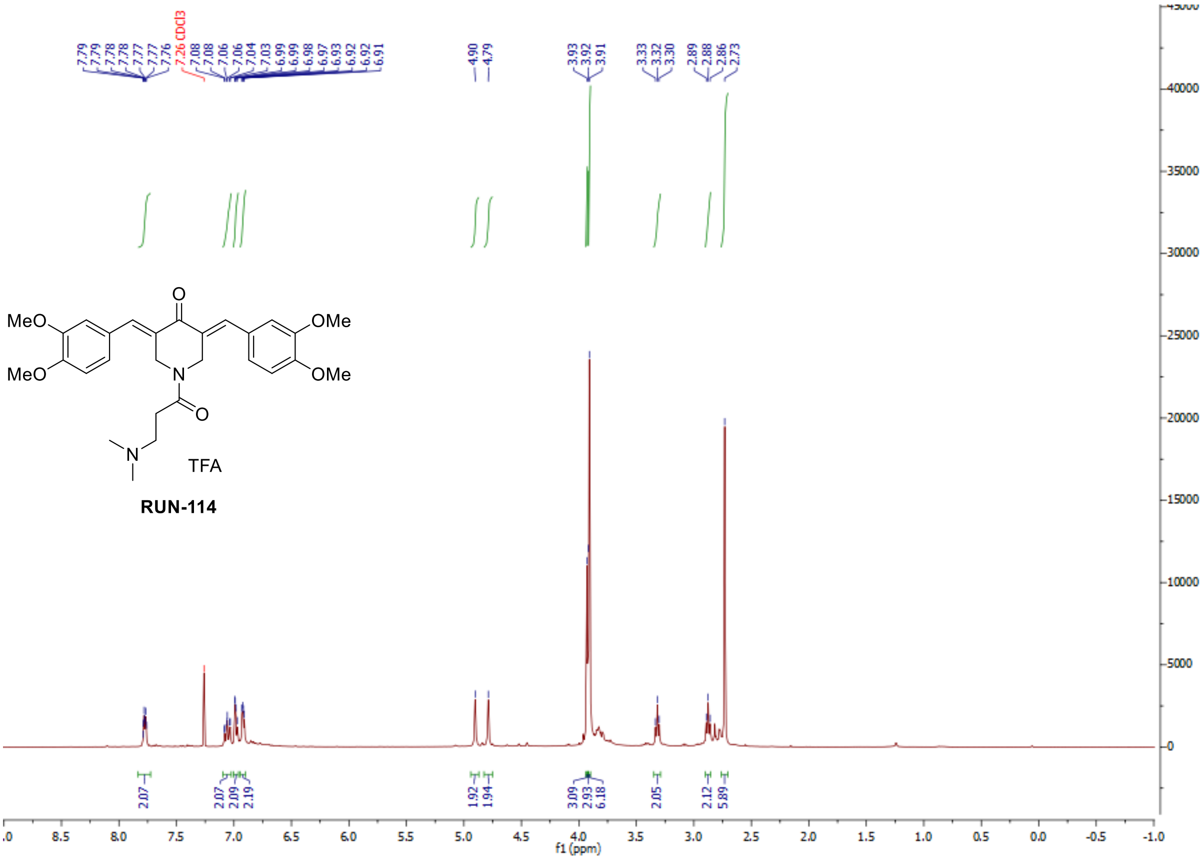

7.79
7.79
7.78
7.78
7.77
7.77
7.76
7.26 CDCl3
7.08
7.08
7.06
7.06
7.04
7.04
7.03
6.99
6.99
6.98
6.97
6.93
6.92
6.92
6.91
4.90
4.79
3.93
3.92
3.91
3.33
3.32
3.30
2.89
2.88
2.86
2.73
MeO
MeO
O
OMe
OMe
N
O
N
TFA
RUN-114
2.07
2.07
2.09
2.19
1.92
1.94
3.09
2.93
6.18
2.05
2.12
5.89
f1 (ppm)

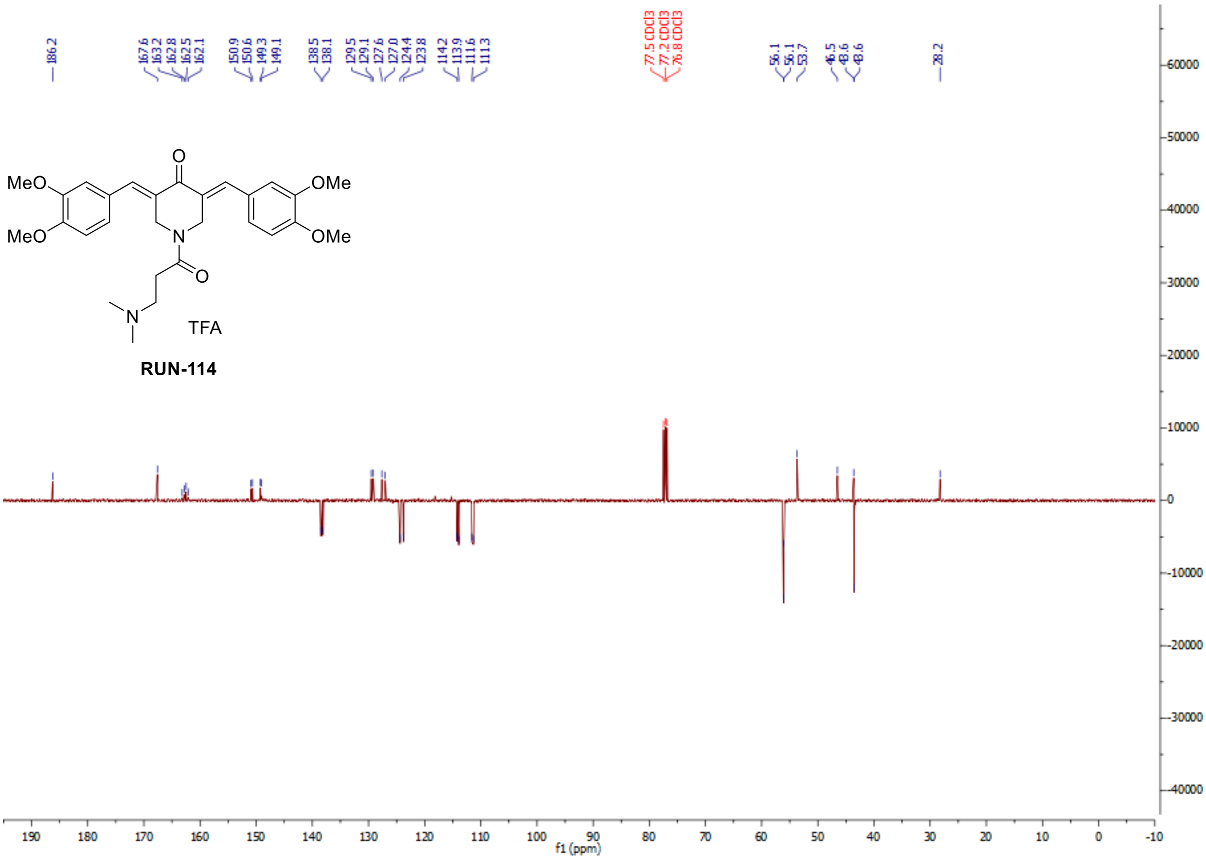

186.2
167.6
163.2
162.8
162.5
162.1
150.9
150.6
149.3
149.1
138.5
138.1
129.5
129.1
127.6
127.0
124.4
123.8
114.2
113.9
111.6
111.3
77.5 CDCl3
77.2 CDCl3
76.8 CDCl3
56.1
56.1
53.7
46.5
43.6
43.6
28.2
MeO
MeO
O
OMe
OMe
N
O
N
TFA
RUN-114
f1 (ppm)

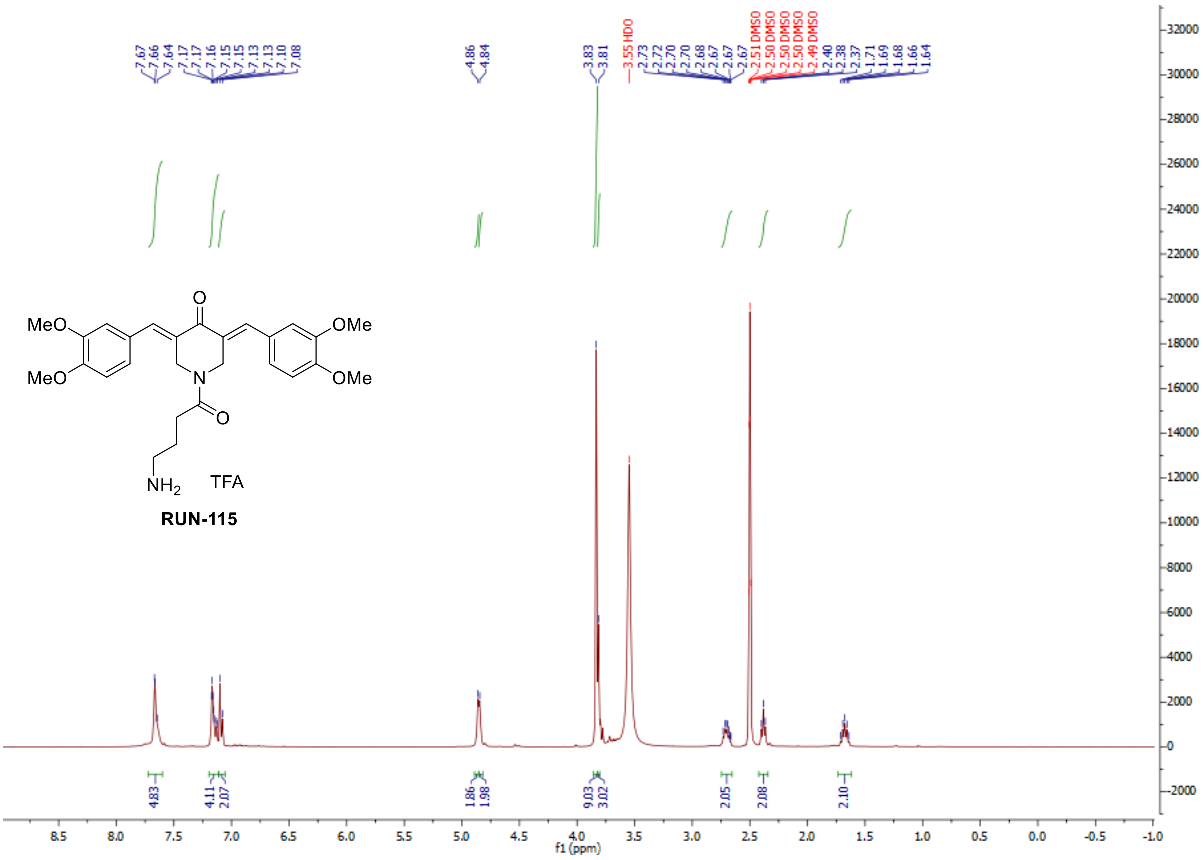
7.67 7.66 7.64 7.17 7.17 7.16 7.15 7.15 7.13 7.13 7.10 7.08
4.86 4.84
3.83 3.81
3.55 HDO
2.73 2.72 2.70 2.70 2.68 2.67 2.67 2.67
2.51 DMSO 2.50 DMSO 2.50 DMSO 2.50 DMSO 2.49 DMSO
2.40 2.38 2.37 1.71 1.69 1.68 1.66 1.64
MeO
MeO
O
OMe
OMe
N
O
NH2
TFA
RUN-115
4.83 4.11 2.07 1.86 1.98 9.03 3.02 2.05 2.08 2.10
f1 (ppm)

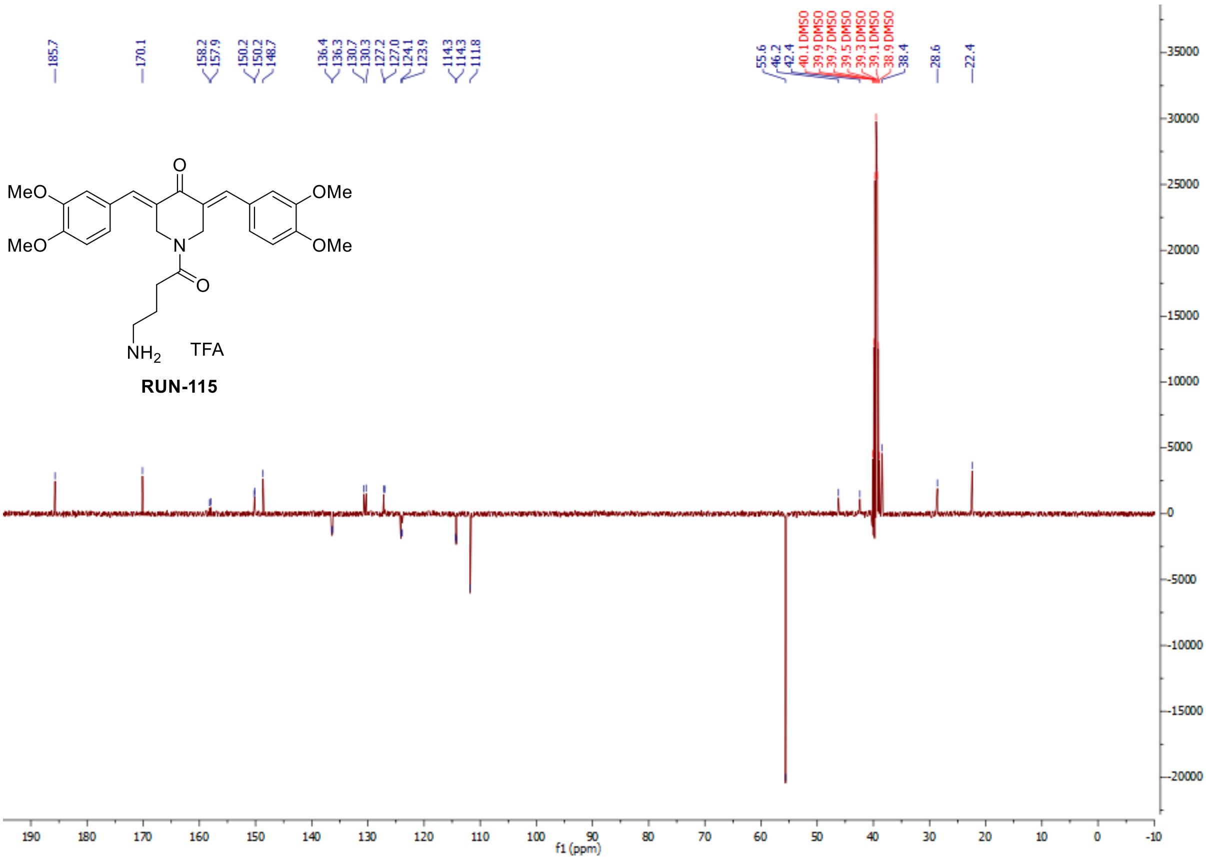
185.7
170.1
158.2 157.9
150.2 150.2 148.7
136.4 136.3 130.7 130.3 127.2 127.0 124.1 123.9
114.3 114.3 111.8
55.6 46.2 42.4
40.1 DMSO 39.9 DMSO 39.7 DMSO 39.5 DMSO 39.3 DMSO 39.1 DMSO 38.9 DMSO
38.4
28.6
22.4
MeO
MeO
O
OMe
OMe
N
O
NH2
TFA
RUN-115
f1 (ppm)

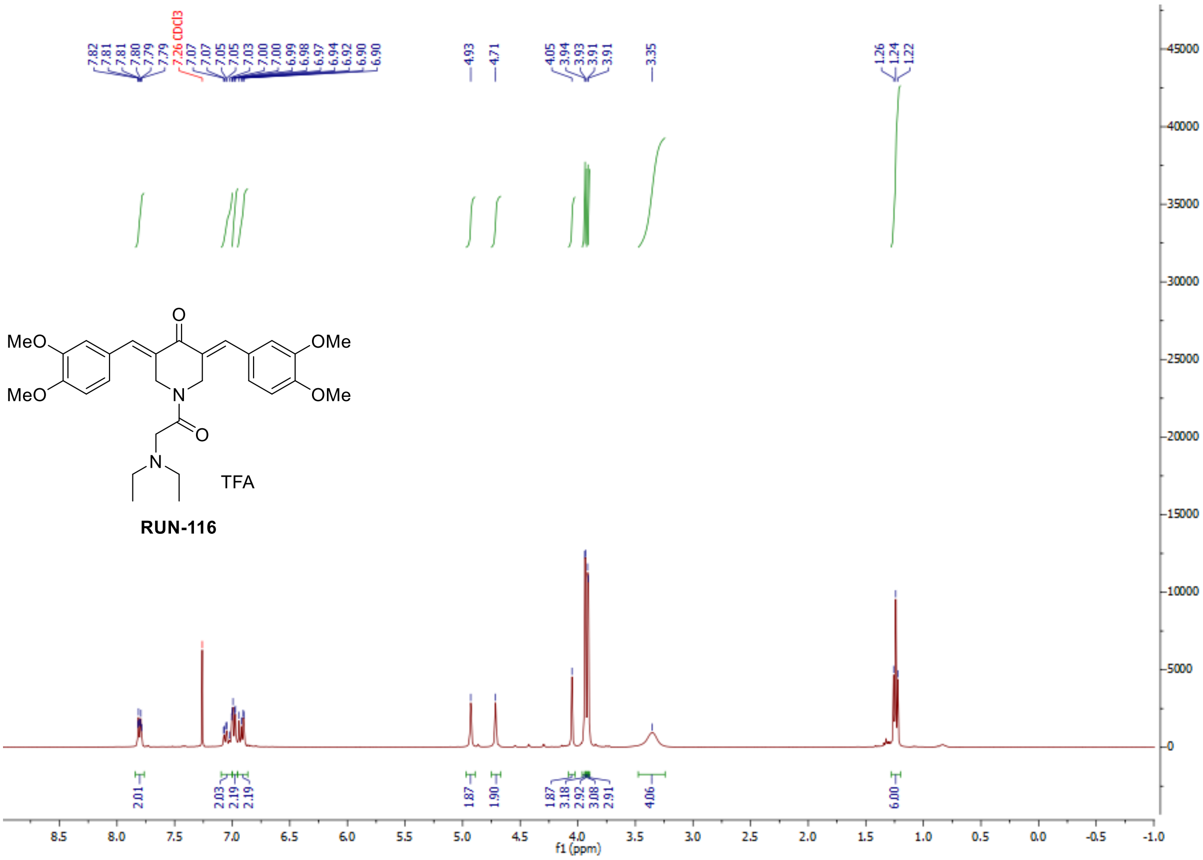
MeO
MeO
OMe
OMe
O
N
O
N
TFA
RUN-116
7.82
7.81
7.81
7.80
7.79
7.79
7.26 CDCl3
7.07
7.07
7.05
7.05
7.03
7.00
7.00
6.99
6.98
6.97
6.94
6.92
6.90
6.90
4.93
4.71
4.05
3.94
3.93
3.93
3.91
3.91
3.35
1.26
1.24
1.22
2.01
2.03
2.19
2.19
1.87
1.90
1.87
3.18
2.92
3.08
2.91
4.06
6.00
f1 (ppm)

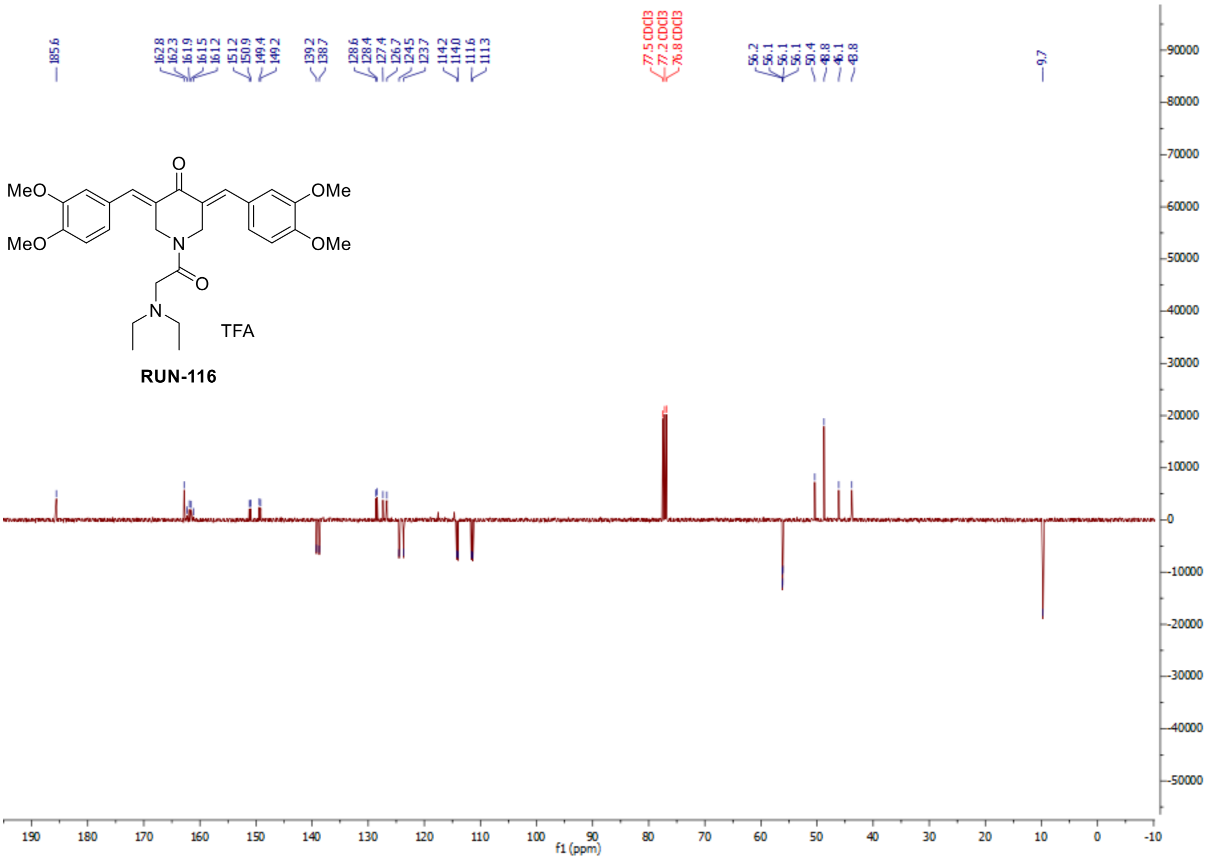
MeO
MeO
OMe
OMe
O
N
O
N
TFA
RUN-116
185.6
162.8
162.3
161.9
161.5
161.2
151.2
150.9
149.4
149.2
139.2
138.7
128.6
128.4
127.4
126.7
124.5
123.7
114.2
114.0
111.6
111.3
77.5 CDCl3
77.2 CDCl3
76.8 CDCl3
56.2
56.1
56.1
56.1
50.4
48.8
46.1
43.8
9.7
f1 (ppm)

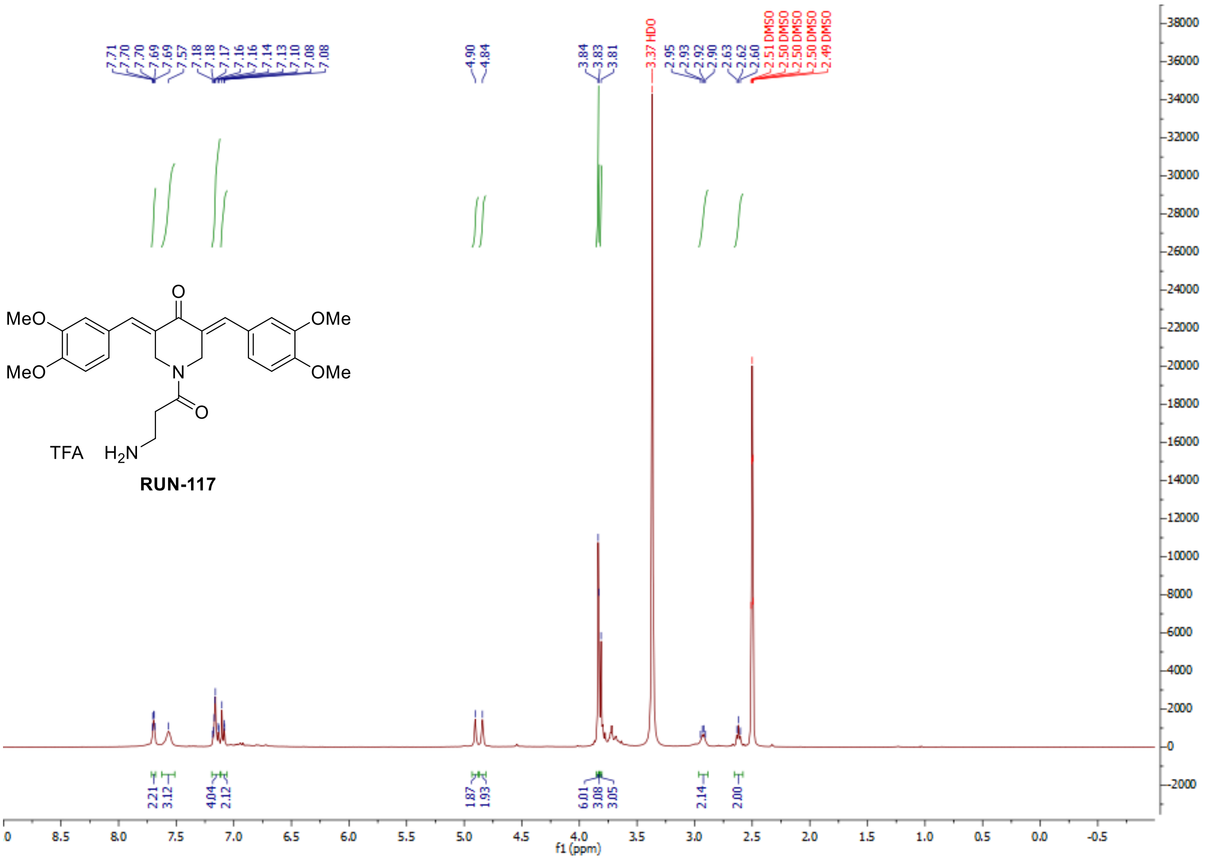
MeO
MeO
O
OMe
OMe
N
O
TFA
H2N
RUN-117
7.71 7.70 7.70 7.69 7.69 7.57 7.18 7.18 7.17 7.16 7.16 7.14 7.13 7.10 7.08 7.08
4.90 4.84
3.84 3.83 3.81
3.37 HDO
2.95 2.93 2.92 2.90 2.63 2.62 2.60
2.51 DMSO 2.50 DMSO 2.50 DMSO 2.50 DMSO 2.49 DMSO
2.21 3.12 4.04 2.12 1.87 1.93 6.01 3.08 3.05 2.14 2.00
f1 (ppm)

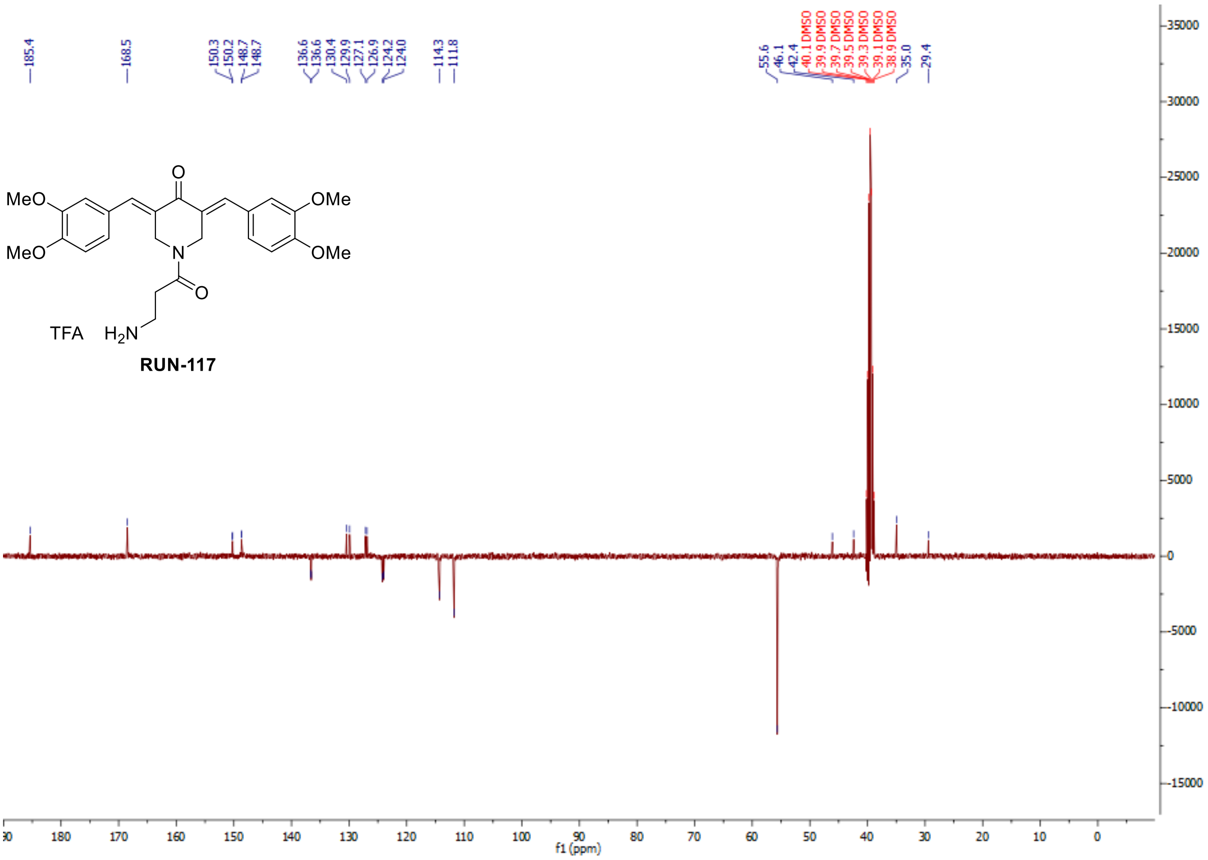
MeO
MeO
O
OMe
OMe
N
O
TFA
H2N
RUN-117
185.4
168.5
150.3 150.2 148.7 148.7
136.6 136.6 130.4 129.9 127.1 126.9 124.2 124.0
114.3 111.8
55.6 46.1 42.4
40.1 DMSO 39.9 DMSO 39.7 DMSO 39.5 DMSO 39.3 DMSO 39.1 DMSO 38.9 DMSO
35.0 29.4
f1 (ppm)

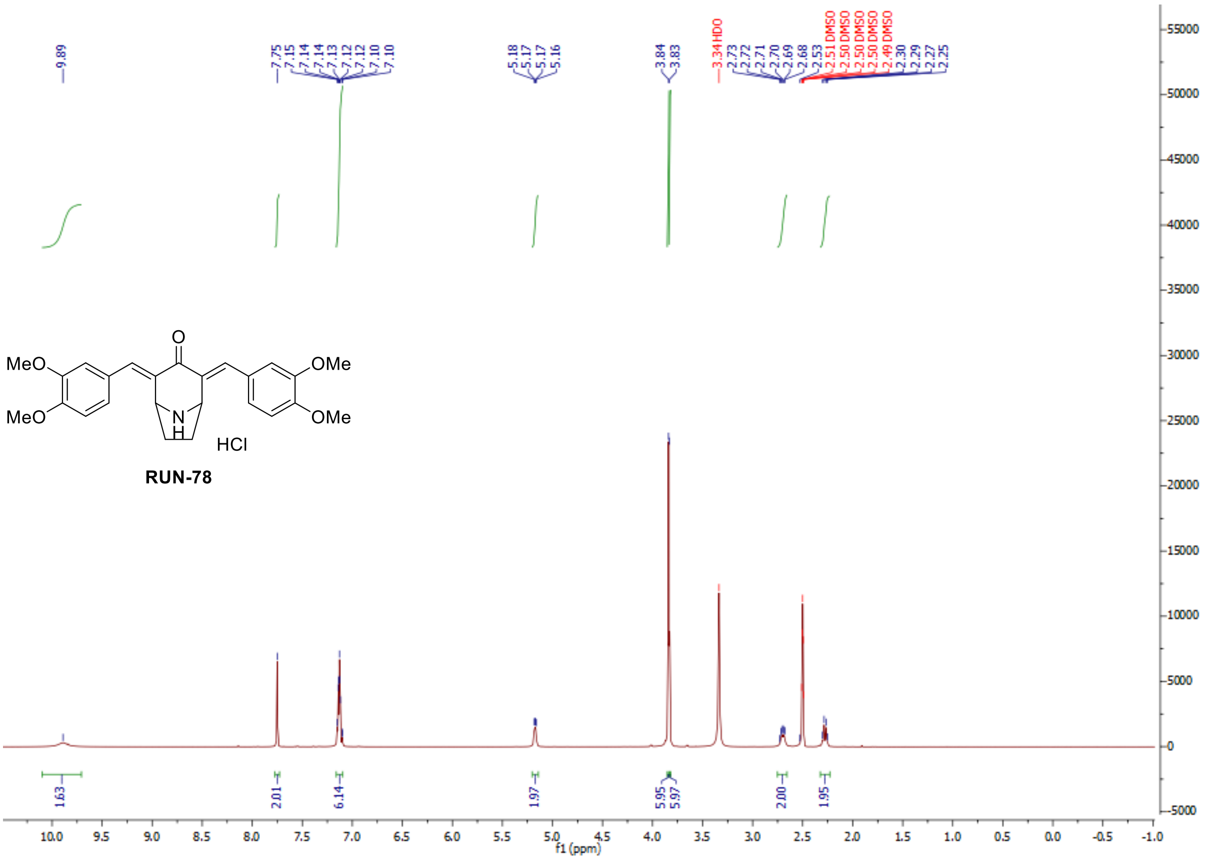

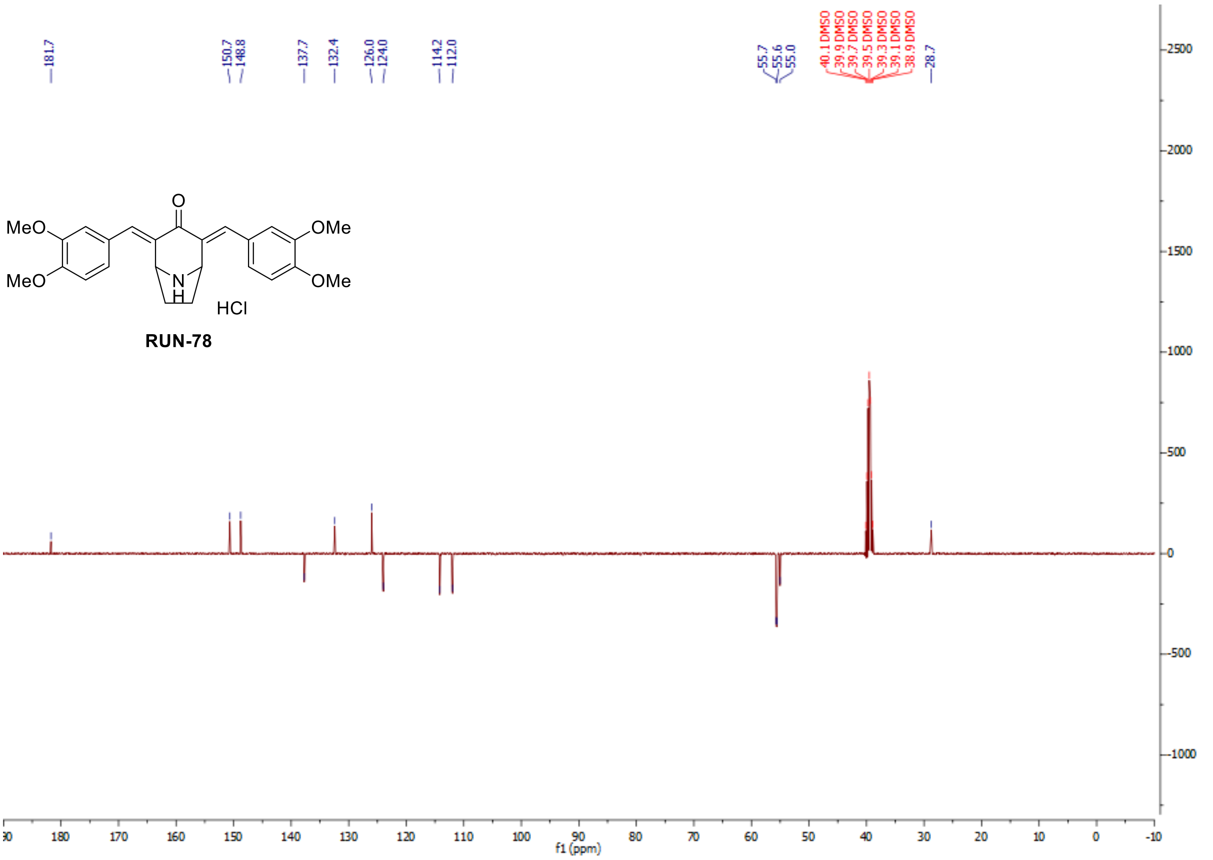

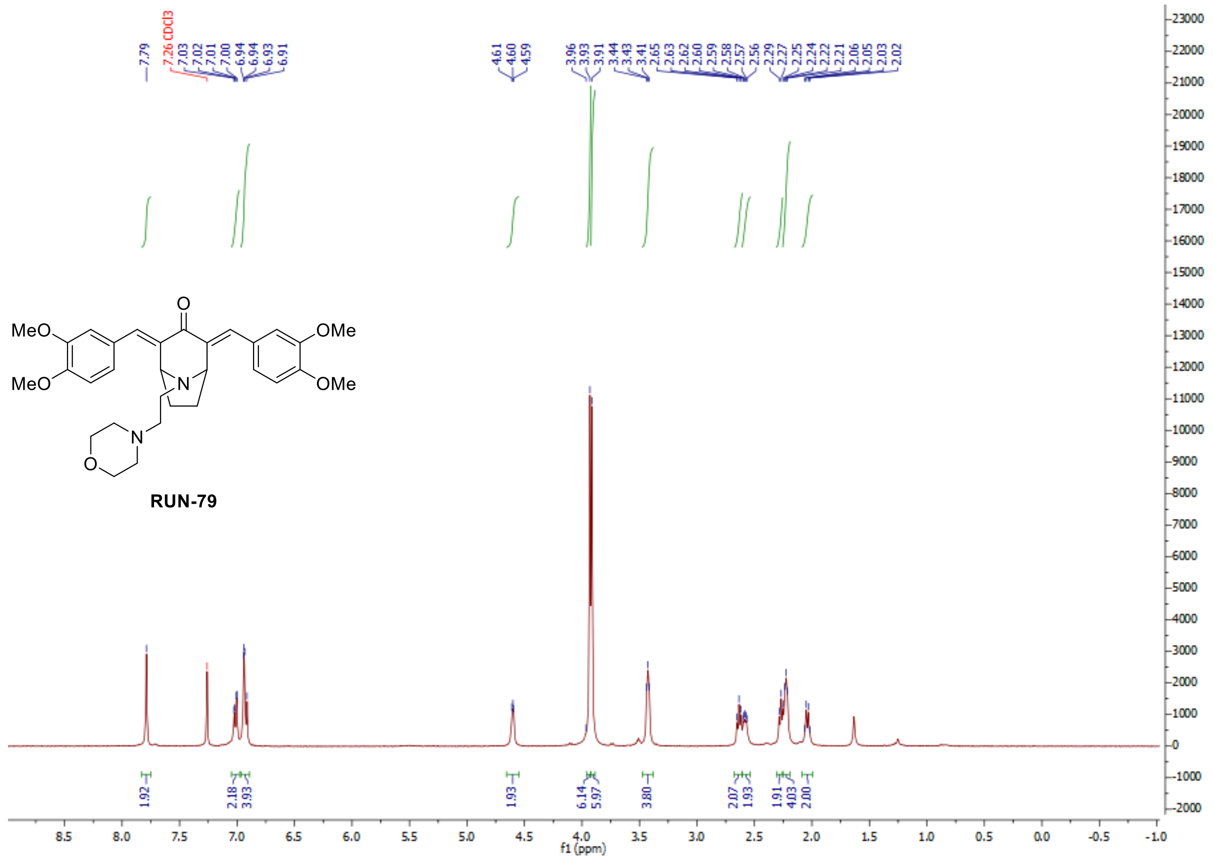

RUN-79

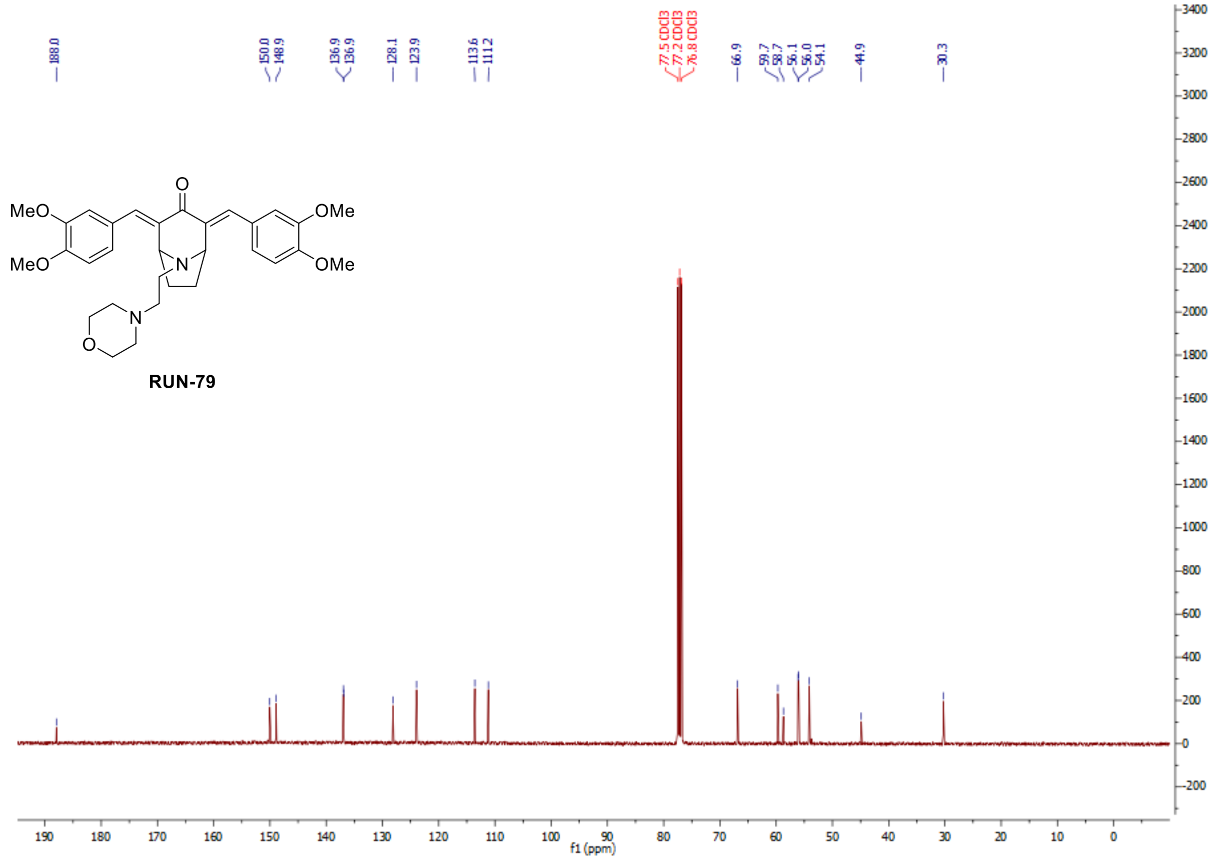

RUN-79

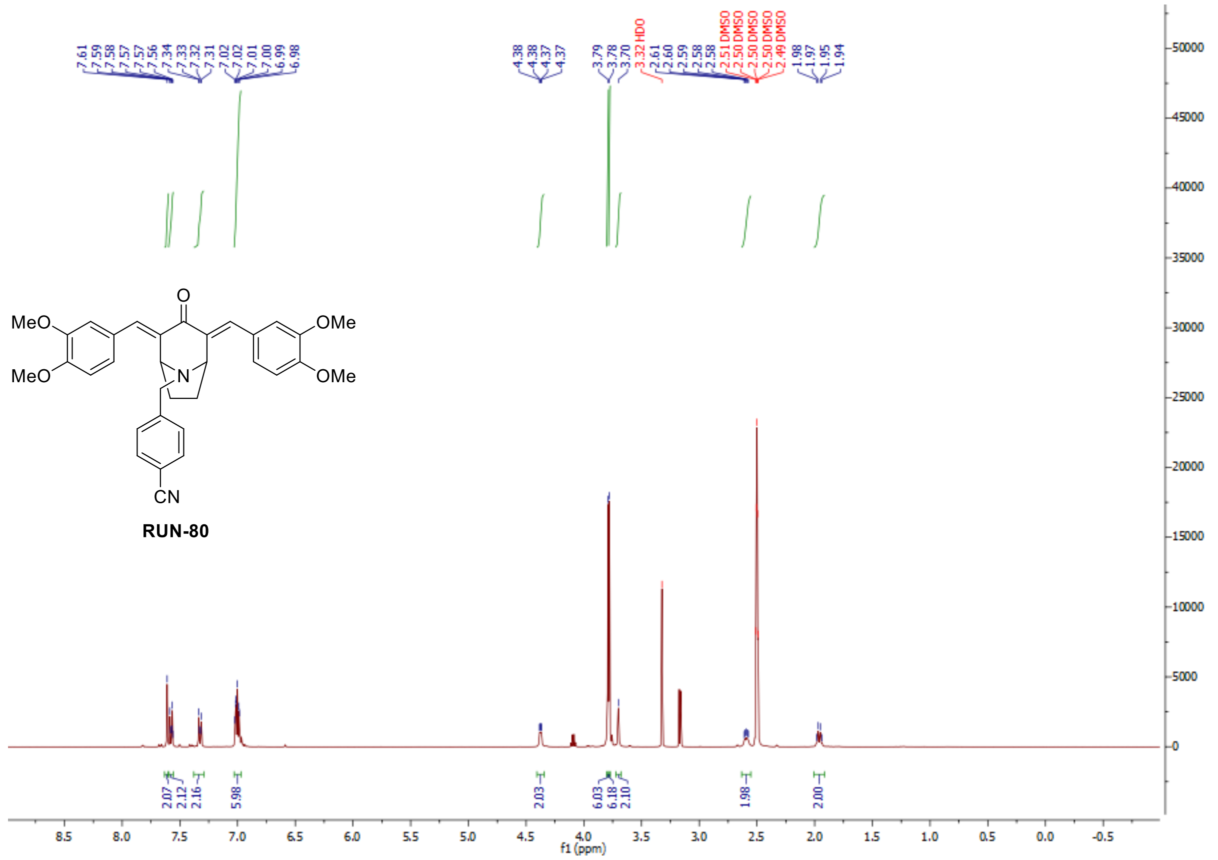

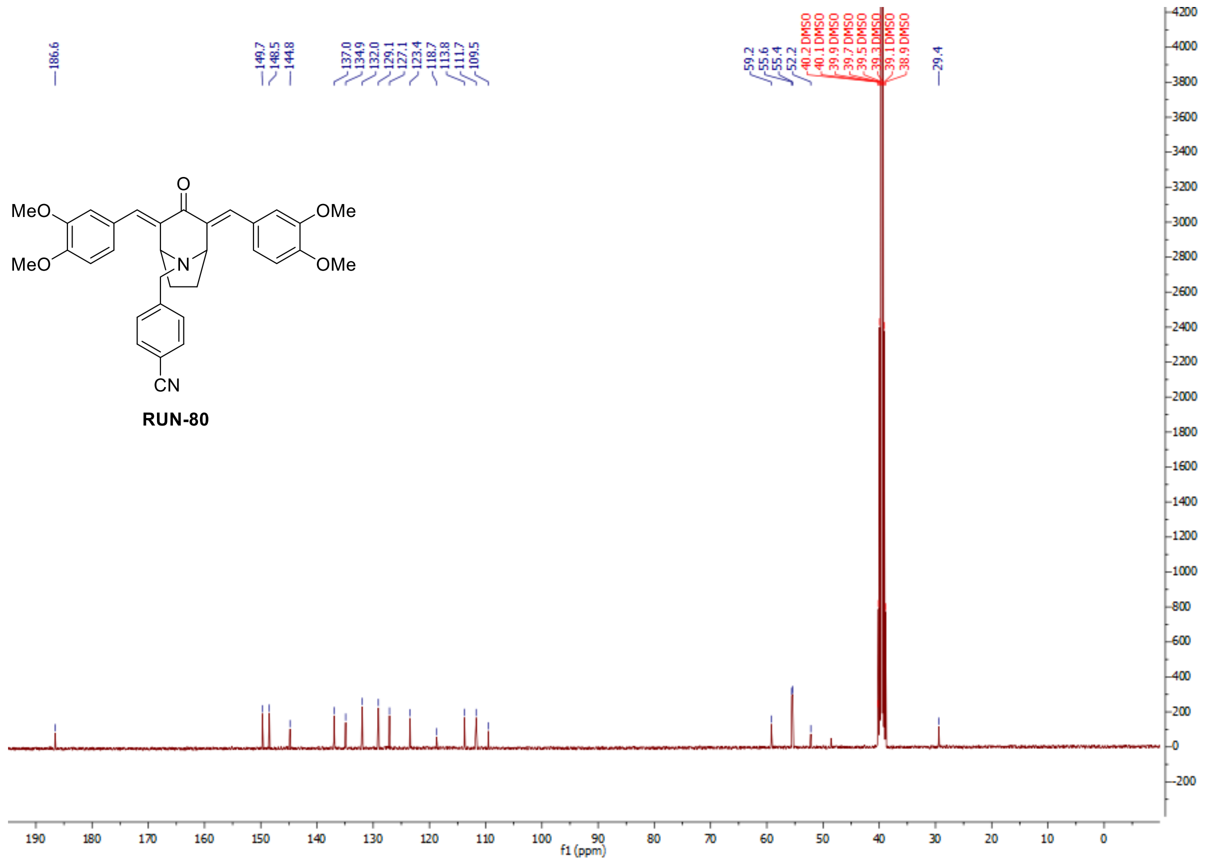

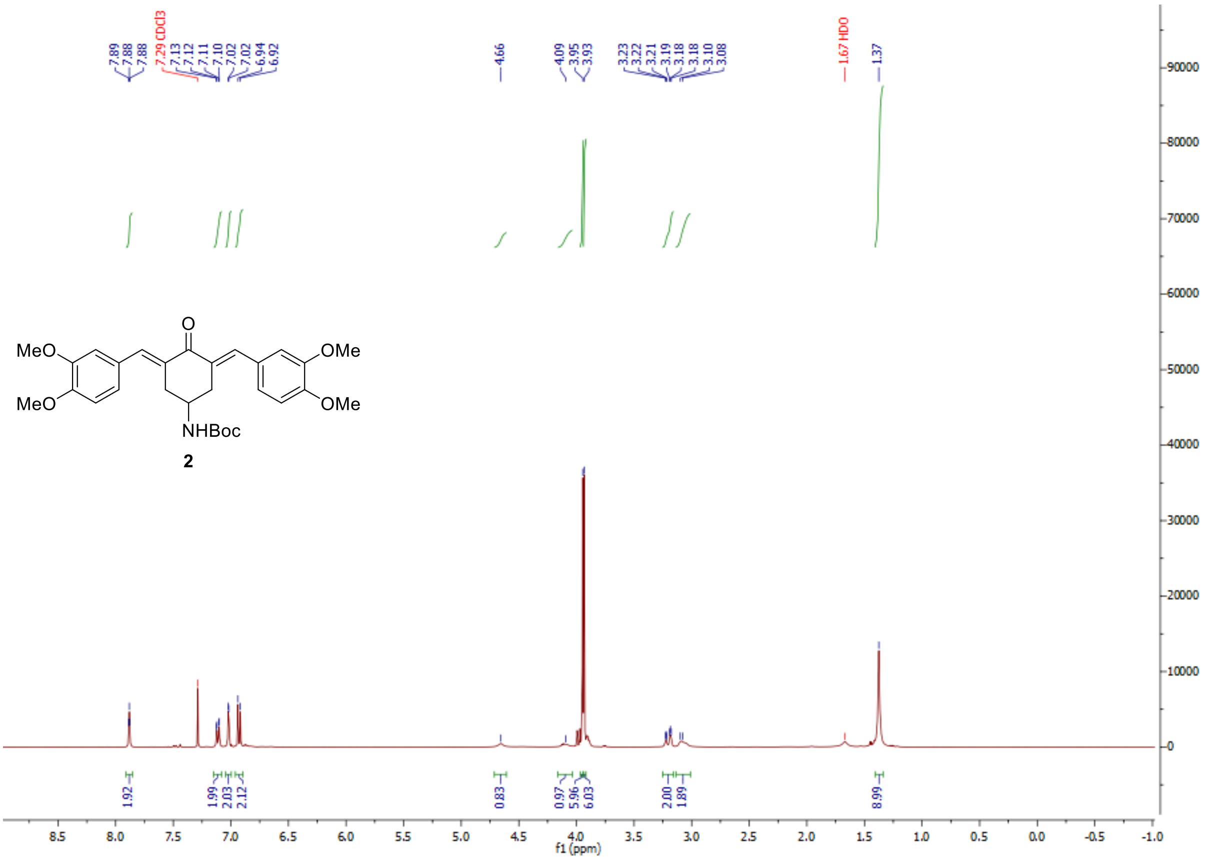
7.89
7.88
7.88
7.29 CDCl3
7.13
7.12
7.11
7.10
7.02
7.02
6.94
6.92
4.66
4.09
3.95
3.93
3.23
3.22
3.21
3.19
3.18
3.18
3.10
3.08
1.67 HDO
1.37
MeO
MeO
O
OMe
OMe
NHBoc
2
1.92
1.99
2.03
2.12
0.83
0.97
5.96
6.03
2.00
1.89
8.99
f1 (ppm)

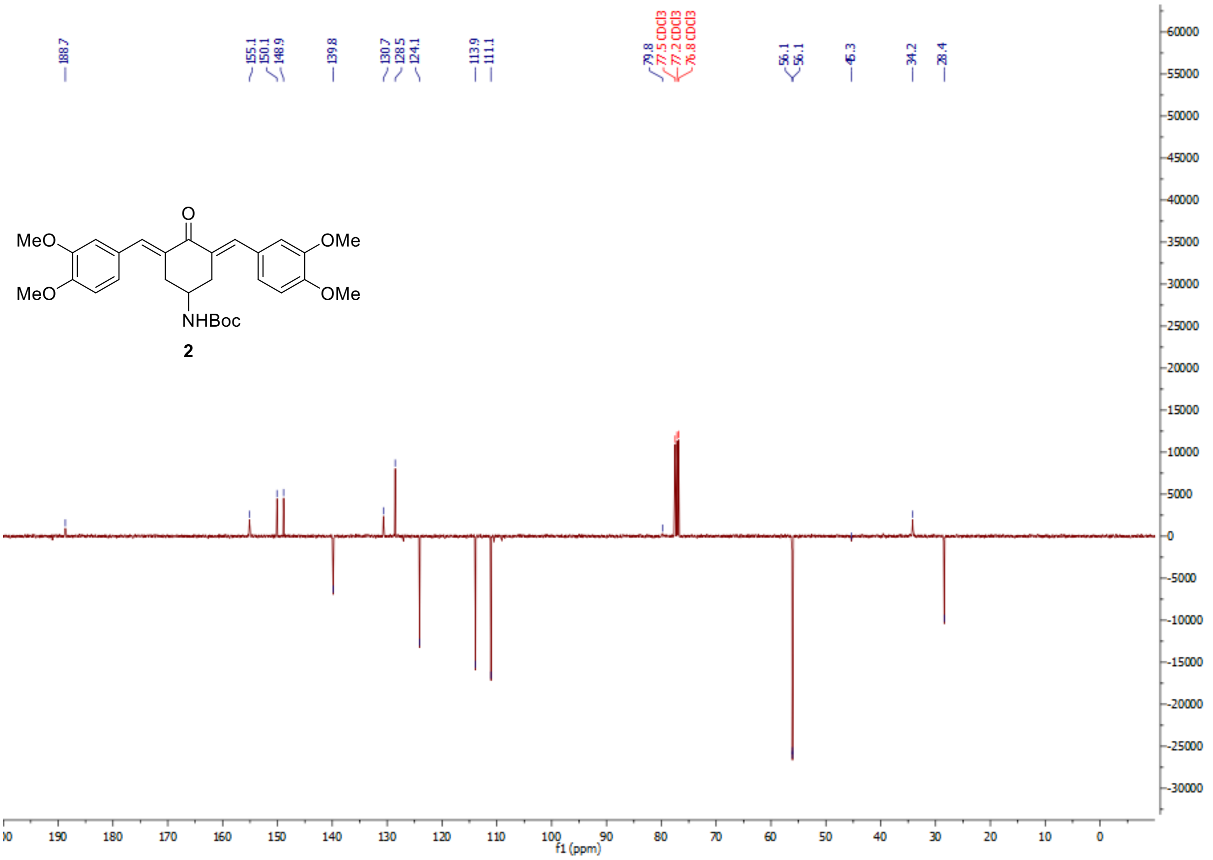
188.7
155.1
150.1
148.9
139.8
130.7
128.5
124.1
113.9
111.1
79.8
77.5 CDCl3
77.2 CDCl3
76.8 CDCl3
56.1
56.1
45.3
34.2
28.4
MeO
MeO
O
OMe
OMe
NHBoc
2
f1 (ppm)

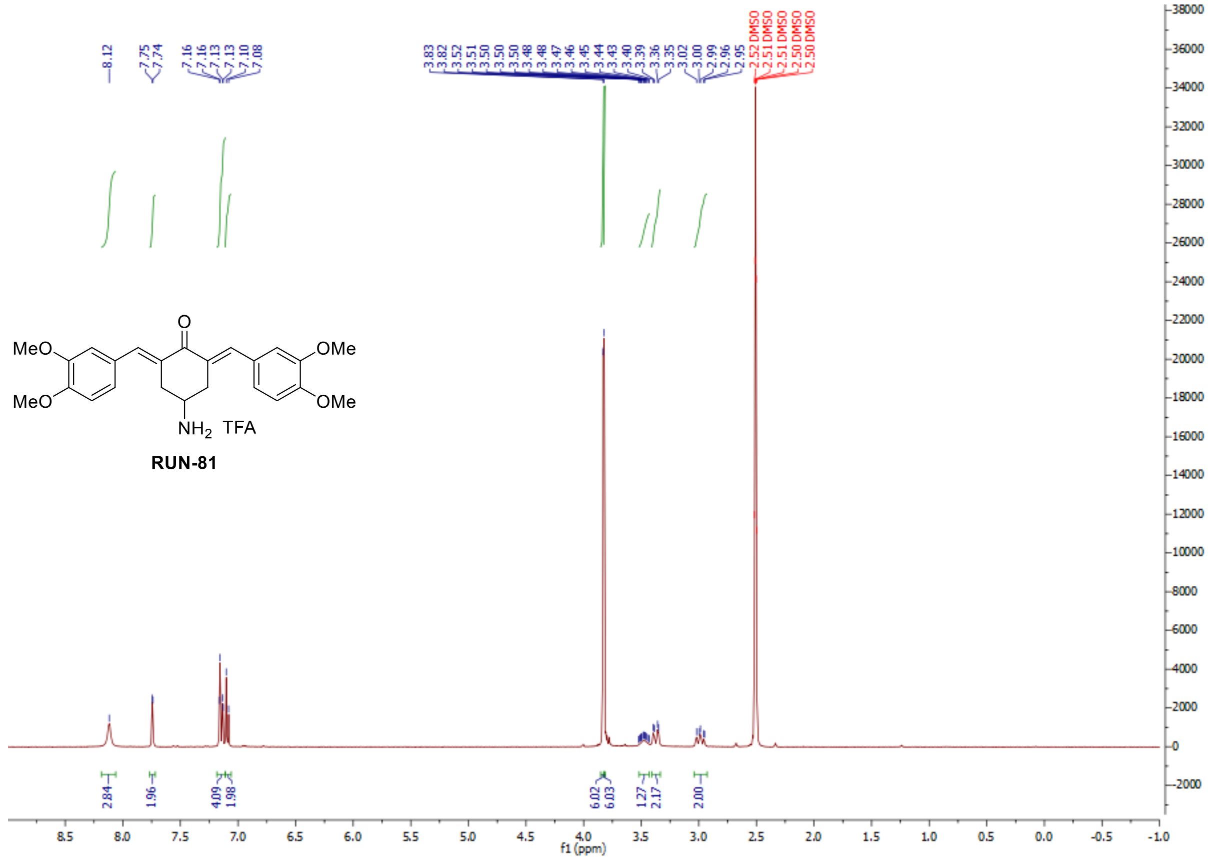
MeO
MeO
O
OMe
OMe
NH2 TFA
RUN-81
f1 (ppm)

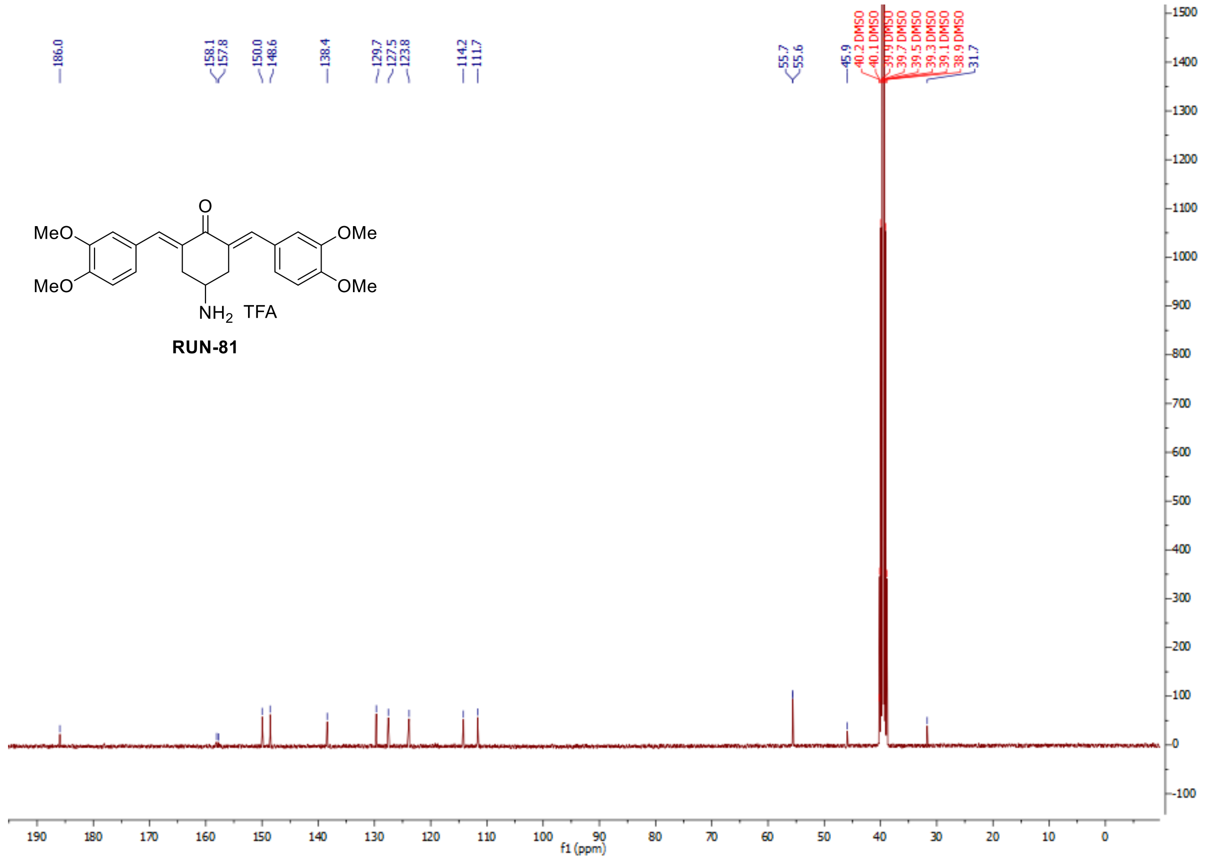
MeO
MeO
O
OMe
OMe
NH2 TFA
RUN-81
f1 (ppm)

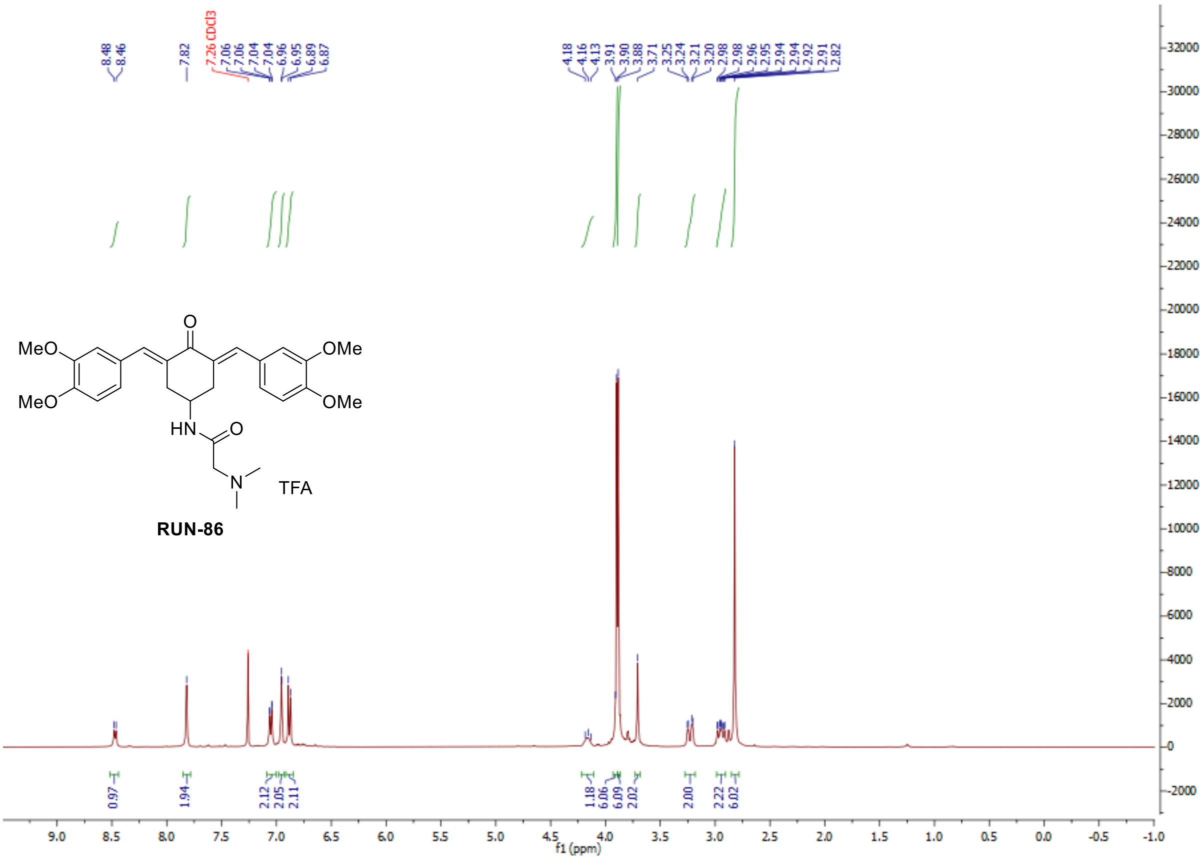
RUN-86
TFA
f1 (ppm)

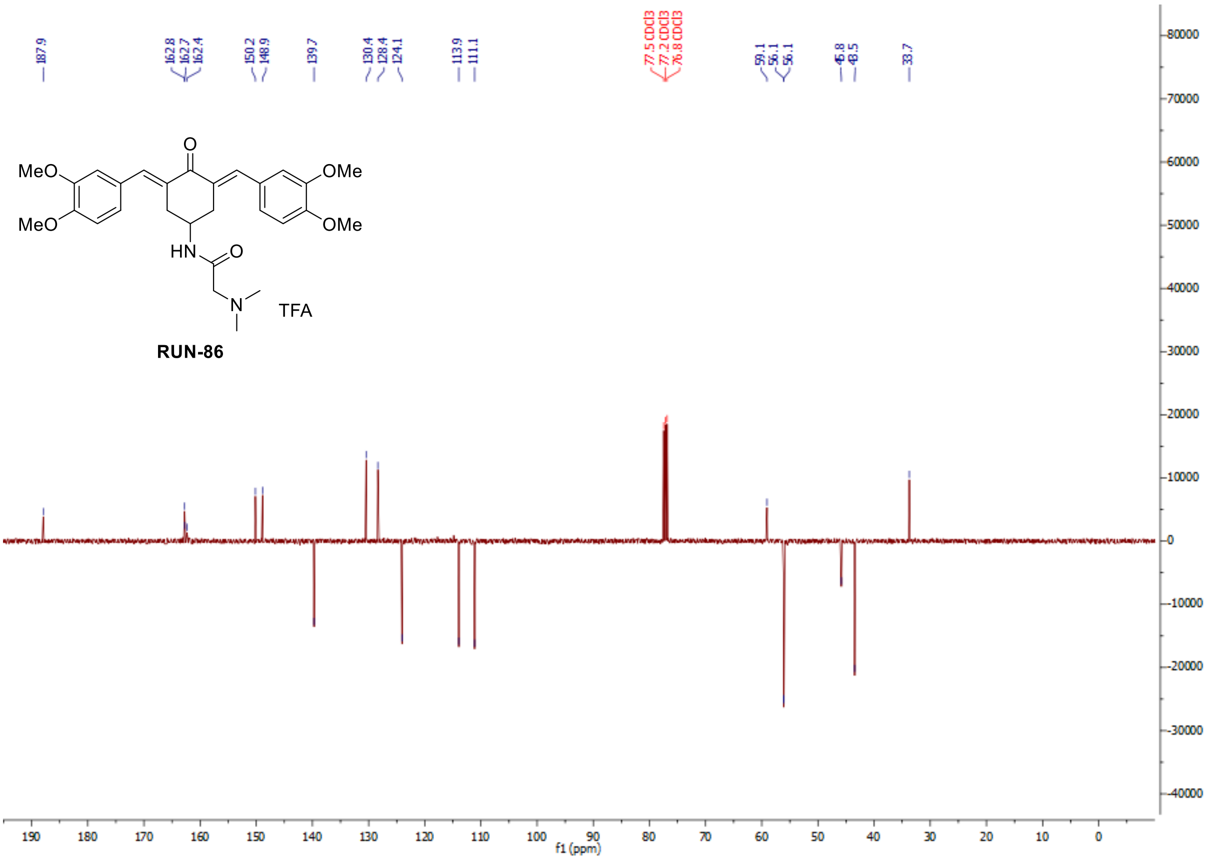
RUN-86
TFA
f1 (ppm)

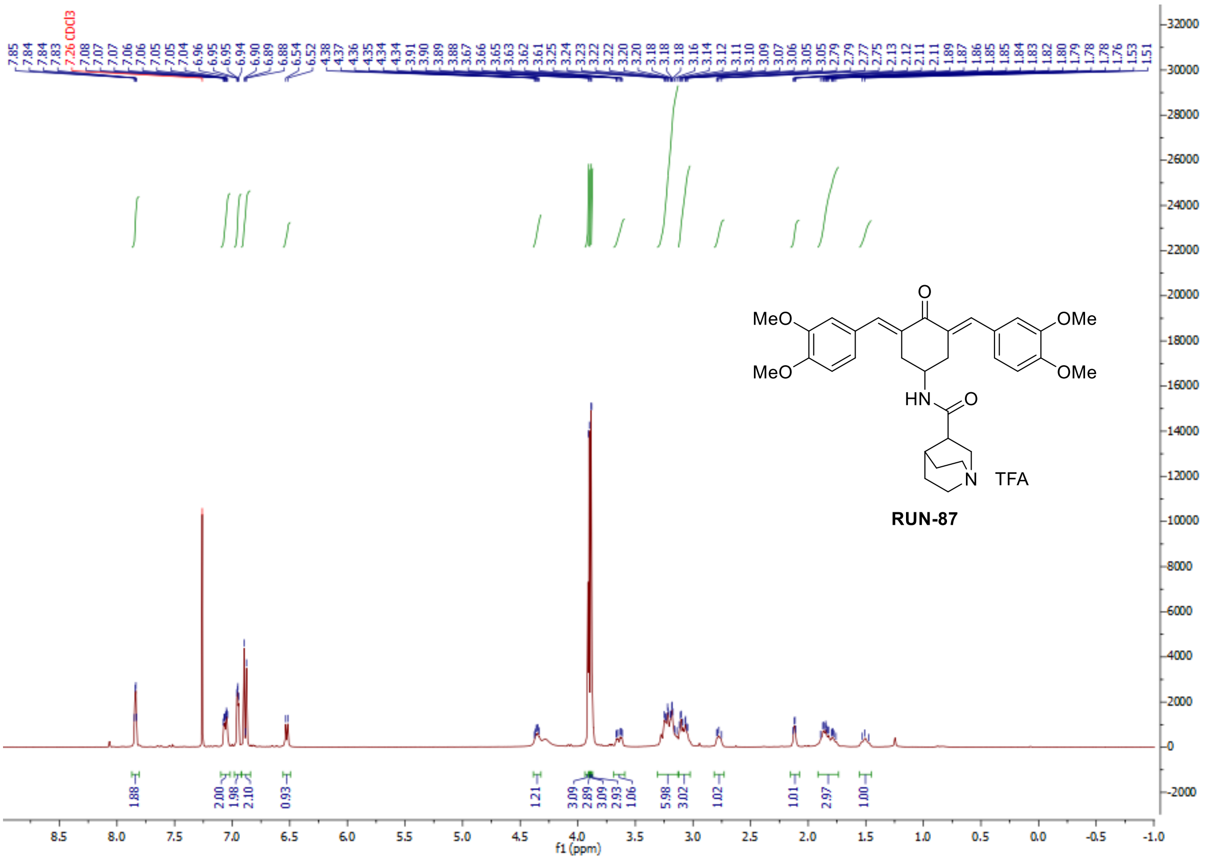

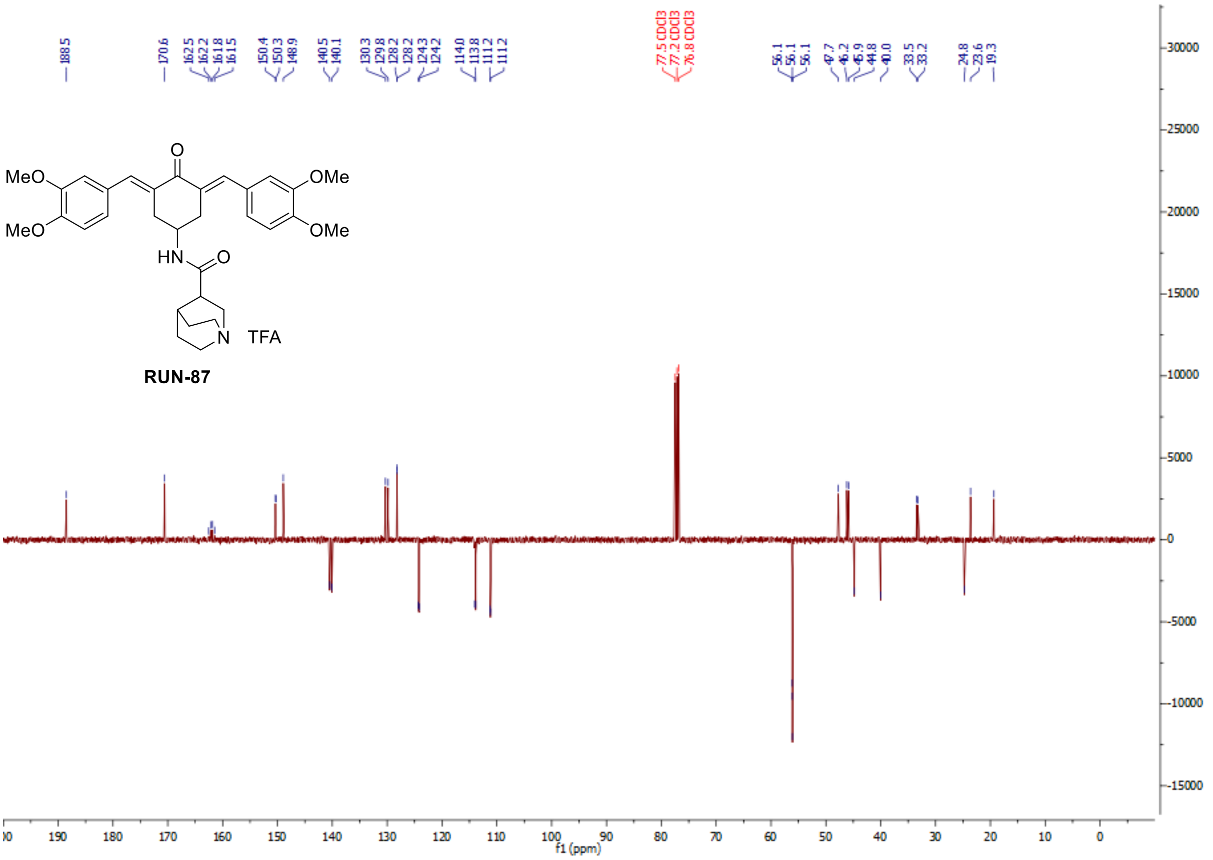

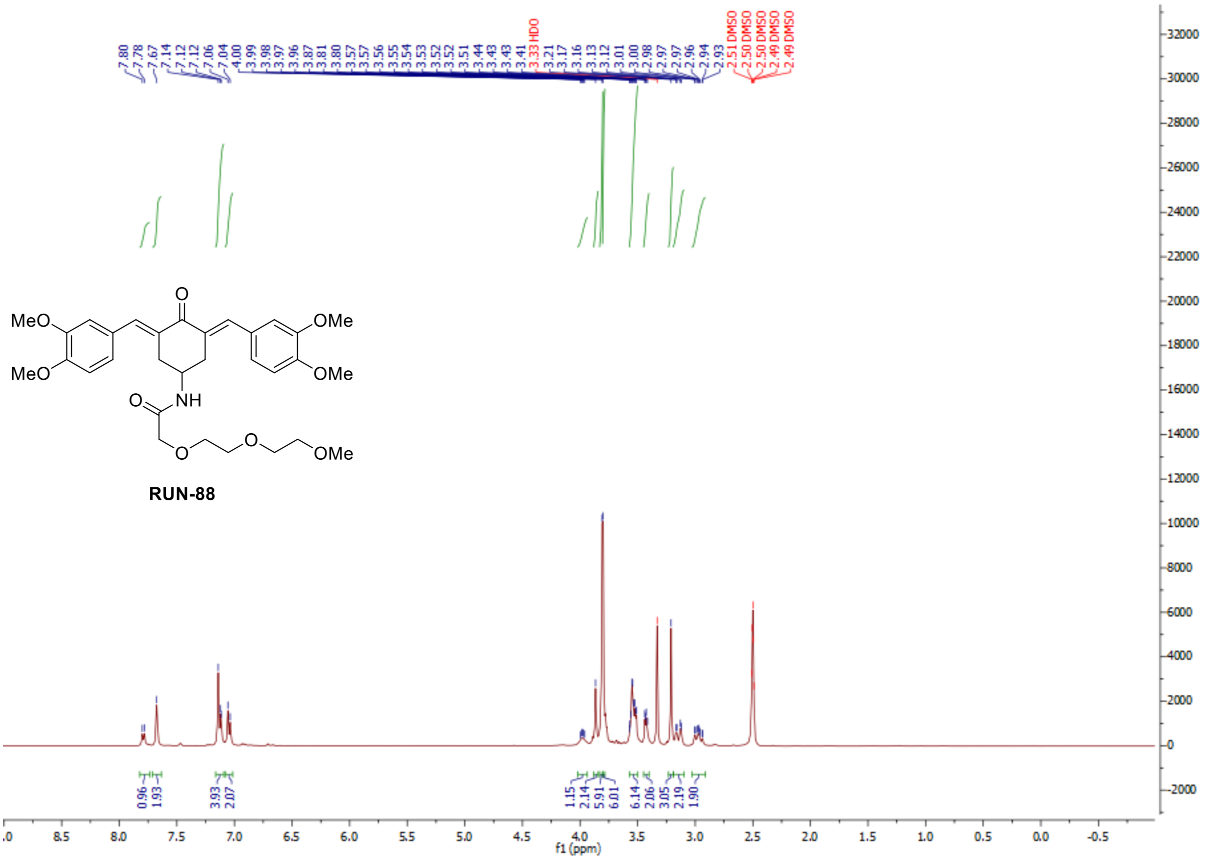

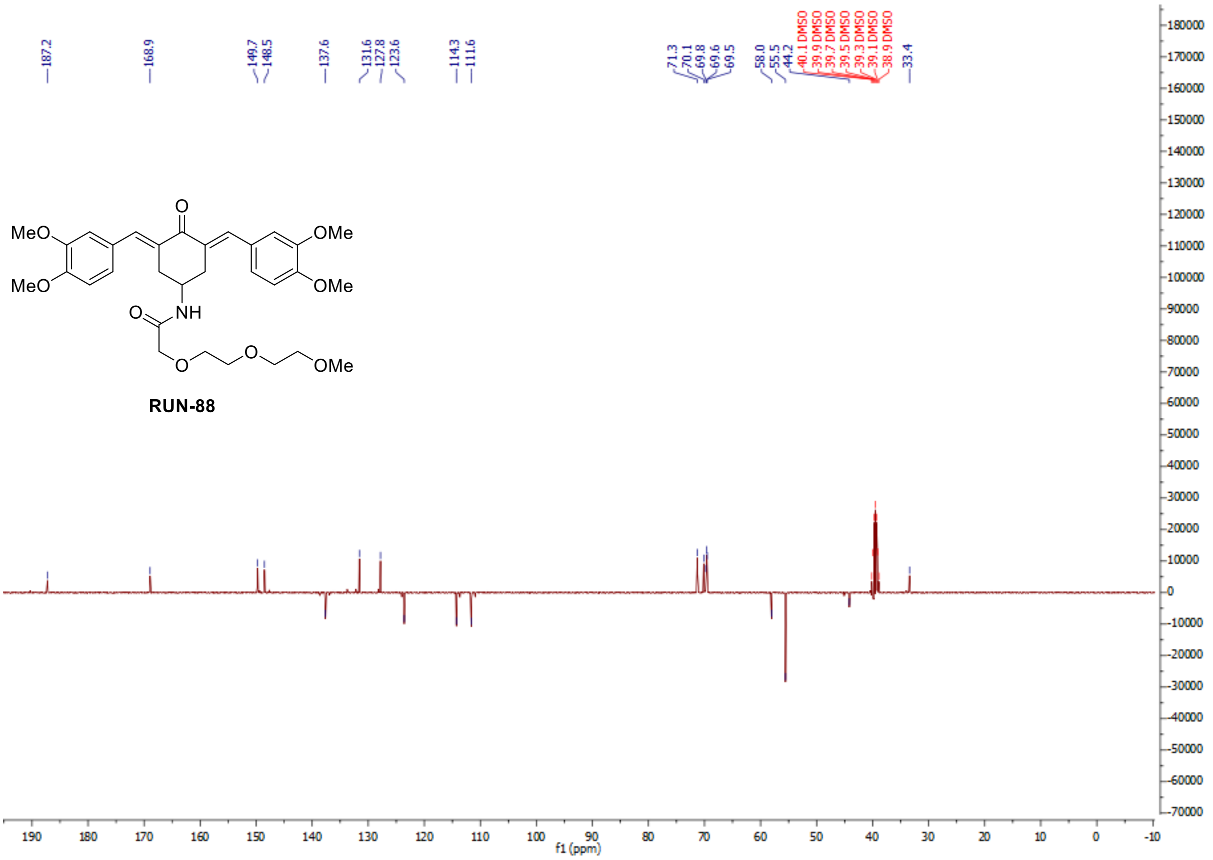

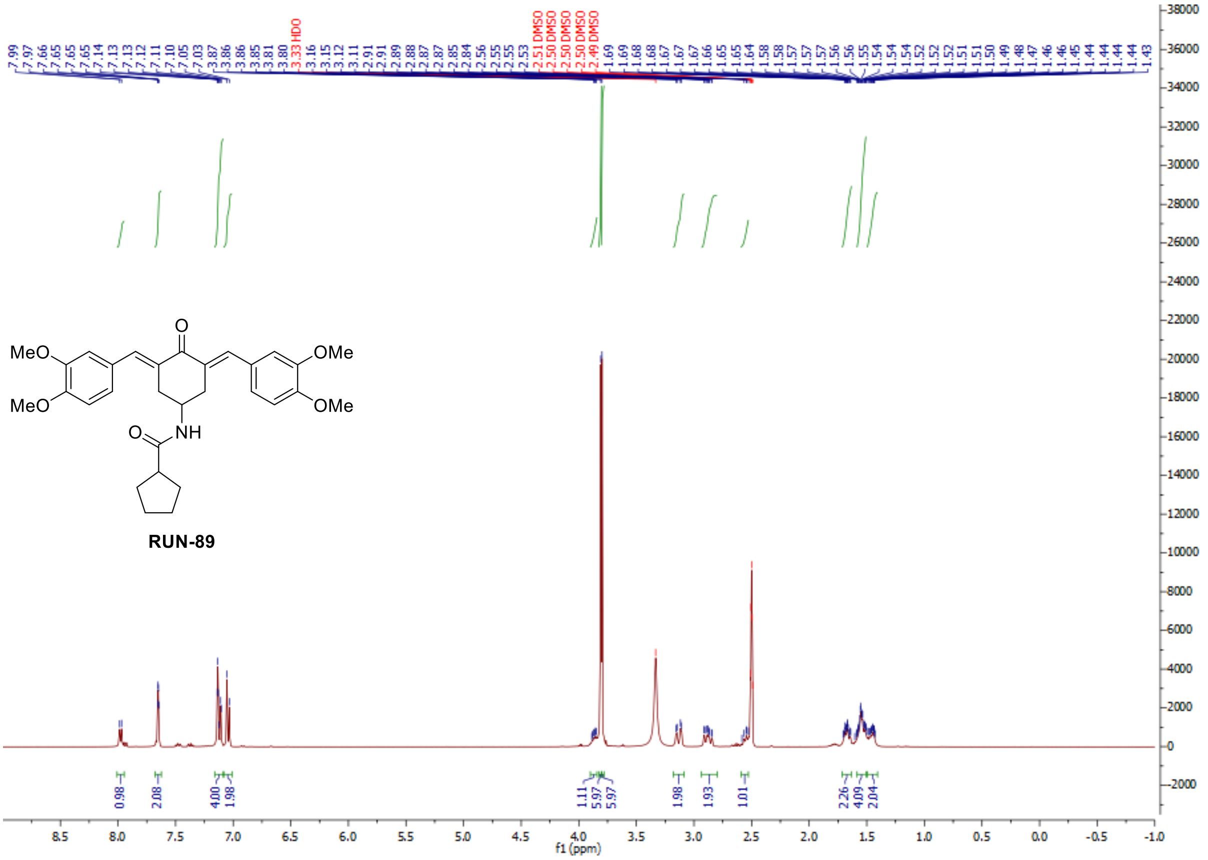
RUN-89
f1 (ppm)

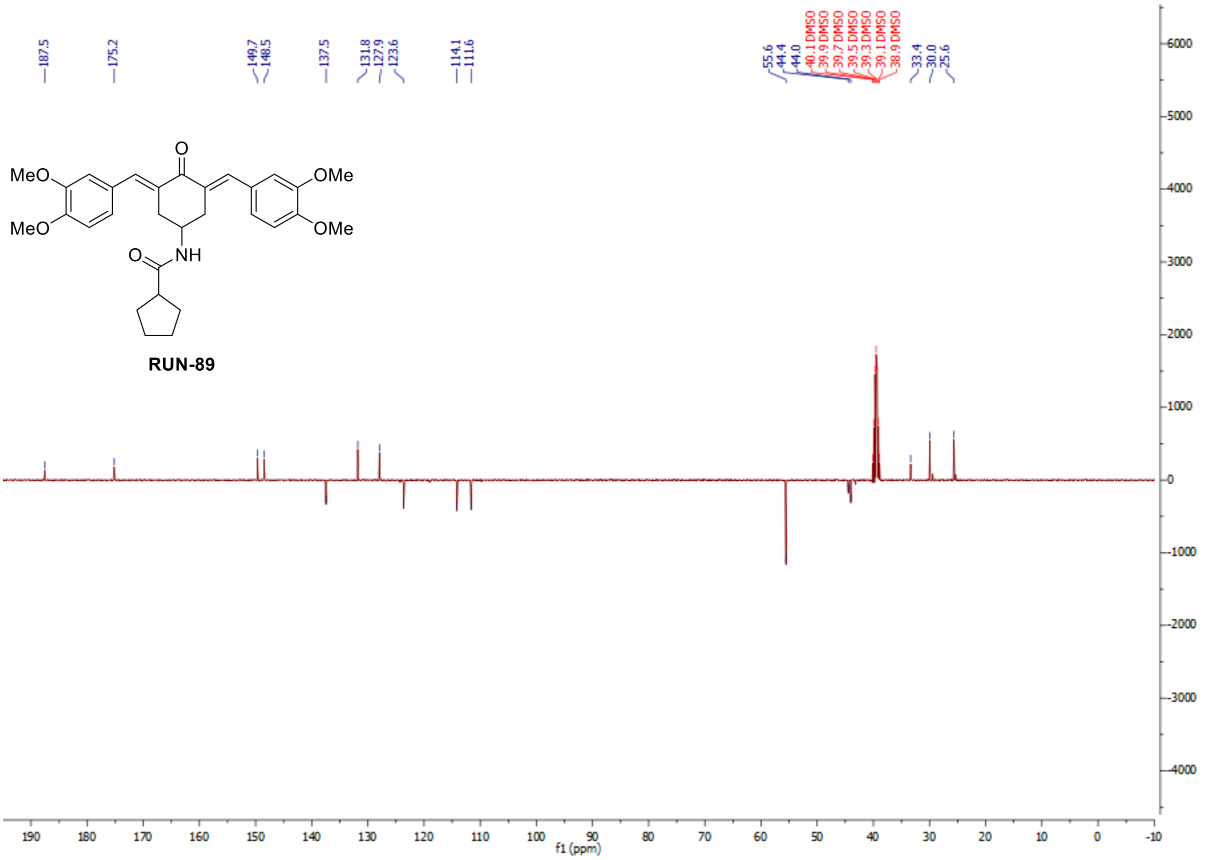
RUN-89
f1 (ppm)

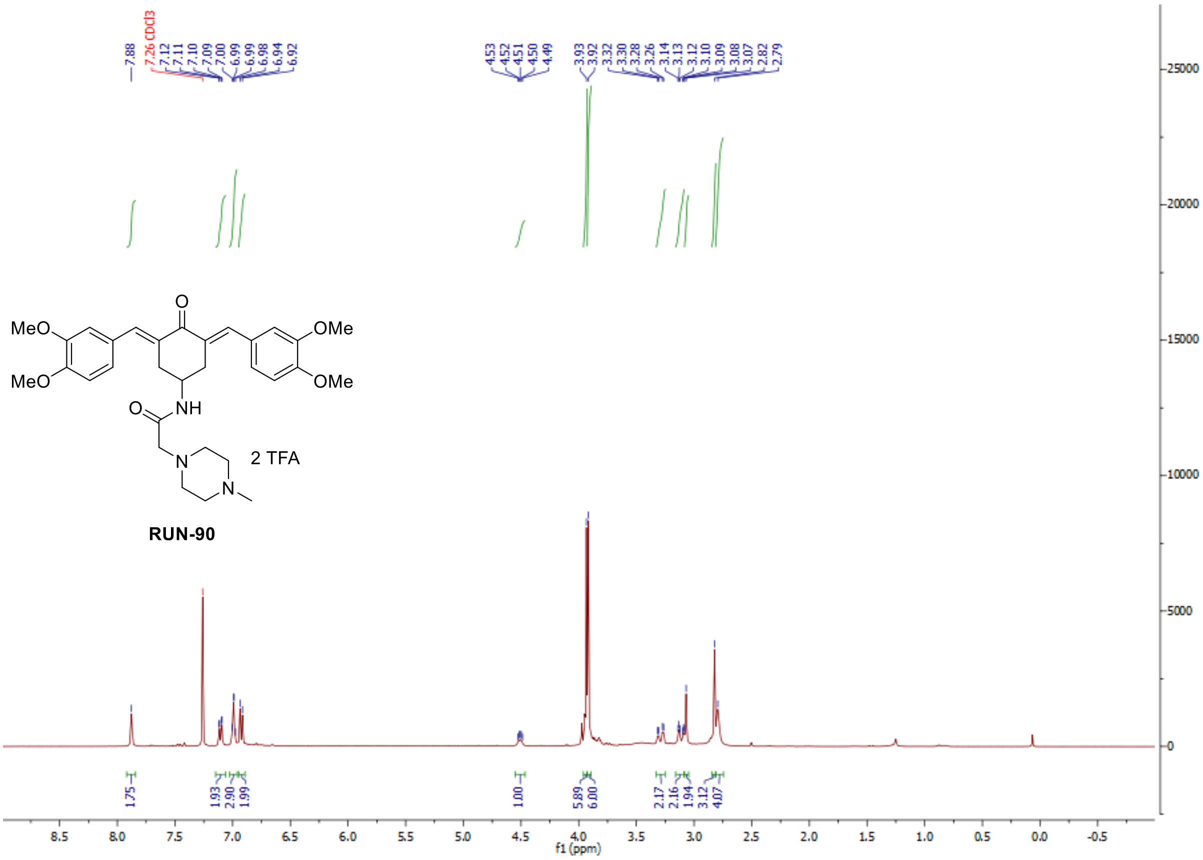

7.88
7.26 CDCl3
7.12
7.11
7.10
7.09
7.00
6.99
6.99
6.98
6.94
6.92
4.53
4.52
4.51
4.50
4.49
3.93
3.92
3.32
3.30
3.28
3.26
3.14
3.13
3.12
3.10
3.09
3.08
3.07
2.82
2.79
MeO
MeO
O
OMe
OMe
O
NH
N
N
2 TFA
RUN-90
1.75
1.93
2.90
1.99
1.00
5.89
6.00
2.17
2.16
1.94
3.12
4.07
f1 (ppm)

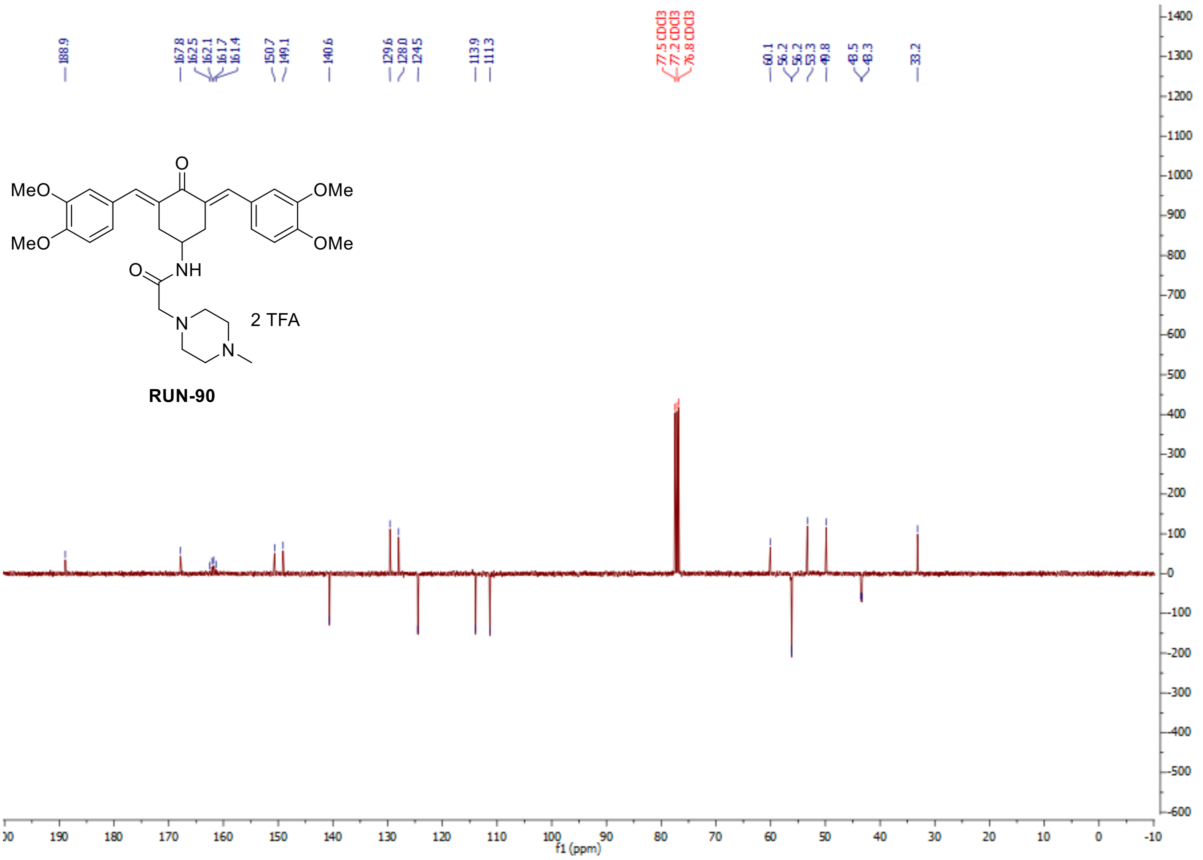

188.9
167.8
162.5
162.1
161.7
161.4
150.7
149.1
140.6
129.6
128.0
124.5
113.9
111.3
77.5 CDCl3
77.2 CDCl3
76.8 CDCl3
60.1
56.2
56.2
53.3
49.8
43.5
43.3
33.2
MeO
MeO
O
OMe
OMe
O
NH
N
N
2 TFA
RUN-90
f1 (ppm)

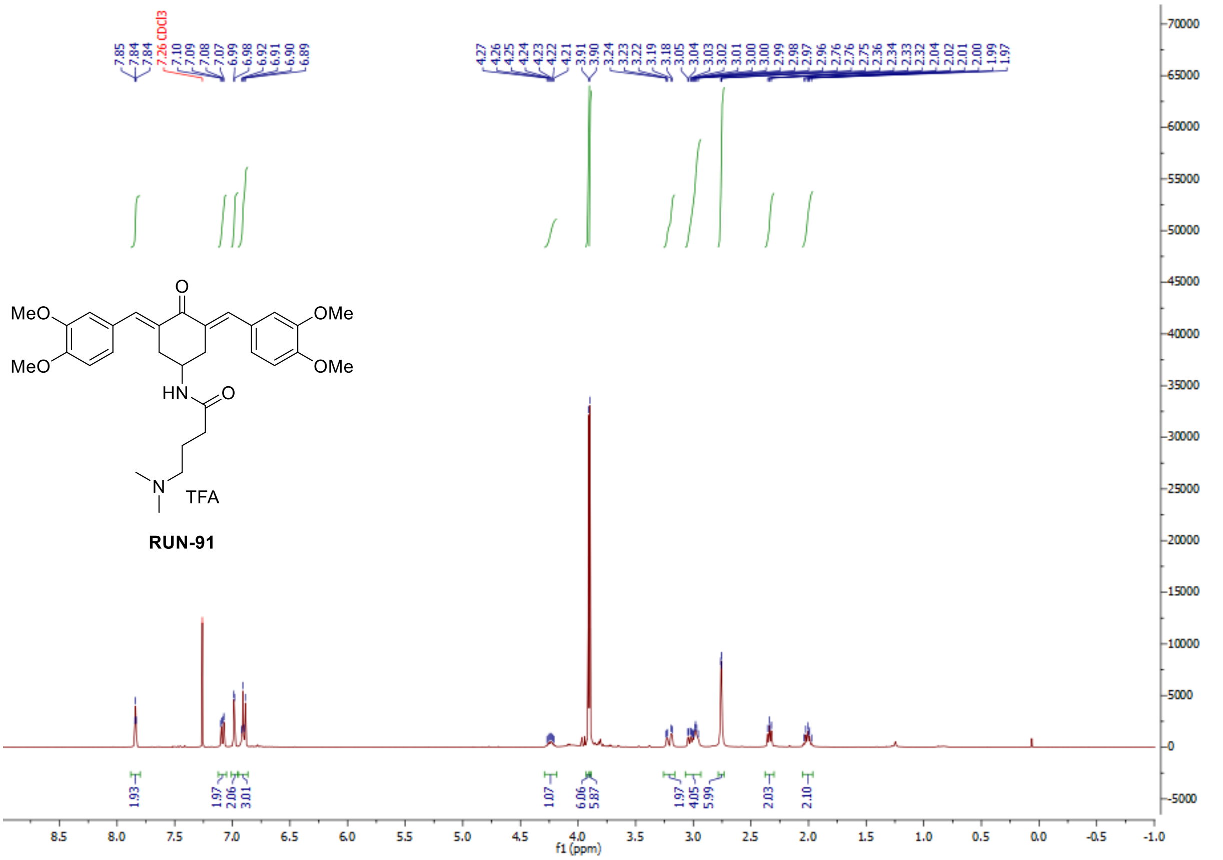

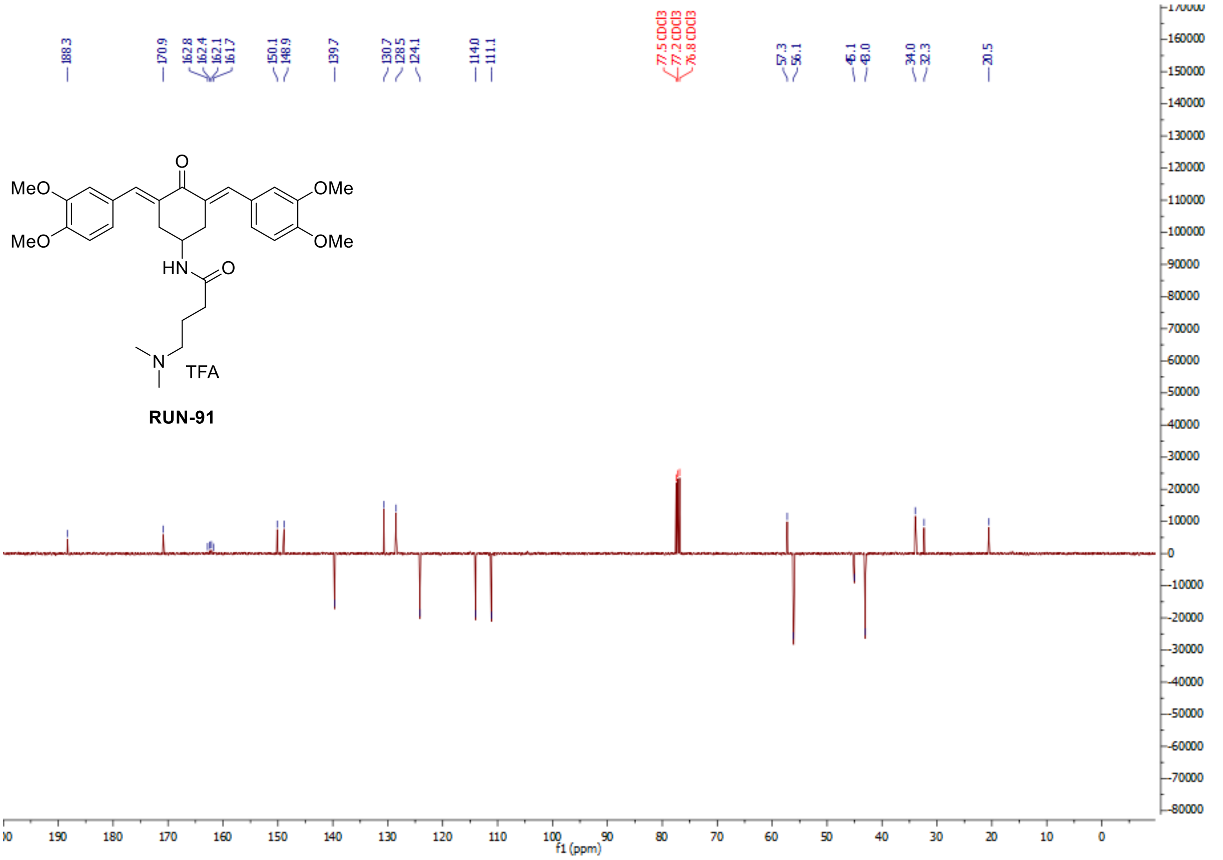

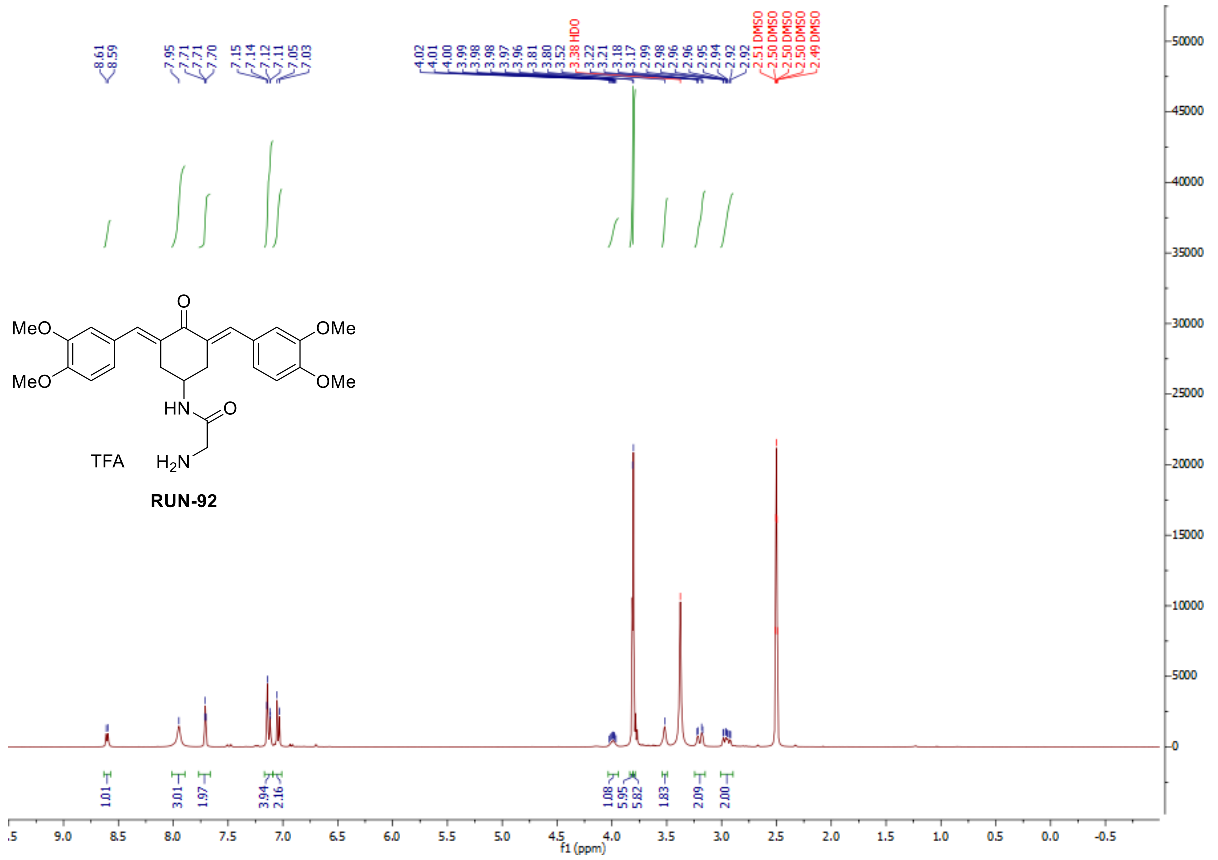

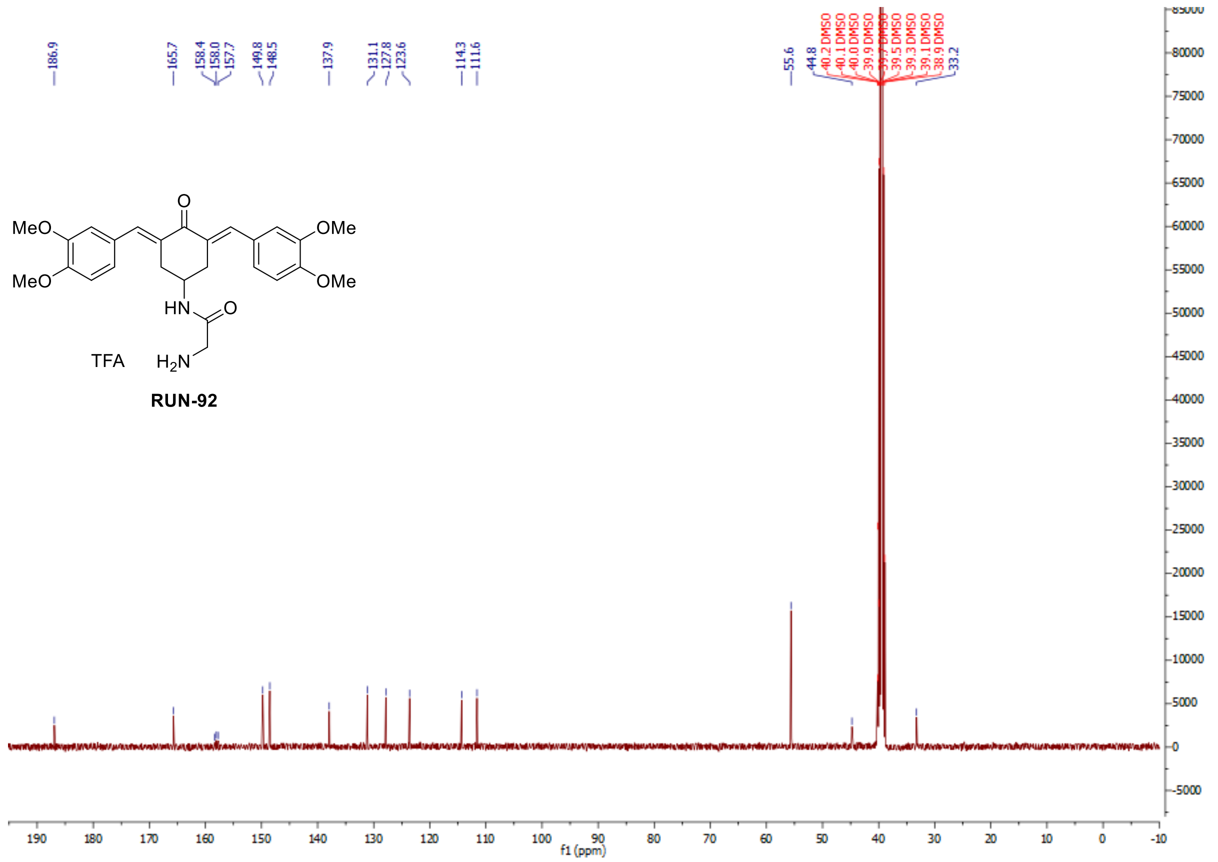

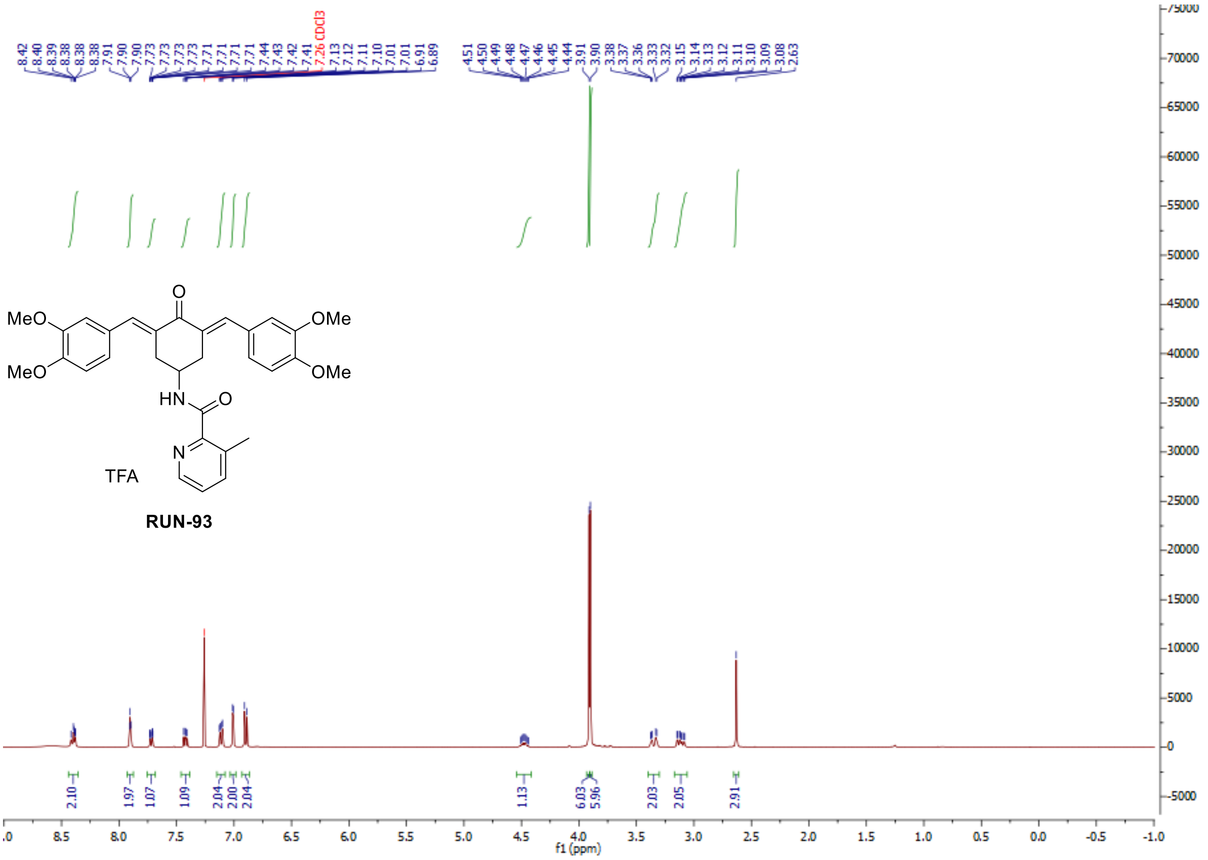

MeO
MeO
OMe
OMe
O
HN
O
N
TFA
RUN-93
f1 (ppm)

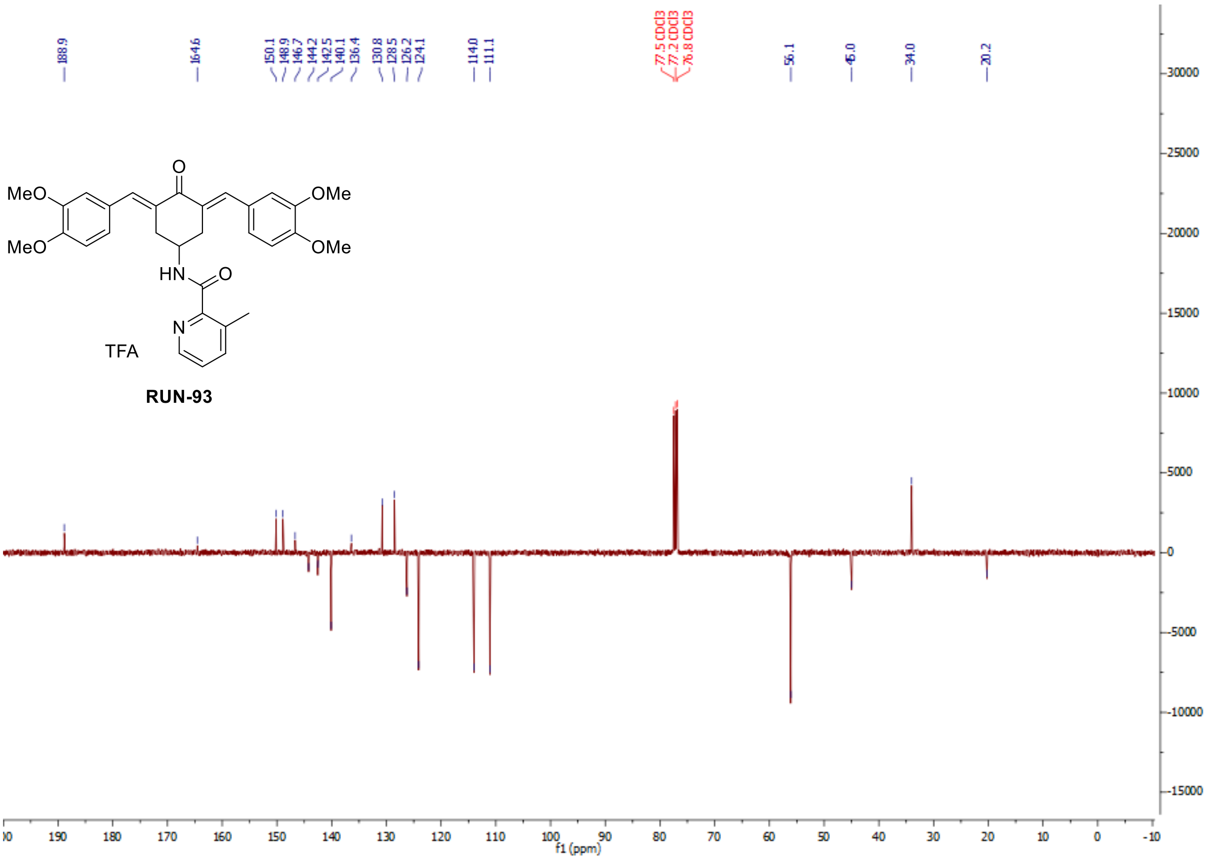

MeO
MeO
OMe
OMe
O
HN
O
N
TFA
RUN-93
f1 (ppm)

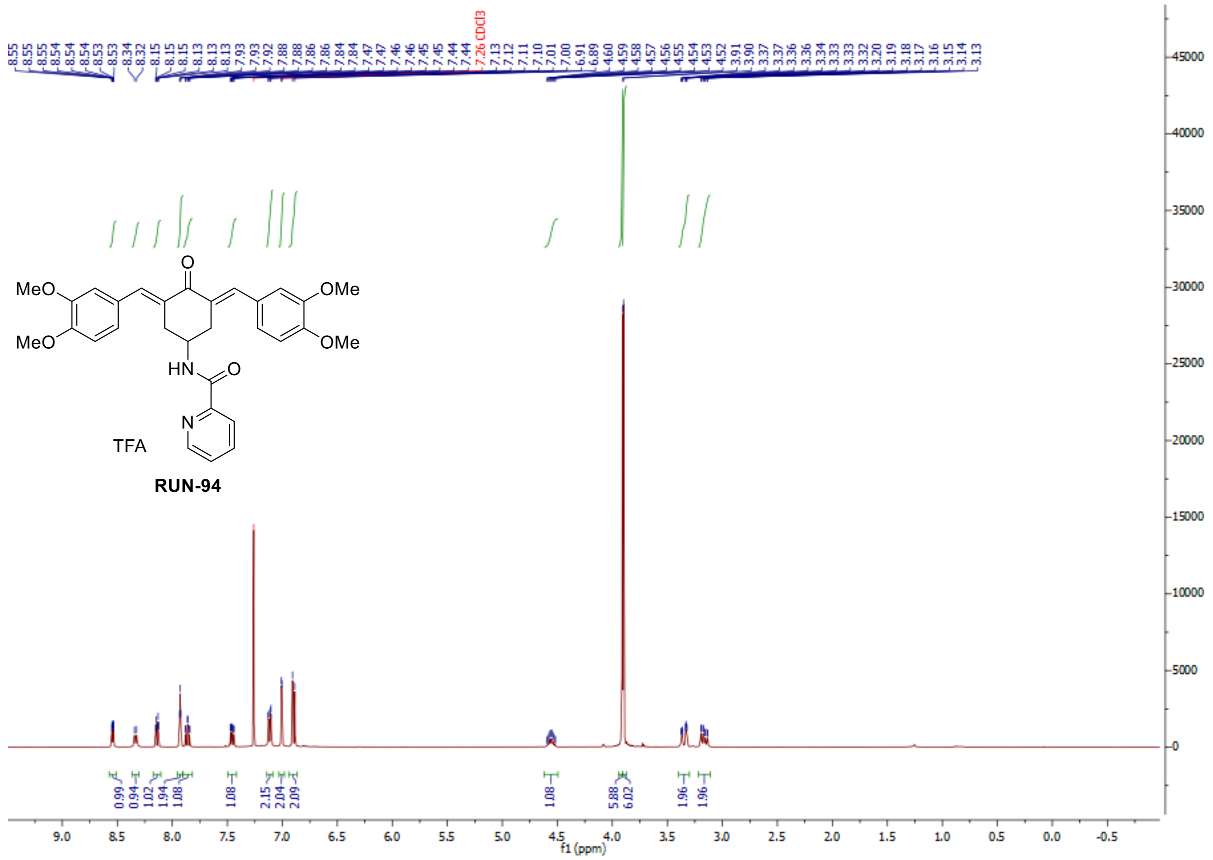
MeO
MeO
OMe
OMe
O
HN
O
N
TFA
RUN-94
f1 (ppm)

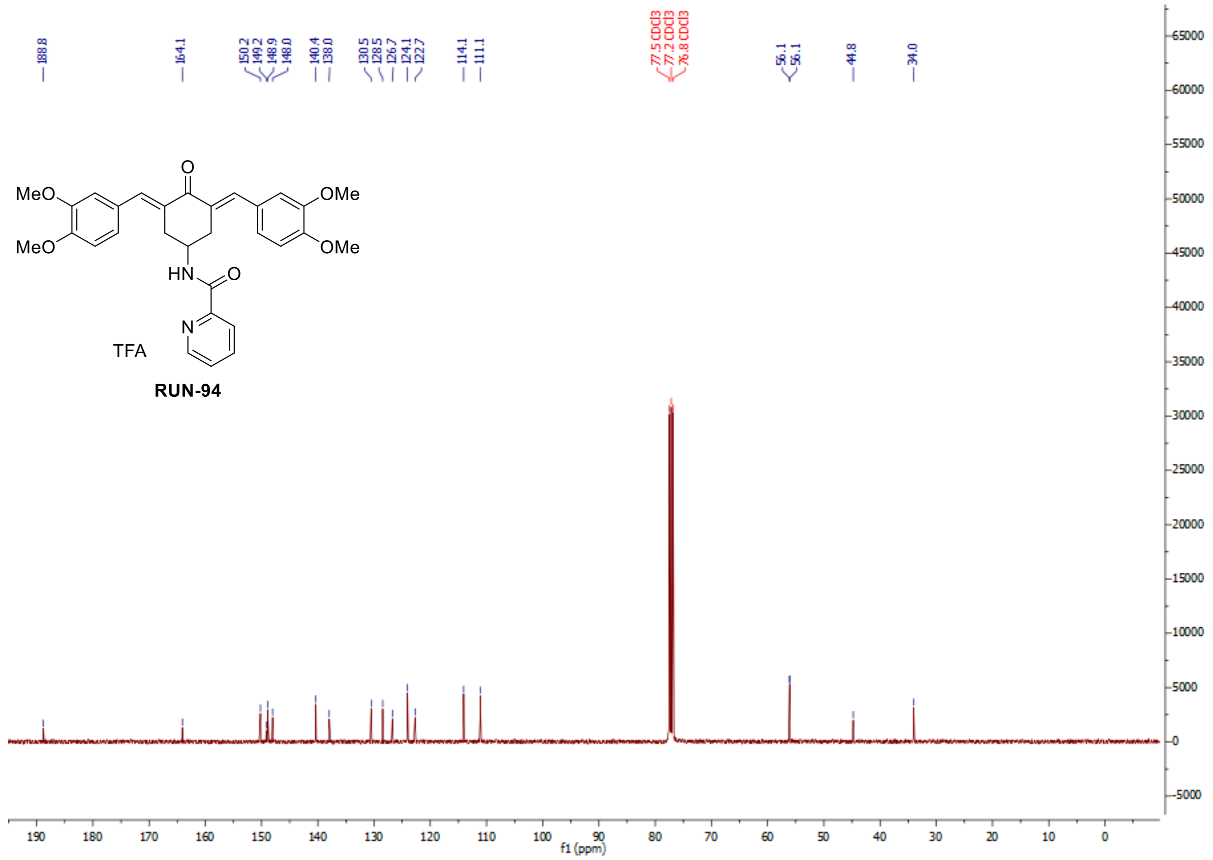
MeO
MeO
OMe
OMe
O
HN
O
N
TFA
RUN-94
f1 (ppm)

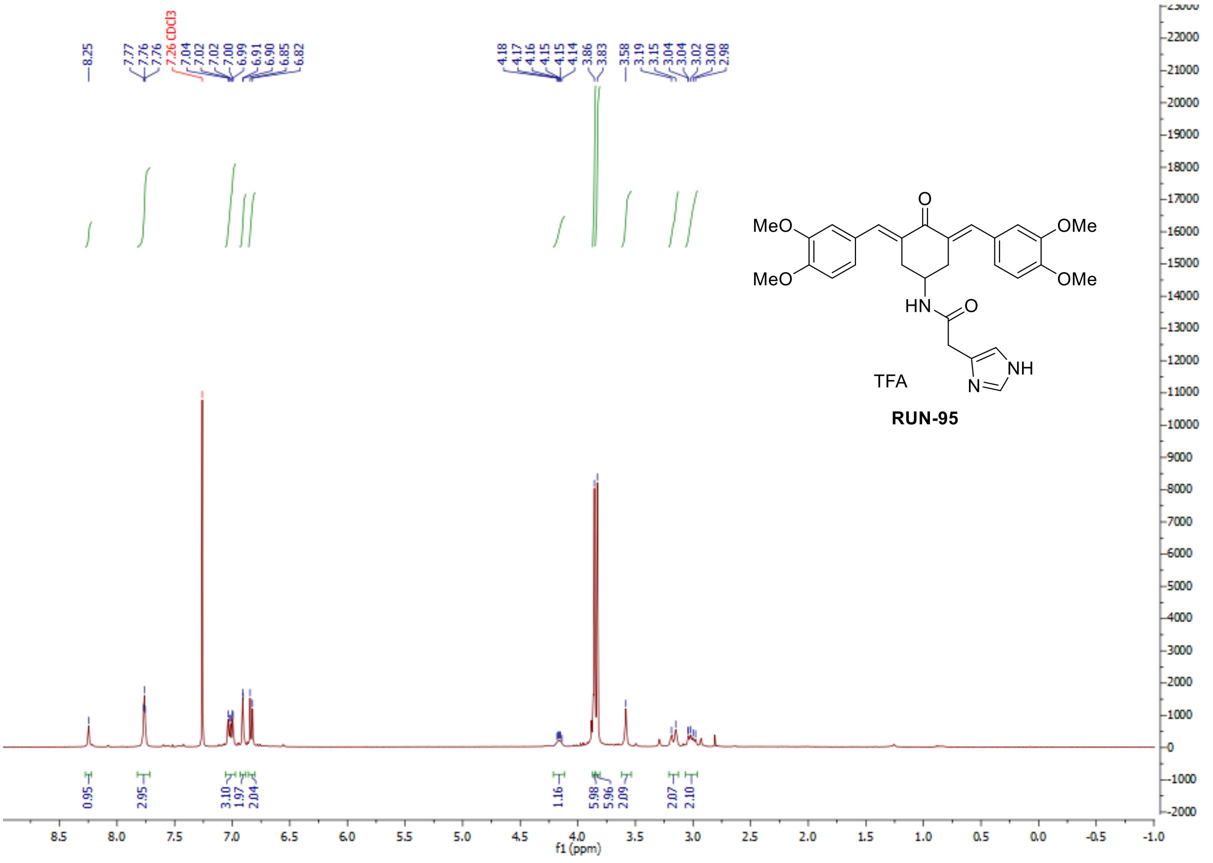
MeO
MeO
OMe
OMe
O
HN
O
NH
N
TFA
RUN-95
f1 (ppm)

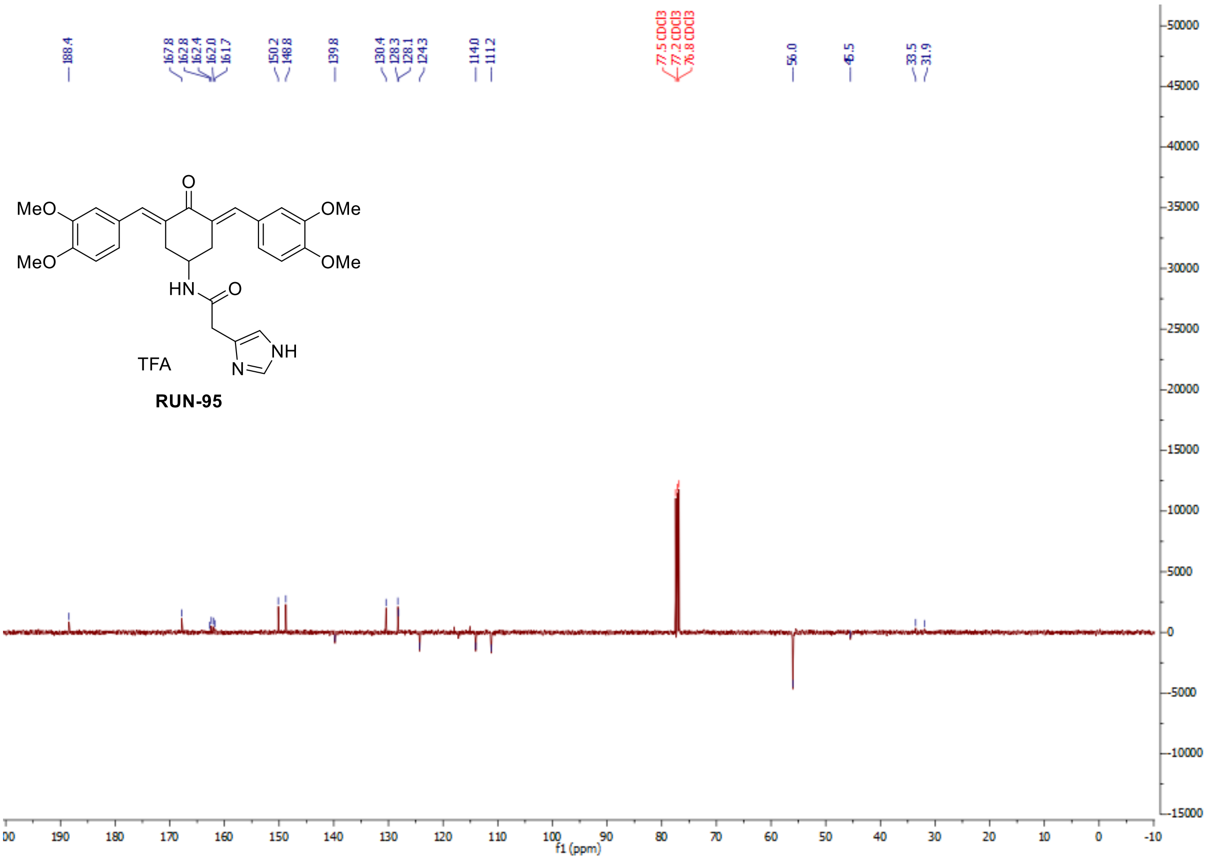
MeO
MeO
OMe
OMe
O
HN
O
NH
N
TFA
RUN-95
f1 (ppm)

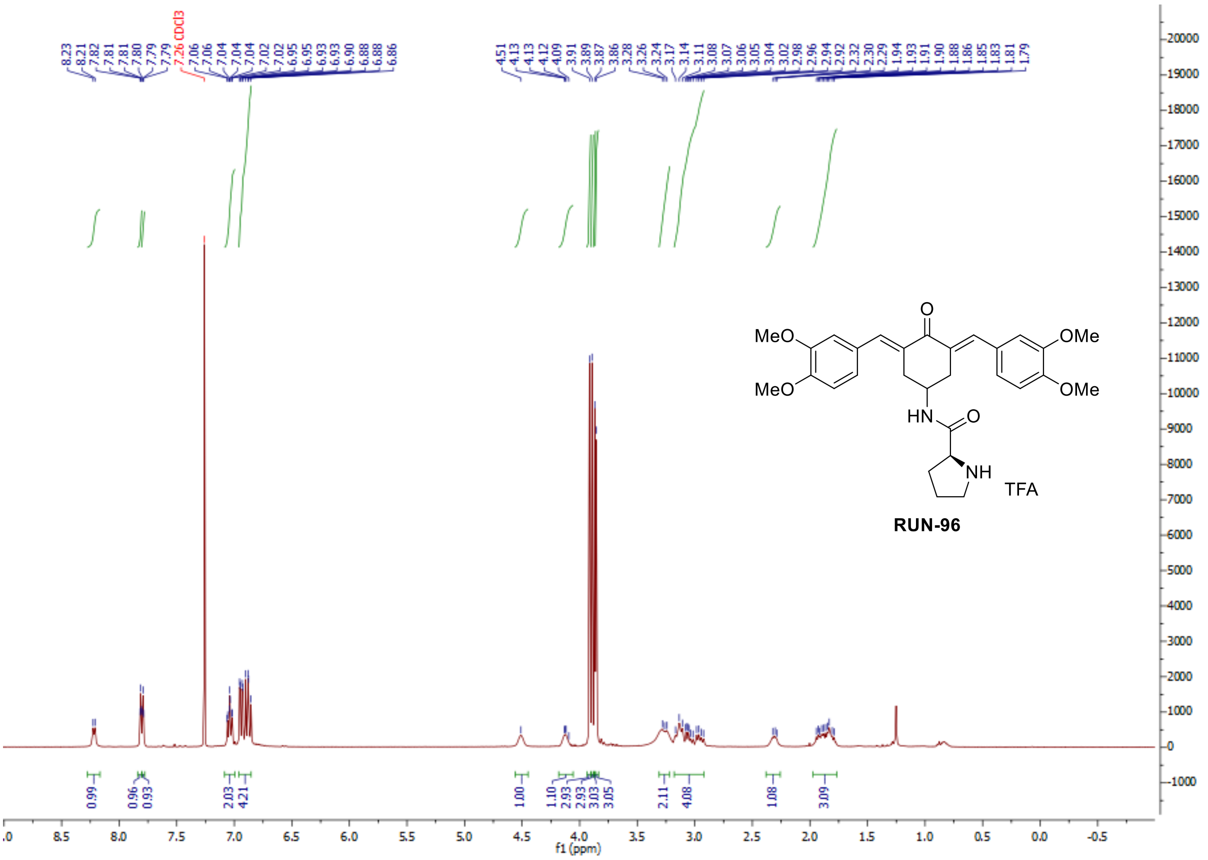

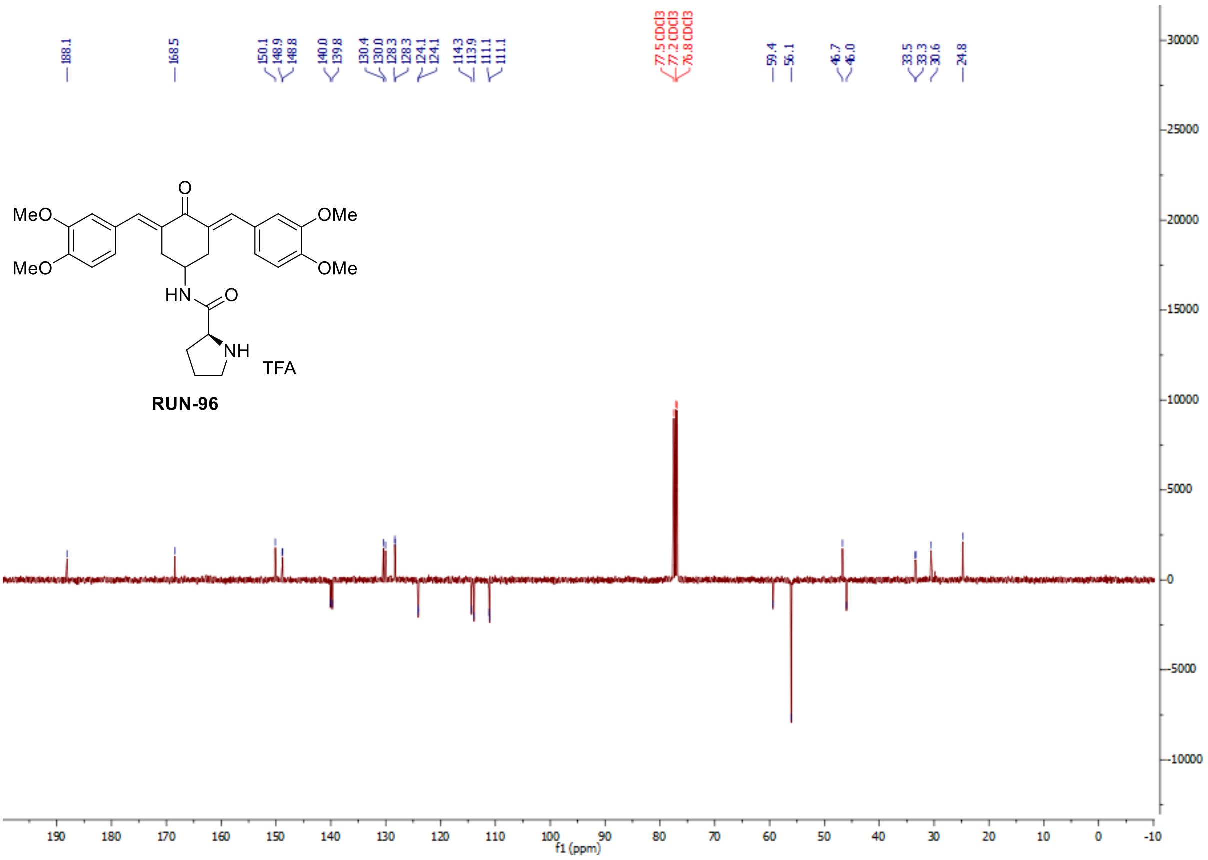

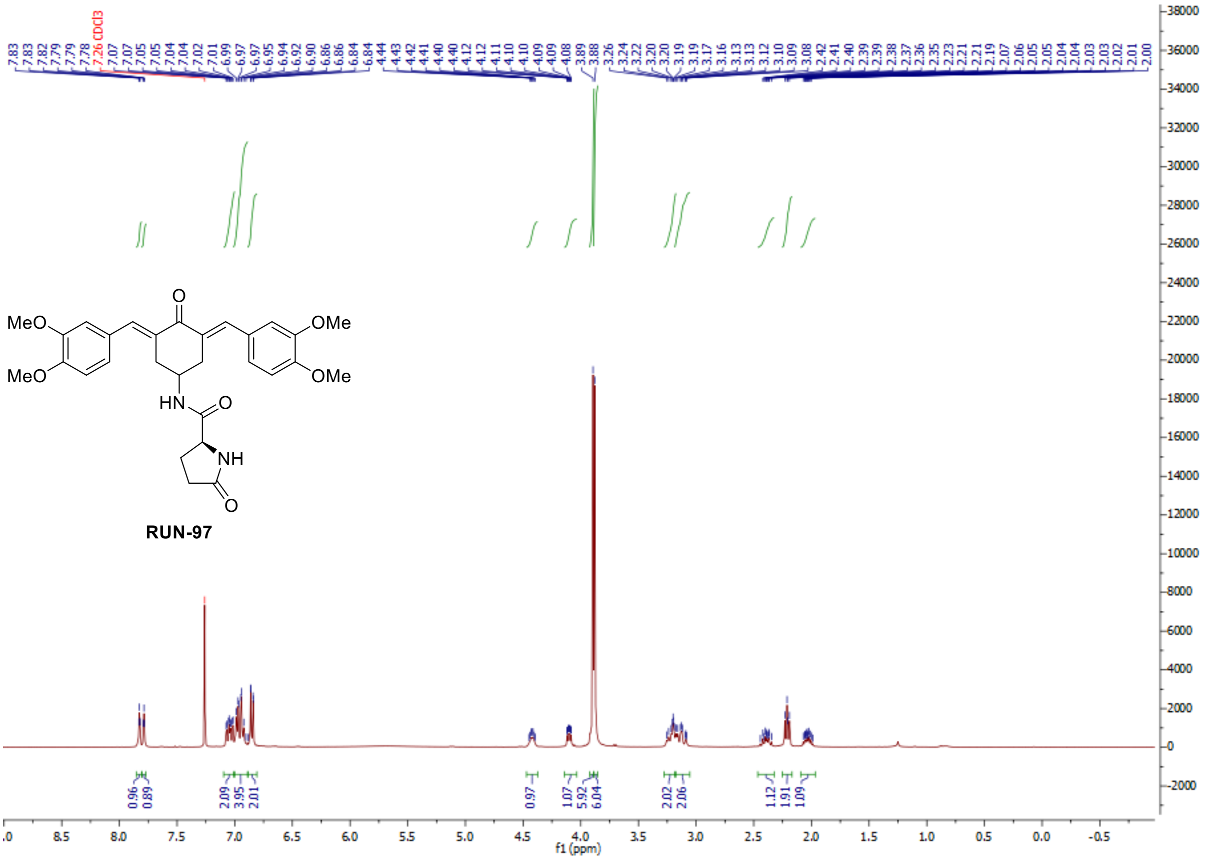
MeO
MeO
OMe
OMe
O
HN
O
NH
O
RUN-97
f1 (ppm)

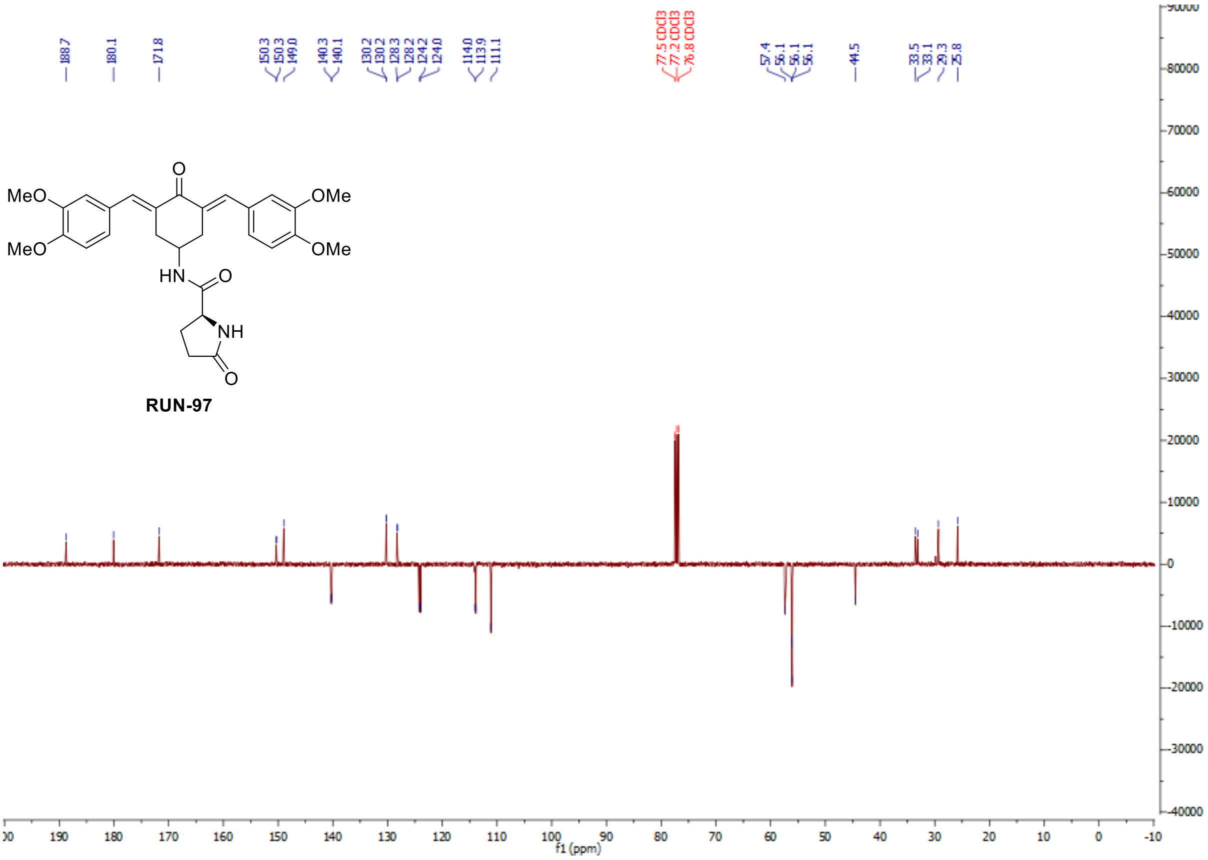
188.7
180.1
171.8
150.3
150.3
149.0
140.3
140.1
130.2
130.2
128.3
128.2
124.2
124.0
114.0
113.9
111.1
77.5 CDCl3
77.2 CDCl3
76.8 CDCl3
57.4
56.1
56.1
56.1
44.5
33.5
33.1
29.3
25.8
MeO
MeO
OMe
OMe
O
HN
O
NH
O
RUN-97
f1 (ppm)

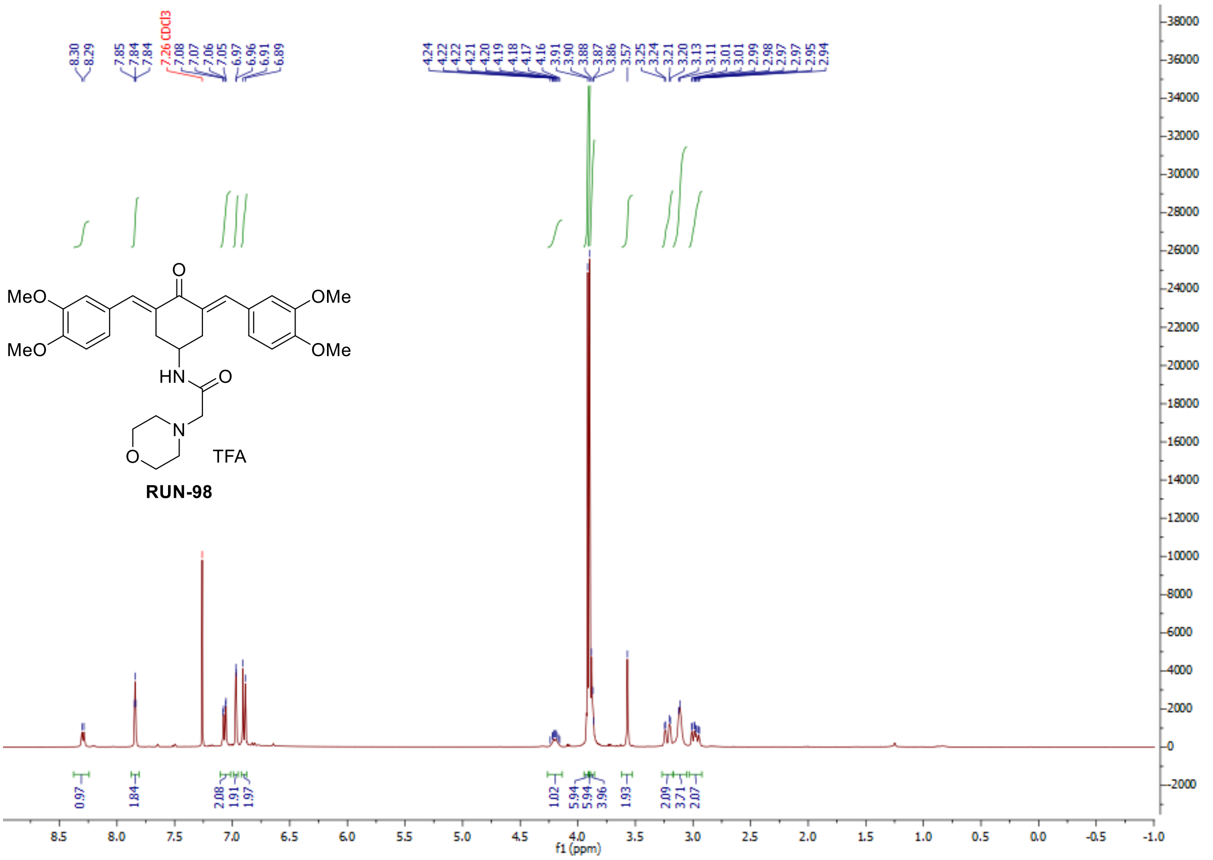
MeO
MeO
OMe
OMe
O
HN
O
N
O
TFA
RUN-98
f1 (ppm)

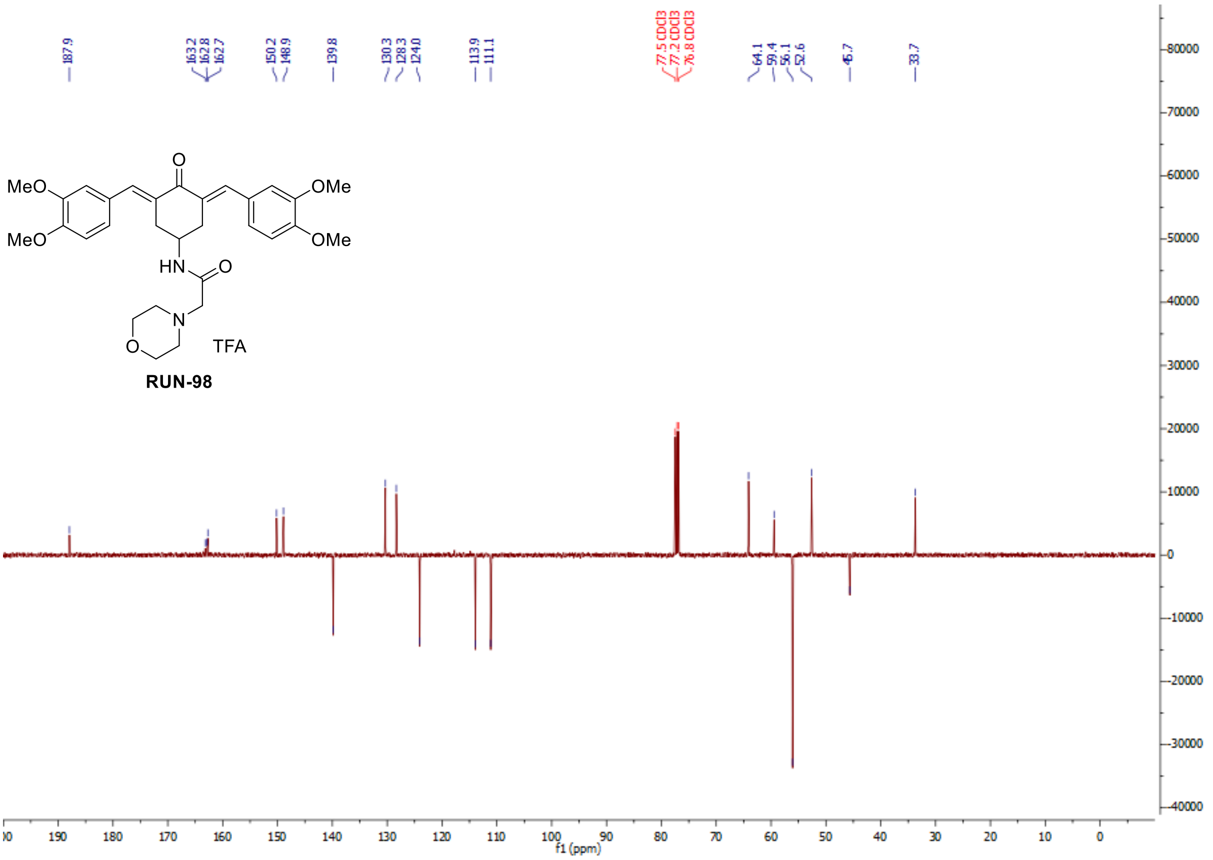
MeO
MeO
OMe
OMe
O
HN
O
N
O
TFA
RUN-98
f1 (ppm)

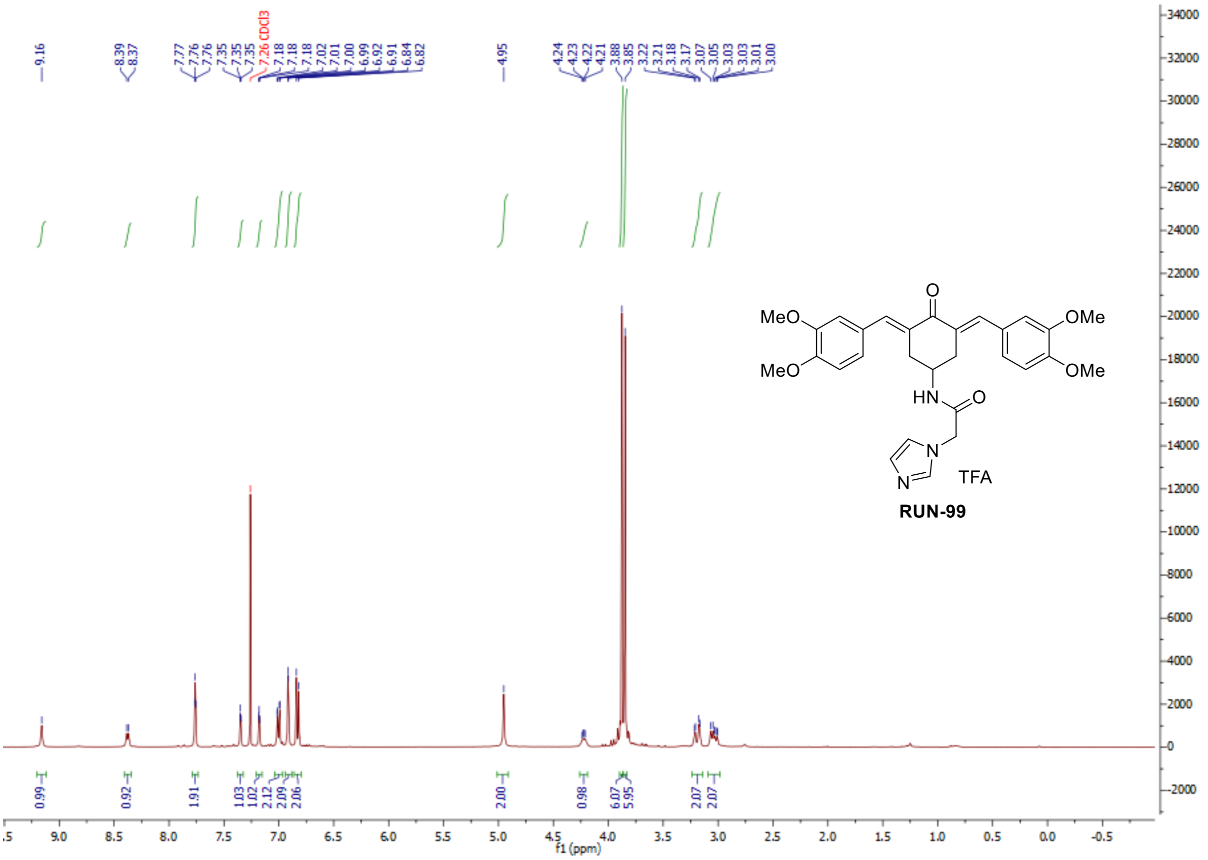
RUN-99
TFA
f1 (ppm)

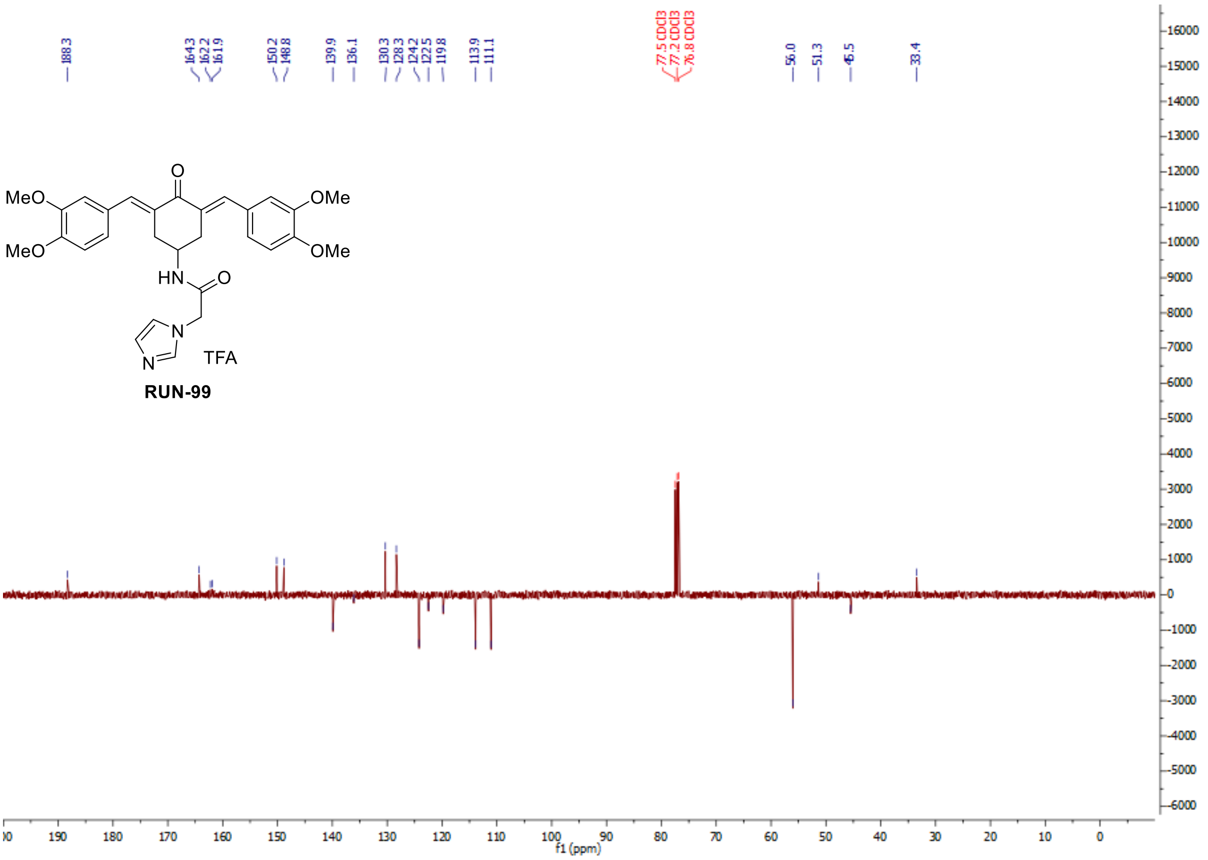
RUN-99
TFA
f1 (ppm)

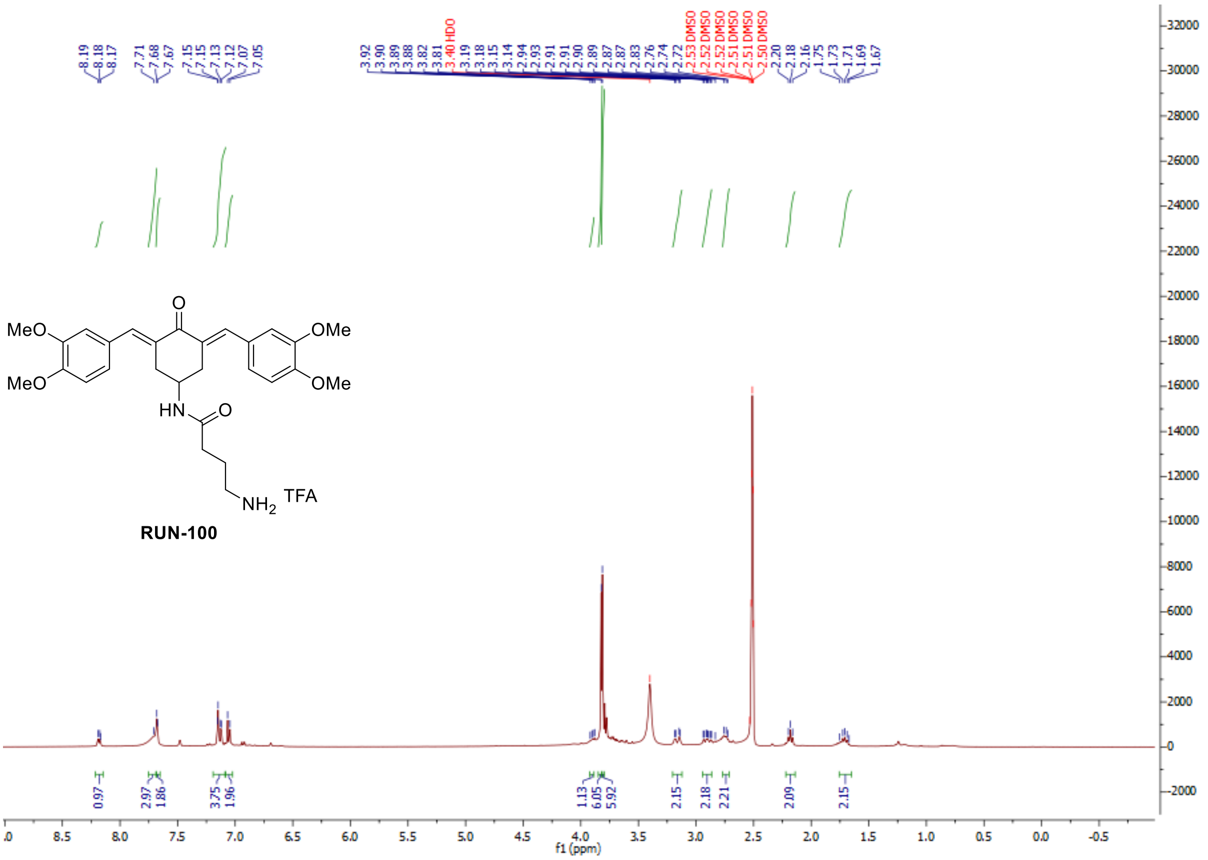
MeO
MeO
OMe
OMe
O
HN
O
NH2
TFA
RUN-100
f1 (ppm)

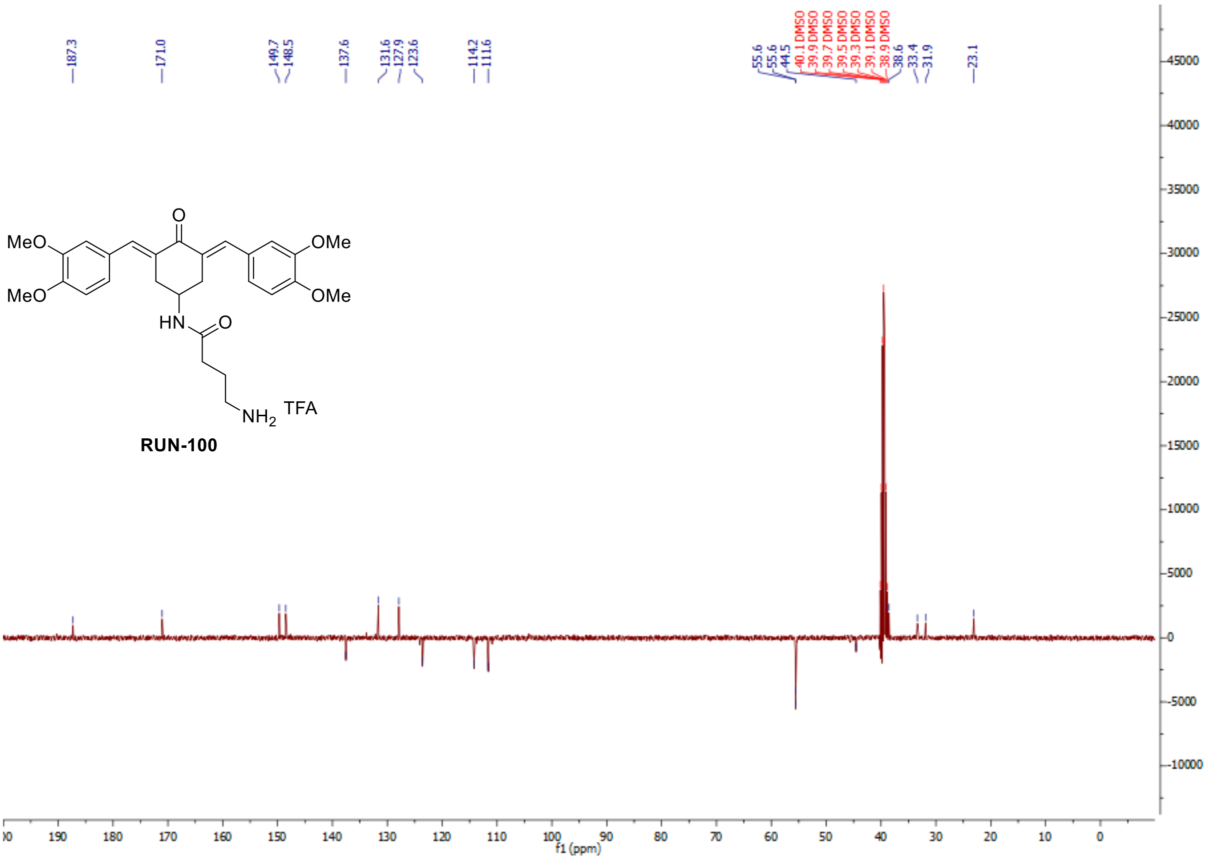
MeO
MeO
OMe
OMe
O
HN
O
NH2
TFA
RUN-100
f1 (ppm)

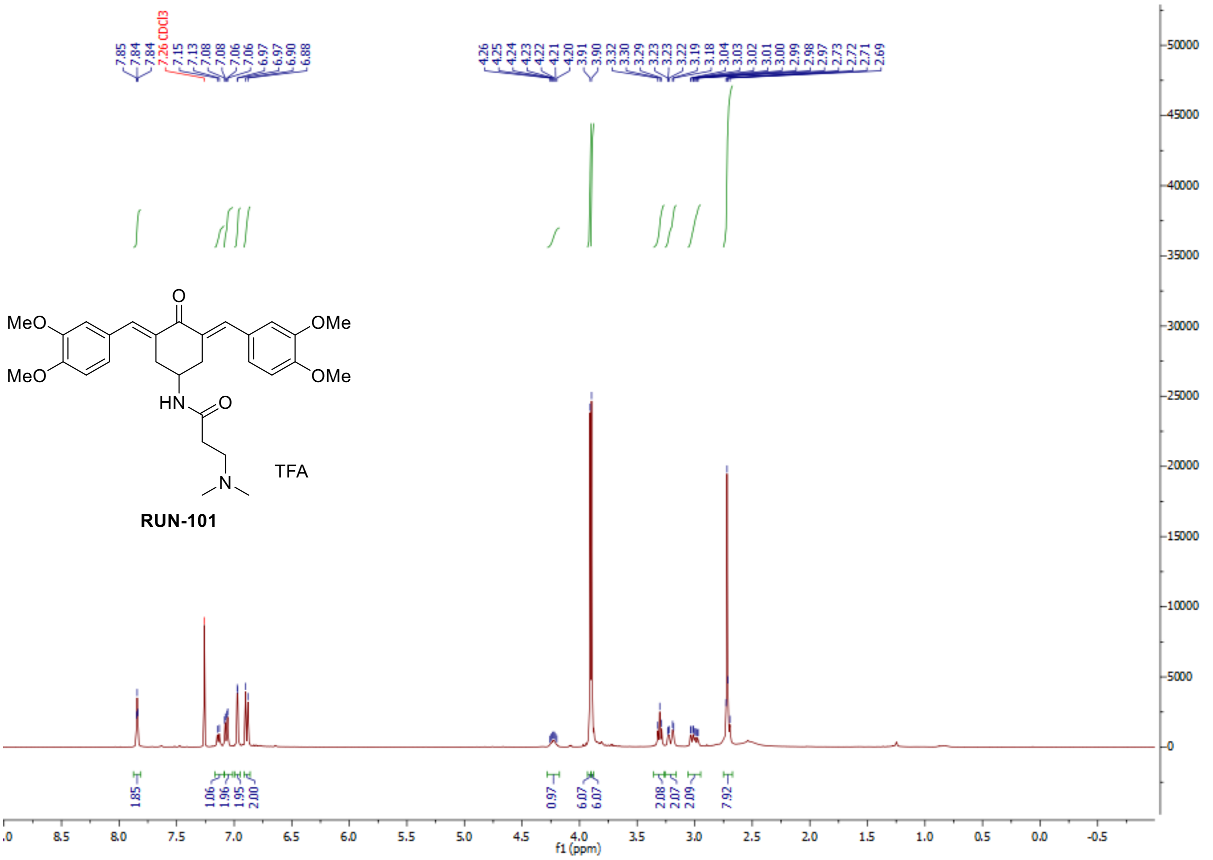
RUN-101
TFA

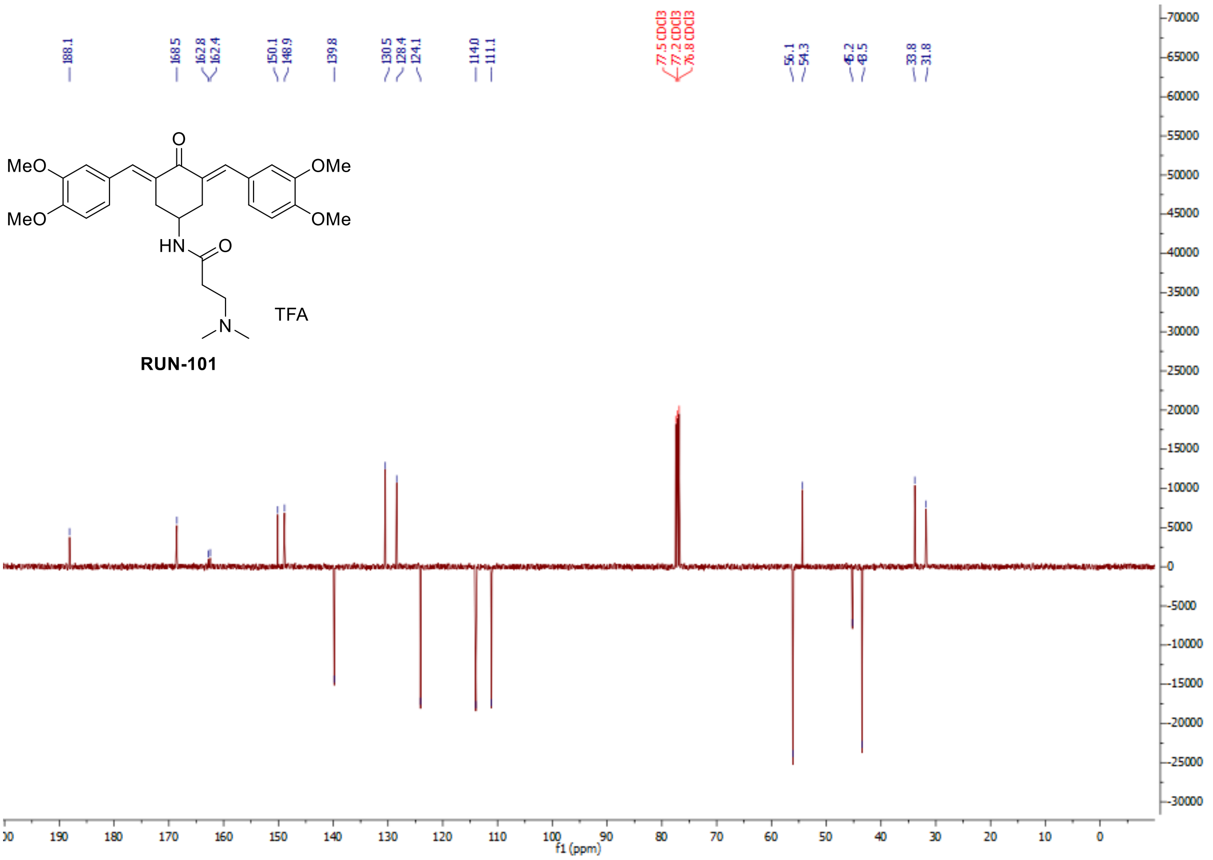
RUN-101
TFA

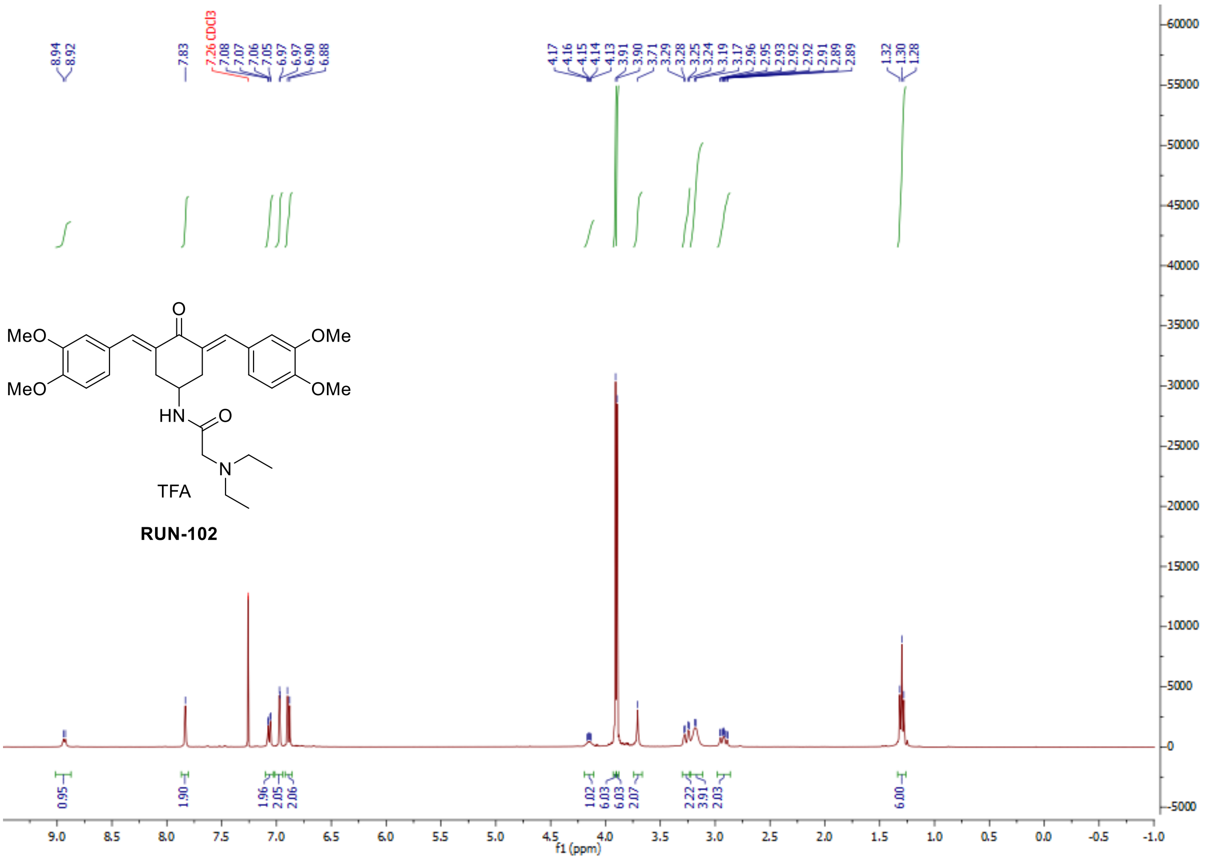
MeO
MeO
OMe
OMe
O
HN
O
N
TFA
RUN-102
f1 (ppm)

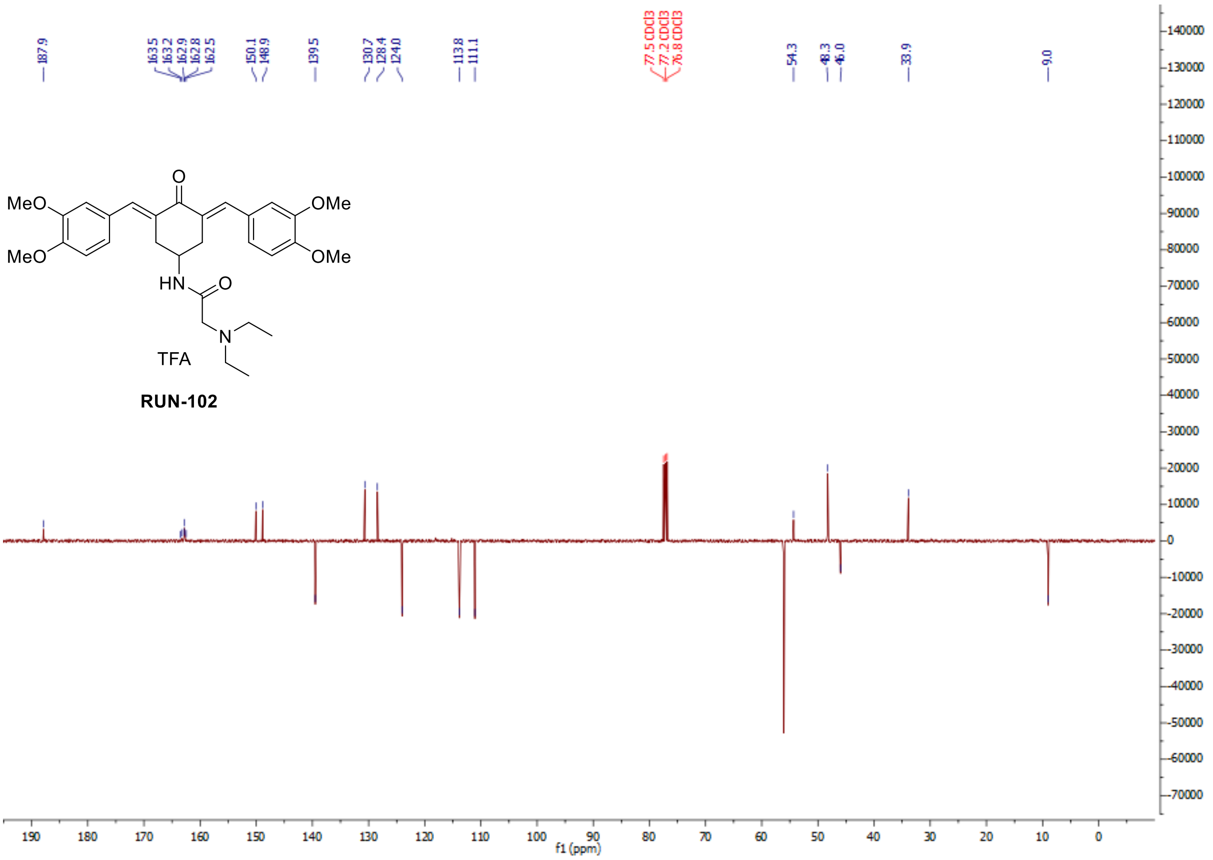
MeO
MeO
OMe
OMe
O
HN
O
N
TFA
RUN-102
f1 (ppm)

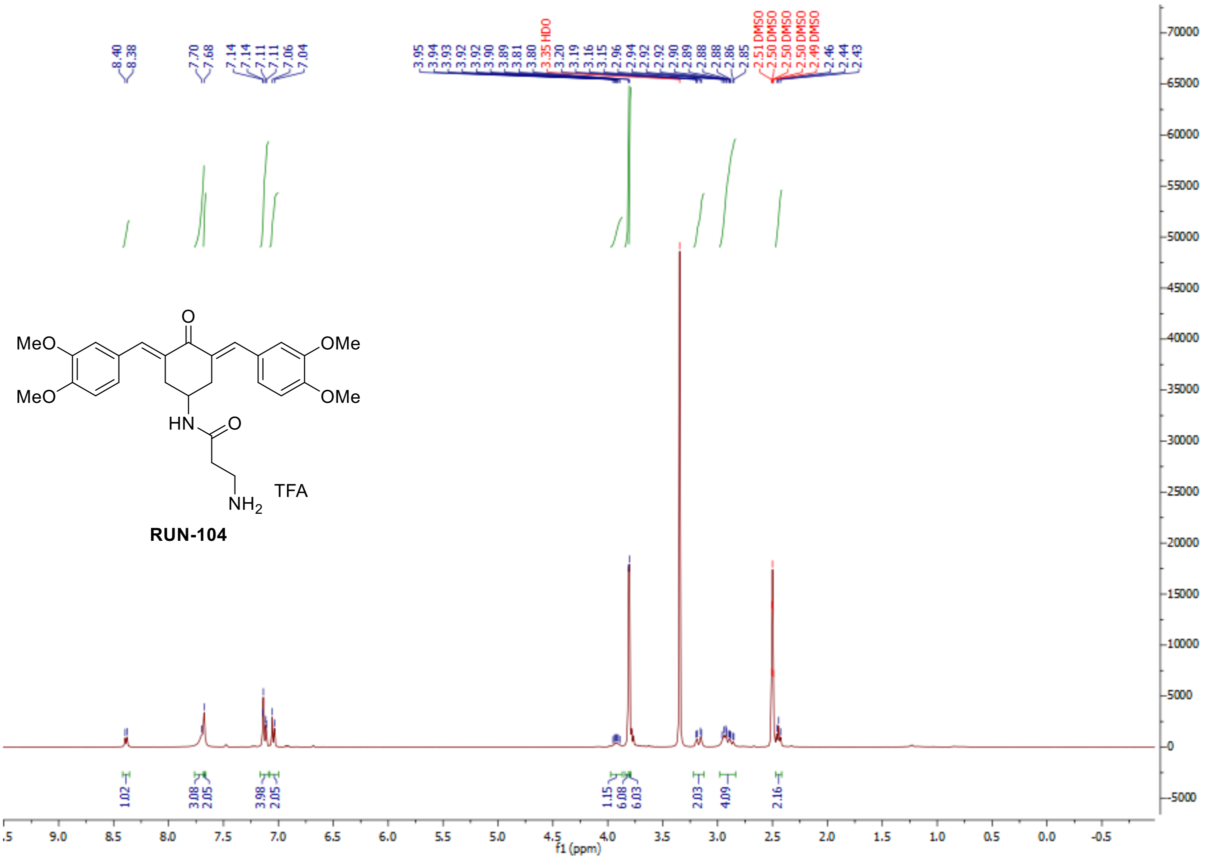
8.40
8.38
7.70
7.68
7.14
7.14
7.11
7.11
7.06
7.04
3.95
3.94
3.93
3.92
3.92
3.90
3.89
3.81
3.80
3.35 HDO
3.20
3.19
3.16
3.15
2.96
2.94
2.92
2.92
2.90
2.89
2.88
2.88
2.86
2.85
2.51 DMSO
2.50 DMSO
2.50 DMSO
2.50 DMSO
2.49 DMSO
2.46
2.44
2.43
MeO
MeO
O
OMe
OMe
HN
O
NH2
TFA
RUN-104
1.02
3.08
2.05
3.98
2.05
1.15
6.08
6.03
2.03
4.09
2.16
f1 (ppm)

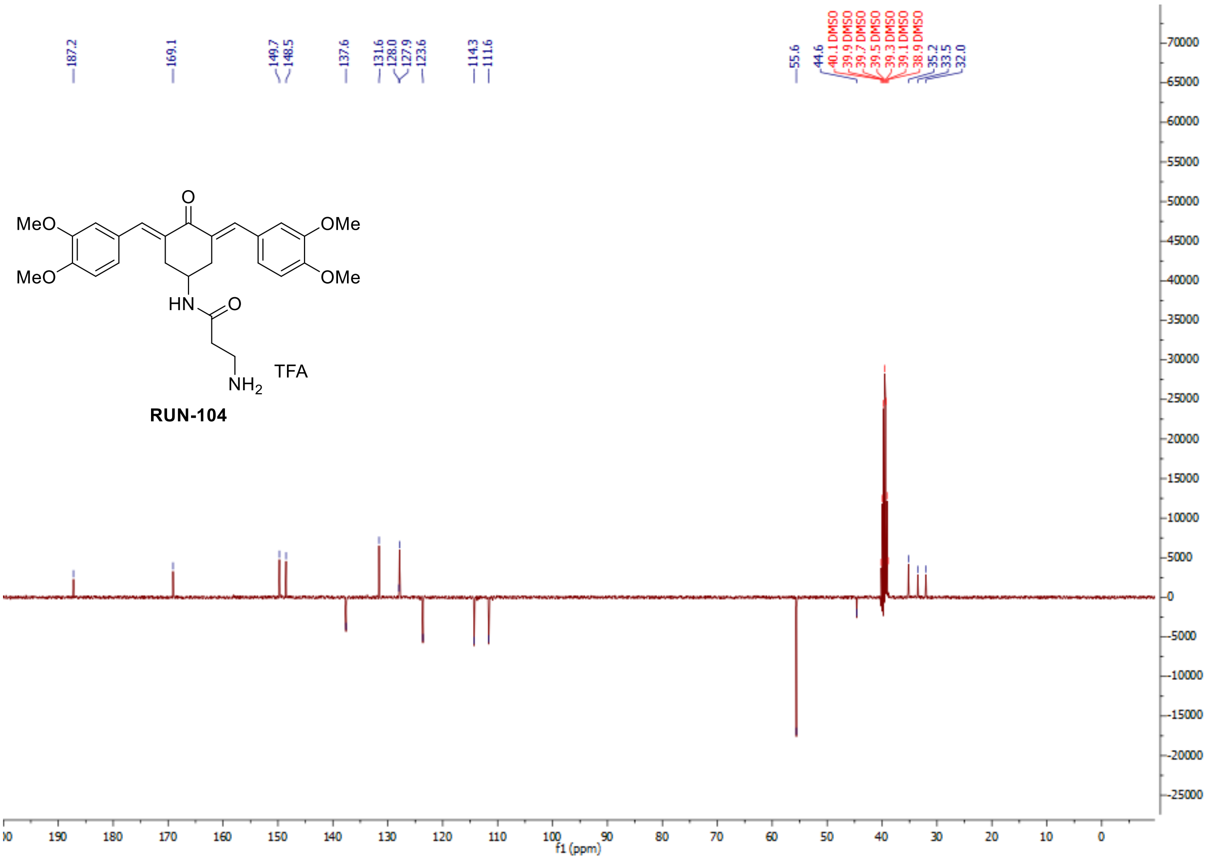
187.2
169.1
149.7
148.5
137.6
131.6
128.0
127.9
123.6
114.3
111.6
55.6
44.6
40.1 DMSO
39.9 DMSO
39.7 DMSO
39.5 DMSO
39.3 DMSO
39.1 DMSO
38.9 DMSO
35.2
33.5
32.0
MeO
MeO
O
OMe
OMe
HN
O
NH2
TFA
RUN-104
f1 (ppm)

4 References

1. Riss TL, Moravec RA, Niles AL, Duellman S, Benink HA, Worzella TJ, et al. Cell viability assays. Assay guidance manual [Internet]. 2016.
2. Fassmannová D, Sedlák F, Sedláček J, Špička I, Grantz Šašková K. Nelfinavir inhibits the TCF11/Nrf1-mediated proteasome recovery pathway in multiple myeloma. Cancers. 2020;12(5):1065.
3. Dyer BW, Ferrer FA, Klinedinst DK, Rodriguez R. A noncommercial dual luciferase enzyme assay system for reporter gene analysis. Analytical biochemistry. 2000;282(1):158-61.
4. Tannous BA. Gaussia luciferase reporter assay for monitoring biological processes in culture and in vivo. Nat Protoc. 2009;4(4):582-91.
5. Senchuk MM, Dues DJ, Van Raamsdonk JM. Measuring Oxidative Stress in Caenorhabditis elegans: Paraquat and Juglone Sensitivity Assays. Bio Protoc. 2017;7(1).
6. Wang E, Wink M. Chlorophyll enhances oxidative stress tolerance in Caenorhabditis elegans and extends its lifespan. PeerJ. 2016;4:e1879.
7. Hejdankova Z, Vanek V, Sedlak F, Prochazka J, Diederichs A, Kereïche S, et al. Lipid nanoparticles for broad‐spectrum nucleic acid delivery. Advanced Functional Materials. 2021;31(47):2101391.
8. Johnson KA, Biswas S, Weix DJ. Cross‐Electrophile Coupling of Vinyl Halides with Alkyl Halides. Chemistry–A European Journal. 2016;22(22):7399-402.
9. Aliabadi A, Shamsa F, Ostad SN, Emami S, Shafiee A, Davoodi J, et al. Synthesis and biological evaluation of 2-phenylthiazole-4-carboxamide derivatives as anticancer agents. Eur J Med Chem. 2010;45(11):5384-9.
